# Palladium-catalyzed direct arylation and cyclization of *o*-iodobiaryls to a library of tetraphenylenes

**DOI:** 10.1038/srep33131

**Published:** 2016-09-15

**Authors:** Chendan Zhu, Yue Zhao, Di Wang, Wei-Yin Sun, Zhuangzhi Shi

**Affiliations:** 1 State Key Laboratory of Coordination Chemistry, School of Chemistry and Chemical Engineering, Nanjing University, Nanjing, 210093, China

## Abstract

Aryl–aryl bond formation constitutes one of the most important subjects in organic synthesis. The recent developments in direct arylation reactions forming aryl–aryl bond have emerged as very attractive alternatives to traditional cross-coupling reactions. Here, we describe a general palladium-catalyzed direct arylation and cyclization of *o*-iodobiaryls to build a library of tetraphenylenes. This transformation represents one of the very few examples of C-H activation process that involves simultaneous formation of two aryl–aryl bonds. Oxygen plays a vital role by ensuring high reactivity, with air as the promoter furnished the best results. We anticipate this ligand-free and aerobic catalytic system will simplify the synthesis of tetraphenylenes as many of the reported methods involve use of preformed organometallic reagents and will lead to the discovery of highly efficient new direct arylation process.

Over the past decade, a number of methods have been developed for the synthesis of biaryl compounds^1^. The classical method involves the reaction of organometallic nucleophiles with a wide range of organo(pseudo)halides in presence of transition-metal catalysis^2^. Synthesis of such compounds without the aid of organometallics have drawn tremendous attention to the chemist. Substitution of the preactivated species with a simple arene as a nucleophile has become the most widely used mode of attack (Fig. 1, a-1)^3^,^4^,^5^,^6^,^7^,^8^,^9^,^10^,^11^,^12^,^13^. The most atom economic and attractive alternative is the dehydrogenative coupling of two aryl C–H bonds, neither of which requires preactivation of substrates (Fig. 1, a-2)^14^,^15^,^16^,^17^,^18^. Most recently, Weix *et al*. also developed reductive coupling of aryl bromides with aryl triflates *via* a multimetallic catalyzed process (Fig. 1, a-3)^19^. As a result of significant progress in biaryl synthesis, these green and efficient protocols have been applied extensively, particularly in construction and arylation of π-conjugated molecules with high selectivity^20^.

Flexible π-conjugated skeletons are the focus of considerable interest due to their dynamic molecular motions that can exhibit interesting molecule-based functions^21^,^22^,^23^. Tetraphenylene is one of the classical flexible π-conjugated molecules, in which four benzene rings are *ortho*-annelated to form an eight-membered ring at the center of the molecule (Fig. 1b)^24^. This unique structure characterizes tetraphenylene and their derivatives to be employed as liquid crystals^25^, molecular devices^26^, blue organic light-emitting diodes^27^, chiral ligands^28^,^29^ and key building blocks^30^. Traditional methods involving homocoupling of Grignard, zinc or lithium reagents derived from 2,2′-dihalobiaryls in presence of stoichiometric CuCl or NiCl_2_ provides the tetraphenylenes in low to moderate yields^31^,^32^. Additionally, dimerization of diphenylene^33^, [2 + 2 + 2] cycloaddition^34^ and Ullmann coupling^35^,^36^ reactions were optional methods to synthesize the tetraphenylene frameworks. In spite of these highly important pioneering studies, approaches based on C–H activation process in a general manner *via* transition-metal catalysis have remained underdeveloped. As part of our continuous effort on developing direct arylation procedure^37^,^38^, we decided to tackle a new synthetic route to simplify the synthesis of the eight-membered π-conjugated compounds. Here we have reported the first example of dual aryl-aryl bond formation to build a series of tetraphenylenes by a palladium-catalyzed direct arylation and cyclization process (Fig. 1c). Notably, a protocol has been developed that enables the use of readily accessible 2-iodobiaryls as the starting materials directly, which previously required the use of 2,2′-dihalobiaryls as the precursors^39^.

## Results and Discussion

**Figure 2:**
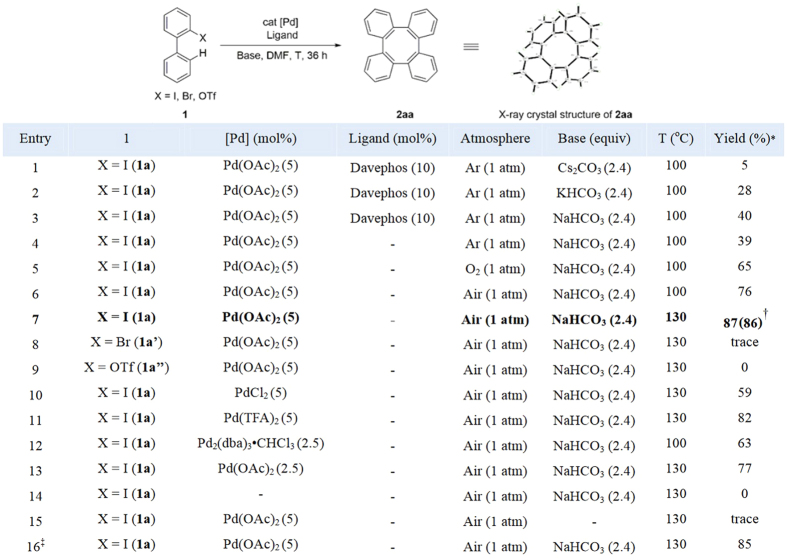
Reaction optimization. Reaction conditions: **1a** (0.4 mmol), [Pd] (5 mol %), ligand (10 mol %), base (2.4 equiv), Solvent (2 mL), 130 °C, 36 h, in air. *^1^H NMR yield using CH_2_Br_2_ as internal standard. ^†^Isolated yield. ^‡^In duck.

Initially we have evaluated the homo-coupling and cyclization of commercially available *o*-iodobiaryl (**1a**) (Fig. 2). By employing catalytic Pd(OAc)_2_ (5 mol%), 10 mol% of Davephos as the ligand and 2.0 equiv of Cs_2_CO_3_ as the base in DMF at 100 °C under argon, we indeed observed trace amount of **2aa** in GC-MS and crude ^1^H NMR (entry 1). Gratifyingly, the yield could be improved up to 40% by switching the base to KHCO_3_ and NaHCO_3_ (entry 2–3). Surprisingly, removal of the ligand maintained the similar reactivity and afforded the desired product in 39% yield (entry 4). This result indicated that the ligand might not be a driving force for this transformation. After extensive optimization, we have observed that the reaction atmosphere has an important effect and dioxygen atmosphere could significantly improve the yield (65%, entry 5). Notably, air atmosphere was superior for the process (entry 6)^40^,^41^,^42^. In addition, raising the reaction temperature to 130 °C under air, the desired product 2a was isolated in maximum yield (86%, entry 7). The use of 2-bromo-1,1′-biphenyl (**1a’**) and [1,1′-biphenyl]-2-yl trifluoromethanesulfonate (**1a”**) as the substrates provided lower yield of the desired product **2aa** (entries 8–9). Investigation with other palladium sources also exhibited inferior results (entries 10−12). Lowering of catalyst loading did not alter efficacy of the process (entry 13). Control experiments revealed that palladium and base were crucial for the transformation (entries 14−15). Finally, a light off experiment verified that the reaction was not promoted *via* superoxide radical pathway by visible-light irradiation (entry 16).

**Figure 3:**
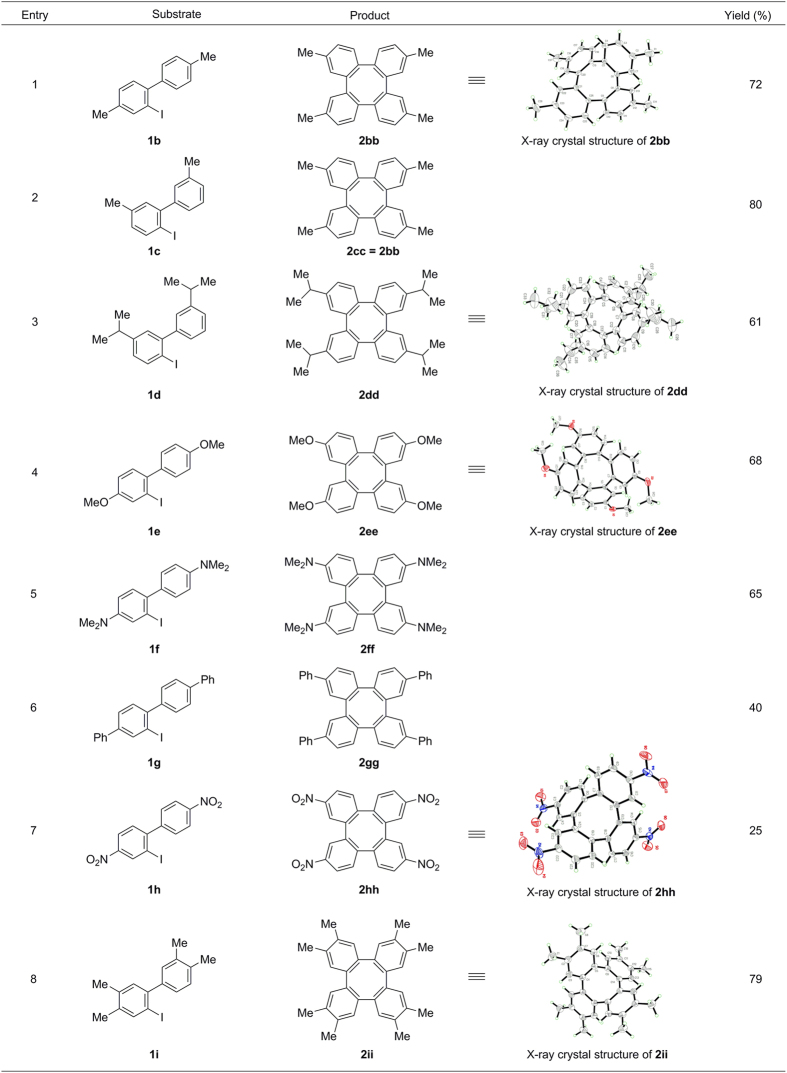
Substrate scope of homo-coupling and cyclization of *o*-iodobiaryls. Reaction conditions: **1** (0.4 mmol), Pd(OAc)_2_ (5 mol%), NaHCO_3_ (2.4 equiv), DMF (2 mL), 130 °C, 36 h, in air; The yields shown reflect the average of two isolation experiments.

With the optimum conditions in hand, we have extended the scope of this cyclization process (Fig. 3). 2-iodo-4,4′-dimethyl-1,1′-biphenyl (**1b**) afforded the desired product 2,7,10,15-tetramethyltetraphenylene (**2bb,** entry 1) in 72% yield. An interesting phenomenon is that 2-iodo-3′,5-dimethyl-1,1′-biphenyl (**1c**), an isomeric structure of **1b** also yield the same product **2bb** in 80% yield (Entry 2), due to its *D*_2_ symmetry structure. This result also indicated that the C-H activation position occurs at less sterically hindered position. It has been shown for a number of tetraphenylenes with bulky alkyl substituents show special optical, electrochemical and solution-state aggregation behaviour^43^. In the present investigation, we found that the substrate **1d** also worked effectively to provide bulky substituted tetraphenylene **2dd** (entry 3). Considering that the multi-methoxy substituted tetraphenylenes have been suggested to be potential applicant in the field of host-guest chemistry, molecular devices and liquid crystal meterials, we have synthesized the substrate **1e** and tested. Gratifyingly, the desired product **2ee** was obtained in 68% yield (entry 4). Moreover, a very electron-rich *N*^2^,*N*^2^,*N*^7^,*N*^7^,*N*^10^,*N*^10^,*N*^15^,*N*^15^-octamethyltetraphenylene-2,7,10,15-tetraamine (**2ff,** entry 5) was isolated in in 65% yield under the reaction condition. Large aromatic conjugated tetraphenylene **2gg** (entry 6), possessing special optical properties^44^ could also be generated from the *p*-quaterphenyl iodide **1g**. Electron-deficient substrate nitro (**1h,** entry 7) were also compatible for this process, albeit much lower reactivity. In addition, multi-substituted *o*-iodobiaryl **1i** reacted smoothly under the optimal conditions, affording the corresponding octamethyltetraphenylene**2ii** in good yield (entry 8).

**Figure 4:**
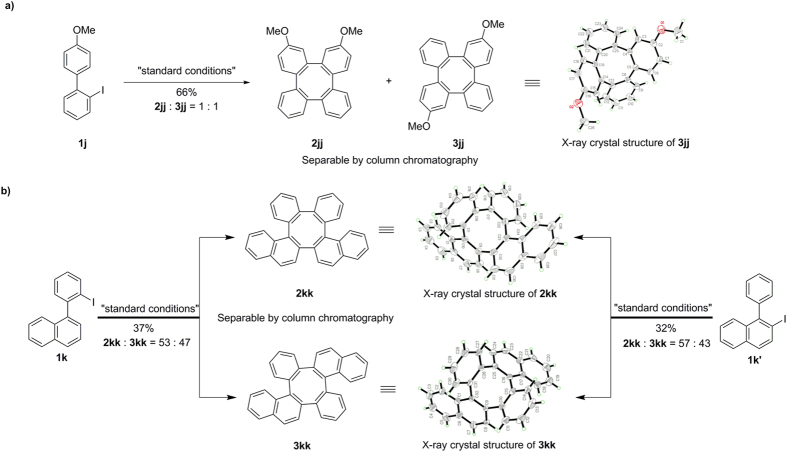
Synthesis, isolation and structural characterization of tetraphenylenes *via* homo-coupling and cyclization of unsymmetrical *o*-iodobiphenyls. Reaction conditions: **1** (0.4 mmol), Pd(OAc)_2_ (5 mol%), NaHCO_3_ (2.4 equiv), DMF (2 mL), 130 °C, 36 h, in air.

Differently ring substituted *o*-iodobiaryls were also tested to generate a novel synthetic entry to the mixed tetraphenylene derivatives in Fig. 4. The coupling of 2-iodo-4′-methoxy-1,1′-biphenyl (**1j**) under the standard conditions provided the regioisomeric tetraphenylenes **2jj** and **3jj** (1:1) and were collected in 66% combined yield as they were inseparable. Recrystallization of the mixture afforded **3jj** as a single isomer and it was characterized by X-ray crystal structure analysis (Fig. 4a). Subsequently we have carried out coupling of the isomers 1-(2-iodophenyl)naphthalene (**1k**) and 2-iodo-1-phenylnaphthalene (**1k’**). Both isomers produced mixed tetraphenylene derivatives with different selectivity (Fig. 4b). The isomers **2kk** and **3kk** were separated by column chromatography on silica and subsequently each isomer was subjected to recrystallization and finally confirmed *via* X-ray crystal structure analysis.

**Figure 5:**
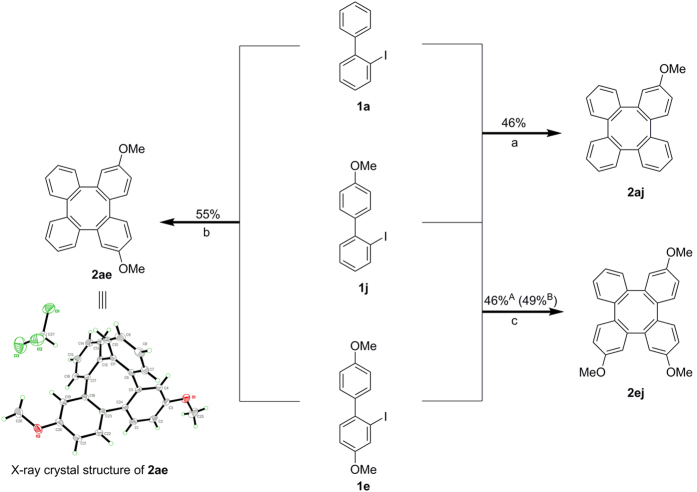
Synthesis, isolation and structural characterization of mono-, di-, tri-methoxy substituted tetraphenylenes *via* cross-coupling and cyclization of *o*-iodobiphenyls. Reaction conditions: (a) **1a** (0.4 mmol), **1j** (0.2 mmol), Pd(OAc)_2_ (5 mol%), NaHCO_3_ (2.4 equiv), DMF (2 mL), 130 °C, 36 h, in air; (b) **1a** (0.4 mmol), **1e** (0.2 mmol), Pd(OAc)_2_ (5 mol%), NaHCO_3_ (2.4 equiv), DMF (2 mL), 130 °C, 36 h, in air; (c) Method A: **1e** (0.4 mmol), **1j** (0.2 mmol), Pd(OAc)_2_ (5 mol%), NaHCO_3_ (2.4 equiv), DMF (2 mL), 130 °C, 36 h, in air; Method B: **1e** (0.2 mmol), **1j** (0.4 mmol), Pd(OAc)_2_ (5 mol%), NaHCO_3_ (2.4 equiv), DMF (2 mL), 130 °C, 36 h, in air.

To deliver structures with greater synthetic value, we sought to develop the cross-coupling of two different *o*-iodobiaryls (Fig. 5). The 2-iodobiphenyl (**1a**) was reacted with **1j** to synthesize the cross-coupled product, 2-methoxytetraphenylene (**2aj**) as main product in 46% yield. The byproducts involving the homo-coupling product of **1a** and **1j** can then be removed easily by column chromatography. Additionally, we have synthesized 2,7-dimethoxytetraphenylene (**2ae**) and 2,7,10-trimethoxytetraphenylene (**2ej**) under the same reaction conditions. In combination with homo- and cross-coupling, tetraphenylenes with mono- to tetra-substituted methoxy groups can be generated easily.

**Figure 6:**
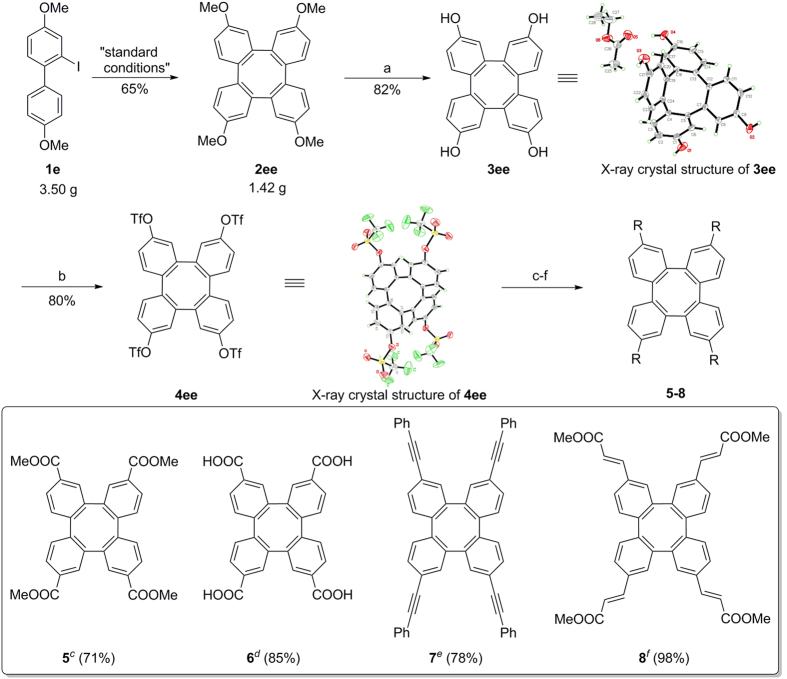
Gram-scale transformation and diversification of 2ee. Reaction conditions: **1e** (10.3 mmol), Pd(OAc)_2_ (5 mol%), NaHCO_3_ (2.4 equiv), DMF (50 mL), 130 °C, 36 h, in air; (a) BBr_3_, DCM, −78 °C to rt; (b) Tf_2_O, Py, DCM, 0 °C to rt; (c) Pd(OAc)_2_ (20 mol%), dppf (80 mol%), KOAc (16.0 equiv), DMSO/MeOH, CO (1 atm), 60 °C; (d) Pd(OAc)_2_ (20 mol%), dppf (80 mol%), KOAc (16.0 equiv), DMSO, CO (1 atm), 60 °C; (e) Pd(OAc)_2_ (12 mol%), PPh_3_ (48 mol%), Phenylacetylene (6.0 equiv), K_3_PO_4_ (4.8 equiv), DMSO, 80 °C; (f) Pd(OAc)_2_ (20 mol%), dppp (22 mol%), Methyl acrylate (40.0 equiv), Et_3_N (8.0 equiv), DMF, 115 °C.

A major benefit of this mild cyclization procedure is its amenability to gram-scale applications (Fig. 6). Under the standard conditions, gram-scale synthesis of **2ee** without notable erosion of yield proved the practicality of this new method. Synthetic utility of the tetraphenylenes derivatives also increases the importance of these classes of compounds. The demethylation of **2ee** with boron tribromide generated tetraphenylene-2,7,10,15-tetraol (**3ee,** 82%), which can be readily converted into the triflate **4ee** in presence of triflic anhydride and pyridine. Both the structures **3ee** and **4ee** were confirmed by X-ray crystal structure analysis. Using tetraphenylene-2,7,10,15-tetrayl tetrakis(trifluoromethanesulfonate) (**4ee**), a range of useful tetraphenylene derivatives can be prepared (**5**–**8**, Fig. 6) by palladium catalyst. Methoxycarbonylation of **4ee** with methanol in CO atmosphere (1 atm) proceeded smoothly to afford ester **5** in 71% yield. Notably, the free carboxylic acid **6** was also directly accessible in 85% yield in the absence of methanol using the same reaction condition. In addition, palladium-catalyzed Sonogashira and Heck Coupling of **4ee** with phenylacetylene and methyl acrylate afforded π-extended tetraphenylenes, **7**–**8** respectively in excellent yields. With a wide variety of these functionalized tetraphenylenes in hand, we can predict boldly that they have potential applications in many chemistry and material field such as rapid construction of a class of metal–organic frameworks (MOFs)^45^.

### Mechanism

**Figure 7:**
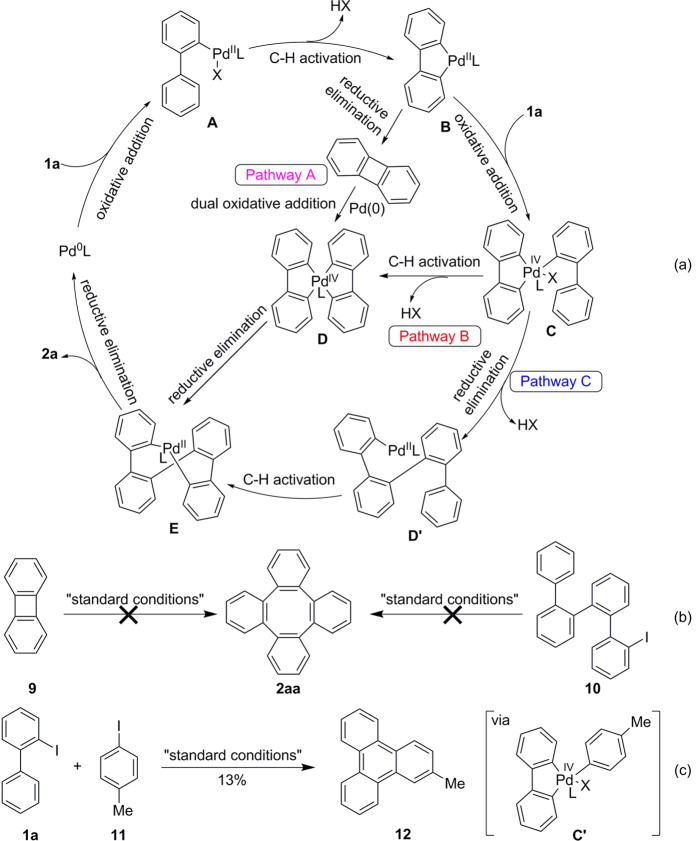
Proposed mechanism and the supporting mechanistic studies. (**a**) Proposed mechanism. (**b**) Mechanistic experiments. (**c**) Cross-coupling reaction. Reaction conditions: **1a** (0.2 mmol), **11** (0.2 mmol), Pd(OAc)_2_ (5 mol%), NaHCO_3_ (2.4 equiv), DMF (2 mL), 130 °C, 36 h, in air.

Several transition-metal complexes including Ni^46^, Pt^47^ and Pd^48^ can catalyze the formation of tetraphenylene from biphenylene (**9**) *via* C-C bond cleavage. Based on these results, initially we thought that compound **9** might be the key intermediate in this transformation. As shown in Fig. 7, oxidative addition of **1**a to a Pd(0) species appears to trigger the reaction to afford the intermediate **A**, which undergoes activation of a neighboring C-H bond to produce a five membered palladacycle **B**^49^. This intermediate has the possibility to undergo reductive elimination forming a biphenylene product **9**. According to the literature, the Pd(0) can catalyze the dimerization of **9** *via* intermediate **D**^50^ and **E** to produce the final product **2aa** (pathway A). However, when substrate **9** was subjected to our standard conditions, the corresponding product **2aa** was not observed (Fig. 7b, left). This clearly rules out the possibility of the pathway A. The second possible pathway is the oxidative addition of palladacycle **B** with another substrate molecule to generate **C**, which can undergo a second C-H activation providing the spiropalladacycle **D**. Finally, two successive reductive elimination of **D** generates the product **2aa** and the Pd(0) species (pathway B). The third possibility involves reductive eliminationof **C** to form an intermediate **D**’ ahead of the second C-H activation. Due to the configuration of **D**’, the second C-H activation can occur at the remote position to formintermediate **E**, which the convert to the tetraphenylene **2aa**. To find out the best possible intermediate **D** or **D**’, we employed compound **10** under the standard conditions and did not observe the desired cyclization product **2aa** (Fig. 7b, right). This demonstrates that intermediate **D**’ can’t undergo remote C-H activation to form the product. Considering these results, we have proposed pathway **B** to be the most favorable at the current stage. In view of the active role of oxygen molecule in the present transformation, mores studies are required to fully elucidate the reaction pathway. Notably, employing two iodoarene substrates such as *o*-iodobiphenyl (**1a**) and 1-iodo-4-methylbenzene (**11**) in our catalytic system, the cross-coupling and cyclization product **12**^51^ was formed in 13% yield *via* intermediate **C**’ along with 65% yield of homo-coupling product **2aa** (Fig. 7c). This transformation between two different aryl electrophiles can further prove that the pathway **B** is reasonable and the discovery of new transformation based on this chemistry is feasible.

## Conclusion

In combination with the known modes for aryl–aryl bond formation, we have now reported an efficient method to build two aryl–aryl bonds simultaneously *via* direct C–H arylations. This reaction sequence involves rupture of two C-H bonds and two C-I bonds, as well as the formation of an eight-membered ring. This palladium-catalyzed reaction represents as one of the few efficient methods for the formation of flexible π-expanded cyclooctatetraenes. Undoubtedly, this development will help both synthetic and material chemists making this direct arylation as a valuable tool for easy synthesis of various tetraphenylenes. In addition, more than ten structures of tetraphenylene derivatives were observed by X-ray crystal analysis. All these compounds show the saddle-shaped structures wherein the central cyclooctatetraene (COT) parties display the tub conformation (see the supporting information). In the laboratory, more investigations are going on to get insight the reaction mechanism and to apply this type of reactivity profile in other processes.

## Methods

### General procedure for tetraphenylene synthesis

To a 10 mL Schlenk flask equipped with a magnetic stir bar was charged with Pd(OAc)_2_ (2.3 mg, 0.01 mol, 5 mol%), *o*-iodobiaryls (0.40 mmol, 2.0 equiv), NaHCO_3_ (40 mg, 0.48 mmol, 2.4 equiv) and 2 mL DMF. Then the mixture was stirred at 130 °C under air for 36 h. After cooling to room temperature, the solvent was removed under reduce pressure. The crude product was further purified by column chromatography on silica gel. Full experimental details and characterization of new compounds can be found in the Supplementary Methods.

### Data Availability

 Crystallographic data for the structural analysis of the compounds have been deposited with the Cambridge Crystallographic Data Centre, under CCDC no. 1491922 (**2aa**), 1491929 (**2bb**), 1491930 (**2dd**), 1491931 (**2ee**), 1491932 (**2hh**), 1491937 (**2ii**), 1491938 (**3jj**), 1491939 (**2kk**), 1491940 (**3kk**), 1491941 (**2ae**), 1491942 (**3ee**), and 1491948 (**4ee**). These data can be obtained free of charge from The Cambridge Crystallographic Data Centre via www.ccdc.cam.ac.uk/data_request/cif.

## Additional Information

**How to cite this article**: Zhu, C. *et al*. Palladium-catalyzed direct arylation and cyclization of *o*-iodobiaryls to a library of tetraphenylenes. *Sci. Rep.* **6**, 33131; doi: 10.1038/srep33131 (2016).

## Supplementary Material

Supplementary Information

## Figures and Tables

**Figure 1:**
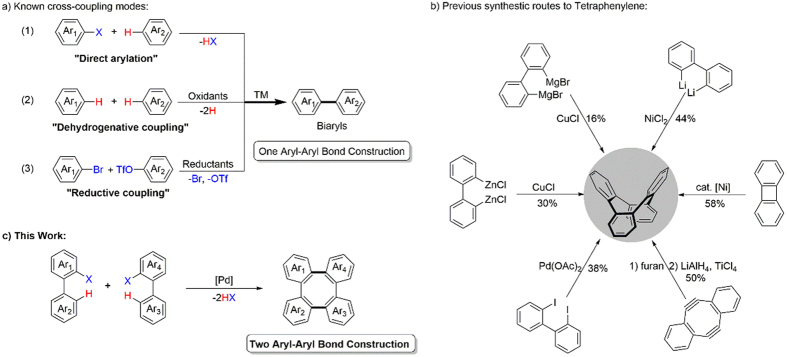
The attempted synthesis of tetraphenylene inspired from modern aryl-aryl bond construction. (**a**) Transition-metal-catalyzed coupling reactions without the aid of organometallic reagents. (**b**) Known progress toward tetraphenylene synthesis. (**c**) New route to tetraphenylene construction.
