# Supplementary material for: Palladium-catalyzed direct arylation and cyclization of o-iodobiaryls to a library of tetraphenylenes
Source: Sci Rep. 2016 Sep 15;6:33131. doi: 10.1038/srep33131 (PMC5024091; doi:10.1038/srep33131)
Supplement: Supplementary Information [file srep33131-s1.pdf]

Palladium-catalyzed direct arylation and cyclization of *o*-iodobiaryls to a library of tetraphenylenes

(Supporting Information)

Chendan Zhu, Yue Zhao, Di Wang, Wei-Yin Sun and Zhuangzhi Shi*

State Key Laboratory of Coordination Chemistry, School of Chemistry and Chemical Engineering, Nanjing University, Nanjing, 210093, China

**e-mail:* shiz@nju.edu.cn

Table of Contents

1. General information. 2

2. General procedure for synthesis of starting materials....................2

4. General procedure for tetraphenylene synthesis....................9

5. Cross-coupling and cyclization of two different o-iodobiaryls 15

6. Gram-scale transformation and diversification of **2ee**....................18

7. Mechanism insights 22

8. References....................25

9. X-ray crystallographic date and analysis 26

10. ^{1}H, ^{13}C and ^{19}F NMR spectra of substrates and products 52

1. General information.

All new compounds were fully characterized. NMR-spectra were recorded on Bruker ARX-400 MHz. Mass spectra were conducted at Micromass Q-Tof instrument (ESI) and Agilent Technologies 5973N (EI). IR spectra were recorded on a FT-IR spectrometer. All reactions were carried out open in air. Unless otherwise noted, materials obtained from commercial suppliers were used without further purification. 2-Iodo-1,1'-biphenyl (**1a**) was purchased from Alfa Aesar.

2. General procedure for synthesis of starting materials[1]

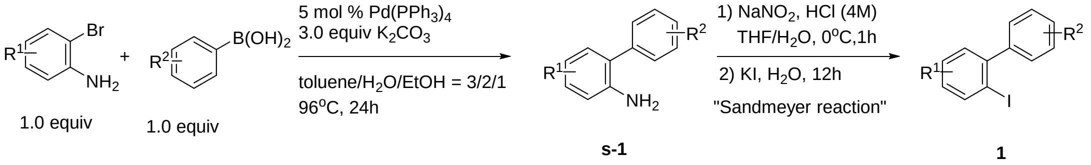

To a stirred solution of substituted phenylboronic acid (6.0 mmol, 1.0 equiv) and 2-bromoaniline (6.0 mmol, 1.0 equiv) in toluene/H_2O/EtOH (30/20/10 mL) was added K_3PO_4 (18 mmol, 3.0 equiv), $Pd(PPh_3)_4$ (0.3 mmol, 5 mol%). The mixture was stirred at 96 ^oC for 24 h under argon atmosphere. After cooling, the organic phase was separated and the aqueous phase was extracted with EtOAc. The combined organic phases were washed with brine and dried over $MgSO_4$. The solvent was evaporated under reduced pressure and purified by column chromatography to afford the corresponding [1,1'-biphenyl]-2-amine (**s-1**).

To a stirred solution of corresponding **s-1** (3.0 mmol, 1.0 equiv) in THF/H_2O (4/8 mL) was added 4 M aqueous HCl (3.6 mL), and the solution was cooled in an ice water bath. A solution of $NaNO_2$ (310 mg, 4.5 mmol, 1.5 equiv) in H_2O (1 mL) was added dropwise for 20 min and stirred for 1 h at the same temperature. A solution of KI (1.2 g, 7.2 mmol, 2.4 equiv) in H_2O (1 mL) was added dropwise. The reaction mixture was stirred for 5 min in the ice water bath, then slowly warmed up to r.t. and stirred overnight. DCM and H_2O was added to the mixture and the organic phase was separated. The aqueous phase was extracted with DCM. The combined organic phases were treated with aqueous NaOH (33 %, 5mL) and stirred for 5 min before a more

part of H_2O was added. The organic phase was isolated and dried over $MgSO_4$. The corresponding *o*-halobiaryl was purified by column chromatography on silica gel.

2-Iodo-4,4'-dimethyl-1,1'-biphenyl (1b)

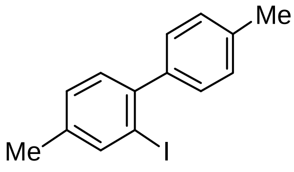

1b

Following general procedure, 4,4'-dimethyl-[1,1'-biphenyl]-2-amine (**s-1b**) (1.12 g, 95%) was prepared from 2-bromo-5-methylaniline (1.116 g, 6.0 mmol) and *p*-tolylboronic acid (816 mg, 6.0 mmol) as white solid. Using 3.0 mmol **s-1b** under the Sandmeyer reaction conditions, the substarte **1b** was obtained as colorless oil in 86% yield (795 mg). **^{1}H NMR (400 MHz, $CDCl_3$)** δ 7.80 (s, 1H), 7.24 (s, 4H), 7.19 (s, 2H), 2.42 (s, 3H), 2.36 (s, 3H). **^{13}C NMR (101 MHz, $CDCl_3$)** δ 143.65, 141.18, 139.87, 138.62, 137.17, 129.74, 129.21, 128.93, 128.58, 98.63, 21.27, 20.42. **ATR-FTIR (cm^{-1}):** 3022, 2919, 2864, 1600, 1474, 1380, 810, 544. **EI-MS (m/z, relative intensity):** 309 (M^+, 20), 308 (100), 166 (68), 165 (74), 89 (16).

2-Iodo-3',5-dimethyl-1,1'-biphenyl (1c)

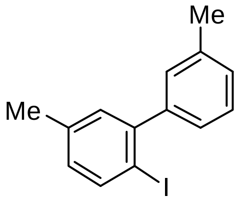

1c

Following general procedure, 3',5-dimethyl-[1,1'-biphenyl]-2-amine (**s-1c**) (1.067 g, 90%) was prepared from 2-bromo-4-methylaniline (1.116 g, 6.0 mmol) and *m*-tolylboronic acid (816 mg, 6.0 mmol) as brown oil. Using 3.0 mmol **s-1c** under the Sandmeyer reaction conditions, the substarte **1c** was obtained as pale yellow oil in 94% yield (868 mg). **^{1}H NMR (400 MHz, $CDCl_3$)** δ 7.81 (d, J = 8.1 Hz, 1H), 7.34 – 7.28 (m, 1H), 7.23 – 7.18 (m, 1H), 7.18 – 7.11 (m, 3H), 6.89 – 6.83 (m, 1H), 2.42 (s, 3H), 2.34 (s, 3H). **^{13}C NMR (101 MHz, $CDCl_3$)** δ 146.46, 144.13, 139.16, 138.01, 137.48, 131.00, 129.89, 129.65, 128.21, 127.73, 126.31, 94.43, 21.45, 20.86. **ATR-FTIR (cm^{-1}):** 3032, 2923, 2859, 1623, 1545, 1395, 797, 702. **EI-MS (m/z, relative intensity):** 309 (M^+, 22), 308 (100), 166 (68), 165 (74), 89 (16).

2-Iodo-3',5-diisopropyl-1,1'-biphenyl (1d)

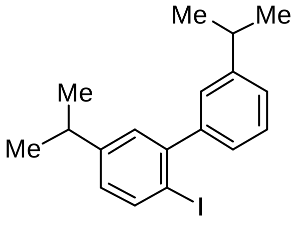

1d

Following general procedure, 3',5-diisopropyl-[1,1'-biphenyl]-2-amine (**s-1d** (1.366 g, 90%) was prepared from 2-bromo-4-isopropylaniline (1.284 g, 6.0 mmol) and (3-isopropylphenyl)boronic acid (984 mg, 6.0 mmol) as white solid. Using 3.0 mmol **s-1d** under the Sandmeyer reaction conditions, the substarte **1d** was obtained as colorless oil in 83% yield (906 mg). **^{1}H NMR (400 MHz, CDCl$_3$)** δ 7.85 (d, *J* = 8.2 Hz, 1H), 7.37 – 7.31 (m, 1H), 7.26 – 7.22 (m, 2H), 7.19 (d, *J* = 2.2 Hz, 1H), 7.17 – 7.13 (m, 1H), 6.91 (dd, *J* = 8.2, 2.3 Hz, 1H), 3.04 – 2.93 (m, 1H), 2.93 – 2.82 (m, 1H), 1.30 (d, *J* = 6.9 Hz, 6H), 1.25 (d, *J* = 6.9 Hz, 6H). **^{13}C NMR (101 MHz, CDCl$_3$)** δ 149.10, 148.29, 146.65, 144.19, 139.32, 128.50, 127.83, 127.75, 127.00, 126.56, 125.62, 94.95, 34.10, 33.71, 23.95, 23.83. **ATR-FTIR (cm^{-1}):** 3047, 2963, 2874, 1597, 1460, 1391, 804, 707. **EI-MS (m/z, relative intensity):** 365 (M$^+$, 22), 364 (100), 350 (17), 349 (98), 179 (46), 178 (34), 167 (22), 165 (22).

2-Iodo-4,4'-dimethoxy-1,1'-biphenyl (1e)

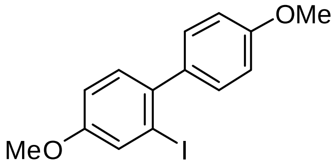

1e

Following general procedure, 4,4'-dimethoxy-[1,1'-biphenyl]-2-amine (**s-1e**) (1.25 g, 91%) was prepared from 2-bromo-5-methoxylaniline (1.212 g, 6.0 mmol) and (4-methoxyphenyl)boronic acid (912 mg, 6.0 mmol) as white soild. Using 3.0 mmol **s-1e** under the Sandmeyer reaction conditions, the substarte **1e** was obtained as white solid in 83% yield (843 mg). **^{1}H NMR (400 MHz, CDCl$_3$)** δ 7.48 (t, *J* = 2.1 Hz, 1H), 7.25 (dd, *J* = 8.0, 2.1 Hz, 2H), 7.19 (dd, *J* = 8.5, 1.1 Hz, 1H), 6.96 – 6.91 (m, 3H), 3.86 (s, 3H), 3.82 (s, 3H). **^{13}C NMR (101 MHz, CDCl$_3$)** δ 158.86, 158.71, 138.82, 136.36, 130.64, 130.27, 124.39, 114.22, 113.19, 99.03, 55.55, 55.24. **ATR-FTIR (cm^{-1}):** 2932, 1597, 1472, 1235, 1025, 804, 549. **EI-MS (m/z, relative intensity):** 341 (M$^+$, 18), 340 (100), 325 (24), 198 (36), 170 (18), 155 (18), 127 (17).

2-Iodo-4,4'-dinitro-1,1'-biphenyl (1h)

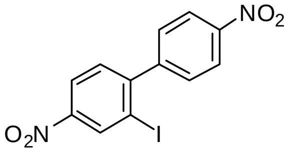

1h

Following general procedure, 4,4'-dinitro-[1,1'-biphenyl]-2-amine (**s-1h**) (1.13 g, 73%) was prepared from 2-bromo-5-nitroaniline (1.30 g, 6.0 mmol) and (4-nitrophenyl)boronic acid (1.00 g, 6.0 mmol) as brown solid. Using 3.0 mmol **s-1h** under the Sandmeyer reaction conditions, the substarte **1h** was obtained as pale yellow solid in 80% yield (888 mg). **^{1}H NMR (400 MHz, $CDCl_3$)** δ 8.83 (d, J = 2.2 Hz, 1H), 8.37 – 8.32 (m, 2H), 8.30 (dd, J = 8.4, 2.3 Hz, 1H), 7.56 – 7.51 (m, 2H), 7.47 (d, J = 8.4 Hz, 1H). **^{13}C NMR (101 MHz, $CDCl_3$)** δ 150.58, 148.24, 147.92, 147.62, 134.61, 130.00, 123.69, 123.27, 97.02. **ATR-FTIR (cm^{-1}):** 1630, 1514, 1398, 1397, 1338, 1113, 844. **EI-MS (m/z, relative intensity):** 371 (M^+, 14), 370 (100), 278 (21), 151 (59), 150 (59), 139 (32), 75 (16).

2-Iodo-3',4,4',5-tetramethyl-1,1'-biphenyl (1i)

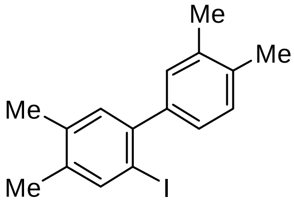

1i

Following general procedure, 3',4,4',5-tetramethyl-[1,1'-biphenyl]-2-amine (**s-1i**) (1.16 g, 86%) was prepared from 2-bromo-4,5-dimethylaniline (1.20 g, 6.0 mmol) and (3,4-dimethylphenyl)boronic acid (900 mg, 6.0 mmol) as brown soild. Using 3.0 mmol **s-1i** under the Sandmeyer reaction conditions, the substarte **1i** was obtained as white solid in 85% yield (857 mg). **^{1}H NMR (400 MHz, $CDCl_3$)** δ 7.71 (s, 1H), 7.17 (d, J = 7.6 Hz, 1H), 7.12 – 7.05 (m, 3H), 2.31 (s, 6H), 2.25 (s, 3H), 2.23 (s, 3H). **^{13}C NMR (101 MHz, $CDCl_3$)** δ 144.05, 141.60, 140.02, 137.42, 136.68, 136.02, 135.70, 131.35, 130.47, 129.08, 126.72, 94.77, 19.82, 19.57, 19.30, 18.85. **ATR-FTIR (cm^{-1}):** 2924, 2861, 1631, 1443, 1392, 1120, 877, 817. **EI-MS (m/z, relative intensity):** 337 (M^+, 22), 336 (100), 194 (56), 179 (50), 178 (30).

2-Iodo-4'-methoxy-1,1'-biphenyl (1j)

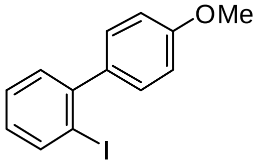

1j

Following general procedure, 4'-methoxy-[1,1'-biphenyl]-2-amine (**s-1j**) (1.12 g, 94%) was prepared from 2-bromoaniline (1.032 g, 6.0 mmol) and (4-methoxyphenyl)boronic acid (912 mg, 6.0 mmol) as white soild. Using 3.0 mmol **s-1j** under the Sandmeyer reaction conditions, the substarte **1j** was obtained as white solid in 92% yield (856 mg). **^{1}H NMR (400 MHz, $CDCl_3$)** δ 7.94 (dd, J = 7.9, 0.9 Hz, 1H), 7.40 – 7.33 (m, 1H), 7.32 – 7.23 (m, 3H), 7.04 – 6.92 (m, 3H), 3.86 (s, 3H). **^{13}C NMR (101 MHz, $CDCl_3$)** δ 159.05, 146.22, 139.45, 136.71, 130.40, 130.18, 128.49, 128.09, 113.26, 99.17, 55.25. **ATR-FTIR (cm^{-1}):** 2926, 2836, 1614, 1509, 1452, 1242, 1025, 753. **EI-MS (m/z, relative intensity):** 311 (M^+, 20), 310 (100), 168 (48), 140 (34), 139 (52).

1-(2-Iodophenyl)naphthalene (1k)

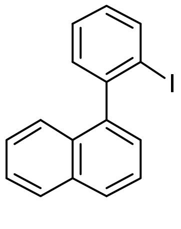

1k

Following general procedure, 2-(naphthalen-1-yl)aniline (**s-1k**) (920 mg, 70%) was prepared from 2-bromoaniline (1.032 g, 6.0 mmol) and naphthalen-1-ylboronic acid (1.032 g, 6.0 mmol) as brown soild. Using 3.0 mmol **s-1k** under the Sandmeyer reaction conditions, the substarte **1k** was obtained as white solid in 82% yield (812 mg). **^{1}H NMR (400 MHz, $CDCl_3$)** δ 8.02 (dd, J = 8.0, 1.0 Hz, 1H), 7.93 (d, J = 8.2 Hz, 2H), 7.58 – 7.41 (m, 5H), 7.38 – 7.31 (m, 2H), 7.14 (td, J = 7.8, 1.7 Hz, 1H). **^{13}C NMR (101 MHz, $CDCl_3$)** δ 145.36, 142.22, 139.08, 133.43, 131.39, 130.86, 129.05, 128.22, 128.14, 127.94, 126.90, 126.11, 125.96, 125.89, 125.16, 100.57. **ATR-FTIR (cm^{-1}):** 3053, 1631, 1396, 1258, 1112, 1017, 762. **EI-MS (m/z, relative intensity):** 331 (M^+, 14), 330 (74), 203 (100), 202 (98), 201 (26), 200 (28), 101 (31).

2-Iodo-1-phenylnaphthalene (1k')

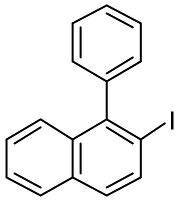

1k'

Following general procedure, 1-phenylnaphthalen-2-amine (**s-1k'**) (920 mg, 70%) was prepared from 1-bromonaphthalen-2-amine[2] (1.332 g, 6.0 mmol) and phenylboronic acid (732 mg, 6.0 mmol) as brown solid. Using 3.0 mmol **s-1k'** under the Sandmeyer reaction conditions, the substarte **1k'** was obtained as colorless oil in 75% yield (742 mg). **^{1}H NMR (400 MHz, $CDCl_3$)** δ 7.98 (d, *J* = 8.7 Hz, 1H), 7.85 (d, *J* = 8.1 Hz, 1H), 7.58 (d, *J* = 8.7 Hz, 1H), 7.56 – 7.53 (m, 1H), 7.53 – 7.47 (m, 3H), 7.44 – 7.39 (m, 1H), 7.38 – 7.32 (m, 1H), 7.30 – 7.24 (m, 2H). **^{13}C NMR (101 MHz, $CDCl_3$)** δ 144.43, 143.23, 135.46, 133.33, 132.80, 129.94, 128.96, 128.39, 127.84, 127.83, 127.15, 126.71, 126.18, 98.34. **ATR-FTIR (cm^{-1}):** 1630, 1397, 1117, 1028, 803. **EI-MS (m/z, relative intensity):** 331 (M^+, 19), 330 (100), 203 (58), 202 (91), 201 (24), 200 (26), 101 (60).

2-Iodo-N^4,N^4,$N^{4'}$,$N^{4'}$-tetramethyl-[1,1'-biphenyl]-4,4'-diamine (1f) [3]

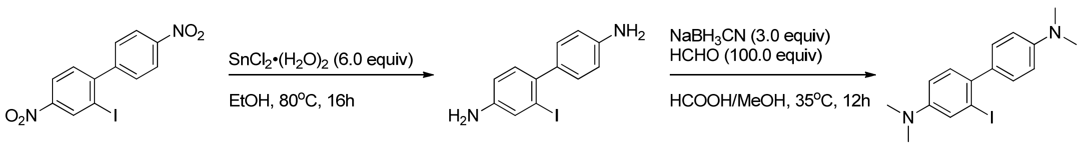

1i　　s-1f (98%)　　1f

1i (1.11 g, 3.0 mmol, 1.0 equiv) and $SnCl_2 \cdot (H_2O)_2$ (4.5 g, 18 mmol, 6.0 equiv) were dissolved in EtOH (7.5 mL) under argon and stirred at 80 °C. The reaction was monitored by TLC until complete conversion and the reaction mixture was allowed to reach room temperature after 60 minutes. The reaction mixture was then poured over ice and $NaHCO_3$ was added to reach pH 8. EtOAc was then added and product was extracted with EtOAc in brine and water. Organic layers were dried over Na_2SO_4, filtered, and concentrated to give the crude product **s-1f** as brown oil (911 m g, 98%).

To a solution of **s-1f** (911 mg, 2.9 mmol, 1.0 equiv) in MeOH (15 mL) under Ar were added formic acid to adjust pH to 3 - 4. $NaBH_3CN$ (188 mg, 9.0 mmol, 3.0 equiv) was added to the reaction mixture and stirred for 10 min at room temperature. The mixture was cooled in a ice-water bath, and formaldehyde (10.8 mL, 300 mmol, 100

equiv) was added slowly via a syringe. The mixture was allowed to warm to 35 °C and stirred under Ar for 12 h. The resulting mixture was concentrated and the solution of K_2CO_3(5 M) was added to pH ≈ 12. The aqueous layer was extracted with DCM. The combined organic layer was dried over Na_2SO_4. The filtrate was concentrated and **1f** was purified by column chromatography on silica gel (DCM: MeOH = 20 : 1) as yellow solid (699 mg, 65%). **^{1}H NMR (400 MHz, CDCl$_3$)** δ 7.27 – 7.21 (m, 3H), 7.14 (d, *J* = 8.5 Hz, 1H), 6.77 – 6.72 (m, 3H), 2.99 (s, 6H), 2.95 (s, 6H). **^{13}C NMR (101 MHz, CDCl$_3$)** δ 149.98, 149.37, 134.71, 130.35, 130.06, 122.93, 112.49, 111.59, 100.42, 40.56, 40.51. **ATR-FTIR (cm^{-1}):** 1602, 1491, 1437, 1399, 1342, 1120, 806. **HRMS m/z (ESI):** calcd for $C_{14}H_{13}IN_2H^+$ 367.0666, found 367.0667.

2''-Iodo-1,1':4',1'':4'',1'''-quaterphenyl (1g)

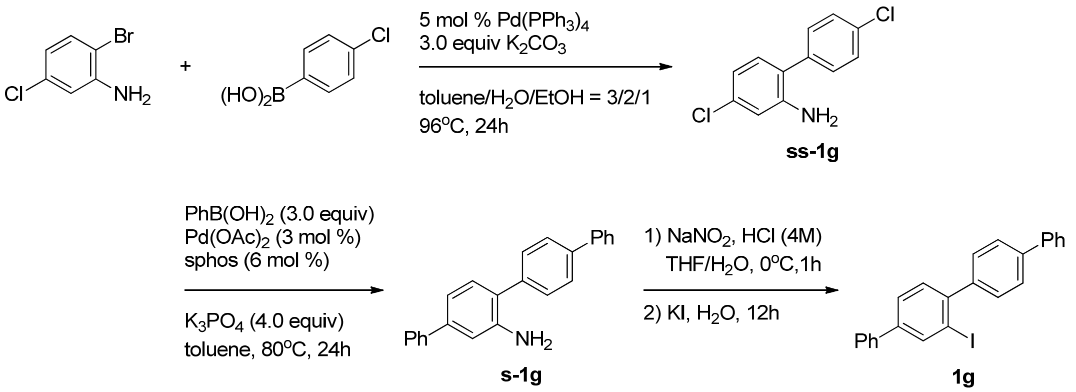

1g was prepared according to general procedure with one more step between 4,4'-dichloro-[1,1'-biphenyl]-2-amine (**ss-1g**) and [1,1':4',1'':4'',1'''-quaterphenyl]-2''-amine (**s-1g**). **ss-1g** was prepared according to general procedure from 2-bromo-5-chloroaniline (1.236 g, 6.0 mmol) and (4-chlorophenyl)boronic acid (936 mg, 6.0 mmol) as white solid (1.07 g, 75%). **s-1g** was prepared from **ss-1g** according to the reported procedure[4]. An oven-dried Schlenk tube containing a magnetic stir bar was charged with $Pd(OAc)_2$ (40.2 mg, 0.18 mmol, 3.0 mol%), dicyclohexyl(2',6'-dimethoxy-[1,1'-biphenyl]-2-yl)phosphine (sphos) (147 mg, 0.36 mmol, 6.0 mol%), the phenylboronic acid (2.92 g, 18 mmol, 3.0 equiv) and powdered, anhydrous K_3PO_4 (5.08 g, 24.0 mmol, 4.0 equiv). The Schlenk tube was

evacuated and backfilled with argon three times. Dry toluene (24.0 mL) was added under argon and the resulting mixture was stirred at room temperature for ~2 min before the **ss-1g** (1.428 g, 6.0 mmol, 1.0 equiv) was added. The reaction mixture was heated at 80 °C with vigorous stirring for 24h. After cooling to room temperature, the mixture was diluted with EtOAc, filtered through a thin pad of silica gel (eluting with EtOAc) and concentrated under reduced pressure. The **s-1g** was purified by flash chromatography on silica gel (1.40 g, 73%) as white soild. **1g** was obtained from **s-1g** (963 mg, 3.0 mmol, 1.0 equiv) according to general procedure as pale yellow solid (998 mg, 77%). **^{1}H NMR (400 MHz, CDCl$_3$)** δ 8.22 (d, *J* = 1.8 Hz, 1H), 7.70 – 7.67 (m, 4H), 7.65 – 7.60 (m, 3H), 7.52 – 7.43 (m, 6H), 7.43 – 7.33 (m, 3H). **^{13}C NMR (101 MHz, CDCl$_3$)** δ 144.93, 142.64, 141.93, 140.64, 140.46, 139.10, 138.18, 130.29, 129.76, 128.92, 128.80, 127.86, 127.42, 127.13, 127.10, 126.94, 126.68, 98.88. **ATR-FTIR (cm^{-1}):** 1632, 1455, 1397, 759, 689. **EI-MS (m/z, relative intensity):** 433 (M^{+}, 26), 432 (100), 305 (20), 304 (15), 303 (13), 302 (20), 289 (30), 226 (20), 216 (24), 152 (20), 151 (22).

4. General procedure for tetraphenylene synthesis

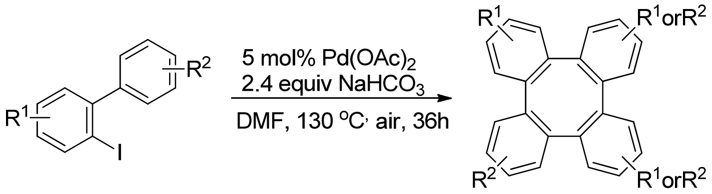

To a 10 mL Schlenk flask equipped with a magnetic stir bar was charged with Pd(OAc)$_2$ (2.3 mg, 0.01 mol, 5 mol%), *o*-iodobiaryls (0.40 mmol, 2.0 equiv), NaHCO$_3$ (40 mg, 0.48 mmol, 2.4 equiv) and 2 mL DMF. Then the mixture was stirred at 130 ^{o}C under air for 36 h. After cooling to room temperature, the solvent was removed under reduce pressure. The crude product was further purified by column chromatography on silica gel.

Tetraphenylene (2aa)

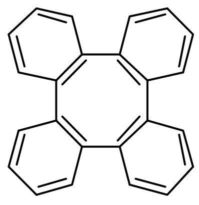

2aa

According to the general procedure, the reaction of **1a** (112 mg, 0.40 mmol, 2.0 equiv), $Pd(OAc)_2$ (2.3 mg, 0.01 mol, 5 mol%), $NaHCO_3$ (40 mg, 0.48 mmol, 2.4 equiv) and 2 mL DMF under air at 130 °C for 36 h after flash column chromatography on silica to afford 49.9 mg (82%) of **2aa** as white solid. **^{1}H NMR (400 MHz, $CDCl_3$)** δ 7.29 – 7.24 (m, 8H), 7.19 – 7.12 (m, 8H). **^{13}C NMR (101 MHz, $CDCl_3$)** δ 141.50, 129.01, 127.22. **ATR-FTIR (cm^{-1}):** 3059, 3015, 1632, 1463, 1424, 740, 577. **EI-MS (m/z, relative intensity):** 305 (M^+, 20), 304 (100), 303 (82), 302 (72), 300 (28), 150 (32).

2,7,10,15-Tetramethyltetraphenylene (2bb and 2cc)

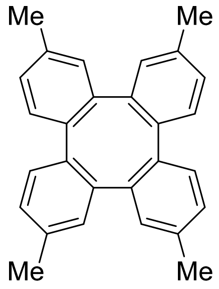

2bb/2cc

According to the general procedure, the reaction of **1b** (124 mg, 0.40 mmol, 2.0 equiv), $Pd(OAc)_2$ (2.3 mg, 0.01 mol, 5 mol%), $NaHCO_3$ (40 mg, 0.48 mmol, 2.4 equiv) and 2 mL DMF under air at 130 °C for 36 h after flash column chromatography on silica to afford 51.9 mg (72%) of **2bb** as white solid. According to the general procedure, the reaction of **1c** (124 mg, 0.40 mmol, 2.0 equiv), $Pd(OAc)_2$ (2.3 mg, 0.01 mol, 5 mol%), $NaHCO_3$ (40 mg, 0.48 mmol, 2.4 equiv) and 2 mL DMF under air at 130 °C for 36 h after flash column chromatography on silica to afford 57.6 mg (80%) of **2cc** as white solid. **^{1}H NMR (400 MHz, $CDCl_3$)** δ 7.11 – 7.02 (m, 8H), 6.98 (s, 4H), 2.33 (s, 12H). **^{13}C NMR (101 MHz, $CDCl_3$)** δ 141.68, 138.73, 136.51, 129.88, 129.07, 127.85, 21.05. **ATR-FTIR (cm^{-1}):** 3021, 2921, 2861, 1739, 1610, 1476, 1389, 1238, 817, 558. **HRMS m/z (ESI):** calcd for $C_{28}H_{24}H^+$ 361.1951, found 361.1957.

2,7,10,15-Tetraisopropyltetraphenylene (2dd)

According to the general procedure, the reaction of **1d** (146 mg, 0.40 mmol, 2.0 equiv), $Pd(OAc)_2$ (2.3 mg, 0.01 mol, 5 mol%), $NaHCO_3$ (40 mg, 0.48 mmol, 2.4 equiv) and 2 mL DMF under air at 130 °C for 36 h after flash column chromatography on

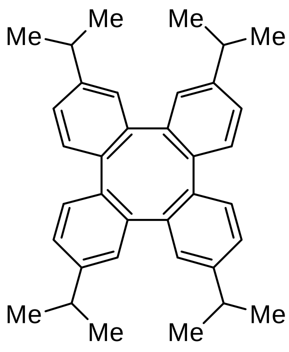

2dd

silica to afford 57.8 mg (61%) of **2dd** as white solid. **^{1}H NMR (400 MHz, CDCl$_3$)** δ 7.15 (dd, *J* = 7.9, 1.8 Hz, 4H), 7.08 (d, *J* = 7.9 Hz, 4H), 7.03 (d, *J* = 1.8 Hz, 4H), 2.91 (hept, *J* = 6.9 Hz, 4H), 1.27 (dd, *J* = 6.9, 1.5 Hz, 24H). **^{13}C NMR (101 MHz, CDCl$_3$)** δ 147.25, 141.92, 139.15, 129.49, 127.67, 124.98, 33.57, 23.99, 23.84. **ATR-FTIR (cm^{-1}):** 2960, 2872, 1634, 1471, 1395, 1049, 825. **HRMS m/z (ESI):** calcd for $C_{36}H_{40}Na^+$ 495.3022, found 495.3022.

2,7,10,15-Tetramethoxytetraphenylene (2ee)

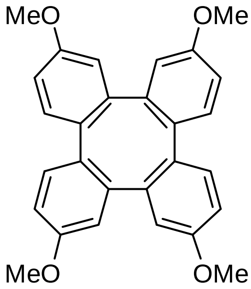

2ee

According to the general procedure, the reaction of **1e** (136 mg, 0.40 mmol, 2.0 equiv), Pd(OAc)$_2$ (2.3 mg, 0.01 mol, 5 mol%), NaHCO$_3$ (40 mg, 0.48 mmol, 2.4 equiv) and 2 mL DMF under air at 130 °C for 36 h after flash column chromatography on silica to afford 57.7 mg (68%) of **2ee** as white solid. **^{1}H NMR (400 MHz, CDCl$_3$)** δ 7.06 (d, *J* = 8.4 Hz, 4H), 6.82 (dd, *J* = 8.4, 2.7 Hz, 4H), 6.72 (d, *J* = 2.7 Hz, 4H), 3.77 (s, 12H). **^{13}C NMR (101 MHz, CDCl$_3$)** δ 158.39, 142.94, 133.76, 130.39, 114.16, 112.86, 55.19. **ATR-FTIR (cm^{-1}):** 2923, 2839, 1602, 1469, 1416, 1226, 1032, 807. **HRMS m/z (ESI):** calcd for $C_{28}H_{24}O_4Na^+$ 447.1567, found 447.1563.

***N^2,N^2,N^7,N^7,N^{10},N^{10},N^{15},N^{15}*-octamethyltetraphenylene-2,7,10,15-tetraamine (2ff)**

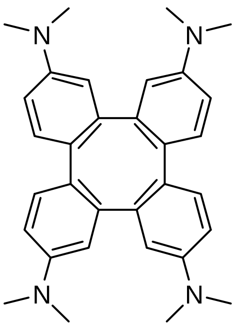

2ff

According to the general procedure, the reaction of **1f** (146 mg, 0.40 mmol, 2.0 equiv), Pd(OAc)$_2$ (2.3 mg, 0.01 mol, 5 mol%), NaHCO$_3$ (40 mg, 0.48 mmol, 2.4 equiv) and 2 mL DMF under air at 130 °C for 36 h after flash column chromatography on silica to afford 62.0 mg (65%) of **2ff** as brown solid. **^{1}H NMR (400 MHz, CDCl$_3$)** δ 7.03 (d, *J* = 8.4 Hz, 4H), 6.65 (dd, *J* = 8.4, 2.7 Hz, 4H), 6.58 (d, *J* = 2.7 Hz, 4H), 2.91 (s, 24H). **^{13}C NMR (101 MHz, CDCl$_3$)** δ 149.07, 143.64, 130.74, 130.06, 113.58, 111.31, 40.66. **ATR-FTIR (cm^{-1}):**

1630, 1398, 1118, 799. **HRMS m/z (ESI):** calcd for $C_{32}H_{36}N_4Na^+$ 499.2832, found 499.2831.

2,7,10,15-Tetraphenyltetraphenylene (2gg)

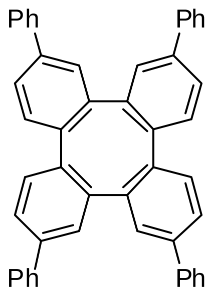

2gg

According to the general procedure, the reaction of **1g** (173 mg, 0.40 mmol, 2.0 equiv), $Pd(OAc)_2$ (2.3 mg, 0.01 mol, 5 mol%), $NaHCO_3$ (40 mg, 0.48 mmol, 2.4 equiv) and 2 mL DMF under air at 130 °C for 36 h after flash column chromatography on silica to afford 48.7 mg (40%) of **2gg** as pale yellow solid. **^{1}H NMR (400 MHz, $CDCl_3$)** δ 7.94 (d, *J* = 1.3 Hz, 4H), 7.75 (dd, *J* = 7.7, 1.7 Hz, 4H), 7.66 – 7.60 (m, 12H), 7.47 (t, *J* = 7.5 Hz, 8H), 7.40 (d, *J* = 7.4 Hz, 4H). **^{13}C NMR (101 MHz, $CDCl_3$)** δ 143.06, 142.23, 139.81, 135.21, 133.32, 128.95, 127.92, 126.78, 123.05, 120.76. **ATR-FTIR (cm^{-1}):** 1629, 1472, 1397, 1117, 761, 692.

2,7,10,15-Tetranitrotetraphenylene (2hh)

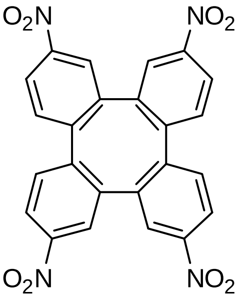

2hh

According to the general procedure, the reaction of **1h** (148 mg, 0.40 mmol, 2.0 equiv), $Pd(OAc)_2$ (2.3 mg, 0.01 mol, 5 mol%), $NaHCO_3$ (40 mg, 0.48 mmol, 2.4 equiv) and 2 mL DMF under air at 130 °C for 36 h after flash column chromatography on silica to afford 24.2 mg (25%) of **2hh** as white solid. **^{1}H NMR (400 MHz, $CDCl_3$)** δ 8.31 (dd, *J* = 8.4, 2.3 Hz, 4H), 8.15 (d, *J* = 2.3 Hz, 4H), 7.47 (d, *J* = 8.4 Hz, 4H). **^{13}C NMR (101 MHz, $CDCl_3$)** δ 148.13, 144.81, 139.70, 130.45, 124.32, 124.07. **ATR-FTIR (cm^{-1}):** 1632, 1516, 1398, 1341, 1116.

2,3,6,7,10,11,14,15-Octamethyltetraphenylene (2ii)

According to the general procedure, the reaction of **1i** (134 mg, 0.40 mmol, 2.0 equiv), $Pd(OAc)_2$ (2.3 mg, 0.01 mol, 5 mol%), $NaHCO_3$ (40 mg, 0.48 mmol, 2.4 equiv) and 2 mL DMF under air at 130 °C for 36 h after flash column chromatography on

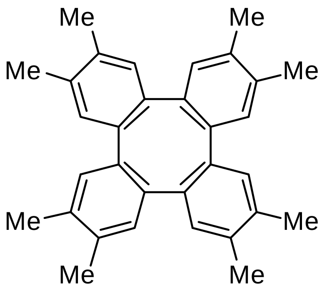

2i

silica to afford 65.8 mg (79%) of **2ii** as white solid. **^{1}H NMR (400 MHz, CDCl$_3$)** δ 6.93 (s, 8H), 2.23 (s, 24H). **^{13}C NMR (101 MHz, CDCl$_3$)** δ 139.28, 135.02, 130.69, 19.33. **ATR-FTIR (cm^{-1}):** 3011, 2923, 2861, 1630, 1445, 1396, 1114, 1027, 804. **HRMS m/z (ESI):** calcd for $C_{32}H_{32}Na^{+}$ 439.2396, found 439.2392.

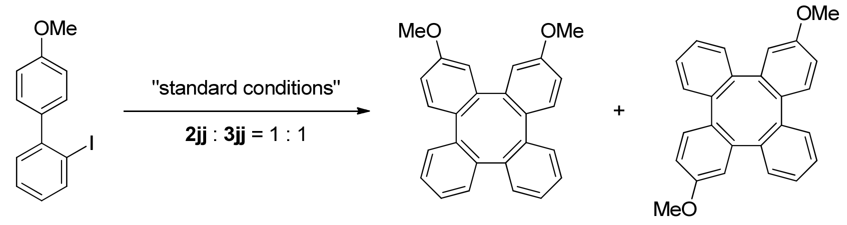

1j **2jj** **3jj**

2,15-Dimethoxytetraphenylene (2jj) and 2,10-dimethoxytetraphenylene (3jj)

According to the general procedure, the reaction of **1j** (124 mg, 0.40 mmol, 2.0 equiv), Pd(OAc)$_2$ (2.3 mg, 0.01 mol, 5 mol%), NaHCO$_3$ (40 mg, 0.48 mmol, 2.4 equiv) and 2 mL DMF under air at 130 ^{o}C for 36 h after flash column chromatography on silica to afford 48.1 mg (66%) of **2jj** and **3jj** as white solid. The ratio of **2jj** and **3jj** was determined by crude ^{1}H NMR. **2jj** and **3jj** can be separated by oft-repeated column chromatography on silica gel.

2,15-Dimethoxytetraphenylene (2jj)

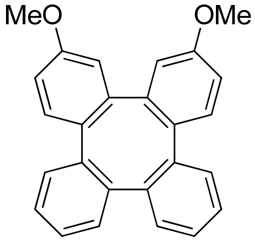

2jj

^{1}H NMR (400 MHz, CDCl$_3$) δ 7.28 – 7.25 (m, 2H), 7.16 – 7.12 (m, 2H), 7.07 (d, *J* = 8.4 Hz, 1H), 6.82 (dd, *J* = 8.4, 2.7 Hz, 1H), 6.72 (d, *J* = 2.7 Hz, 1H), 3.78 (s, 3H). **^{13}C NMR (101 MHz, CDCl$_3$)** δ 158.54, 142.59, 141.93, 141.19, 134.15, 130.19, 129.27, 129.03, 127.13, 127.00, 114.19, 112.97, 55.23. **ATR-FTIR (cm^{-1}):** 3057, 2927, 2840, 1606, 1468, 1287, 1217, 1035, 819, 760. **EI-MS (m/z, relative intensity):** 365 (M^{+}, 26), 364 (100), 289 (15), 276 (15), 138 (28).

2,10-Dimethoxytetraphenylene (3jj)

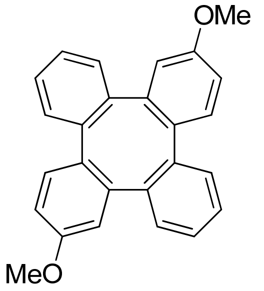

3jj

^{1}H NMR (400 MHz, $CDCl_3$) δ 7.28 – 7.24 (m, 2H), 7.18 – 7.12 (m, 2H), 7.08 (d, *J* = 8.4 Hz, 1H), 6.83 (dd, *J* = 8.4, 2.7 Hz, 1H), 6.70 (d, *J* = 2.7 Hz, 1H), 3.78 (s, 3H). **^{13}C NMR (101 MHz, $CDCl_3$)** δ 158.56, 142.80, 141.71, 141.15, 134.17, 130.14, 129.30, 128.92, 127.26, 126.97, 114.24, 112.87, 55.21. **ATR-FTIR (cm^{-1}):** 3057, 2927, 2840, 1606, 1468, 1287, 1217, 1035, 819, 760. **EI-MS (m/z, relative intensity):** 365 (M^+, 26), 364 (100), 289 (15), 276 (15), 138 (28).

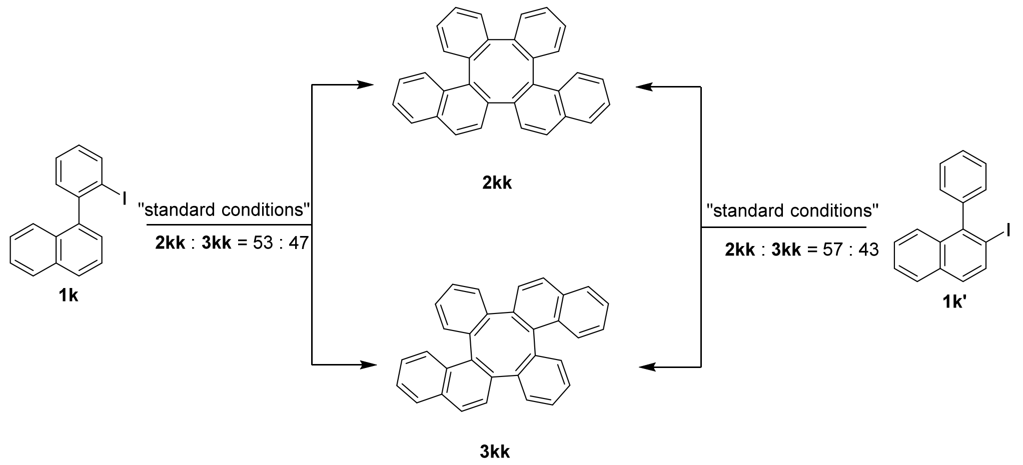

According to the general procedure, the reaction of **1k** (132 mg, 0.40 mmol, 2.0 equiv), $Pd(OAc)_2$ (2.3 mg, 0.01 mol, 5 mol%), $NaHCO_3$ (40 mg, 0.48 mmol, 2.4 equiv) and 2 mL DMF under air at 130 °C for 36 h after flash column chromatography on silica to afford 15.6 mg (19%) of **2kk** and 14.2 mg (18%) of **3kk** as white solid. The ratio of **2kk** and **3kk** was determined by crude ^{1}H NMR. **2kk** and **3kk** can be separated by column chromatography on silica gel.

According to the general procedure, the reaction of **1k'** (132 mg, 0.40 mmol, 2.0 equiv), $Pd(OAc)_2$ (2.3 mg, 0.01 mol, 5 mol%), $NaHCO_3$ (40 mg, 0.48 mmol, 2.4 equiv) and 2 mL DMF under air at 130 °C for 36 h after flash column chromatography on silica to afford 14.3 mg (18%) of **2kk** and 11.3 mg (14%) of **3kk** as white solid. The **ratio** of **2kk** and **3kk** was determined by crude ^{1}H NMR. **2kk** and **3kk** can be separated by column chromatography on silica gel.

Dibenzo[*a,i*]tetraphenylene (2kk)

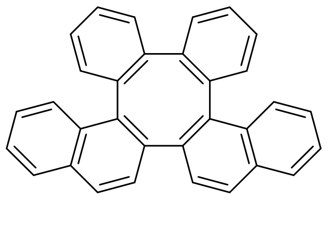

2kk

^{1}H NMR (400 MHz, $CDCl_3$) δ7.84 – 7.77 (m, 4H), 7.50 – 7.31 (m, 10H), 7.25 – 7.19 (m, 4H), 7.16 – 7.12 (m, 2H). **^{13}C NMR (101 MHz, $CDCl_3$)** δ 142.61, 139.29, 138.56, 137.60, 132.76, 132.19, 130.18, 128.31, 127.80, 127.43, 127.28, 126.83, 126.67, 126.43, 126.07, 125.44. **ATR-FTIR (cm^{-1}):** 3056, 1630, 1470, 1393, 1239, 1118, 749. **HRMS m/z (ESI):** calcd for $C_{32}H_{20}Na^+$ 427.1457, found 427.1463. **EI-MS (m/z, relative intensity):** 405 (M^+, 31), 404 (100), 403 (23), 402 (18), 401 (18), 400 (18), 200 (19).

Dibenzo[*a,m*]tetraphenylene (3kk)

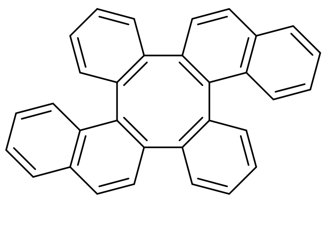

3kk

^{1}H NMR (400 MHz, $CDCl_3$) δ 7.75 – 7.71 (m, 2H), 7.67 (d, *J* = 8.4 Hz, 2H), 7.48 (d, *J* = 8.3 Hz, 2H), 7.41 – 7.26 (m, 12H), 7.20 – 7.14 (m, 2H). **^{13}C NMR (101 MHz, $CDCl_3$)** δ 143.04, 138.95, 138.77, 137.41, 132.74, 131.95, 130.49, 128.80, 127.75, 127.35, 127.30, 126.54, 126.52, 126.49, 126.00, 125.33. **ATR-FTIR (cm^{-1}):** 3056, 1638, 1431, 1181, 1244, 1044, 819, 756. **EI-MS (m/z, relative intensity):** 405 (M^+, 34), 404 (100), 403 (24), 402 (18), 401 (18), 400 (18), 200 (20).

5. Cross-coupling and cyclization of two different *o*-iodobiaryls

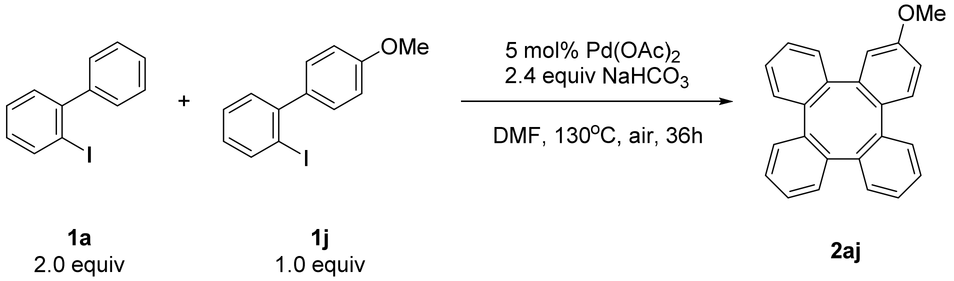

To a 10 mL Schlenk flask equipped with a magnetic stir bar was charged with $Pd(OAc)_2$ (2.3 mg, 0.01 mol, 5 mol%), **1j** (62.0 mg, 0.20 mol, 1.0 equiv), **1a** (112 mg, 0.40 mol, 2.0 equiv), $NaHCO_3$ (40 mg, 0.48 mmol, 2.4 equiv) and 2 mL DMF. Then

the mixture was stirred at 130 °C under air for 36 h. After cooling to room temperature, the solvent was removed under reduce pressure. The crude product after column chromatography on silica afford 30.6 mg (46%) **2aj** as white solid. **^{1}H NMR (400 MHz, $CDCl_3$)** δ 7.30 – 7.23 (m, 7H), 7.20 – 7.10 (m, 6H), 7.07 (d, *J* = 8.4 Hz, 1H), 6.82 (dd, *J* = 8.4, 2.6 Hz, 1H), 6.70 (d, *J* = 2.6 Hz, 1H), 3.77 (s, 3H). **^{13}C NMR (101 MHz, $CDCl_3$)** δ 158.57, 142.64, 141.76, 141.69, 141.49, 141.47, 141.22, 134.22, 130.11, 129.23, 129.09, 129.06, 129.02, 128.91, 127.29, 127.24, 127.21, 127.19, 127.15, 126.98, 114.26, 112.92, 55.20. **ATR-FTIR (cm^{-1}):** 3061, 2924, 2847, 1615, 1399, 1117, 749. **EI-MS (m/z, relative intensity):** 335 (M^+, 26), 334 (100), 289 (32), 151 (30), 144 (30).

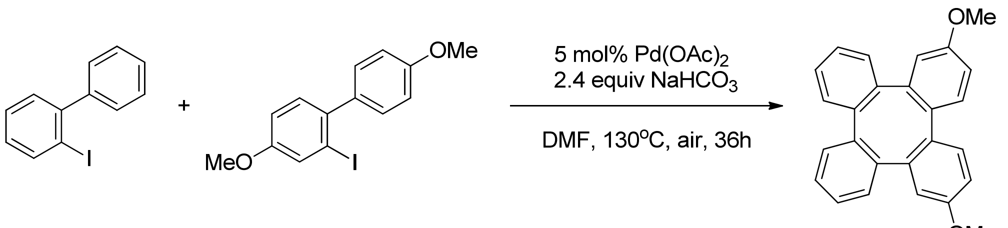

1a 2.0 equiv　**1e** 1.0 equiv　**2ae**

To a 10 mL Schlenk flask equipped with a magnetic stir bar was charged with $Pd(OAc)_2$ (2.3 mg, 0.01 mol, 5 mol%), **1e** (68.0 mg, 0.20 mol, 1.0 equiv), **1a** (112 mg, 0.40 mol, 2.0 equiv), $NaHCO_3$ (40 mg, 0.48 mmol, 2.4 equiv) and 2 mL DMF. Then the mixture was stirred at 130 °C under air for 36 h. After cooling to room temperature, the solvent was removed under reduce pressure. The crude product after column chromatography on silica afford 40.1 mg (55%) **2ae** as white solid. **^{1}H NMR (400 MHz, $CDCl_3$)** δ 7.30 – 7.25 (m, 4H), 7.19 – 7.14 (m, 4H), 7.04 (d, *J* = 8.4 Hz, 2H), 6.79 (dd, *J* = 8.4, 2.7 Hz, 2H), 6.69 (d, *J* = 2.7 Hz, 2H), 3.75 (s, 3H). **^{13}C NMR (101 MHz, $CDCl_3$)** δ 158.37, 142.81, 141.63, 141.42, 133.86, 130.27, 129.13, 128.90, 127.23, 114.24, 112.87, 55.18. **ATR-FTIR (cm^{-1}):** 3057, 2931, 2837, 1737, 1602, 1467, 1213, 757. **HRMS m/z (ESI):** calcd for $C_{26}H_{20}O_2Na^+$ 387.1356, found 387.1353.

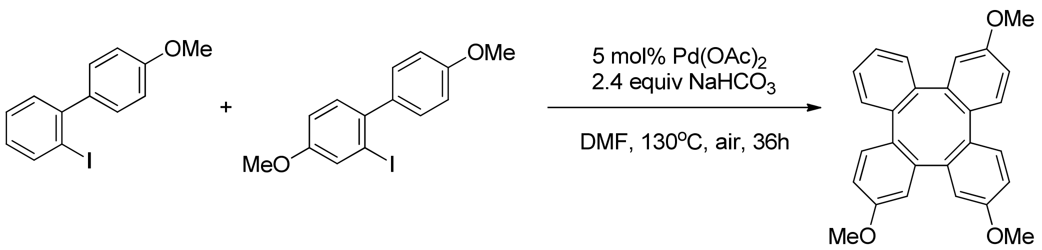

1j **1e** **2ej**

Method A: To a 10 mL Schlenk flask equipped with a magnetic stir bar was charged with $Pd(OAc)_2$ (2.3 mg, 0.01 mol, 5 mol%), **1e** (68.0 mg, 0.20 mol, 1.0 equiv), **1j** (124 mg, 0.40 mol, 2.0 equiv), $NaHCO_3$ (40 mg, 0.48 mmol, 2.4 equiv) and 2 mL DMF. Then the mixture was stirred at 130 ^oC under air for 36 h. After cooling to room temperature, the solvent was removed under reduce pressure. The crude product after column chromatography on silica afford 36.6 mg (46%) **2ej** as white solid. **Method B**: To a 10 mL Schlenk flask equipped with a magnetic stir bar was charged with $Pd(OAc)_2$ (2.3 mg, 0.01 mol, 5 mol%), **1j** (62.0 mg, 0.20 mol, 1.0 equiv), **1e** (136 mg, 0.40 mol, 2.0 equiv), $NaHCO_3$ (40 mg, 0.48 mmol, 2.4 equiv) and 2 mL DMF. Then the mixture was stirred at 130 ^oC under air for 36 h. After cooling to room temperature, the solvent was removed under reduce pressure. The crude product after column chromatography on silica afford 38.7 mg (49%) **2ej** as white solid. **^{1}H NMR (400 MHz, $CDCl_3$)** δ 7.29 – 7.24 (m, 2H), 7.18 – 7.12 (m, 2H), 7.09 – 7.03 (m, 3H), 6.85 – 6.78 (m, 3H), 6.74 – 6.66 (m, 3H), 3.82 – 3.74 (m, 9H). **^{13}C NMR (101 MHz, $CDCl_3$)** δ 158.56, 158.40, 158.37, 142.99, 142.77, 142.75, 141.88, 141.13, 134.10, 133.83, 133.79, 130.34, 130.29, 130.21, 129.34, 128.90, 127.19, 126.98, 114.25, 114.19, 114.16, 112.90, 112.82, 55.18. **ATR-FTIR (cm^{-1}):** 3058, 2926, 2841, 1604, 1470, 1409, 1216, 1034, 813, 733. **HRMS m/z (ESI):** calcd for $C_{27}H_{27}O_3Na^+$ 417.1461, found 417.1455.

6. Gram-scale transformation and diversification of 2ee

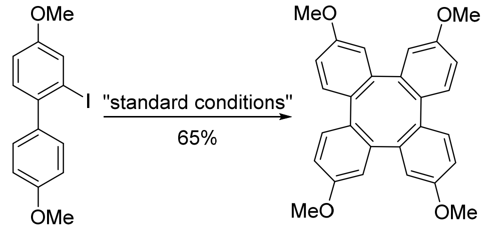

To a 200 mL flask equipped with a magnetic stir bar was charged with **1e** (3.5 g, 10.3 mol, 2.0 equiv), $Pd(OAc)_2$ (115 mg, ,0.51 mol, 5 mol%) and $NaHCO_3$ (1.04 mg, 10.3 mmol, 2.4 equiv) and 50 mL DMF. Then the mixture was stirred at 130 ^{o}C under air for 36 h. After cooling to room temperature, the solvent was removed under reduce pressure. The crude product after column chromatography on silica afford 1.42g (65%) **2ee** as white solid.

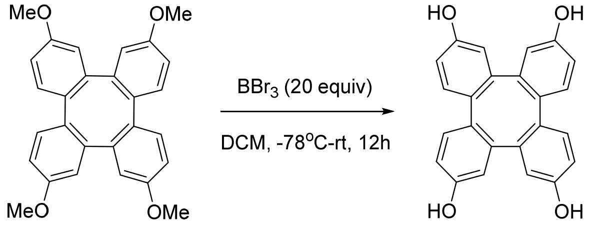

To a solution of compound **2ee** (848 mg, 2.0 mmol, 1.0 equiv) in dry CH_2Cl_2 (5.0 mL) under Ar at -78 °C was added dropwise a solution of BBr_3 (40 mL, 40 mmol, 1M in CH_2Cl_2). The reaction solution was stirred at this temperature for 1 h before allowing to warm to room temperature and stirred for 12 h. The reaction mixture was quenched with methanol, the solvent was removed in vacuo and the residue was extracted with CH_2Cl_2 and washed with a solution of $NaHCO_3$. The organic layer was washed with brine, dried over anhydrous Na_2SO_4 and concentrated in vacuo. The residue was purified by column chromatography (PE/EtOAc 1:1) to give 604 mg (82%) of tetraphenylene-2,7,10,15-tetraol (**3ee**) as white solid. **^{1}H NMR (400 MHz, d$_6$-Acetone)** δ 8.31 (s, 4H), 6.93 (d, *J* = 8.3 Hz, 4H), 6.74 (dd, *J* = 8.3, 2.6 Hz, 4H), 6.62 (d, *J* = 2.5 Hz, 4H). **^{13}C NMR (101 MHz, d$_6$-Acetone)** δ 157.08, 144.20, 133.90,

131.24, 116.30, 114.99. **ATR-FTIR (cm^{-1}):** 3418, 2948, 2839, 1648, 1443, 1023. **HRMS m/z (ESI):** calcd for $C_{24}H_{16}O_4Na^+$ 391.0941, found 391.0946.

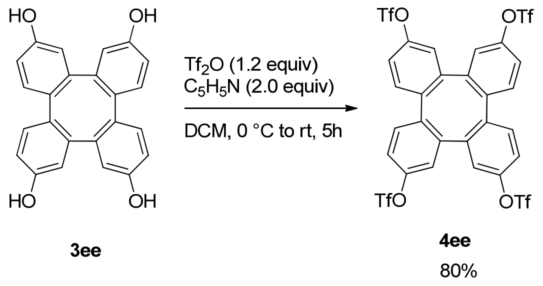

To a stirring solution of **3ee** (368 mg, 1.0 mmol, 1.0 equiv) in DCM (5.0 mL) under Ar was added pyridine (632 mg, 8.0 mmol, 8.0 equiv). The mixture was cooled in an ice-water bath, and triflic anhydride (1.35 g, 4.8 mmol, 4.8 equiv) was added dropwise. The mixture was allowed to warm to rt and stir for five hours. Then the reaction was quenched by the addition of water. The layers were separated, and the aqueous phase was extracted three times with DCM. The combined organic extracts were washed with 1 M HCl, saturated aqueous sodium bicarbonate, and brine, then dried over Na_2SO_4, filtered, and evaporated under reduced pressure. The product tetraphenylene-2,7,10,15-tetrayl tetrakis(trifluoromethanesulfonate) (**4ee**) was purified on silica gel (PE/EtOAc 20:1) as white solid (717 mg, 80%). **^{1}H NMR (400 MHz, $CDCl_3$)** δ 7.35 – 7.27 (m, 8H), 7.11 (d, J = 2.3 Hz, 4H). **^{13}C NMR (101 MHz, $CDCl_3$)** δ 149.11, 141.12, 139.25, 131.24, 121.93, 121.63, 118.68 (q, J = 320.8 Hz). **^{19}F NMR (377 MHz, $CDCl_3$)** δ -72.78. **ATR-FTIR (cm^{-1}):** 1576, 1473, 1423, 1213, 1138, 943, 886, 821, 603. **HRMS m/z (ESI):** calcd for $C_{28}H_{12}F_{12}O_{12}S_4Na^+$ 918.8912, found 918.8921.

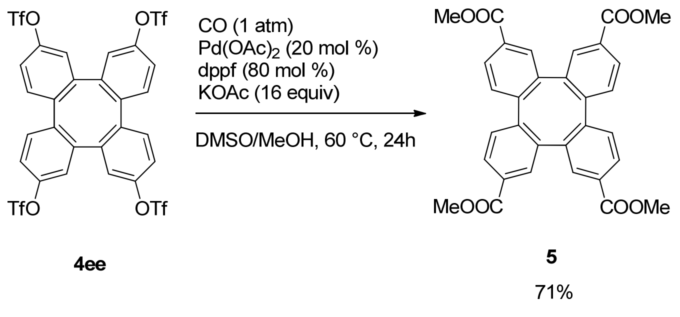

To a solution of **4ee** (179 mg, 0.20 mol, 1.0 equiv) in DMSO/MeOH (5/2.4 mL) was added $Pd(OAc)_2$ (8.9 mg, 0.04 mmol, 20 mol%), 1,1'-bis(diphenylphosphino)ferrocene (dppf) (88.6 mg, 0.16 mmol, 80 mol%), and potassium acetate (314 mg, 3.2 mmol, 16.0 equiv). CO was bubbled through the mixture for 5 minutes, and then the reaction mixture was placed in an oil bath with stirring at 60 °C under a balloon of CO overnight. The mixture was then cooled to rt, quenched with 1 M HCl (pH < 3), and extracted three times with EtOAc. The combined organic extracts were washed with brine, dried over Na_2SO_4, filtered and evaporated under reduced pressure. The tetramethyl tetraphenylene-2,7,10,15-tetracarboxylate (**5**) was purified on silica gel (PE:EtOAc = 80:40) as white solid (76.1 mg, 71%). **^{1}H NMR (400 MHz, $CDCl_3$)** δ 7.99 (dd, J = 8.0, 1.7 Hz, 4H), 7.87 (d, *J* = 1.6 Hz, 4H), 7.26 (d, *J* = 8.0 Hz, 4H), 3.89 (s, 12H). **^{13}C NMR (101 MHz, $CDCl_3$)** δ 166.44, 144.74, 140.21, 130.41, 129.92, 129.13, 129.09, 52.23. **ATR-FTIR (cm^{-1}):** 2951, 1721, 1600, 1439, 1249, 1112, 767. **HRMS m/z (ESI):** calcd for $C_{32}H_{24}O_8Na^+$ 559.1363, found 559.1359.

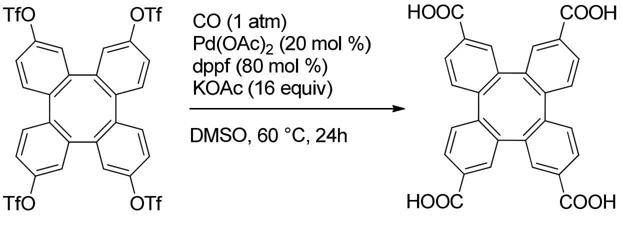

4ee

6

85%

To a solution of **4ee** (179 mg, 0.20 mol, 1.0 equiv) in DMSO (2.0 mL) was added $Pd(OAc)_2$ (8.9 mg, 0.04 mmol, 20 mol%), 1,1'-bis(diphenylphosphino)ferrocene (dppf) (88.6 mg, 0.16 mmol, 80 mol%), and potassium acetate (314 mg, 3.2 mmol, 16.0 equiv). CO was bubbled through the mixture for 5 minutes, and then the reaction mixture was placed in an oil bath with stirring at 60 °C under a balloon of CO overnight. The mixture was then cooled to rt, quenched with 1 M HCl (pH < 3), and extracted three times with EtOAc. The combined organic extracts were washed with brine, dried over Na_2SO_4, filtered and

evaporated under reduced pressure. The tetraphenylene-2,7,10,15-tetracarboxylic acid (**6**) was purified on silica gel (PE:EtOAc:AcOH = 80:40:1) as white solid (81.6 mg, 85%). **^{1}H NMR (400 MHz, CD$_3$OD)** δ 8.02 (dd, *J* = 8.0, 1.7 Hz, 4H), 7.82 (d, *J* = 1.6 Hz, 4H), 7.35 (d, *J* = 8.0 Hz, 4H). **^{13}C NMR (101 MHz, CD$_3$OD)** δ 169.15, 146.20, 141.77, 132.04, 131.25, 130.38, 130.31. **ATR-FTIR (cm^{-1}):** 3445, 1704, 1637, 1389, 1218, 1123, 776. **HRMS m/z (ESI):** calcd for $C_{28}H_{16}O_8Na^+$ 503.0737, found 503.0748.

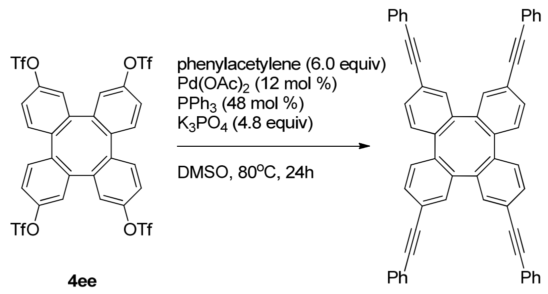

4ee

7
78%

To a solution of **4ee** (179 mg, 0.20 mol, 1.0 equiv) in DMSO (2.0 mL) was added ethynylbenzene (122.4 mg, 1.2 mmol, 6.0 equiv), Pd(OAc)$_2$ (5.4 mg, 0.024 mmol, 12 mol%), PPh$_3$ (25.2 mg, 0.096 mmol, 48 mol%), and K_3PO_4 (314 mg, 0.96 mmol, 4.8 equiv) under Ar. After stirring at 80 °C for 24 h, water was added and the resulting mixture was extracted with diethyl ether. The combined organic layer was washed with brine, and dried over $MgSO_4$. Evaporation of the solvent followed by purification with column chromatography (PE:EtOAc = 50:1) gave the 2,7,10,15-tetrakis(phenylethynyl)tetraphenylene (**7**) as white solid (109.8 mg, 78%). **^{1}H NMR (400 MHz, CDCl$_3$)** δ 7.52 – 7.47 (m, 12H), 7.41 (d, *J* = 1.5 Hz, 4H), 7.36 – 7.31 (m, 12H), 7.18 (d, *J* = 7.9 Hz, 4H). **^{13}C NMR (101 MHz, CDCl$_3$)** δ 140.75, 140.67, 132.35, 131.60, 130.80, 129.16, 128.33, 123.09, 122.74, 90.20, 88.82. **ATR-FTIR (cm^{-1}):** 1630, 1396, 1258, 1112, 800, 753.

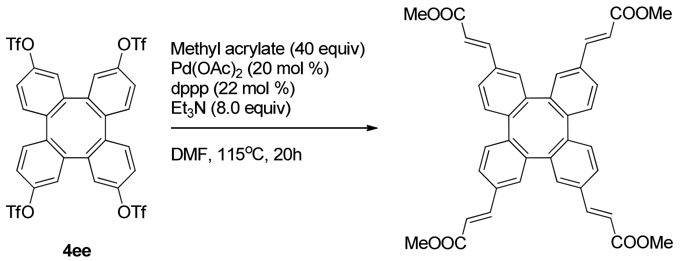

8
98%

To a stirred solution of **4ee** (179 mg, 0.20 mol, 1.0 equiv) in DMF (3.6 mL) under Ar at rt were sequentially added Et_3N (162 mg, 1.6 mmol, 8.0 equiv), methyl acrylate (688 mg, 8.0 mmol, 40 equiv), 1,3-bis(diphenylphosphino)propane (dppp) (18 mg, 0.044 mmol, 22 mol%) and $Pd(OAc)_2$ (8.9 mg, 0.04 mmol, 20 mol%). The reaction was stirred and heated at 115 °C for 5 h and then cooled to rt, diluted with CH_2Cl_2, and sequentially washed with 5% HCl and water until neutrality was achieved. The organic phase was dried over Na_2SO_4, and the solvent was removed under reduced pressure. The (2*E*,2'*E*,2''*E*,2'''*E*)-tetramethyl 3,3',3'',3'''-(tetraphenylene-2,7,10,15-tetrayl)tetraacrylate (**8**) was purified by flash chromatography (PE:EtOAc = 3:1) as white solid (125.4 mg, 98%). **^{1}H NMR (400 MHz, $CDCl_3$)** δ 7.65 (d, *J* = 16.0 Hz, 4H), 7.48 (dd, *J* = 8.0, 1.5 Hz, 4H), 7.32 (d, *J* = 1.3 Hz, 4H), 7.21 (d, *J* = 7.9 Hz, 4H), 6.42 (d, *J* = 16.0 Hz, 4H), 3.78 (s, 12H). **^{13}C NMR (101 MHz, $CDCl_3$)** δ 167.14, 143.77, 142.44, 141.09, 134.11, 129.71, 128.83, 127.48, 118.54, 51.73. **ATR-FTIR (cm^{-1}):** 2923, 2852, 1708, 1635, 1426, 1171.

7. Mechanism insights

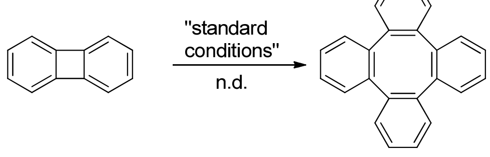

9 2aa

To a 10 mL Schlenk flask equipped with a magnetic stir bar was charged with $Pd(OAc)_2$ (2.3 mg, 0.01 mol, 5 mol%), biphenylene (**9**) (60.8 mg, 0.40 mol, 2.0 equiv), $NaHCO_3$ (40 mg, 0.48 mmol, 2.4 equiv) and 2 mL DMF. Then the mixture was stirred

at 130 °C under air for 36 h. However, **2aa** can not be detected by GC-MS and crude ^{1}H NMR.

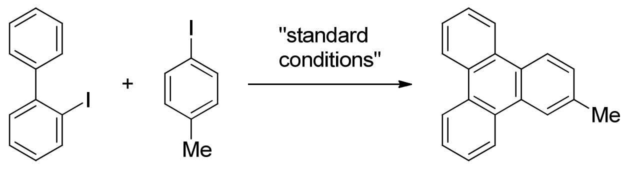

1a **10** **11**

To a 10 mL Schlenk flask equipped with a magnetic stir bar was charged with $Pd(OAc)_2$ (2.3 mg, 0.01 mol, 5 mol%), **1a** (56.0 mg, 0.20 mol, 1.0 equiv), **10** (43.6 mg, 0.20 mol, 1.0 equiv), $NaHCO_3$ (40 mg, 0.48 mmol, 2.4 equiv) and 2 mL DMF. Then the mixture was stirred at 130 °C under air for 36 h. After cooling to room temperature, the solvent was removed under reduce pressure. The crude product after column chromatography on silica afford 6.2 mg (13%) **11** as white solid. **^{1}H NMR (400 MHz, CDCl$_3$)** δ 8.70 – 8.60 (m, 4H), 8.54 (d, J = 8.3 Hz, 1H), 8.45 (s, 1H), 7.71 – 7.58 (m, 4H), 7.49 (dd, J = 8.4, 1.7 Hz, 1H), 2.62 (s, 3H). **^{13}C NMR (101 MHz, CDCl$_3$)** δ 136.85, 129.86, 129.72, 129.67, 129.37, 128.66, 127.44, 127.13, 127.06, 127.04, 126.74, 123.28, 123.26, 123.22, 123.05, 21.84. **EI-MS (m/z, relative intensity):** 243 (M^+, 21), 242 (100), 241 (48), 239 (36), 120 (23).

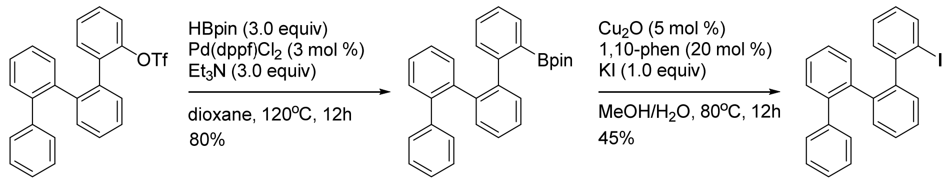

ss-12 **s-12** **12**

[1,1':2',1'':2'',1'''-Quaterphenyl]-2-yl trifluoromethanesulfonate (**ss-12**) was prepared from phenol according to the literature.[5]

To a solution of Pd(dppf)Cl_2 (66 mg, 0.09 mmol, 3 mol%) in dioxane (2.4 mL) was sequentially added [1,1':2',1'':2'',1'''-quaterphenyl]-2-yl trifluoromethanesulfonate (**ss-12**) (1.36 g, 3.0 mol, 1.0 equiv), Et_3N (909 mg, 9.0 mmol, 3.0 equiv), and pinacolborane (1.152 g, 9.0 mmol, 3.0 equiv). After being stirred for 12 h at 120 °C, the reaction mixture was quenched with methanol dropwise. The solvent was removed

in vacuo and the residue was extracted with CH_2Cl_2 and washed with water, dried over $MgSO_4$, and concentrated. The residue was purified by flash column chromatography on silica gel (PE/EtOAc 50:1) to give 1.04 g (80% yield) of 2-([1,1':2',1'':2'',1'''-quaterphenyl]-2-yl)-4,4,5,5-tetramethyl-1,3,2-dioxaborolane (**s-12**) as pale yellow oil.

To a 250 mL pressure vessel containing the **s-12** (650 mg, 1.5 mmol, 1.0 equiv), Cu_2O (10.7 mg, 0.075 mmol, 5.0 mol%), 1,10-phen (54.0 mg, 0.30 mmol, 20.0 mol%) and KI (250 mg, 1.5 mmol, 1.00 equiv) was added MeOH (10 mL). The mixture was stirred at room temperature, and water (2.5 mL) was added. The flask was sealed under air, and the mixture was heated at 80 °C for 12h. The reaction was cooled to room temperature, water was added, and the mixture was extracted with EtOA. The combined organic phases were washed with brine, dried over $MgSO_4$ and concentrated in vacuo. The residue was purified by flash column chromatography on silica gel (only PE) to give 292 mg (45% yield) of 2-iodo-1,1':2',1'':2'',1'''-quaterphenyl (**12**) as white solid.

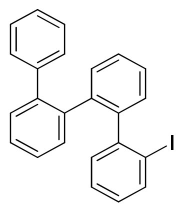

12

^{1}H NMR (400 MHz, CDCl$_3$) δ 7.72 (d, *J* = 7.5 Hz, 1H), 7.42 – 6.91 (m, 14H), 6.86 – 6.76 (m, 1H), 6.31 (d, *J* = 6.2 Hz, 1H). **^{13}C NMR (101 MHz, CDCl$_3$)** δ 145.92, 143.00, 141.35, 140.32, 138.91, 138.48, 132.34, 131.93, 130.62, 130.41, 129.88, 128.11, 127.77, 127.45, 127.30, 126.76, 126.48, 100.45. **ATR-FTIR (cm^{-1}):** 3056, 3018, 1443, 1421, 1003, 749, 697. **HRMS m/z (ESI):** calcd for $C_{24}H_{17}INa^+$ 455.0267, found 455.0264.

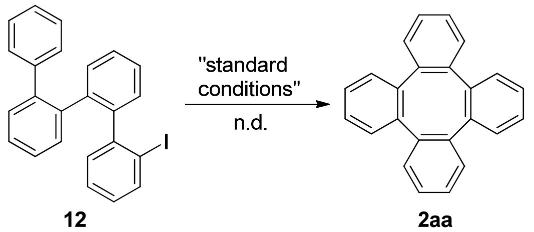

12 **2aa**

To a 10 mL Schlenk flask equipped with a magnetic stir bar was charged with $Pd(OAc)_2$ (2.3 mg, 0.01 mol, 5 mol%), **12** (86.4.0 mg, 0.20 mol, 1.0 equiv), $NaHCO_3$ (40 mg, 0.48 mmol, 2.4 equiv) and 2 mL DMF. Then the mixture was stirred at 130

^{o}C under air for 36 h. We observed that **2aa** could not form detected by GC-MS and crude ^{1}H NMR.

8. References

1. (a) Cho, S. Y.; Grimsdale, A. C.; Jones, D. J.; Watkins, S. E.; Holmes, A. B., Polyfluorenes without monoalkylfluorene defects. *J. Am. Chem. Soc.* **2007,** *129* (39), 11910-1; (b) Liu, Z.; Zhu, D.; Luo, B.; Zhang, N.; Liu, Q.; Hu, Y.; Pi, R.; Huang, P.; Wen, S., Mild Cu(I)-catalyzed cascade reaction of cyclic diaryliodoniums, sodium azide, and alkynes: efficient synthesis of triazolophenanthridines. *Org. lett* **2014,** *16* (21), 5600-3.

2. Das, B.; Venkateswarlu, K.; Majhi, A.; Siddaiah, V.; Reddy, K. R., A facile nuclear bromination of phenols and anilines using NBS in the presence of ammonium acetate as a catalyst. *J. Mol. Catal. A-Chem.* **2007,** *267* (1-2), 30-33.

3. Lux, J.; Chan, M.; Elst, L. V.; Schopf, E.; Mahmoud, E.; Laurent, S.; Almutairi, A., Metal Chelating Crosslinkers Form Nanogels with High Chelation Stability. *J. Mater. Chem. B.* **2013,** *1* (46), 6359-6364.

4. Barder, T. E.; Walker, S. D.; Martinelli, J. R.; Buchwald, S. L., Catalysts for Suzuki-Miyaura coupling processes: scope and studies of the effect of ligand structure. *J. Am. Chem. Soc.* **2005,** *127* (13), 4685-96.

5. Mathew, S. M.; Hartley, C. S., Parento-Phenylene Oligomers: Synthesis, Conformational Behavior, and Characterization. *Macromolecules* **2011,** *44* (21), 8425-8432.

9. X-ray crystallographic date and analysis.

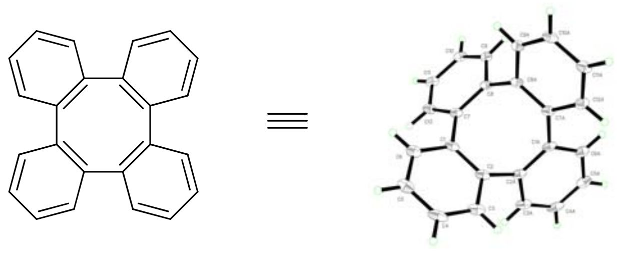

2aa

C(1)-C(2)	1.400(5)	C(4)-C(3)-H(3)	119.4
C(1)-C(6)	1.410(5)	C(2)-C(3)-H(3)	119.4
C(1)-C(7)	1.494(4)	C(5)-C(4)-C(3)	119.5(3)
C(2)-C(3)	1.399(4)	C(5)-C(4)-H(4)	120.2
C(2)-C(2)#1	1.502(7)	C(3)-C(4)-H(4)	120.2
C(3)-C(4)	1.396(5)	C(4)-C(5)-C(6)	120.4(4)
C(3)-H(3)	0.9300	C(4)-C(5)-H(5)	119.8
C(4)-C(5)	1.371(5)	C(6)-C(5)-H(5)	119.8
C(4)-H(4)	0.9300	C(5)-C(6)-C(1)	120.9(3)
C(5)-C(6)	1.383(5)	C(5)-C(6)-H(6)	119.6
C(5)-H(5)	0.9300	C(1)-C(6)-H(6)	119.6
C(6)-H(6)	0.9300	C(12)-C(7)-C(8)	118.6(3)
C(7)-C(12)	1.400(4)	C(12)-C(7)-C(1)	119.2(3)
C(7)-C(8)	1.411(4)	C(8)-C(7)-C(1)	122.1(3)
C(8)-C(9)	1.403(4)	C(9)-C(8)-C(7)	118.2(3)
C(8)-C(8)#1	1.479(6)	C(9)-C(8)-C(8)#1	118.0(2)
C(9)-C(10)	1.377(5)	C(7)-C(8)-C(8)#1	123.4(2)
C(9)-H(9)	0.9300	C(10)-C(9)-C(8)	122.2(3)
C(10)-C(11)	1.385(5)	C(10)-C(9)-H(9)	118.9
C(10)-H(10)	0.9300	C(8)-C(9)-H(9)	118.9
C(11)-C(12)	1.384(5)	C(9)-C(10)-C(11)	119.5(3)
C(11)-H(11)	0.9300	C(9)-C(10)-H(10)	120.3
C(12)-H(12)	0.9300	C(11)-C(10)-H(10)	120.3
C(2)-C(1)-C(6)	118.9(3)	C(12)-C(11)-C(10)	119.6(3)
C(2)-C(1)-C(7)	122.3(3)	C(12)-C(11)-H(11)	120.2
C(6)-C(1)-C(7)	118.6(3)	C(10)-C(11)-H(11)	120.2
C(3)-C(2)-C(1)	119.0(3)	C(11)-C(12)-C(7)	121.8(3)
C(3)-C(2)-C(2)#1	117.4(3)	C(11)-C(12)-H(12)	119.1
C(1)-C(2)-C(2)#1	123.4(2)	C(7)-C(12)-H(12)	119.1
C(4)-C(3)-C(2)	121.3(3)		

Symmetry transformations used to generate equivalent atoms:

#1 -x+3/2,-y+1/2,z

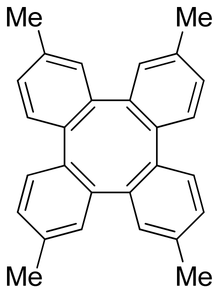

2bb

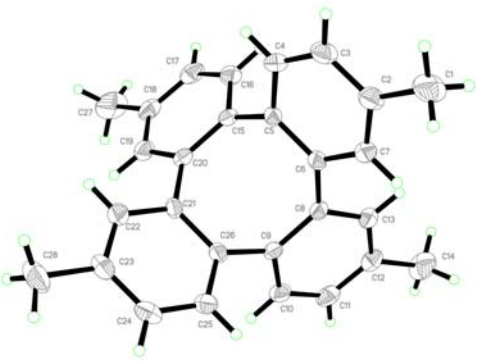

C(1)-C(2)	1.483(7)	C(17)-H(17)	0.9300
C(1)-H(1A)	0.9600	C(18)-C(19)	1.393(7)
C(1)-H(1B)	0.9600	C(18)-C(27)	1.490(7)
C(1)-H(1C)	0.9600	C(19)-C(20)	1.402(6)
C(2)-C(3)	1.377(7)	C(19)-H(19)	0.9300
C(2)-C(7)	1.392(6)	C(20)-C(21)	1.493(6)
C(3)-C(4)	1.382(7)	C(21)-C(22)	1.386(6)
C(3)-H(3)	0.9300	C(21)-C(26)	1.409(6)
C(4)-C(5)	1.422(6)	C(22)-C(23)	1.397(7)
C(4)-H(4)	0.9300	C(22)-H(22)	0.9300
C(5)-C(6)	1.399(6)	C(23)-C(24)	1.370(7)
C(5)-C(15)	1.472(6)	C(23)-C(28)	1.501(7)
C(6)-C(7)	1.401(6)	C(24)-C(25)	1.391(7)
C(6)-C(8)	1.460(6)	C(24)-H(24)	0.9300
C(7)-H(7)	0.9300	C(25)-C(26)	1.385(6)
C(8)-C(13)	1.397(6)	C(25)-H(25)	0.9300
C(8)-C(9)	1.404(6)	C(27)-H(27A)	0.9600
C(9)-C(10)	1.403(6)	C(27)-H(27B)	0.9600
C(9)-C(26)	1.480(6)	C(27)-H(27C)	0.9600
C(10)-C(11)	1.387(7)	C(28)-H(28A)	0.9600
C(10)-H(10)	0.9300	C(28)-H(28B)	0.9600
C(11)-C(12)	1.367(7)	C(28)-H(28C)	0.9600
C(11)-H(11)	0.9300	C(2)-C(1)-H(1A)	109.5
C(12)-C(13)	1.393(7)	C(2)-C(1)-H(1B)	109.5
C(12)-C(14)	1.518(7)	H(1A)-C(1)-H(1B)	109.5
C(13)-H(13)	0.9300	C(2)-C(1)-H(1C)	109.5
C(14)-H(14A)	0.9600	H(1A)-C(1)-H(1C)	109.5
C(14)-H(14B)	0.9600	H(1B)-C(1)-H(1C)	109.5
C(14)-H(14C)	0.9600	C(3)-C(2)-C(7)	117.8(4)
C(15)-C(20)	1.399(6)	C(3)-C(2)-C(1)	120.7(5)
C(15)-C(16)	1.401(6)	C(7)-C(2)-C(1)	121.5(5)
C(16)-C(17)	1.381(7)	C(2)-C(3)-C(4)	120.9(4)
C(16)-H(16)	0.9300	C(2)-C(3)-H(3)	119.6
C(17)-C(18)	1.378(7)	C(4)-C(3)-H(3)	119.6

C(3)-C(4)-C(5)	121.7(4)	C(16)-C(17)-C(18)	121.6(5)
C(3)-C(4)-H(4)	119.2	C(16)-C(17)-H(17)	119.2
C(5)-C(4)-H(4)	119.2	C(18)-C(17)-H(17)	119.2
C(4)-C(5)-C(6)	117.8(4)	C(19)-C(18)-C(17)	117.0(5)
C(4)-C(5)-C(15)	117.3(4)	C(19)-C(18)-C(27)	121.7(5)
C(6)-C(5)-C(15)	124.7(4)	C(17)-C(18)-C(27)	121.4(5)
C(7)-C(6)-C(5)	118.8(4)	C(18)-C(19)-C(20)	122.9(5)
C(7)-C(6)-C(8)	119.5(4)	C(18)-C(19)-H(19)	118.5
C(5)-C(6)-C(8)	121.5(4)	C(20)-C(19)-H(19)	118.5
C(6)-C(7)-C(2)	123.1(4)	C(15)-C(20)-C(19)	119.0(4)
C(6)-C(7)-H(7)	118.4	C(15)-C(20)-C(21)	122.9(4)
C(2)-C(7)-H(7)	118.4	C(19)-C(20)-C(21)	118.1(4)
C(13)-C(8)-C(9)	118.4(4)	C(22)-C(21)-C(26)	119.7(4)
C(13)-C(8)-C(6)	118.6(4)	C(22)-C(21)-C(20)	118.6(4)
C(9)-C(8)-C(6)	122.9(4)	C(26)-C(21)-C(20)	121.5(4)
C(8)-C(9)-C(10)	118.4(4)	C(23)-C(22)-C(21)	121.6(4)
C(8)-C(9)-C(26)	123.9(4)	C(23)-C(22)-H(22)	119.2
C(10)-C(9)-C(26)	117.5(4)	C(21)-C(22)-H(22)	119.2
C(11)-C(10)-C(9)	121.3(4)	C(24)-C(23)-C(22)	118.4(4)
C(11)-C(10)-H(10)	119.3	C(24)-C(23)-C(28)	120.2(5)
C(9)-C(10)-H(10)	119.3	C(22)-C(23)-C(28)	121.5(5)
C(12)-C(11)-C(10)	120.9(5)	C(23)-C(24)-C(25)	120.7(4)
C(12)-C(11)-H(11)	119.6	C(23)-C(24)-H(24)	119.7
C(10)-C(11)-H(11)	119.6	C(25)-C(24)-H(24)	119.7
C(11)-C(12)-C(13)	118.2(4)	C(24)-C(25)-C(26)	121.6(4)
C(11)-C(12)-C(14)	120.7(5)	C(24)-C(25)-H(25)	119.2
C(13)-C(12)-C(14)	121.0(5)	C(26)-C(25)-H(25)	119.2
C(12)-C(13)-C(8)	122.6(4)	C(25)-C(26)-C(21)	117.9(4)
C(12)-C(13)-H(13)	118.7	C(25)-C(26)-C(9)	118.8(4)
C(8)-C(13)-H(13)	118.7	C(21)-C(26)-C(9)	123.2(4)
C(12)-C(14)-H(14A)	109.5	C(18)-C(27)-H(27A)	109.5
C(12)-C(14)-H(14B)	109.5	C(18)-C(27)-H(27B)	109.5
H(14A)-C(14)-H(14B)	109.5	H(27A)-C(27)-H(27B)	109.5
C(12)-C(14)-H(14C)	109.5	C(18)-C(27)-H(27C)	109.5
H(14A)-C(14)-H(14C)	109.5	H(27A)-C(27)-H(27C)	109.5
H(14B)-C(14)-H(14C)	109.5	H(27B)-C(27)-H(27C)	109.5
C(20)-C(15)-C(16)	117.9(4)	C(23)-C(28)-H(28A)	109.5
C(20)-C(15)-C(5)	123.4(4)	C(23)-C(28)-H(28B)	109.5
C(16)-C(15)-C(5)	118.6(4)	H(28A)-C(28)-H(28B)	109.5
C(17)-C(16)-C(15)	121.7(5)	C(23)-C(28)-H(28C)	109.5
C(17)-C(16)-H(16)	119.2	H(28A)-C(28)-H(28C)	109.5
C(15)-C(16)-H(16)	119.2	H(28B)-C(28)-H(28C)	109.5

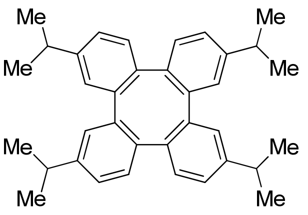

2dd

≡

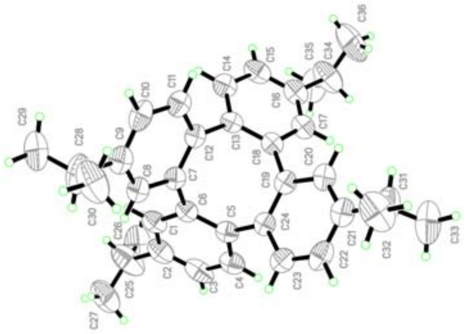

C(1)-C(2)	1.353(6)	C(19)-C(20)	1.409(5)
C(1)-C(6)	1.394(5)	C(20)-C(21)	1.340(6)
C(1)-H(1)	0.9300	C(20)-H(20)	0.9300
C(2)-C(3)	1.376(7)	C(21)-C(22)	1.388(6)
C(2)-C(25)	1.512(7)	C(21)-C(31)	1.524(6)
C(3)-C(4)	1.394(6)	C(22)-C(23)	1.383(6)
C(3)-H(3)	0.9300	C(22)-H(22)	0.9300
C(4)-C(5)	1.398(6)	C(23)-C(24)	1.385(5)
C(4)-H(4)	0.9300	C(23)-H(23)	0.9300
C(5)-C(6)	1.383(5)	C(25)-C(27)	1.373(7)
C(5)-C(24)	1.490(5)	C(25)-C(26)	1.437(7)
C(6)-C(7)	1.481(5)	C(25)-H(25)	0.9800
C(7)-C(12)	1.382(5)	C(26)-H(26A)	0.9600
C(7)-C(8)	1.399(5)	C(26)-H(26B)	0.9600
C(8)-C(9)	1.344(6)	C(26)-H(26C)	0.9600
C(8)-H(8)	0.9300	C(27)-H(27A)	0.9600
C(9)-C(10)	1.389(6)	C(27)-H(27B)	0.9600
C(9)-C(28)	1.523(7)	C(27)-H(27C)	0.9600
C(10)-C(11)	1.418(6)	C(28)-C(29)	1.389(7)
C(10)-H(10)	0.9300	C(28)-C(30)	1.480(7)
C(11)-C(12)	1.406(5)	C(28)-H(28)	0.9800
C(11)-H(11)	0.9300	C(29)-H(29A)	0.9600
C(12)-C(13)	1.488(5)	C(29)-H(29B)	0.9600
C(13)-C(18)	1.392(5)	C(29)-H(29C)	0.9600
C(13)-C(14)	1.392(5)	C(30)-H(30A)	0.9600
C(14)-C(15)	1.409(6)	C(30)-H(30B)	0.9600
C(14)-H(14)	0.9300	C(30)-H(30C)	0.9600
C(15)-C(16)	1.370(6)	C(31)-C(33)	1.383(7)
C(15)-H(15)	0.9300	C(31)-C(32)	1.479(7)
C(16)-C(17)	1.357(6)	C(31)-H(31)	0.9800
C(16)-C(34)	1.520(7)	C(32)-H(32A)	0.9600
C(17)-C(18)	1.401(5)	C(32)-H(32B)	0.9600
C(17)-H(17)	0.9300	C(32)-H(32C)	0.9600
C(18)-C(19)	1.471(5)	C(33)-H(33A)	0.9600
C(19)-C(24)	1.385(5)	C(33)-H(33B)	0.9600

C(33)-H(33C)	0.9600	C(7)-C(12)-C(13)	123.2(4)
C(34)-C(36)	1.365(7)	C(11)-C(12)-C(13)	117.9(4)
C(34)-C(35)	1.486(7)	C(18)-C(13)-C(14)	118.6(4)
C(34)-H(34)	0.9800	C(18)-C(13)-C(12)	123.1(4)
C(35)-H(35A)	0.9600	C(14)-C(13)-C(12)	118.2(4)
C(35)-H(35B)	0.9600	C(13)-C(14)-C(15)	120.3(4)
C(35)-H(35C)	0.9600	C(13)-C(14)-H(14)	119.9
C(36)-H(36A)	0.9600	C(15)-C(14)-H(14)	119.9
C(36)-H(36B)	0.9600	C(16)-C(15)-C(14)	121.0(5)
C(36)-H(36C)	0.9600	C(16)-C(15)-H(15)	119.5
C(2)-C(1)-C(6)	123.1(5)	C(14)-C(15)-H(15)	119.5
C(2)-C(1)-H(1)	118.5	C(17)-C(16)-C(15)	118.0(5)
C(6)-C(1)-H(1)	118.5	C(17)-C(16)-C(34)	121.8(5)
C(1)-C(2)-C(3)	117.0(5)	C(15)-C(16)-C(34)	120.2(5)
C(1)-C(2)-C(25)	122.5(6)	C(16)-C(17)-C(18)	123.3(5)
C(3)-C(2)-C(25)	120.4(6)	C(16)-C(17)-H(17)	118.3
C(2)-C(3)-C(4)	121.9(5)	C(18)-C(17)-H(17)	118.3
C(2)-C(3)-H(3)	119.0	C(13)-C(18)-C(17)	118.7(4)
C(4)-C(3)-H(3)	119.0	C(13)-C(18)-C(19)	123.3(4)
C(3)-C(4)-C(5)	120.3(5)	C(17)-C(18)-C(19)	117.8(4)
C(3)-C(4)-H(4)	119.8	C(24)-C(19)-C(20)	119.0(4)
C(5)-C(4)-H(4)	119.8	C(24)-C(19)-C(18)	123.4(4)
C(6)-C(5)-C(4)	117.4(4)	C(20)-C(19)-C(18)	117.4(4)
C(6)-C(5)-C(24)	123.6(4)	C(21)-C(20)-C(19)	123.2(5)
C(4)-C(5)-C(24)	118.9(4)	C(21)-C(20)-H(20)	118.4
C(5)-C(6)-C(1)	120.2(4)	C(19)-C(20)-H(20)	118.4
C(5)-C(6)-C(7)	122.3(4)	C(20)-C(21)-C(22)	117.7(5)
C(1)-C(6)-C(7)	117.4(4)	C(20)-C(21)-C(31)	122.2(5)
C(12)-C(7)-C(8)	119.5(4)	C(22)-C(21)-C(31)	120.2(5)
C(12)-C(7)-C(6)	123.3(4)	C(23)-C(22)-C(21)	120.7(5)
C(8)-C(7)-C(6)	117.0(4)	C(23)-C(22)-H(22)	119.6
C(9)-C(8)-C(7)	123.2(5)	C(21)-C(22)-H(22)	119.6
C(9)-C(8)-H(8)	118.4	C(22)-C(23)-C(24)	121.5(5)
C(7)-C(8)-H(8)	118.4	C(22)-C(23)-H(23)	119.2
C(8)-C(9)-C(10)	118.4(5)	C(24)-C(23)-H(23)	119.2
C(8)-C(9)-C(28)	122.0(5)	C(19)-C(24)-C(23)	117.9(4)
C(10)-C(9)-C(28)	119.6(5)	C(19)-C(24)-C(5)	122.6(4)
C(9)-C(10)-C(11)	120.5(5)	C(23)-C(24)-C(5)	119.4(4)
C(9)-C(10)-H(10)	119.7	C(27)-C(25)-C(26)	124.2(6)
C(11)-C(10)-H(10)	119.7	C(27)-C(25)-C(2)	115.2(6)
C(12)-C(11)-C(10)	119.6(5)	C(26)-C(25)-C(2)	113.7(5)
C(12)-C(11)-H(11)	120.2	C(27)-C(25)-H(25)	98.8
C(10)-C(11)-H(11)	120.2	C(26)-C(25)-H(25)	98.8
C(7)-C(12)-C(11)	118.8(4)	C(2)-C(25)-H(25)	98.8

C(25)-C(26)-H(26A)	109.5	C(33)-C(31)-H(31)	101.0
C(25)-C(26)-H(26B)	109.5	C(32)-C(31)-H(31)	101.0
H(26A)-C(26)-H(26B)	109.5	C(21)-C(31)-H(31)	101.0
C(25)-C(26)-H(26C)	109.5	C(31)-C(32)-H(32A)	109.5
H(26A)-C(26)-H(26C)	109.5	C(31)-C(32)-H(32B)	109.5
H(26B)-C(26)-H(26C)	109.5	H(32A)-C(32)-H(32B)	109.5
C(25)-C(27)-H(27A)	109.5	C(31)-C(32)-H(32C)	109.5
C(25)-C(27)-H(27B)	109.5	H(32A)-C(32)-H(32C)	109.5
H(27A)-C(27)-H(27B)	109.5	H(32B)-C(32)-H(32C)	109.5
C(25)-C(27)-H(27C)	109.5	C(31)-C(33)-H(33A)	109.5
H(27A)-C(27)-H(27C)	109.5	C(31)-C(33)-H(33B)	109.5
H(27B)-C(27)-H(27C)	109.5	H(33A)-C(33)-H(33B)	109.5
C(29)-C(28)-C(30)	122.5(6)	C(31)-C(33)-H(33C)	109.5
C(29)-C(28)-C(9)	114.0(5)	H(33A)-C(33)-H(33C)	109.5
C(30)-C(28)-C(9)	111.1(5)	H(33B)-C(33)-H(33C)	109.5
C(29)-C(28)-H(28)	101.9	C(36)-C(34)-C(35)	124.4(6)
C(30)-C(28)-H(28)	101.9	C(36)-C(34)-C(16)	115.4(6)
C(9)-C(28)-H(28)	101.9	C(35)-C(34)-C(16)	111.5(5)
C(28)-C(29)-H(29A)	109.5	C(36)-C(34)-H(34)	99.8
C(28)-C(29)-H(29B)	109.5	C(35)-C(34)-H(34)	99.8
H(29A)-C(29)-H(29B)	109.5	C(16)-C(34)-H(34)	99.8
C(28)-C(29)-H(29C)	109.5	C(34)-C(35)-H(35A)	109.5
H(29A)-C(29)-H(29C)	109.5	C(34)-C(35)-H(35B)	109.5
H(29B)-C(29)-H(29C)	109.5	H(35A)-C(35)-H(35B)	109.5
C(28)-C(30)-H(30A)	109.5	C(34)-C(35)-H(35C)	109.5
C(28)-C(30)-H(30B)	109.5	H(35A)-C(35)-H(35C)	109.5
H(30A)-C(30)-H(30B)	109.5	H(35B)-C(35)-H(35C)	109.5
C(28)-C(30)-H(30C)	109.5	C(34)-C(36)-H(36A)	109.5
H(30A)-C(30)-H(30C)	109.5	C(34)-C(36)-H(36B)	109.5
H(30B)-C(30)-H(30C)	109.5	H(36A)-C(36)-H(36B)	109.5
C(33)-C(31)-C(32)	122.6(5)	C(34)-C(36)-H(36C)	109.5
C(33)-C(31)-C(21)	115.2(5)	H(36A)-C(36)-H(36C)	109.5
C(32)-C(31)-C(21)	111.4(5)	H(36B)-C(36)-H(36C)	109.5

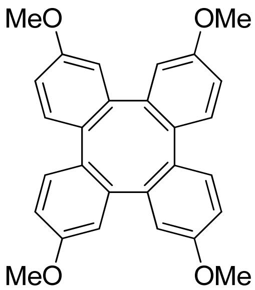

2ee

≡

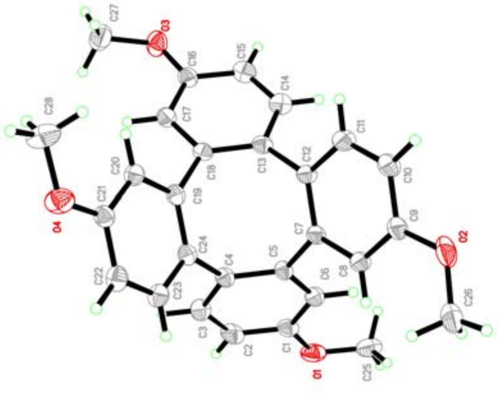

C(1)-O(1)	1.369(3)	C(25)-O(1)	1.412(3)
C(1)-C(2)	1.380(3)	C(25)-H(25A)	0.9600
C(1)-C(6)	1.381(3)	C(25)-H(25B)	0.9600
C(2)-C(3)	1.371(3)	C(25)-H(25C)	0.9600
C(2)-H(2)	0.9300	C(26)-O(2)	1.419(3)
C(3)-C(4)	1.402(3)	C(26)-H(26A)	0.9600
C(3)-H(3)	0.9300	C(26)-H(26B)	0.9600
C(4)-C(5)	1.387(3)	C(26)-H(26C)	0.9600
C(4)-C(24)	1.486(3)	C(27)-O(3)	1.407(3)
C(5)-C(6)	1.394(3)	C(27)-H(27A)	0.9600
C(5)-C(7)	1.495(3)	C(27)-H(27B)	0.9600
C(6)-H(6)	0.9300	C(27)-H(27C)	0.9600
C(7)-C(8)	1.394(3)	C(28)-O(4)	1.396(3)
C(7)-C(12)	1.399(3)	C(28)-H(28A)	0.9600
C(8)-C(9)	1.385(3)	C(28)-H(28B)	0.9600
C(8)-H(8)	0.9300	C(28)-H(28C)	0.9600
C(9)-O(2)	1.362(3)	O(1)-C(1)-C(2)	115.72(19)
C(9)-C(10)	1.382(3)	O(1)-C(1)-C(6)	124.6(2)
C(10)-C(11)	1.366(3)	C(2)-C(1)-C(6)	119.7(2)
C(10)-H(10)	0.9300	C(3)-C(2)-C(1)	120.4(2)
C(11)-C(12)	1.398(3)	C(3)-C(2)-H(2)	119.8
C(11)-H(11)	0.9300	C(1)-C(2)-H(2)	119.8
C(12)-C(13)	1.488(3)	C(2)-C(3)-C(4)	120.9(2)
C(13)-C(14)	1.398(3)	C(2)-C(3)-H(3)	119.6
C(13)-C(18)	1.402(3)	C(4)-C(3)-H(3)	119.6
C(14)-C(15)	1.374(3)	C(5)-C(4)-C(3)	118.5(2)
C(14)-H(14)	0.9300	C(5)-C(4)-C(24)	122.37(18)
C(15)-C(16)	1.380(3)	C(3)-C(4)-C(24)	118.93(19)
C(15)-H(15)	0.9300	C(4)-C(5)-C(6)	120.27(19)
C(16)-O(3)	1.368(3)	C(4)-C(5)-C(7)	121.44(19)
C(16)-C(17)	1.389(3)	C(6)-C(5)-C(7)	118.25(19)
C(17)-C(18)	1.394(3)	C(1)-C(6)-C(5)	120.2(2)
C(17)-H(17)	0.9300	C(1)-C(6)-H(6)	119.9
C(18)-C(19)	1.496(3)	C(5)-C(6)-H(6)	119.9
C(19)-C(20)	1.391(3)	C(8)-C(7)-C(12)	120.01(19)
C(19)-C(24)	1.399(3)	C(8)-C(7)-C(5)	118.71(19)
C(20)-C(21)	1.380(3)	C(12)-C(7)-C(5)	121.20(19)
C(20)-H(20)	0.9300	C(9)-C(8)-C(7)	121.0(2)
C(21)-O(4)	1.368(3)	C(9)-C(8)-H(8)	119.5
C(21)-C(22)	1.384(3)	C(7)-C(8)-H(8)	119.5
C(22)-C(23)	1.365(3)	O(2)-C(9)-C(10)	116.0(2)
C(22)-H(22)	0.9300	O(2)-C(9)-C(8)	124.9(2)
C(23)-C(24)	1.398(3)	C(10)-C(9)-C(8)	119.1(2)
C(23)-H(23)	0.9300	C(11)-C(10)-C(9)	120.1(2)

C(11)-C(10)-H(10)	120.0	C(23)-C(22)-H(22)	120.1
C(9)-C(10)-H(10)	120.0	C(21)-C(22)-H(22)	120.1
C(10)-C(11)-C(12)	122.4(2)	C(22)-C(23)-C(24)	121.9(2)
C(10)-C(11)-H(11)	118.8	C(22)-C(23)-H(23)	119.1
C(12)-C(11)-H(11)	118.8	C(24)-C(23)-H(23)	119.1
C(11)-C(12)-C(7)	117.4(2)	C(23)-C(24)-C(19)	118.2(2)
C(11)-C(12)-C(13)	118.33(19)	C(23)-C(24)-C(4)	117.86(19)
C(7)-C(12)-C(13)	124.25(18)	C(19)-C(24)-C(4)	123.94(19)
C(14)-C(13)-C(18)	117.5(2)	O(1)-C(25)-H(25A)	109.5
C(14)-C(13)-C(12)	118.9(2)	O(1)-C(25)-H(25B)	109.5
C(18)-C(13)-C(12)	123.1(2)	H(25A)-C(25)-H(25B)	109.5
C(15)-C(14)-C(13)	122.5(2)	O(1)-C(25)-H(25C)	109.5
C(15)-C(14)-H(14)	118.8	H(25A)-C(25)-H(25C)	109.5
C(13)-C(14)-H(14)	118.8	H(25B)-C(25)-H(25C)	109.5
C(14)-C(15)-C(16)	119.6(2)	O(2)-C(26)-H(26A)	109.5
C(14)-C(15)-H(15)	120.2	O(2)-C(26)-H(26B)	109.5
C(16)-C(15)-H(15)	120.2	H(26A)-C(26)-H(26B)	109.5
O(3)-C(16)-C(15)	116.0(2)	O(2)-C(26)-H(26C)	109.5
O(3)-C(16)-C(17)	124.5(2)	H(26A)-C(26)-H(26C)	109.5
C(15)-C(16)-C(17)	119.5(2)	H(26B)-C(26)-H(26C)	109.5
C(16)-C(17)-C(18)	120.9(2)	O(3)-C(27)-H(27A)	109.5
C(16)-C(17)-H(17)	119.6	O(3)-C(27)-H(27B)	109.5
C(18)-C(17)-H(17)	119.6	H(27A)-C(27)-H(27B)	109.5
C(17)-C(18)-C(13)	119.9(2)	O(3)-C(27)-H(27C)	109.5
C(17)-C(18)-C(19)	117.7(2)	H(27A)-C(27)-H(27C)	109.5
C(13)-C(18)-C(19)	122.2(2)	H(27B)-C(27)-H(27C)	109.5
C(20)-C(19)-C(24)	119.54(19)	O(4)-C(28)-H(28A)	109.5
C(20)-C(19)-C(18)	118.81(18)	O(4)-C(28)-H(28B)	109.5
C(24)-C(19)-C(18)	121.60(19)	H(28A)-C(28)-H(28B)	109.5
C(21)-C(20)-C(19)	121.0(2)	O(4)-C(28)-H(28C)	109.5
C(21)-C(20)-H(20)	119.5	H(28A)-C(28)-H(28C)	109.5
C(19)-C(20)-H(20)	119.5	H(28B)-C(28)-H(28C)	109.5
O(4)-C(21)-C(20)	125.4(2)	C(1)-O(1)-C(25)	117.43(17)
O(4)-C(21)-C(22)	115.0(2)	C(9)-O(2)-C(26)	118.12(18)
C(20)-C(21)-C(22)	119.6(2)	C(16)-O(3)-C(27)	117.9(2)
C(23)-C(22)-C(21)	119.8(2)	C(21)-O(4)-C(28)	118.85(19)

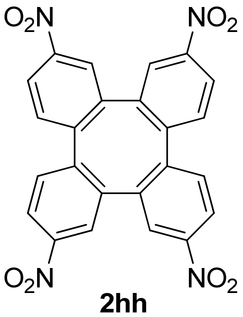

2hh

≡

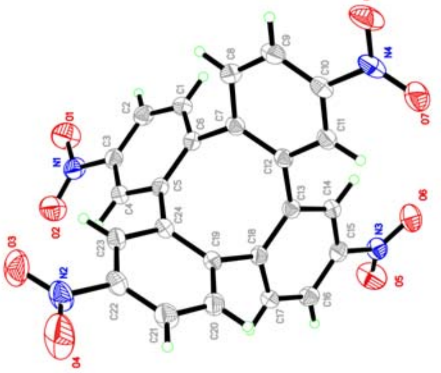

C(1)-C(2)	1.373(4)	C(19)-C(20)	1.398(4)
C(1)-C(6)	1.390(3)	C(19)-C(24)	1.401(3)
C(1)-H(1)	0.9300	C(20)-C(21)	1.380(4)
C(2)-C(3)	1.380(4)	C(20)-H(20)	0.9300
C(2)-H(2)	0.9300	C(21)-C(22)	1.372(4)
C(3)-C(4)	1.377(4)	C(21)-H(21)	0.9300
C(3)-N(1)	1.470(3)	C(22)-C(23)	1.381(4)
C(4)-C(5)	1.394(3)	C(22)-N(2)	1.469(4)
C(4)-H(4)	0.9300	C(23)-C(24)	1.388(3)
C(5)-C(6)	1.398(3)	C(23)-H(23)	0.9300
C(5)-C(24)	1.495(3)	N(1)-O(2)	1.210(4)
C(6)-C(7)	1.485(3)	N(1)-O(1)	1.218(3)
C(7)-C(8)	1.404(4)	N(2)-O(4)	1.185(4)
C(7)-C(12)	1.410(3)	N(2)-O(3)	1.214(4)
C(8)-C(9)	1.380(4)	N(3)-O(6)	1.208(3)
C(8)-H(8)	0.9300	N(3)-O(5)	1.212(3)
C(9)-C(10)	1.368(4)	N(4)-O(7)	1.212(4)
C(9)-H(9)	0.9300	N(4)-O(8)	1.215(3)
C(10)-C(11)	1.369(4)	C(2)-C(1)-C(6)	121.8(2)
C(10)-N(4)	1.479(3)	C(2)-C(1)-H(1)	119.1
C(11)-C(12)	1.393(3)	C(6)-C(1)-H(1)	119.1
C(11)-H(11)	0.9300	C(1)-C(2)-C(3)	117.8(2)
C(12)-C(13)	1.488(3)	C(1)-C(2)-H(2)	121.1
C(13)-C(14)	1.389(3)	C(3)-C(2)-H(2)	121.1
C(13)-C(18)	1.403(3)	C(4)-C(3)-C(2)	122.4(2)
C(14)-C(15)	1.372(4)	C(4)-C(3)-N(1)	118.0(2)
C(14)-H(14)	0.9300	C(2)-C(3)-N(1)	119.6(2)
C(15)-C(16)	1.377(4)	C(3)-C(4)-C(5)	119.6(2)
C(15)-N(3)	1.474(3)	C(3)-C(4)-H(4)	120.2
C(16)-C(17)	1.376(4)	C(5)-C(4)-H(4)	120.2
C(16)-H(16)	0.9300	C(4)-C(5)-C(6)	118.8(2)
C(17)-C(18)	1.398(4)	C(4)-C(5)-C(24)	117.8(2)
C(17)-H(17)	0.9300	C(6)-C(5)-C(24)	123.2(2)
C(18)-C(19)	1.483(3)	C(1)-C(6)-C(5)	119.7(2)

C(1)-C(6)-C(7)	118.0(2)	C(18)-C(17)-H(17)	119.2
C(5)-C(6)-C(7)	122.1(2)	C(17)-C(18)-C(13)	119.1(2)
C(8)-C(7)-C(12)	118.7(2)	C(17)-C(18)-C(19)	118.4(2)
C(8)-C(7)-C(6)	118.5(2)	C(13)-C(18)-C(19)	122.4(2)
C(12)-C(7)-C(6)	122.7(2)	C(20)-C(19)-C(24)	119.1(2)
C(9)-C(8)-C(7)	121.7(2)	C(20)-C(19)-C(18)	119.0(2)
C(9)-C(8)-H(8)	119.2	C(24)-C(19)-C(18)	121.8(2)
C(7)-C(8)-H(8)	119.2	C(21)-C(20)-C(19)	121.6(3)
C(10)-C(9)-C(8)	118.1(2)	C(21)-C(20)-H(20)	119.2
C(10)-C(9)-H(9)	120.9	C(19)-C(20)-H(20)	119.2
C(8)-C(9)-H(9)	120.9	C(22)-C(21)-C(20)	117.9(2)
C(9)-C(10)-C(11)	122.5(2)	C(22)-C(21)-H(21)	121.1
C(9)-C(10)-N(4)	119.3(2)	C(20)-C(21)-H(21)	121.1
C(11)-C(10)-N(4)	118.3(2)	C(21)-C(22)-C(23)	122.4(2)
C(10)-C(11)-C(12)	120.3(2)	C(21)-C(22)-N(2)	119.0(2)
C(10)-C(11)-H(11)	119.9	C(23)-C(22)-N(2)	118.6(3)
C(12)-C(11)-H(11)	119.9	C(22)-C(23)-C(24)	119.7(2)
C(11)-C(12)-C(7)	118.7(2)	C(22)-C(23)-H(23)	120.2
C(11)-C(12)-C(13)	119.6(2)	C(24)-C(23)-H(23)	120.2
C(7)-C(12)-C(13)	121.5(2)	C(23)-C(24)-C(19)	119.2(2)
C(14)-C(13)-C(18)	118.9(2)	C(23)-C(24)-C(5)	118.1(2)
C(14)-C(13)-C(12)	118.7(2)	C(19)-C(24)-C(5)	122.4(2)
C(18)-C(13)-C(12)	122.4(2)	O(2)-N(1)-O(1)	123.4(3)
C(15)-C(14)-C(13)	120.0(2)	O(2)-N(1)-C(3)	118.3(2)
C(15)-C(14)-H(14)	120.0	O(1)-N(1)-C(3)	118.3(3)
C(13)-C(14)-H(14)	120.0	O(4)-N(2)-O(3)	121.8(3)
C(14)-C(15)-C(16)	122.3(2)	O(4)-N(2)-C(22)	119.5(3)
C(14)-C(15)-N(3)	118.9(2)	O(3)-N(2)-C(22)	118.5(3)
C(16)-C(15)-N(3)	118.8(2)	O(6)-N(3)-O(5)	123.9(2)
C(17)-C(16)-C(15)	117.8(2)	O(6)-N(3)-C(15)	118.0(2)
C(17)-C(16)-H(16)	121.1	O(5)-N(3)-C(15)	118.1(2)
C(15)-C(16)-H(16)	121.1	O(7)-N(4)-O(8)	123.5(3)
C(16)-C(17)-C(18)	121.7(2)	O(7)-N(4)-C(10)	118.8(2)
C(16)-C(17)-H(17)	119.2	O(8)-N(4)-C(10)	117.7(3)

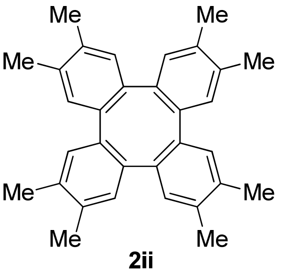

2ii

≡

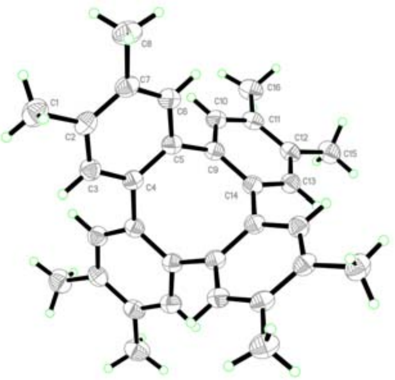

C(1)-C(2)	1.501(3)	C(16)-H(16C)	0.9600
C(1)-H(1A)	0.9600	C(2)-C(1)-H(1A)	109.5
C(1)-H(1B)	0.9600	C(2)-C(1)-H(1B)	109.5
C(1)-H(1C)	0.9600	H(1A)-C(1)-H(1B)	109.5
C(2)-C(7)	1.384(3)	C(2)-C(1)-H(1C)	109.5
C(2)-C(3)	1.388(3)	H(1A)-C(1)-H(1C)	109.5
C(3)-C(4)	1.388(3)	H(1B)-C(1)-H(1C)	109.5
C(3)-H(3)	0.9300	C(7)-C(2)-C(3)	118.5(2)
C(4)-C(5)	1.388(3)	C(7)-C(2)-C(1)	121.3(2)
C(4)-C(4)#1	1.489(4)	C(3)-C(2)-C(1)	120.2(2)
C(5)-C(6)	1.387(3)	C(2)-C(3)-C(4)	122.7(2)
C(5)-C(9)	1.491(3)	C(2)-C(3)-H(3)	118.6
C(6)-C(7)	1.383(3)	C(4)-C(3)-H(3)	118.6
C(6)-H(6)	0.9300	C(5)-C(4)-C(3)	118.5(2)
C(7)-C(8)	1.502(4)	C(5)-C(4)-C(4)#1	121.31(16)
C(8)-H(8A)	0.9600	C(3)-C(4)-C(4)#1	120.14(17)
C(8)-H(8B)	0.9600	C(6)-C(5)-C(4)	118.6(2)
C(8)-H(8C)	0.9600	C(6)-C(5)-C(9)	118.40(19)
C(9)-C(10)	1.387(3)	C(4)-C(5)-C(9)	122.9(2)
C(9)-C(14)	1.394(3)	C(7)-C(6)-C(5)	122.7(2)
C(10)-C(11)	1.385(3)	C(7)-C(6)-H(6)	118.6
C(10)-H(10)	0.9300	C(5)-C(6)-H(6)	118.6
C(11)-C(12)	1.389(3)	C(6)-C(7)-C(2)	118.9(2)
C(11)-C(16)	1.499(3)	C(6)-C(7)-C(8)	119.5(2)
C(12)-C(13)	1.379(3)	C(2)-C(7)-C(8)	121.6(2)
C(12)-C(15)	1.496(3)	C(7)-C(8)-H(8A)	109.5
C(13)-C(14)	1.392(3)	C(7)-C(8)-H(8B)	109.5
C(13)-H(13)	0.9300	H(8A)-C(8)-H(8B)	109.5
C(14)-C(14)#1	1.479(4)	C(7)-C(8)-H(8C)	109.5
C(15)-H(15A)	0.9600	H(8A)-C(8)-H(8C)	109.5
C(15)-H(15B)	0.9600	H(8B)-C(8)-H(8C)	109.5
C(15)-H(15C)	0.9600	C(10)-C(9)-C(14)	118.9(2)
C(16)-H(16A)	0.9600	C(10)-C(9)-C(5)	118.55(19)
C(16)-H(16B)	0.9600	C(14)-C(9)-C(5)	122.36(19)

C(11)-C(10)-C(9)	122.9(2)	C(9)-C(14)-C(14)#1	122.36(16)
C(11)-C(10)-H(10)	118.6	C(12)-C(15)-H(15A)	109.5
C(9)-C(10)-H(10)	118.6	C(12)-C(15)-H(15B)	109.5
C(10)-C(11)-C(12)	118.3(2)	H(15A)-C(15)-H(15B)	109.5
C(10)-C(11)-C(16)	120.3(2)	C(12)-C(15)-H(15C)	109.5
C(12)-C(11)-C(16)	121.3(2)	H(15A)-C(15)-H(15C)	109.5
C(13)-C(12)-C(11)	118.8(2)	H(15B)-C(15)-H(15C)	109.5
C(13)-C(12)-C(15)	120.8(2)	C(11)-C(16)-H(16A)	109.5
C(11)-C(12)-C(15)	120.5(2)	C(11)-C(16)-H(16B)	109.5
C(12)-C(13)-C(14)	123.3(2)	H(16A)-C(16)-H(16B)	109.5
C(12)-C(13)-H(13)	118.3	C(11)-C(16)-H(16C)	109.5
C(14)-C(13)-H(13)	118.3	H(16A)-C(16)-H(16C)	109.5
C(13)-C(14)-C(9)	117.7(2)	H(16B)-C(16)-H(16C)	109.5
C(13)-C(14)-C(14)#1	119.93(17)		

Symmetry transformations used to generate equivalent atoms:

#1 -x+3/2,y,-z+3/2

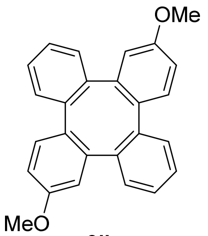

3jj

≡

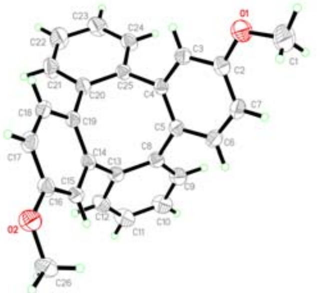

C(1)-O(1)	1.415(4)	C(9)-C(10)	1.380(5)
C(1)-H(1A)	0.9600	C(9)-H(9)	0.9300
C(1)-H(1B)	0.9600	C(10)-C(11)	1.382(5)
C(1)-H(1C)	0.9600	C(10)-H(10)	0.9300
C(2)-O(1)	1.351(4)	C(11)-C(12)	1.389(5)
C(2)-C(3)	1.383(4)	C(11)-H(11)	0.9300
C(2)-C(7)	1.386(4)	C(12)-C(13)	1.387(4)
C(3)-C(4)	1.389(4)	C(12)-H(12)	0.9300
C(3)-H(3)	0.9300	C(13)-C(14)	1.501(4)
C(4)-C(5)	1.404(4)	C(14)-C(15)	1.399(4)
C(4)-C(25)	1.503(4)	C(14)-C(19)	1.402(4)
C(5)-C(6)	1.393(4)	C(15)-C(16)	1.382(4)
C(5)-C(8)	1.492(4)	C(15)-H(15)	0.9300
C(6)-C(7)	1.387(4)	C(16)-O(2)	1.356(4)
C(6)-H(6)	0.9300	C(16)-C(17)	1.395(4)
C(7)-H(7)	0.9300	C(17)-C(18)	1.370(4)
C(8)-C(13)	1.400(4)	C(17)-H(17)	0.9300
C(8)-C(9)	1.400(4)	C(18)-C(19)	1.398(4)

C(18)-H(18)	0.9300	C(10)-C(9)-H(9)	119.3
C(19)-C(20)	1.491(4)	C(8)-C(9)-H(9)	119.3
C(20)-C(21)	1.396(4)	C(9)-C(10)-C(11)	120.3(3)
C(20)-C(25)	1.398(4)	C(9)-C(10)-H(10)	119.9
C(21)-C(22)	1.378(5)	C(11)-C(10)-H(10)	119.9
C(21)-H(21)	0.9300	C(10)-C(11)-C(12)	118.7(3)
C(22)-C(23)	1.373(5)	C(10)-C(11)-H(11)	120.6
C(22)-H(22)	0.9300	C(12)-C(11)-H(11)	120.6
C(23)-C(24)	1.379(5)	C(13)-C(12)-C(11)	121.9(3)
C(23)-H(23)	0.9300	C(13)-C(12)-H(12)	119.1
C(24)-C(25)	1.394(4)	C(11)-C(12)-H(12)	119.1
C(24)-H(24)	0.9300	C(12)-C(13)-C(8)	119.2(3)
C(26)-O(2)	1.424(4)	C(12)-C(13)-C(14)	118.0(3)
C(26)-H(26A)	0.9600	C(8)-C(13)-C(14)	122.4(3)
C(26)-H(26B)	0.9600	C(15)-C(14)-C(19)	119.9(3)
C(26)-H(26C)	0.9600	C(15)-C(14)-C(13)	117.1(3)
O(1)-C(1)-H(1A)	109.5	C(19)-C(14)-C(13)	122.9(3)
O(1)-C(1)-H(1B)	109.5	C(16)-C(15)-C(14)	121.2(3)
H(1A)-C(1)-H(1B)	109.5	C(16)-C(15)-H(15)	119.4
O(1)-C(1)-H(1C)	109.5	C(14)-C(15)-H(15)	119.4
H(1A)-C(1)-H(1C)	109.5	O(2)-C(16)-C(15)	124.2(3)
H(1B)-C(1)-H(1C)	109.5	O(2)-C(16)-C(17)	116.9(3)
O(1)-C(2)-C(3)	115.3(3)	C(15)-C(16)-C(17)	118.9(3)
O(1)-C(2)-C(7)	125.3(3)	C(18)-C(17)-C(16)	120.1(3)
C(3)-C(2)-C(7)	119.4(3)	C(18)-C(17)-H(17)	120.0
C(2)-C(3)-C(4)	121.9(3)	C(16)-C(17)-H(17)	120.0
C(2)-C(3)-H(3)	119.0	C(17)-C(18)-C(19)	122.2(3)
C(4)-C(3)-H(3)	119.0	C(17)-C(18)-H(18)	118.9
C(3)-C(4)-C(5)	119.3(3)	C(19)-C(18)-H(18)	118.9
C(3)-C(4)-C(25)	117.3(3)	C(18)-C(19)-C(14)	117.7(3)
C(5)-C(4)-C(25)	123.4(3)	C(18)-C(19)-C(20)	119.5(3)
C(6)-C(5)-C(4)	117.9(3)	C(14)-C(19)-C(20)	122.7(3)
C(6)-C(5)-C(8)	119.4(3)	C(21)-C(20)-C(25)	118.8(3)
C(4)-C(5)-C(8)	122.7(3)	C(21)-C(20)-C(19)	119.0(3)
C(7)-C(6)-C(5)	122.6(3)	C(25)-C(20)-C(19)	122.1(3)
C(7)-C(6)-H(6)	118.7	C(22)-C(21)-C(20)	121.0(3)
C(5)-C(6)-H(6)	118.7	C(22)-C(21)-H(21)	119.5
C(6)-C(7)-C(2)	118.9(3)	C(20)-C(21)-H(21)	119.5
C(6)-C(7)-H(7)	120.6	C(23)-C(22)-C(21)	120.0(3)
C(2)-C(7)-H(7)	120.6	C(23)-C(22)-H(22)	120.0
C(13)-C(8)-C(9)	118.5(3)	C(21)-C(22)-H(22)	120.0
C(13)-C(8)-C(5)	122.0(3)	C(22)-C(23)-C(24)	120.0(3)
C(9)-C(8)-C(5)	119.4(3)	C(22)-C(23)-H(23)	120.0
C(10)-C(9)-C(8)	121.3(3)	C(24)-C(23)-H(23)	120.0

C(23)-C(24)-C(25)	120.8(3)	O(2)-C(26)-H(26B)	109.5
C(23)-C(24)-H(24)	119.6	H(26A)-C(26)-H(26B)	109.5
C(25)-C(24)-H(24)	119.6	O(2)-C(26)-H(26C)	109.5
C(24)-C(25)-C(20)	119.3(3)	H(26A)-C(26)-H(26C)	109.5
C(24)-C(25)-C(4)	118.5(2)	H(26B)-C(26)-H(26C)	109.5
C(20)-C(25)-C(4)	122.0(2)	C(2)-O(1)-C(1)	119.2(3)
O(2)-C(26)-H(26A)	109.5	C(16)-O(2)-C(26)	118.3(3)

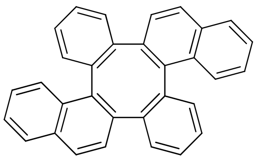

≡

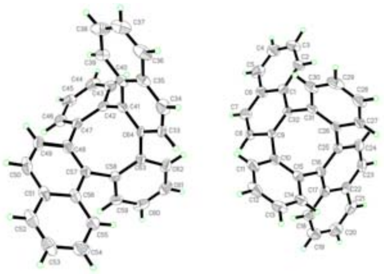

2kk

C(1)-C(2)	1.417(7)	C(16)-C(25)	1.394(6)
C(1)-C(6)	1.435(7)	C(16)-C(17)	1.439(7)
C(1)-C(32)	1.439(6)	C(17)-C(22)	1.410(7)
C(2)-C(3)	1.353(7)	C(17)-C(18)	1.437(7)
C(2)-H(2)	0.9300	C(18)-C(19)	1.371(8)
C(3)-C(4)	1.383(9)	C(18)-H(18)	0.9300
C(3)-H(3)	0.9300	C(19)-C(20)	1.439(9)
C(4)-C(5)	1.343(9)	C(19)-H(19)	0.9300
C(4)-H(4)	0.9300	C(20)-C(21)	1.370(8)
C(5)-C(6)	1.416(7)	C(20)-H(20)	0.9300
C(5)-H(5)	0.9300	C(21)-C(22)	1.416(8)
C(6)-C(7)	1.375(7)	C(21)-H(21)	0.9300
C(7)-C(8)	1.345(7)	C(22)-C(23)	1.386(8)
C(7)-H(7)	0.9300	C(23)-C(24)	1.389(9)
C(8)-C(9)	1.419(6)	C(23)-H(23)	0.9300
C(8)-H(8)	0.9300	C(24)-C(25)	1.406(7)
C(9)-C(32)	1.338(6)	C(24)-H(24)	0.9300
C(9)-C(10)	1.525(6)	C(25)-C(26)	1.457(8)
C(10)-C(15)	1.374(7)	C(26)-C(27)	1.408(7)
C(10)-C(11)	1.377(7)	C(26)-C(31)	1.415(7)
C(11)-C(12)	1.395(8)	C(27)-C(28)	1.395(9)
C(11)-H(11)	0.9300	C(27)-H(27)	0.9300
C(12)-C(13)	1.366(9)	C(28)-C(29)	1.349(9)
C(12)-H(12)	0.9300	C(28)-H(28)	0.9300
C(13)-C(14)	1.370(8)	C(29)-C(30)	1.381(8)
C(13)-H(13)	0.9300	C(29)-H(29)	0.9300
C(14)-C(15)	1.411(6)	C(30)-C(31)	1.389(8)
C(14)-H(14)	0.9300	C(30)-H(30)	0.9300
C(15)-C(16)	1.506(7)	C(31)-C(32)	1.507(7)

C(33)-C(34)	1.315(8)	C(55)-H(55)	0.9300
C(33)-C(64)	1.413(6)	C(56)-C(57)	1.424(6)
C(33)-H(33)	0.9300	C(57)-C(58)	1.512(7)
C(34)-C(35)	1.393(8)	C(58)-C(59)	1.419(7)
C(34)-H(34)	0.9300	C(58)-C(63)	1.407(7)
C(35)-C(40)	1.438(7)	C(59)-C(60)	1.367(8)
C(35)-C(36)	1.430(8)	C(59)-H(59)	0.9300
C(36)-C(37)	1.371(9)	C(60)-C(61)	1.361(9)
C(36)-H(36)	0.9300	C(60)-H(60)	0.9300
C(37)-C(38)	1.401(10)	C(61)-C(62)	1.388(9)
C(37)-H(37)	0.9300	C(61)-H(61)	0.9300
C(38)-C(39)	1.370(8)	C(62)-C(63)	1.378(7)
C(38)-H(38)	0.9300	C(62)-H(62)	0.9300
C(39)-C(40)	1.408(7)	C(63)-C(64)	1.496(7)
C(39)-H(39)	0.9300	C(2)-C(1)-C(6)	116.5(4)
C(40)-C(41)	1.423(7)	C(2)-C(1)-C(32)	124.9(5)
C(41)-C(64)	1.357(6)	C(6)-C(1)-C(32)	118.6(4)
C(41)-C(42)	1.495(6)	C(3)-C(2)-C(1)	122.2(5)
C(42)-C(43)	1.405(6)	C(3)-C(2)-H(2)	118.9
C(42)-C(47)	1.421(6)	C(1)-C(2)-H(2)	118.9
C(43)-C(44)	1.398(7)	C(2)-C(3)-C(4)	121.1(6)
C(43)-H(43)	0.9300	C(2)-C(3)-H(3)	119.5
C(44)-C(45)	1.356(8)	C(4)-C(3)-H(3)	119.5
C(44)-H(44)	0.9300	C(5)-C(4)-C(3)	119.1(6)
C(45)-C(46)	1.395(8)	C(5)-C(4)-H(4)	120.5
C(45)-H(45)	0.9300	C(3)-C(4)-H(4)	120.5
C(46)-C(47)	1.407(7)	C(4)-C(5)-C(6)	122.6(6)
C(46)-H(46)	0.9300	C(4)-C(5)-H(5)	118.7
C(47)-C(48)	1.471(6)	C(6)-C(5)-H(5)	118.7
C(48)-C(57)	1.370(6)	C(7)-C(6)-C(5)	122.8(5)
C(48)-C(49)	1.395(7)	C(7)-C(6)-C(1)	118.9(5)
C(49)-C(50)	1.393(7)	C(5)-C(6)-C(1)	118.3(5)
C(49)-H(49)	0.9300	C(8)-C(7)-C(6)	121.3(5)
C(50)-C(51)	1.413(8)	C(8)-C(7)-H(7)	119.4
C(50)-H(50)	0.9300	C(6)-C(7)-H(7)	119.3
C(51)-C(56)	1.419(7)	C(7)-C(8)-C(9)	121.0(4)
C(51)-C(52)	1.419(7)	C(7)-C(8)-H(8)	119.5
C(52)-C(53)	1.353(9)	C(9)-C(8)-H(8)	119.5
C(52)-H(52)	0.9300	C(32)-C(9)-C(8)	120.4(4)
C(53)-C(54)	1.421(10)	C(32)-C(9)-C(10)	122.1(4)
C(53)-H(53)	0.9300	C(8)-C(9)-C(10)	117.3(4)
C(54)-C(55)	1.395(8)	C(15)-C(10)-C(11)	120.0(4)
C(54)-H(54)	0.9300	C(15)-C(10)-C(9)	121.3(4)
C(55)-C(56)	1.415(7)	C(11)-C(10)-C(9)	118.7(4)

C(12)-C(11)-C(10)	120.2(5)	C(24)-C(25)-C(26)	117.8(4)
C(12)-C(11)-H(11)	119.9	C(27)-C(26)-C(31)	118.1(5)
C(10)-C(11)-H(11)	119.9	C(27)-C(26)-C(25)	118.7(5)
C(13)-C(12)-C(11)	120.4(6)	C(31)-C(26)-C(25)	123.2(4)
C(13)-C(12)-H(12)	119.8	C(28)-C(27)-C(26)	120.5(5)
C(11)-C(12)-H(12)	119.8	C(28)-C(27)-H(27)	119.7
C(12)-C(13)-C(14)	119.5(5)	C(26)-C(27)-H(27)	119.7
C(12)-C(13)-H(13)	120.3	C(29)-C(28)-C(27)	120.0(6)
C(14)-C(13)-H(13)	120.3	C(29)-C(28)-H(28)	120.0
C(13)-C(14)-C(15)	120.9(5)	C(27)-C(28)-H(28)	120.0
C(13)-C(14)-H(14)	119.6	C(28)-C(29)-C(30)	121.4(6)
C(15)-C(14)-H(14)	119.6	C(28)-C(29)-H(29)	119.3
C(10)-C(15)-C(14)	119.0(5)	C(30)-C(29)-H(29)	119.3
C(10)-C(15)-C(16)	122.8(4)	C(29)-C(30)-C(31)	120.1(6)
C(14)-C(15)-C(16)	118.2(4)	C(29)-C(30)-H(30)	119.9
C(25)-C(16)-C(17)	119.4(5)	C(31)-C(30)-H(30)	119.9
C(25)-C(16)-C(15)	120.6(5)	C(30)-C(31)-C(26)	119.7(5)
C(17)-C(16)-C(15)	120.0(4)	C(30)-C(31)-C(32)	119.0(5)
C(22)-C(17)-C(18)	119.5(5)	C(26)-C(31)-C(32)	121.2(5)
C(22)-C(17)-C(16)	119.6(5)	C(9)-C(32)-C(1)	119.7(4)
C(18)-C(17)-C(16)	120.9(5)	C(9)-C(32)-C(31)	122.1(4)
C(19)-C(18)-C(17)	120.1(6)	C(1)-C(32)-C(31)	118.1(4)
C(19)-C(18)-H(18)	119.9	C(34)-C(33)-C(64)	123.3(5)
C(17)-C(18)-H(18)	119.9	C(34)-C(33)-H(33)	118.4
C(18)-C(19)-C(20)	119.6(6)	C(64)-C(33)-H(33)	118.4
C(18)-C(19)-H(19)	120.2	C(33)-C(34)-C(35)	120.6(5)
C(20)-C(19)-H(19)	120.2	C(33)-C(34)-H(34)	119.7
C(21)-C(20)-C(19)	120.9(6)	C(35)-C(34)-H(34)	119.7
C(21)-C(20)-H(20)	119.6	C(34)-C(35)-C(40)	118.6(5)
C(19)-C(20)-H(20)	119.6	C(34)-C(35)-C(36)	123.1(5)
C(20)-C(21)-C(22)	120.1(6)	C(40)-C(35)-C(36)	118.2(5)
C(20)-C(21)-H(21)	119.9	C(37)-C(36)-C(35)	121.7(6)
C(22)-C(21)-H(21)	119.9	C(37)-C(36)-H(36)	119.2
C(23)-C(22)-C(17)	119.1(5)	C(35)-C(36)-H(36)	119.2
C(23)-C(22)-C(21)	121.1(5)	C(36)-C(37)-C(38)	119.8(6)
C(17)-C(22)-C(21)	119.7(5)	C(36)-C(37)-H(37)	120.1
C(24)-C(23)-C(22)	121.9(5)	C(38)-C(37)-H(37)	120.1
C(24)-C(23)-H(23)	119.0	C(39)-C(38)-C(37)	119.8(6)
C(22)-C(23)-H(23)	119.0	C(39)-C(38)-H(38)	120.1
C(23)-C(24)-C(25)	119.7(5)	C(37)-C(38)-H(38)	120.1
C(23)-C(24)-H(24)	120.2	C(38)-C(39)-C(40)	122.9(5)
C(25)-C(24)-H(24)	120.2	C(38)-C(39)-H(39)	118.6
C(16)-C(25)-C(24)	120.2(5)	C(40)-C(39)-H(39)	118.6
C(16)-C(25)-C(26)	121.7(5)	C(39)-C(40)-C(41)	124.1(4)

C(39)-C(40)-C(35)	117.5(5)	C(51)-C(52)-H(52)	120.5
C(41)-C(40)-C(35)	118.4(4)	C(52)-C(53)-C(54)	121.7(6)
C(64)-C(41)-C(40)	120.5(4)	C(52)-C(53)-H(53)	119.2
C(64)-C(41)-C(42)	121.2(4)	C(54)-C(53)-H(53)	119.2
C(40)-C(41)-C(42)	118.3(4)	C(55)-C(54)-C(53)	119.4(6)
C(43)-C(42)-C(47)	118.5(4)	C(55)-C(54)-H(54)	120.3
C(43)-C(42)-C(41)	119.7(4)	C(53)-C(54)-H(54)	120.3
C(47)-C(42)-C(41)	121.6(4)	C(54)-C(55)-C(56)	120.7(6)
C(44)-C(43)-C(42)	121.6(5)	C(54)-C(55)-H(55)	119.7
C(44)-C(43)-H(43)	119.2	C(56)-C(55)-H(55)	119.7
C(42)-C(43)-H(43)	119.2	C(55)-C(56)-C(51)	117.7(5)
C(45)-C(44)-C(43)	118.8(5)	C(55)-C(56)-C(57)	122.9(5)
C(45)-C(44)-H(44)	120.6	C(51)-C(56)-C(57)	119.4(5)
C(43)-C(44)-H(44)	120.6	C(48)-C(57)-C(56)	121.2(5)
C(44)-C(45)-C(46)	122.3(5)	C(48)-C(57)-C(58)	119.9(4)
C(44)-C(45)-H(45)	118.8	C(56)-C(57)-C(58)	118.7(4)
C(46)-C(45)-H(45)	118.8	C(59)-C(58)-C(63)	118.3(5)
C(45)-C(46)-C(47)	119.6(5)	C(59)-C(58)-C(57)	119.9(4)
C(45)-C(46)-H(46)	120.2	C(63)-C(58)-C(57)	121.7(5)
C(47)-C(46)-H(46)	120.2	C(60)-C(59)-C(58)	121.1(5)
C(46)-C(47)-C(42)	119.1(4)	C(60)-C(59)-H(59)	119.4
C(46)-C(47)-C(48)	120.1(4)	C(58)-C(59)-H(59)	119.4
C(42)-C(47)-C(48)	120.7(4)	C(61)-C(60)-C(59)	119.6(6)
C(57)-C(48)-C(49)	119.9(4)	C(61)-C(60)-H(60)	120.2
C(57)-C(48)-C(47)	122.8(5)	C(59)-C(60)-H(60)	120.2
C(49)-C(48)-C(47)	117.2(4)	C(60)-C(61)-C(62)	121.0(6)
C(50)-C(49)-C(48)	120.1(5)	C(60)-C(61)-H(61)	119.5
C(50)-C(49)-H(49)	119.9	C(62)-C(61)-H(61)	119.5
C(48)-C(49)-H(49)	119.9	C(61)-C(62)-C(63)	120.7(5)
C(49)-C(50)-C(51)	121.4(5)	C(61)-C(62)-H(62)	119.6
C(49)-C(50)-H(50)	119.3	C(63)-C(62)-H(62)	119.6
C(51)-C(50)-H(50)	119.3	C(62)-C(63)-C(58)	119.2(5)
C(56)-C(51)-C(52)	121.4(5)	C(62)-C(63)-C(64)	119.8(4)
C(56)-C(51)-C(50)	117.9(5)	C(58)-C(63)-C(64)	121.0(4)
C(52)-C(51)-C(50)	120.7(5)	C(41)-C(64)-C(33)	118.4(4)
C(53)-C(52)-C(51)	119.0(6)	C(41)-C(64)-C(63)	121.3(4)
C(53)-C(52)-H(52)	120.5	C(33)-C(64)-C(63)	120.2(4)

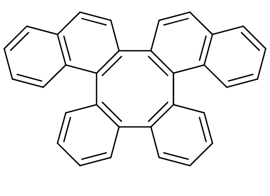 ≡ 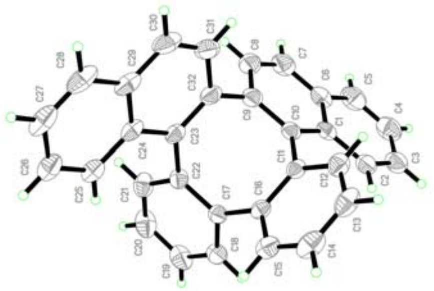

3kk

C(1)-C(2)	1.415(3)	C(20)-C(21)	1.375(3)
C(1)-C(6)	1.423(3)	C(20)-H(20)	0.9300
C(1)-C(10)	1.434(3)	C(21)-C(22)	1.389(3)
C(2)-C(3)	1.369(3)	C(21)-H(21)	0.9300
C(2)-H(2)	0.9300	C(22)-C(23)	1.493(3)
C(3)-C(4)	1.392(3)	C(23)-C(32)	1.377(3)
C(3)-H(3)	0.9300	C(23)-C(24)	1.438(3)
C(4)-C(5)	1.354(4)	C(24)-C(25)	1.411(3)
C(4)-H(4)	0.9300	C(24)-C(29)	1.414(3)
C(5)-C(6)	1.409(3)	C(25)-C(26)	1.366(3)
C(5)-H(5)	0.9300	C(25)-H(25)	0.9300
C(6)-C(7)	1.403(3)	C(26)-C(27)	1.390(3)
C(7)-C(8)	1.352(3)	C(26)-H(26)	0.9300
C(7)-H(7)	0.9300	C(27)-C(28)	1.350(4)
C(8)-C(9)	1.418(3)	C(27)-H(27)	0.9300
C(8)-H(8)	0.9300	C(28)-C(29)	1.419(3)
C(9)-C(10)	1.373(3)	C(28)-H(28)	0.9300
C(9)-C(32)	1.488(3)	C(29)-C(30)	1.406(3)
C(10)-C(11)	1.495(3)	C(30)-C(31)	1.357(3)
C(11)-C(12)	1.390(3)	C(30)-H(30)	0.9300
C(11)-C(16)	1.402(3)	C(31)-C(32)	1.416(3)
C(12)-C(13)	1.377(3)	C(31)-H(31)	0.9300
C(12)-H(12)	0.9300	C(2)-C(1)-C(6)	117.7(2)
C(13)-C(14)	1.370(3)	C(2)-C(1)-C(10)	123.07(19)
C(13)-H(13)	0.9300	C(6)-C(1)-C(10)	119.3(2)
C(14)-C(15)	1.370(3)	C(3)-C(2)-C(1)	121.0(2)
C(14)-H(14)	0.9300	C(3)-C(2)-H(2)	119.5
C(15)-C(16)	1.391(3)	C(1)-C(2)-H(2)	119.5
C(15)-H(15)	0.9300	C(2)-C(3)-C(4)	120.8(2)
C(16)-C(17)	1.486(3)	C(2)-C(3)-H(3)	119.6
C(17)-C(18)	1.390(3)	C(4)-C(3)-H(3)	119.6
C(17)-C(22)	1.396(3)	C(5)-C(4)-C(3)	119.8(2)
C(18)-C(19)	1.375(3)	C(5)-C(4)-H(4)	120.1
C(18)-H(18)	0.9300	C(3)-C(4)-H(4)	120.1
C(19)-C(20)	1.368(3)	C(4)-C(5)-C(6)	121.5(2)
C(19)-H(19)	0.9300	C(4)-C(5)-H(5)	119.3

C(6)-C(5)-H(5)	119.3	C(20)-C(19)-H(19)	120.2
C(7)-C(6)-C(5)	122.0(2)	C(18)-C(19)-H(19)	120.2
C(7)-C(6)-C(1)	118.8(2)	C(19)-C(20)-C(21)	120.2(2)
C(5)-C(6)-C(1)	119.2(2)	C(19)-C(20)-H(20)	119.9
C(8)-C(7)-C(6)	121.2(2)	C(21)-C(20)-H(20)	119.9
C(8)-C(7)-H(7)	119.4	C(20)-C(21)-C(22)	121.1(2)
C(6)-C(7)-H(7)	119.4	C(20)-C(21)-H(21)	119.5
C(7)-C(8)-C(9)	121.0(2)	C(22)-C(21)-H(21)	119.5
C(7)-C(8)-H(8)	119.5	C(21)-C(22)-C(17)	118.75(18)
C(9)-C(8)-H(8)	119.5	C(21)-C(22)-C(23)	120.03(18)
C(10)-C(9)-C(8)	120.0(2)	C(17)-C(22)-C(23)	121.20(16)
C(10)-C(9)-C(32)	121.89(18)	C(32)-C(23)-C(24)	119.98(18)
C(8)-C(9)-C(32)	118.05(19)	C(32)-C(23)-C(22)	120.21(18)
C(9)-C(10)-C(1)	119.62(19)	C(24)-C(23)-C(22)	119.81(18)
C(9)-C(10)-C(11)	120.40(18)	C(25)-C(24)-C(29)	118.0(2)
C(1)-C(10)-C(11)	119.96(17)	C(25)-C(24)-C(23)	123.07(18)
C(12)-C(11)-C(16)	118.73(18)	C(29)-C(24)-C(23)	118.9(2)
C(12)-C(11)-C(10)	119.62(18)	C(26)-C(25)-C(24)	121.3(2)
C(16)-C(11)-C(10)	121.65(16)	C(26)-C(25)-H(25)	119.4
C(13)-C(12)-C(11)	120.9(2)	C(24)-C(25)-H(25)	119.4
C(13)-C(12)-H(12)	119.5	C(25)-C(26)-C(27)	120.6(3)
C(11)-C(12)-H(12)	119.5	C(25)-C(26)-H(26)	119.7
C(14)-C(13)-C(12)	120.1(2)	C(27)-C(26)-H(26)	119.7
C(14)-C(13)-H(13)	119.9	C(28)-C(27)-C(26)	119.9(2)
C(12)-C(13)-H(13)	119.9	C(28)-C(27)-H(27)	120.1
C(13)-C(14)-C(15)	120.1(2)	C(26)-C(27)-H(27)	120.1
C(13)-C(14)-H(14)	120.0	C(27)-C(28)-C(29)	121.6(2)
C(15)-C(14)-H(14)	120.0	C(27)-C(28)-H(28)	119.2
C(14)-C(15)-C(16)	121.0(2)	C(29)-C(28)-H(28)	119.2
C(14)-C(15)-H(15)	119.5	C(30)-C(29)-C(24)	119.2(2)
C(16)-C(15)-H(15)	119.5	C(30)-C(29)-C(28)	122.2(2)
C(15)-C(16)-C(11)	119.17(18)	C(24)-C(29)-C(28)	118.6(2)
C(15)-C(16)-C(17)	119.74(18)	C(31)-C(30)-C(29)	121.3(2)
C(11)-C(16)-C(17)	121.07(16)	C(31)-C(30)-H(30)	119.3
C(18)-C(17)-C(22)	119.05(18)	C(29)-C(30)-H(30)	119.3
C(18)-C(17)-C(16)	119.66(17)	C(30)-C(31)-C(32)	120.6(2)
C(22)-C(17)-C(16)	121.29(16)	C(30)-C(31)-H(31)	119.7
C(19)-C(18)-C(17)	121.2(2)	C(32)-C(31)-H(31)	119.7
C(19)-C(18)-H(18)	119.4	C(23)-C(32)-C(31)	119.9(2)
C(17)-C(18)-H(18)	119.4	C(23)-C(32)-C(9)	121.92(18)
C(20)-C(19)-C(18)	119.7(2)	C(31)-C(32)-C(9)	118.1(2)

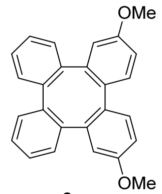

2ae

≡

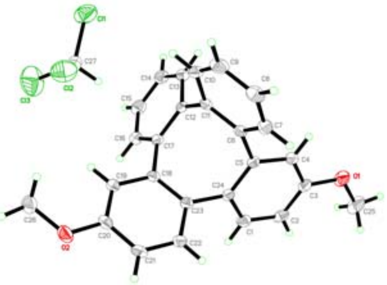

C(1)-C(24)	1.392(4)	C(19)-H(19)	0.9300
C(1)-C(2)	1.393(5)	C(20)-O(2)	1.365(4)
C(1)-H(1)	0.9300	C(20)-C(21)	1.393(5)
C(2)-C(3)	1.374(5)	C(21)-C(22)	1.378(4)
C(2)-H(2)	0.9300	C(21)-H(21)	0.9300
C(3)-O(1)	1.376(4)	C(22)-C(23)	1.410(4)
C(3)-C(4)	1.393(4)	C(22)-H(22)	0.9300
C(4)-C(5)	1.386(4)	C(23)-C(24)	1.489(4)
C(4)-H(4)	0.9300	C(25)-O(1)	1.421(5)
C(5)-C(24)	1.407(4)	C(25)-H(25A)	0.9600
C(5)-C(6)	1.493(4)	C(25)-H(25B)	0.9600
C(6)-C(7)	1.393(4)	C(25)-H(25C)	0.9600
C(6)-C(11)	1.408(4)	C(26)-O(2)	1.429(5)
C(7)-C(8)	1.381(5)	C(26)-H(26A)	0.9600
C(7)-H(7)	0.9300	C(26)-H(26B)	0.9600
C(8)-C(9)	1.379(5)	C(26)-H(26C)	0.9600
C(8)-H(8)	0.9300	C(27)-Cl(3)	1.729(4)
C(9)-C(10)	1.382(5)	C(27)-Cl(1)	1.741(4)
C(9)-H(9)	0.9300	C(27)-Cl(2)	1.749(4)
C(10)-C(11)	1.396(4)	C(27)-H(27)	0.9800
C(10)-H(10)	0.9300	C(24)-C(1)-C(2)	122.5(3)
C(11)-C(12)	1.487(4)	C(24)-C(1)-H(1)	118.7
C(12)-C(17)	1.402(4)	C(2)-C(1)-H(1)	118.7
C(12)-C(13)	1.404(4)	C(3)-C(2)-C(1)	119.1(3)
C(13)-C(14)	1.370(5)	C(3)-C(2)-H(2)	120.4
C(13)-H(13)	0.9300	C(1)-C(2)-H(2)	120.4
C(14)-C(15)	1.377(5)	C(2)-C(3)-O(1)	125.0(3)
C(14)-H(14)	0.9300	C(2)-C(3)-C(4)	119.5(3)
C(15)-C(16)	1.390(5)	O(1)-C(3)-C(4)	115.4(3)
C(15)-H(15)	0.9300	C(5)-C(4)-C(3)	121.5(3)
C(16)-C(17)	1.391(4)	C(5)-C(4)-H(4)	119.3
C(16)-H(16)	0.9300	C(3)-C(4)-H(4)	119.3
C(17)-C(18)	1.493(4)	C(4)-C(5)-C(24)	119.7(3)
C(18)-C(23)	1.394(4)	C(4)-C(5)-C(6)	118.4(2)
C(18)-C(19)	1.404(4)	C(24)-C(5)-C(6)	121.8(2)
C(19)-C(20)	1.381(4)	C(7)-C(6)-C(11)	119.1(3)

C(7)-C(6)-C(5)	118.9(3)	C(20)-C(19)-C(18)	120.4(3)
C(11)-C(6)-C(5)	121.9(2)	C(20)-C(19)-H(19)	119.8
C(8)-C(7)-C(6)	121.2(3)	C(18)-C(19)-H(19)	119.8
C(8)-C(7)-H(7)	119.4	O(2)-C(20)-C(19)	124.7(3)
C(6)-C(7)-H(7)	119.4	O(2)-C(20)-C(21)	115.5(3)
C(9)-C(8)-C(7)	119.9(3)	C(19)-C(20)-C(21)	119.8(3)
C(9)-C(8)-H(8)	120.1	C(22)-C(21)-C(20)	119.9(3)
C(7)-C(8)-H(8)	120.1	C(22)-C(21)-H(21)	120.0
C(8)-C(9)-C(10)	119.8(3)	C(20)-C(21)-H(21)	120.0
C(8)-C(9)-H(9)	120.1	C(21)-C(22)-C(23)	121.3(3)
C(10)-C(9)-H(9)	120.1	C(21)-C(22)-H(22)	119.4
C(9)-C(10)-C(11)	121.3(3)	C(23)-C(22)-H(22)	119.4
C(9)-C(10)-H(10)	119.3	C(18)-C(23)-C(22)	118.2(3)
C(11)-C(10)-H(10)	119.3	C(18)-C(23)-C(24)	123.0(3)
C(10)-C(11)-C(6)	118.6(3)	C(22)-C(23)-C(24)	118.8(3)
C(10)-C(11)-C(12)	119.0(3)	C(1)-C(24)-C(5)	117.7(3)
C(6)-C(11)-C(12)	122.4(2)	C(1)-C(24)-C(23)	120.2(3)
C(17)-C(12)-C(13)	118.7(3)	C(5)-C(24)-C(23)	122.0(3)
C(17)-C(12)-C(11)	121.4(3)	O(1)-C(25)-H(25A)	109.5
C(13)-C(12)-C(11)	119.7(3)	O(1)-C(25)-H(25B)	109.5
C(14)-C(13)-C(12)	121.5(3)	H(25A)-C(25)-H(25B)	109.5
C(14)-C(13)-H(13)	119.3	O(1)-C(25)-H(25C)	109.5
C(12)-C(13)-H(13)	119.3	H(25A)-C(25)-H(25C)	109.5
C(13)-C(14)-C(15)	120.1(3)	H(25B)-C(25)-H(25C)	109.5
C(13)-C(14)-H(14)	119.9	O(2)-C(26)-H(26A)	109.5
C(15)-C(14)-H(14)	119.9	O(2)-C(26)-H(26B)	109.5
C(14)-C(15)-C(16)	119.3(3)	H(26A)-C(26)-H(26B)	109.5
C(14)-C(15)-H(15)	120.3	O(2)-C(26)-H(26C)	109.5
C(16)-C(15)-H(15)	120.3	H(26A)-C(26)-H(26C)	109.5
C(15)-C(16)-C(17)	121.5(3)	H(26B)-C(26)-H(26C)	109.5
C(15)-C(16)-H(16)	119.2	Cl(3)-C(27)-Cl(1)	112.9(2)
C(17)-C(16)-H(16)	119.2	Cl(3)-C(27)-Cl(2)	109.5(2)
C(16)-C(17)-C(12)	118.8(3)	Cl(1)-C(27)-Cl(2)	109.0(2)
C(16)-C(17)-C(18)	119.4(2)	Cl(3)-C(27)-H(27)	108.4
C(12)-C(17)-C(18)	121.8(2)	Cl(1)-C(27)-H(27)	108.4
C(23)-C(18)-C(19)	120.2(3)	Cl(2)-C(27)-H(27)	108.4
C(23)-C(18)-C(17)	122.1(2)	C(3)-O(1)-C(25)	118.0(3)
C(19)-C(18)-C(17)	117.6(2)	C(20)-O(2)-C(26)	118.0(3)

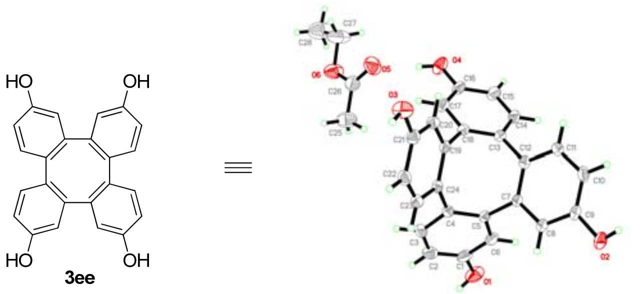

C(1)-C(2)	1.373(4)	C(20)-C(21)	1.383(3)
C(1)-O(1)	1.374(3)	C(20)-H(20)	0.9300
C(1)-C(6)	1.384(3)	C(21)-O(3)	1.356(3)
C(2)-C(3)	1.381(4)	C(21)-C(22)	1.382(3)
C(2)-H(2)	0.9300	C(22)-C(23)	1.372(3)
C(3)-C(4)	1.392(3)	C(22)-H(22)	0.9300
C(3)-H(3)	0.9300	C(23)-C(24)	1.395(3)
C(4)-C(5)	1.398(3)	C(23)-H(23)	0.9300
C(4)-C(24)	1.485(3)	C(25)-C(26)	1.472(4)
C(5)-C(6)	1.384(3)	C(25)-H(25A)	0.9600
C(5)-C(7)	1.497(3)	C(25)-H(25B)	0.9600
C(6)-H(6)	0.9300	C(25)-H(25C)	0.9600
C(7)-C(8)	1.384(3)	C(26)-O(5)	1.198(3)
C(7)-C(12)	1.396(3)	C(26)-O(6)	1.310(4)
C(8)-C(9)	1.377(3)	C(27)-C(28)	1.440(5)
C(8)-H(8)	0.9300	C(27)-O(6)	1.457(4)
C(9)-C(10)	1.369(3)	C(27)-H(27A)	0.9700
C(9)-O(2)	1.377(3)	C(27)-H(27B)	0.9700
C(10)-C(11)	1.380(3)	C(28)-H(28A)	0.9600
C(10)-H(10)	0.9300	C(28)-H(28B)	0.9600
C(11)-C(12)	1.389(3)	C(28)-H(28C)	0.9600
C(11)-H(11)	0.9300	O(1)-H(1)	0.8200
C(12)-C(13)	1.488(3)	O(2)-H(2A)	0.8200
C(13)-C(14)	1.388(3)	O(3)-H(3A)	0.8200
C(13)-C(18)	1.396(3)	O(4)-H(4)	0.8200
C(14)-C(15)	1.380(3)	C(2)-C(1)-O(1)	124.0(2)
C(14)-H(14)	0.9300	C(2)-C(1)-C(6)	119.7(2)
C(15)-C(16)	1.375(3)	O(1)-C(1)-C(6)	116.3(3)
C(15)-H(15)	0.9300	C(1)-C(2)-C(3)	119.8(2)
C(16)-C(17)	1.375(3)	C(1)-C(2)-H(2)	120.1
C(16)-O(4)	1.378(3)	C(3)-C(2)-H(2)	120.1
C(17)-C(18)	1.390(3)	C(2)-C(3)-C(4)	121.5(2)
C(17)-H(17)	0.9300	C(2)-C(3)-H(3)	119.2
C(18)-C(19)	1.494(3)	C(4)-C(3)-H(3)	119.2
C(19)-C(20)	1.381(3)	C(3)-C(4)-C(5)	118.2(2)
C(19)-C(24)	1.403(3)	C(3)-C(4)-C(24)	119.9(2)

C(5)-C(4)-C(24)	121.84(19)	C(20)-C(19)-C(18)	117.56(18)
C(6)-C(5)-C(4)	119.8(2)	C(24)-C(19)-C(18)	122.33(19)
C(6)-C(5)-C(7)	117.8(2)	C(19)-C(20)-C(21)	121.2(2)
C(4)-C(5)-C(7)	122.2(2)	C(19)-C(20)-H(20)	119.4
C(1)-C(6)-C(5)	120.9(2)	C(21)-C(20)-H(20)	119.4
C(1)-C(6)-H(6)	119.5	O(3)-C(21)-C(22)	122.9(2)
C(5)-C(6)-H(6)	119.5	O(3)-C(21)-C(20)	117.7(2)
C(8)-C(7)-C(12)	119.99(19)	C(22)-C(21)-C(20)	119.4(2)
C(8)-C(7)-C(5)	117.26(18)	C(23)-C(22)-C(21)	119.4(2)
C(12)-C(7)-C(5)	122.67(18)	C(23)-C(22)-H(22)	120.3
C(9)-C(8)-C(7)	120.8(2)	C(21)-C(22)-H(22)	120.3
C(9)-C(8)-H(8)	119.6	C(22)-C(23)-C(24)	122.6(2)
C(7)-C(8)-H(8)	119.6	C(22)-C(23)-H(23)	118.7
C(10)-C(9)-O(2)	123.1(2)	C(24)-C(23)-H(23)	118.7
C(10)-C(9)-C(8)	120.2(2)	C(23)-C(24)-C(19)	117.3(2)
O(2)-C(9)-C(8)	116.7(2)	C(23)-C(24)-C(4)	120.14(19)
C(9)-C(10)-C(11)	119.1(2)	C(19)-C(24)-C(4)	122.53(19)
C(9)-C(10)-H(10)	120.4	C(26)-C(25)-H(25A)	109.5
C(11)-C(10)-H(10)	120.4	C(26)-C(25)-H(25B)	109.5
C(10)-C(11)-C(12)	122.2(2)	H(25A)-C(25)-H(25B)	109.5
C(10)-C(11)-H(11)	118.9	C(26)-C(25)-H(25C)	109.5
C(12)-C(11)-H(11)	118.9	H(25A)-C(25)-H(25C)	109.5
C(11)-C(12)-C(7)	117.65(19)	H(25B)-C(25)-H(25C)	109.5
C(11)-C(12)-C(13)	120.45(19)	O(5)-C(26)-O(6)	121.5(3)
C(7)-C(12)-C(13)	121.85(18)	O(5)-C(26)-C(25)	125.3(3)
C(14)-C(13)-C(18)	118.26(19)	O(6)-C(26)-C(25)	113.1(3)
C(14)-C(13)-C(12)	119.68(19)	C(28)-C(27)-O(6)	109.3(3)
C(18)-C(13)-C(12)	122.04(19)	C(28)-C(27)-H(27A)	109.8
C(15)-C(14)-C(13)	121.8(2)	O(6)-C(27)-H(27A)	109.8
C(15)-C(14)-H(14)	119.1	C(28)-C(27)-H(27B)	109.8
C(13)-C(14)-H(14)	119.1	O(6)-C(27)-H(27B)	109.8
C(16)-C(15)-C(14)	119.3(2)	H(27A)-C(27)-H(27B)	108.3
C(16)-C(15)-H(15)	120.4	C(27)-C(28)-H(28A)	109.5
C(14)-C(15)-H(15)	120.4	C(27)-C(28)-H(28B)	109.5
C(15)-C(16)-C(17)	120.3(2)	H(28A)-C(28)-H(28B)	109.5
C(15)-C(16)-O(4)	118.3(2)	C(27)-C(28)-H(28C)	109.5
C(17)-C(16)-O(4)	121.5(2)	H(28A)-C(28)-H(28C)	109.5
C(16)-C(17)-C(18)	120.7(2)	H(28B)-C(28)-H(28C)	109.5
C(16)-C(17)-H(17)	119.7	C(1)-O(1)-H(1)	109.5
C(18)-C(17)-H(17)	119.7	C(9)-O(2)-H(2A)	109.5
C(17)-C(18)-C(13)	119.7(2)	C(21)-O(3)-H(3A)	109.5
C(17)-C(18)-C(19)	117.77(18)	C(16)-O(4)-H(4)	109.5
C(13)-C(18)-C(19)	122.23(18)	C(26)-O(6)-C(27)	117.5(2)
C(20)-C(19)-C(24)	120.1(2)		

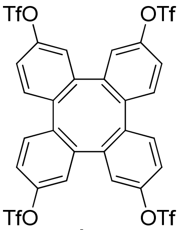

4ee

≡

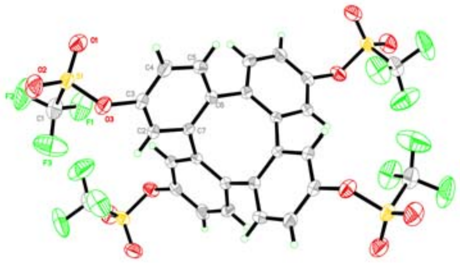

C(1)-F(3)	1.278(10)	C(3)-C(2)-C(7)	119.7(5)
C(1)-F(1)	1.307(10)	C(3)-C(2)-H(2)	120.2
C(1)-F(2)	1.308(9)	C(7)-C(2)-H(2)	120.2
C(1)-S(1)	1.792(9)	C(4)-C(3)-C(2)	123.1(6)
C(2)-C(3)	1.365(8)	C(4)-C(3)-O(3)	120.4(5)
C(2)-C(7)	1.394(8)	C(2)-C(3)-O(3)	116.5(5)
C(2)-H(2)	0.9300	C(3)-C(4)-C(5)	118.1(6)
C(3)-C(4)	1.354(9)	C(3)-C(4)-H(4)	121.0
C(3)-O(3)	1.428(7)	C(5)-C(4)-H(4)	121.0
C(4)-C(5)	1.375(9)	C(4)-C(5)-C(6)	121.1(5)
C(4)-H(4)	0.9300	C(4)-C(5)-H(5)	119.4
C(5)-C(6)	1.398(8)	C(6)-C(5)-H(5)	119.4
C(5)-H(5)	0.9300	C(7)-C(6)-C(5)	119.5(5)
C(6)-C(7)	1.395(7)	C(7)-C(6)-C(6)#1	121.5(5)
C(6)-C(6)#1	1.486(12)	C(5)-C(6)-C(6)#1	118.8(4)
C(7)-C(7)#2	1.472(10)	C(2)-C(7)-C(6)	118.5(5)
O(3)-S(1)	1.558(5)	C(2)-C(7)-C(7)#2	117.9(3)
O(1)-S(1)	1.389(5)	C(6)-C(7)-C(7)#2	123.2(5)
O(2)-S(1)	1.400(5)	C(3)-O(3)-S(1)	120.7(4)
F(3)-C(1)-F(1)	105.5(8)	O(1)-S(1)-O(2)	122.2(3)
F(3)-C(1)-F(2)	111.3(9)	O(1)-S(1)-O(3)	112.3(3)
F(1)-C(1)-F(2)	105.6(8)	O(2)-S(1)-O(3)	105.2(3)
F(3)-C(1)-S(1)	113.7(6)	O(1)-S(1)-C(1)	107.3(4)
F(1)-C(1)-S(1)	112.0(6)	O(2)-S(1)-C(1)	106.1(4)
F(2)-C(1)-S(1)	108.6(6)	O(3)-S(1)-C(1)	101.7(3)

Symmetry transformations used to generate equivalent atoms:

#1 -x+1,-y+1,z #2 -x+1,y,-z

X-ray Crystallographic Analysis.

The structures of **2aa**, **2bb**, **2dd**, **2ee**, **2hh**, **2ii**, **3jj**, **2kk**, **3kk**, **3ee** and **4ee** were determined by X-ray crystallographic analyses, and the structural data from X-ray analysis are summarized in Table S1. All the compounds show the saddle-shaped structures wherein the central cyclooctatetraene (COT) parties display the tub

conformation. The bent angles of these compounds are from 49.31° to 50.81°, which indicate that the tube conformations are similar. Not only the bent angles but also the average inner angles of the central eight-membered rings are identical. The values of the average inner angles are from 121.2° to 123.1°, which are obviously different from the regular octagon (135.0 °). As for the bond lengths of the COT rings of the compounds, the average values of bond A are from 1.384(6) Å to 1.406 (5) Å, and the B are from 1.476(6) Å to 1.497(4) Å, which are show that there are no significant differences each other in these compounds. The bond lengths A are similar with double bonds, while the B is tend to single bond. These results explain that COT structures are not planar configuration and not conjugated.

Table S1|Selected bond lengths (Å) of a series of tetraphenylene derivatives

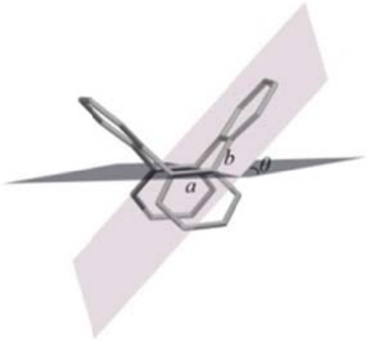

compound	Bond (Å)		Bent angle (°C)	Average inner angle (°C)
	a	b	θ	
**2aa**	1.406(5)	1.492(6)	49.75	122.8
**2bb**	1.403(6)	1.476(6)	49.40	123.0
**2dd**	1.386(5)	1.483(5)	49.31	123.1
**2ee**	1.397(3)	1.491(3)	50.32	122.5
**2hh**	1.403(3)	1.488(3)	50.77	122.3

**2ii**	1.391(3)	1.484(4)	50.81	122.2
**3jj**	1.401(4)	1.497(4)	50.31	122.5
**2kk**	1.387(3)	1.491(3)	52.7	121.2
**3kk**	1.384(6)	1.496(7)	52.00	121.6
**3ee**	1.399(3)	1.491(3)	50.90	122.2
**4ee**	1.395(7)	1.478 (10)	50.64	122.4

[a]thermal ellipsoid at 30% probability level.

10. ^{1}H, ^{13}C and ^{19}F NMR Spectra of Substrates and Products

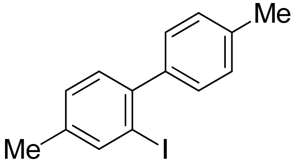

1b

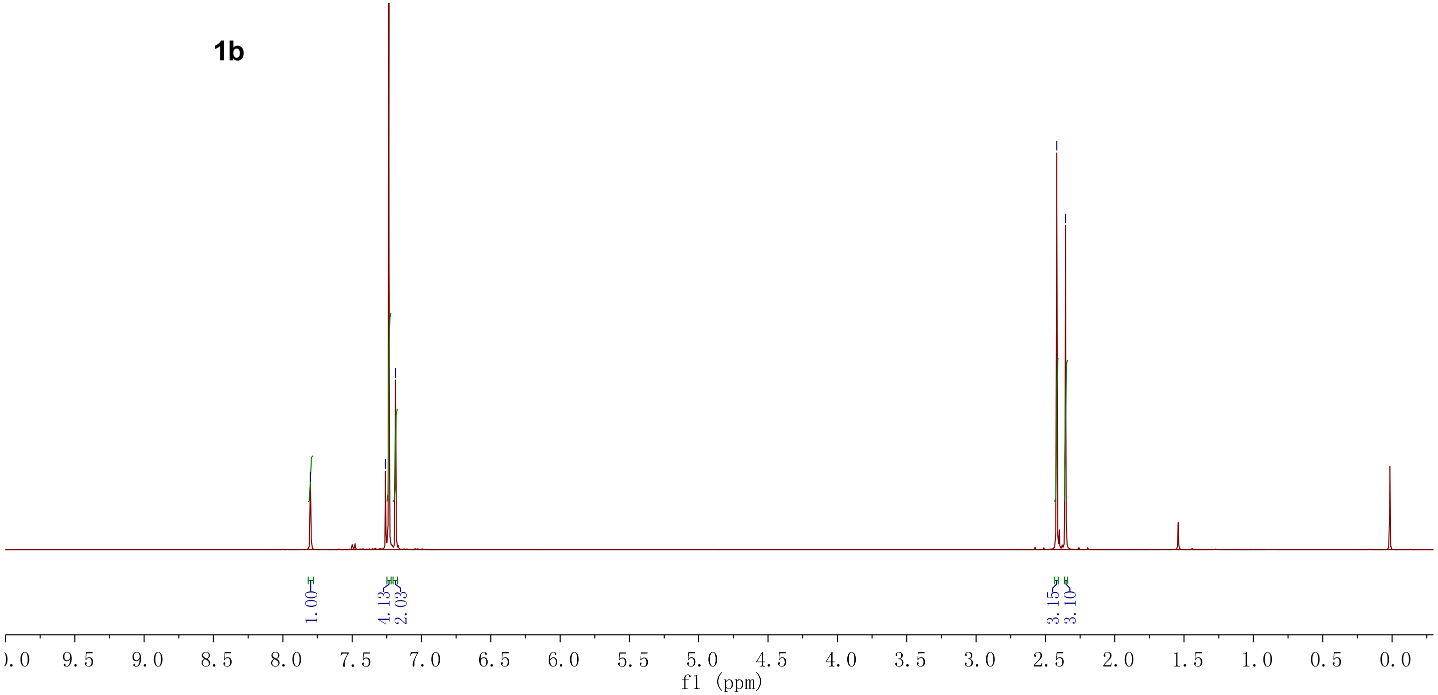

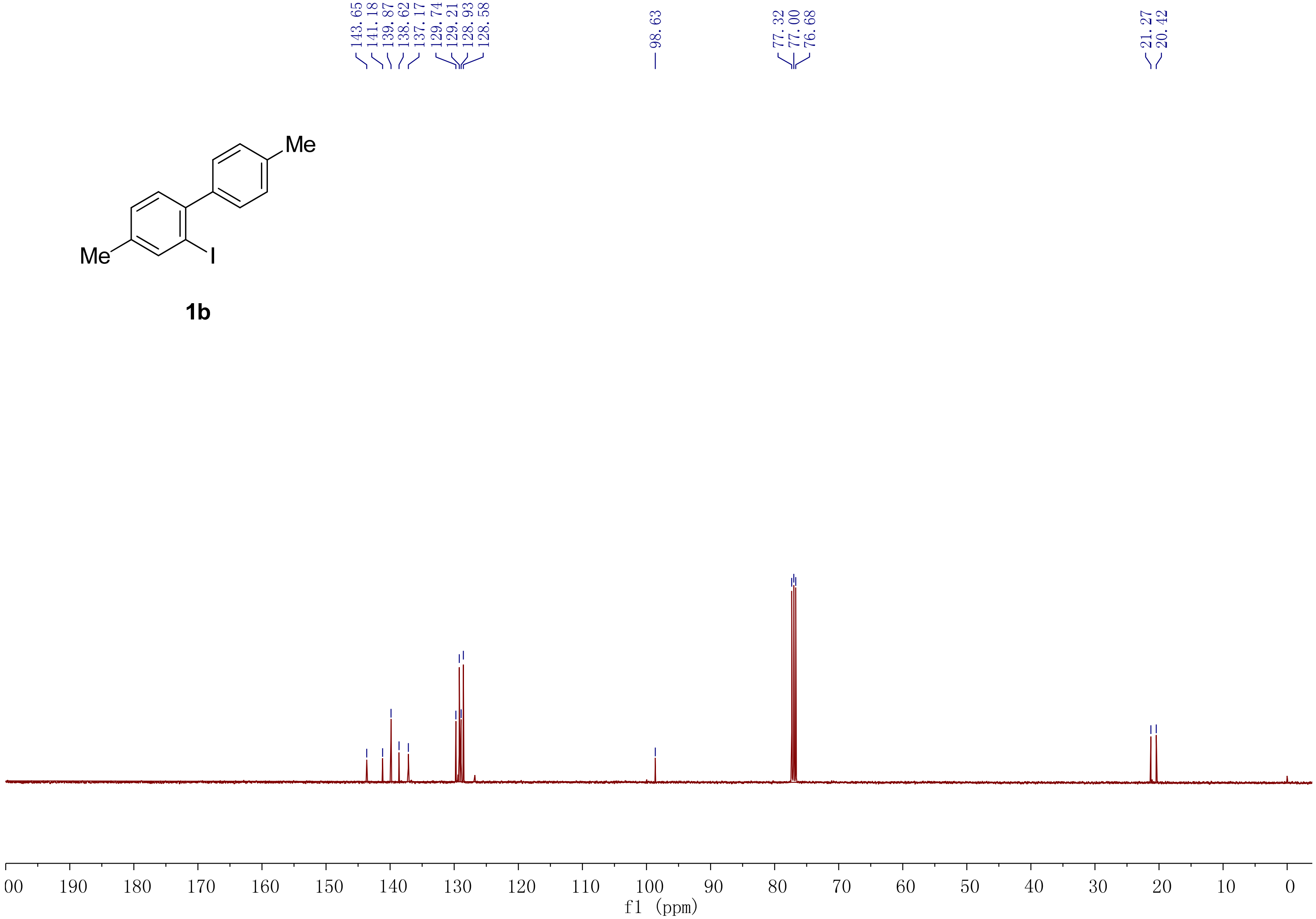
143.65
141.18
139.87
138.62
137.17
129.74
129.21
128.93
128.58
98.63
77.32
77.00
76.68
21.27
20.42
Me
Me
I
1b
00 190 180 170 160 150 140 130 120 110 100 90 80 70 60 50 40 30 20 10 0
f1 (ppm)

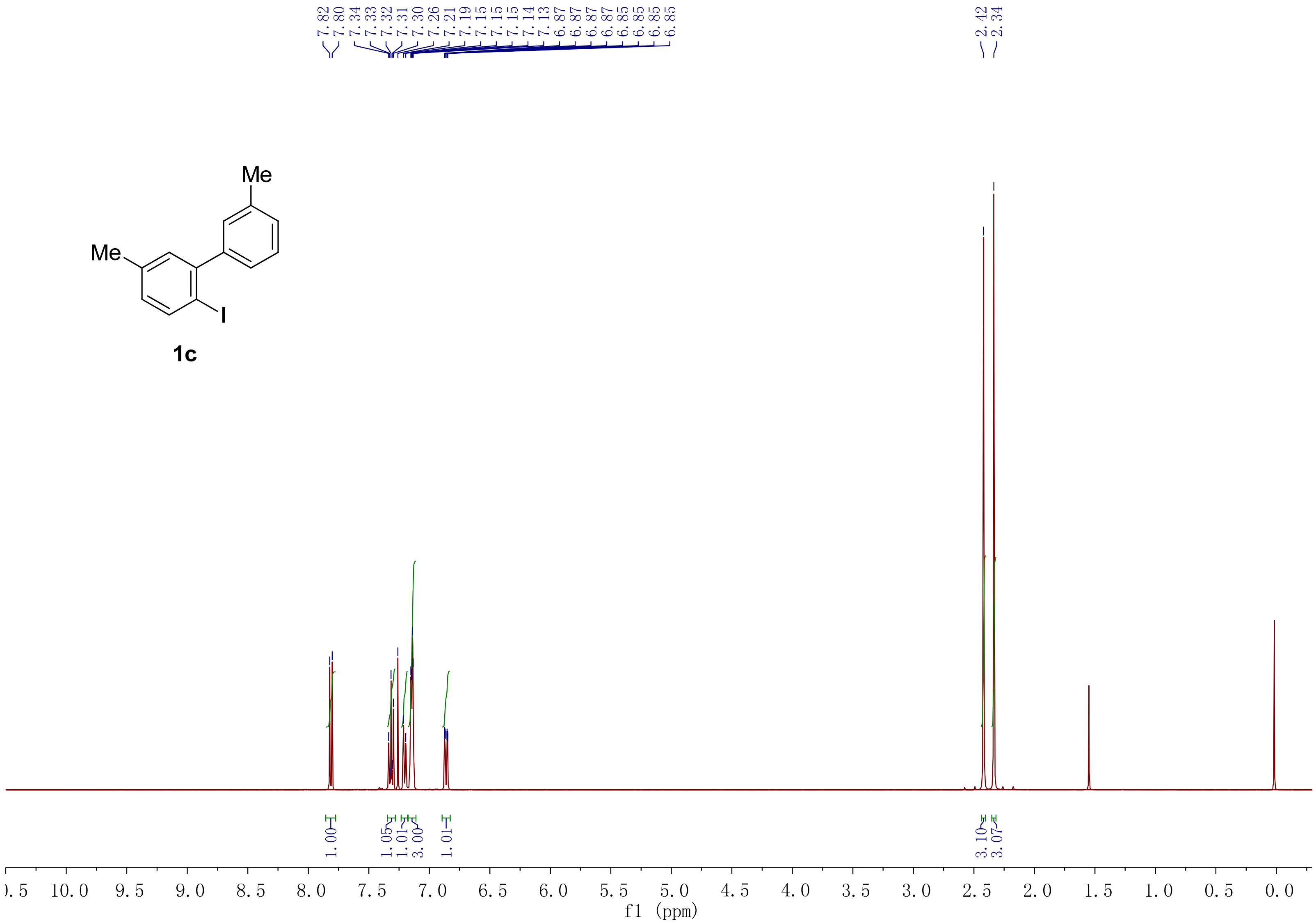
7.82
7.80
7.34
7.33
7.32
7.31
7.30
7.26
7.21
7.19
7.15
7.15
7.15
7.14
7.13
6.87
6.87
6.87
6.85
6.85
6.85
2.42
2.34
Me
Me
I
1c
1.00
1.05
1.01
3.00
1.01
3.10
3.07
10.0
9.5
9.0
8.5
8.0
7.5
7.0
6.5
6.0
5.5
5.0
4.5
4.0
3.5
3.0
2.5
2.0
1.5
1.0
0.5
0.0
f1 (ppm)

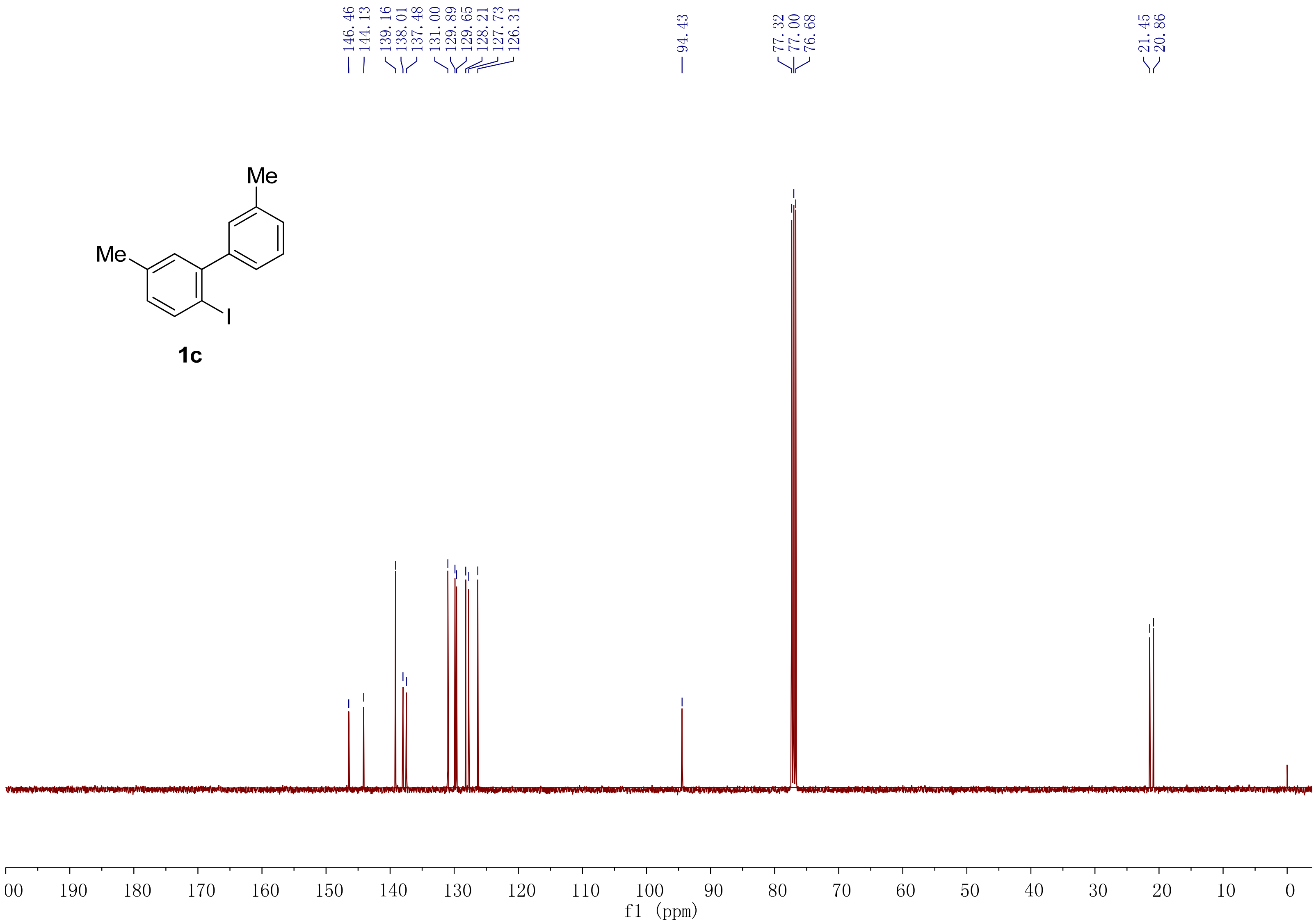
146.46
144.13
139.16
138.01
137.48
131.00
129.89
129.65
128.21
127.73
126.31
94.43
77.32
77.00
76.68
21.45
20.86
Me
Me
I
1c
00
190
180
170
160
150
140
130
120
110
100
90
80
70
60
50
40
30
20
10
0
f1 (ppm)

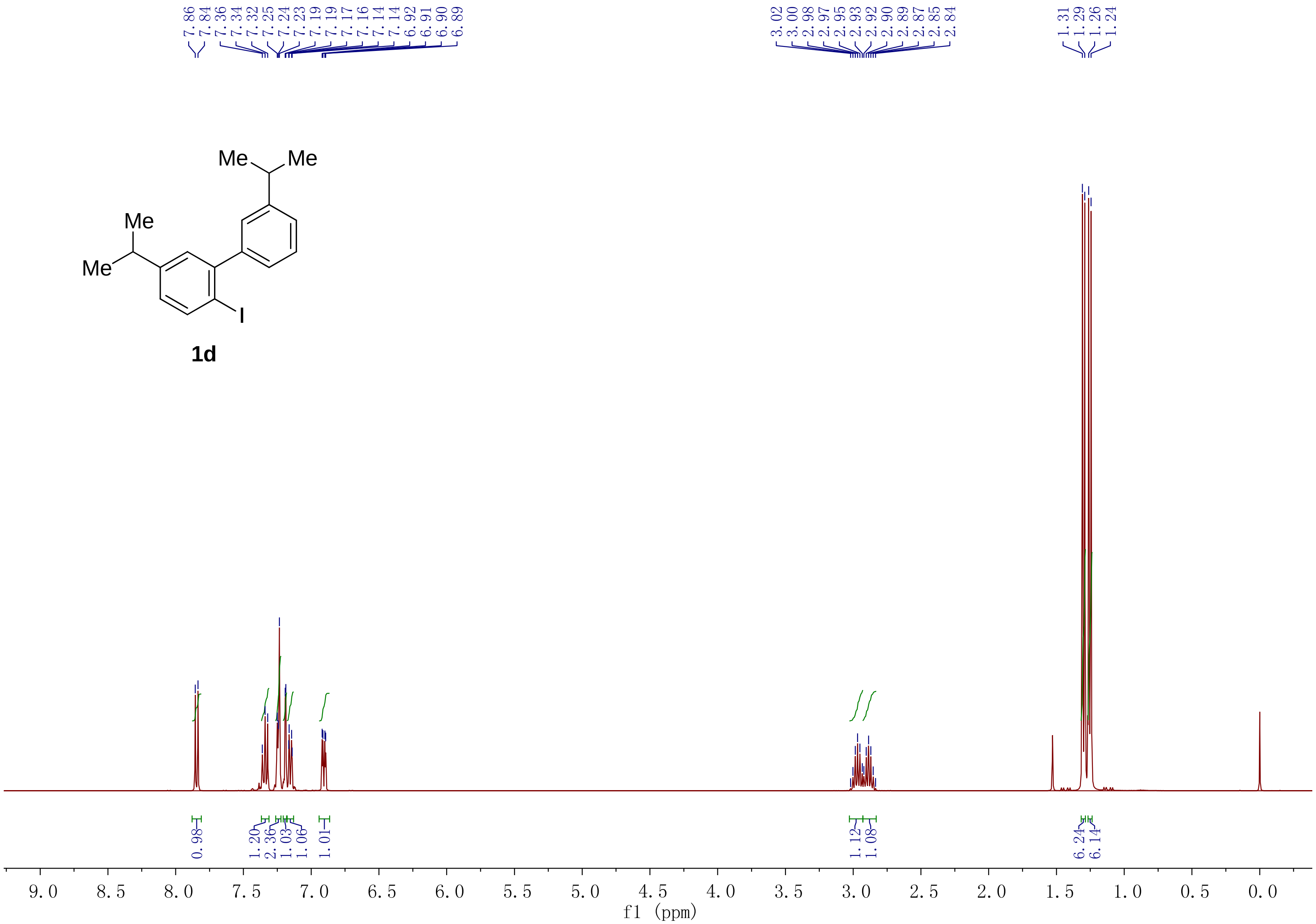
7.86
7.84
7.36
7.34
7.32
7.25
7.24
7.23
7.19
7.19
7.17
7.16
7.14
7.14
6.92
6.91
6.90
6.89
3.02
3.00
2.98
2.97
2.95
2.93
2.92
2.90
2.89
2.87
2.85
2.84
1.31
1.29
1.26
1.24
Me
Me
Me
Me
I
1d
0.98
1.20
2.36
1.03
1.06
1.01
1.12
1.08
6.24
6.14
9.0
8.5
8.0
7.5
7.0
6.5
6.0
5.5
5.0
4.5
4.0
3.5
3.0
2.5
2.0
1.5
1.0
0.5
0.0
f1 (ppm)

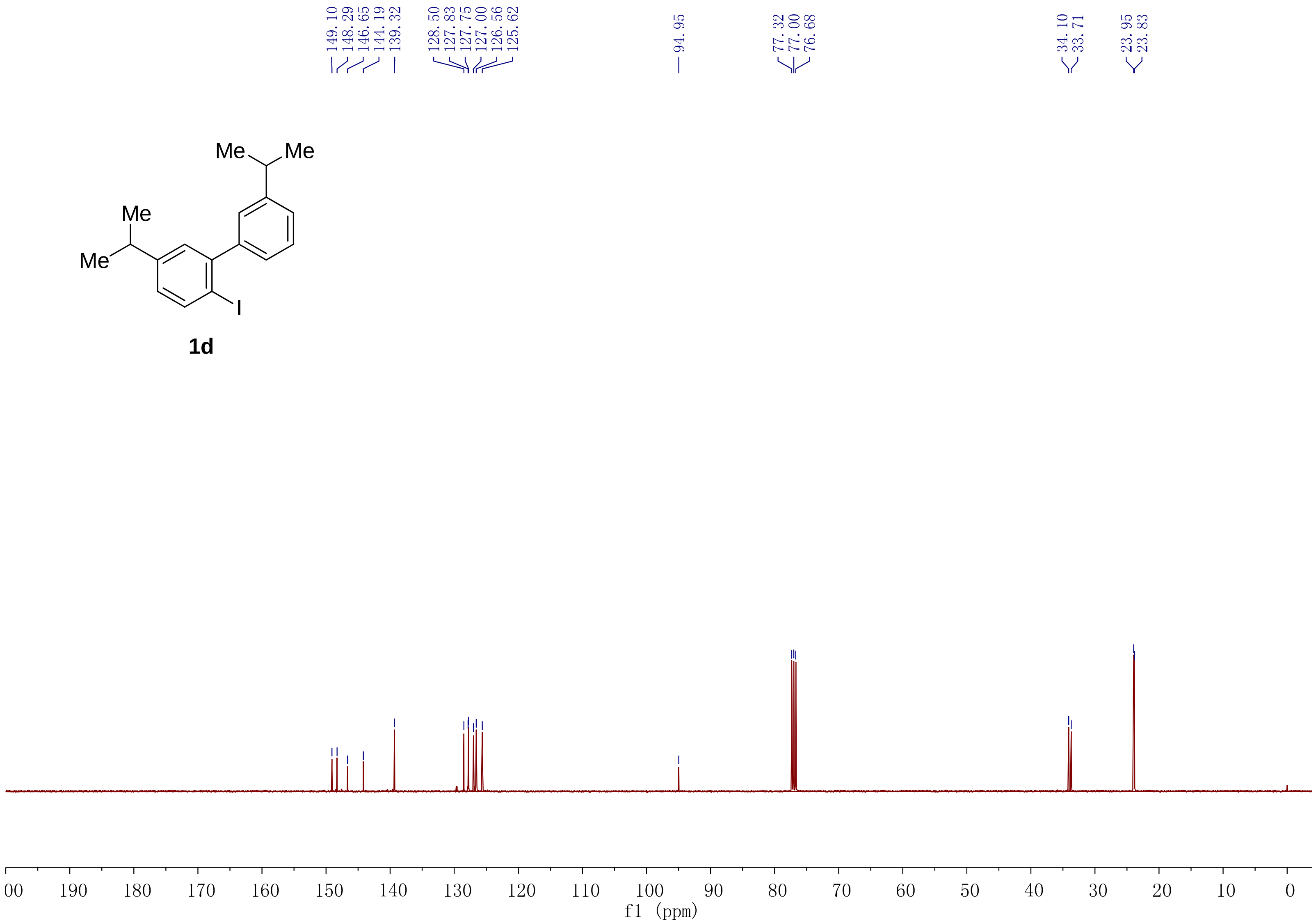
Me
Me
Me
Me
I
149.10
148.29
146.65
144.19
139.32
128.50
127.83
127.75
127.00
126.56
125.62
94.95
77.32
77.00
76.68
34.10
33.71
23.95
23.83
f1 (ppm)

1d

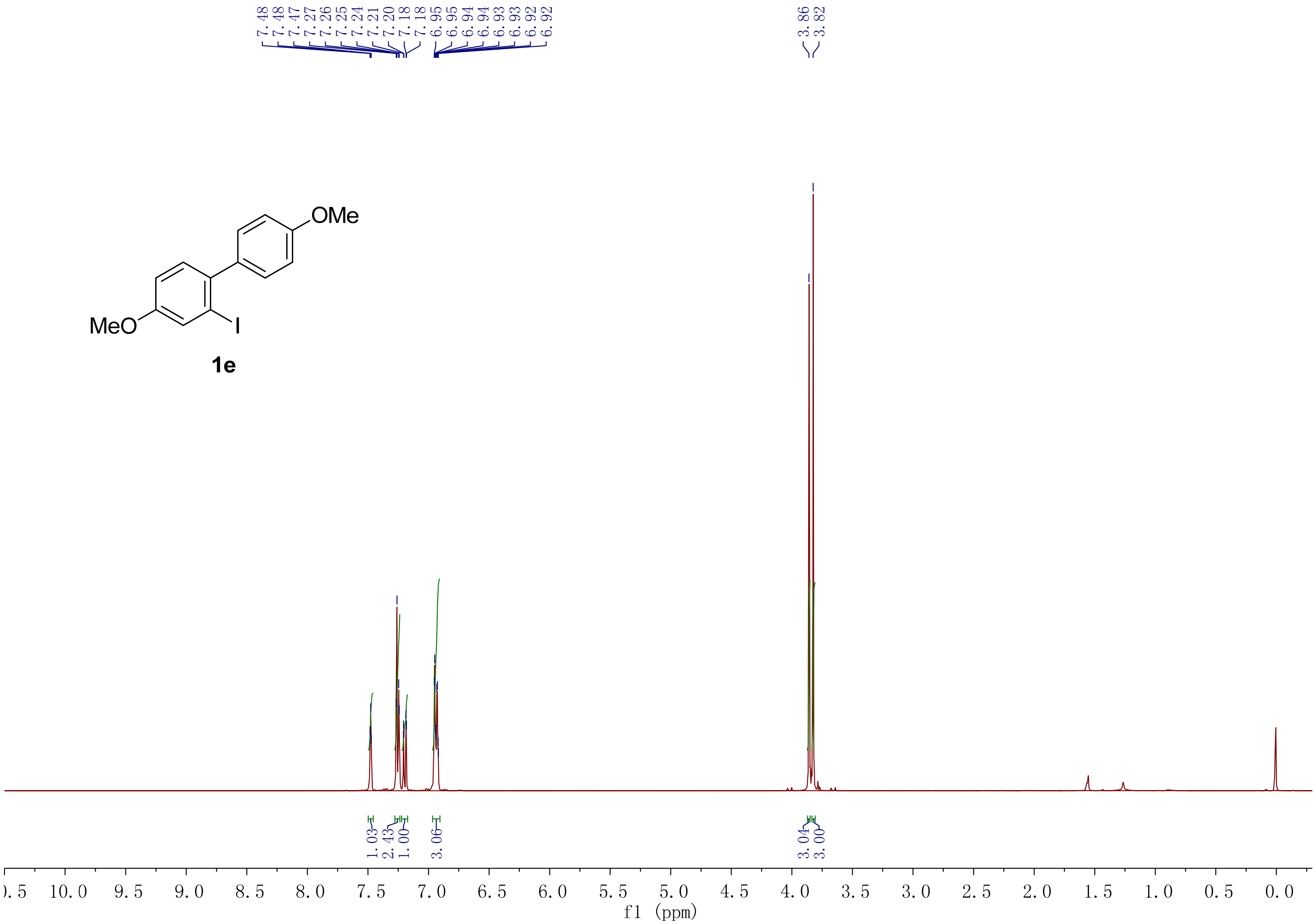
7.48
7.48
7.47
7.27
7.26
7.25
7.24
7.21
7.20
7.18
7.18
6.95
6.95
6.94
6.94
6.93
6.93
6.92
6.92
3.86
3.82
OMe
MeO
I
1e
1.03
2.43
1.00
3.06
3.04
3.00
f1 (ppm)

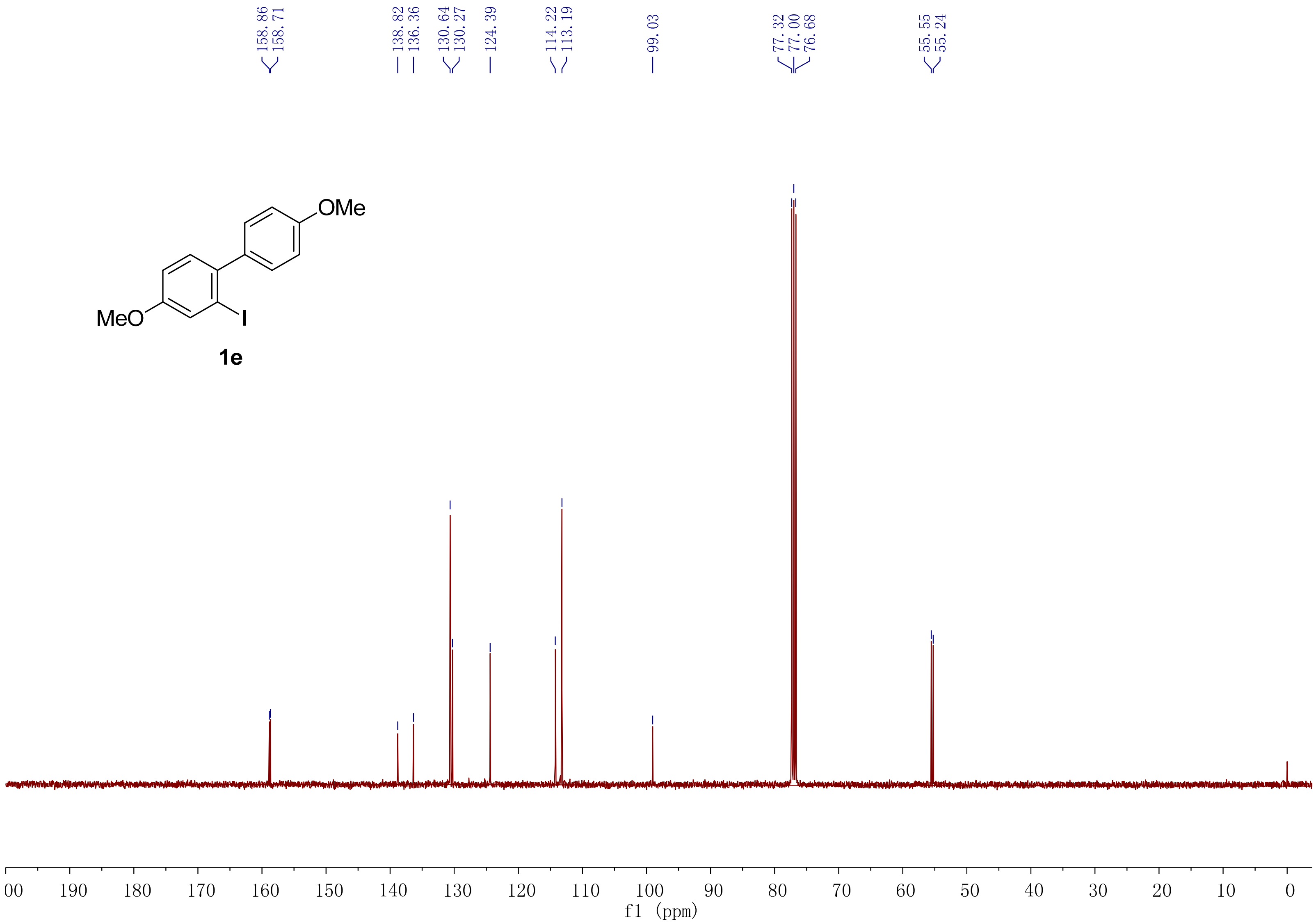
158.86
158.71
138.82
136.36
130.64
130.27
124.39
114.22
113.19
99.03
77.32
77.00
76.68
55.55
55.24
OMe
MeO
I
1e
00 190 180 170 160 150 140 130 120 110 100 90 80 70 60 50 40 30 20 10 0
f1 (ppm)

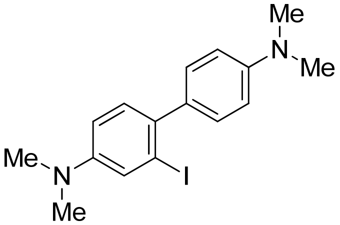

1f

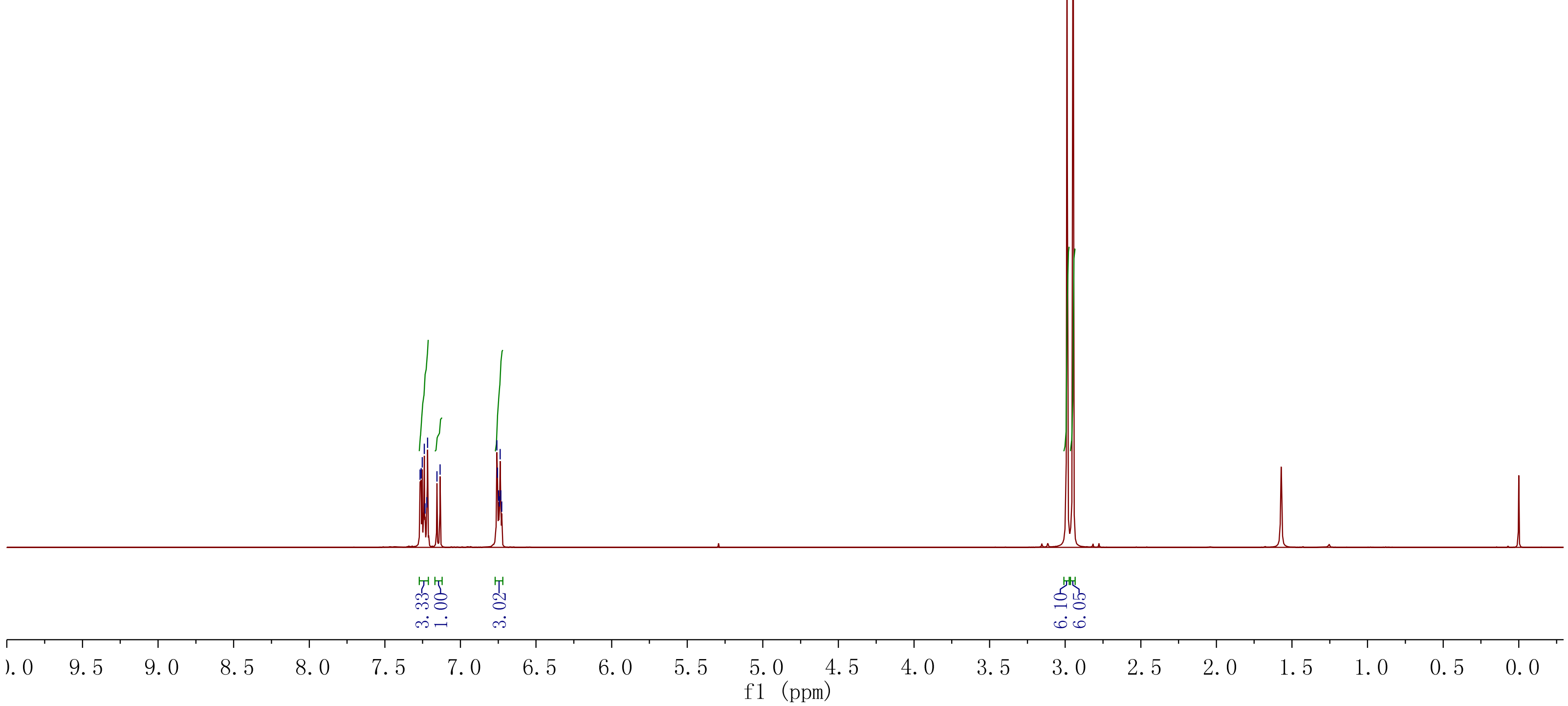
7.27
7.26
7.25
7.24
7.23
7.22
7.22
7.16
7.13
6.76
6.75
6.75
6.74
6.74
6.73
6.73
2.99
2.95
3.33
1.00
3.02
6.10
6.05
f1 (ppm)

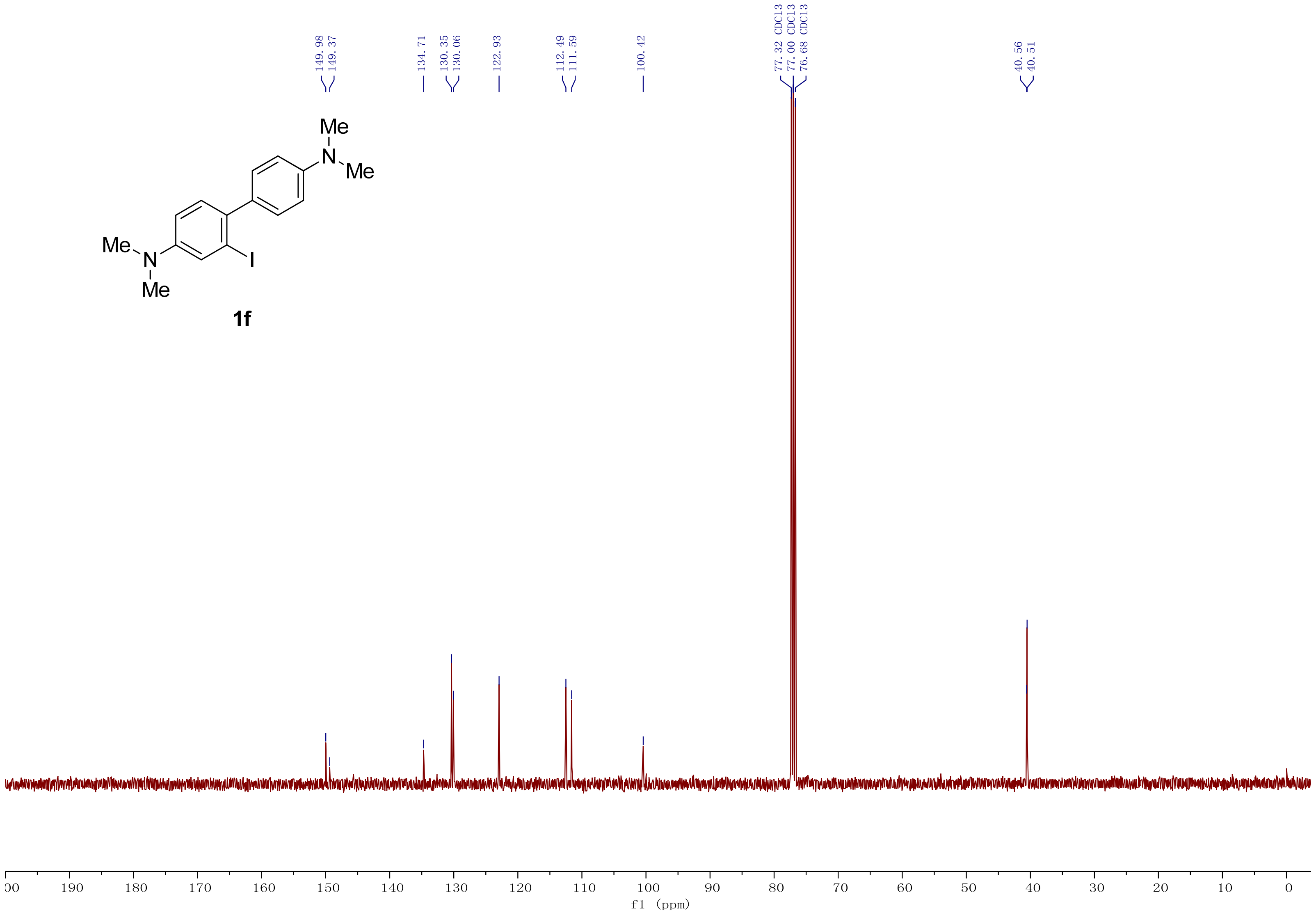

149.98
149.37
134.71
130.35
130.06
122.93
112.49
111.59
100.42
77.32 CDCl3
77.00 CDCl3
76.68 CDCl3
40.56
40.51
00 190 180 170 160 150 140 130 120 110 100 90 80 70 60 50 40 30 20 10 0
f1 (ppm)

1f

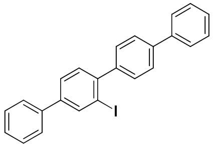

1g

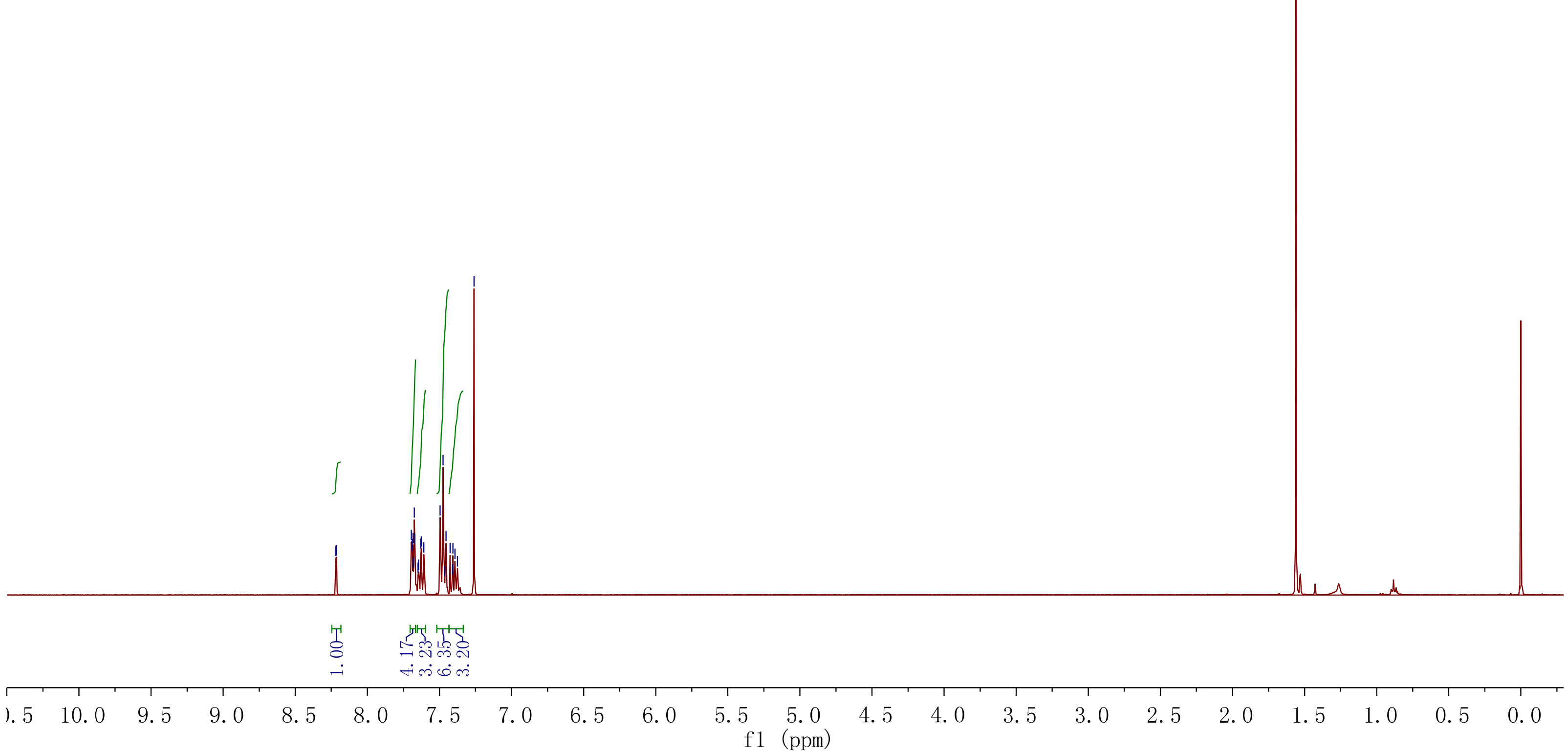
8.22
8.21
7.70
7.69
7.69
7.68
7.68
7.67
7.67
7.65
7.64
7.63
7.63
7.61
7.50
7.47
7.47
7.45
7.43
7.41
7.41
7.39
7.37
7.26
1.00
4.17
3.23
6.35
3.20
f1 (ppm)

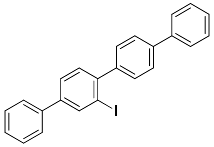

1g

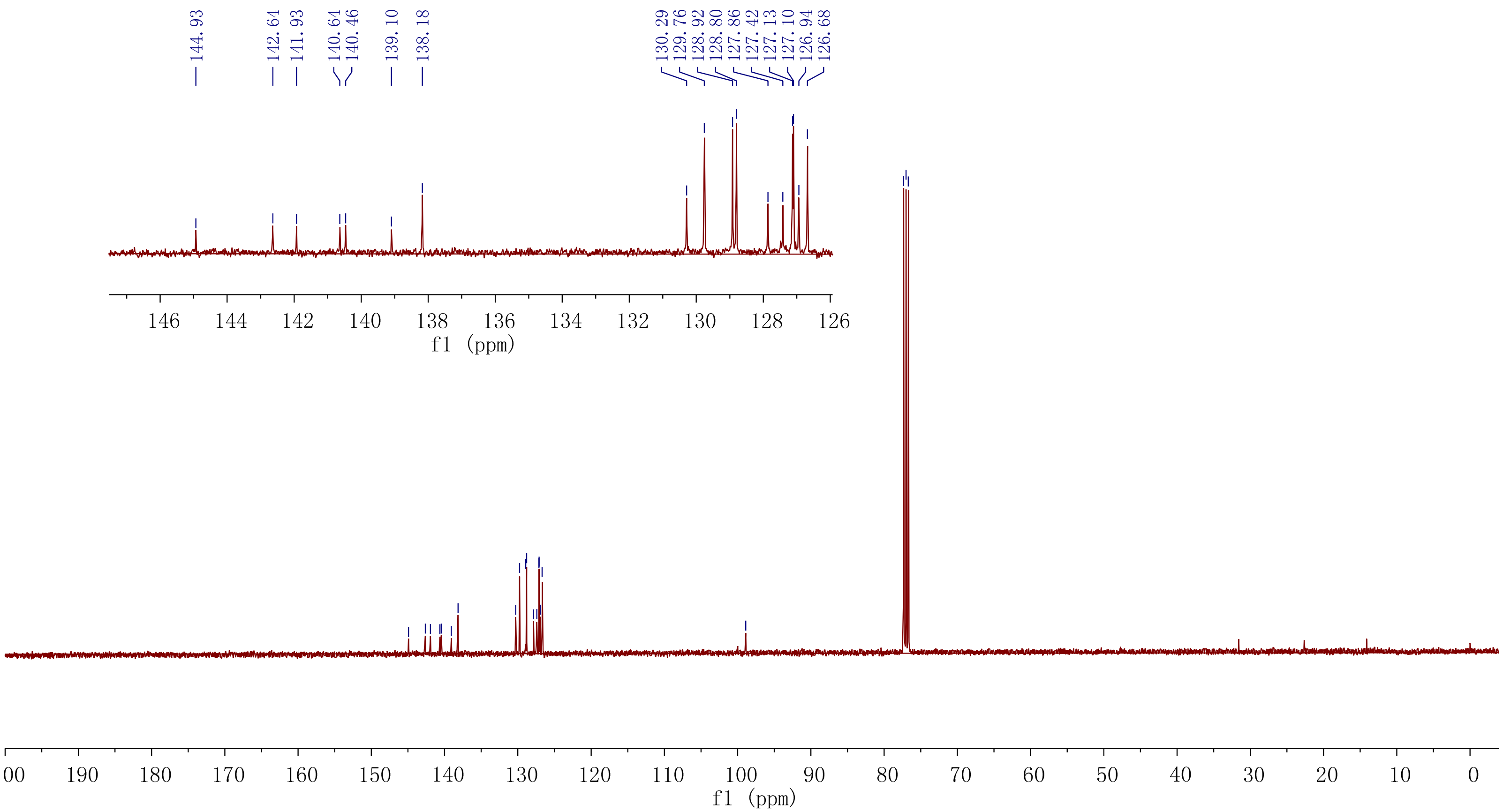
144.93
142.64
141.93
140.64
140.46
139.10
138.18
130.29
129.76
128.92
128.80
127.86
127.42
127.13
127.10
126.94
126.68
98.88
77.32
77.00
76.68
f1 (ppm)
f1 (ppm)

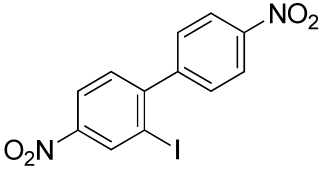
NO2
O2N
I

1h

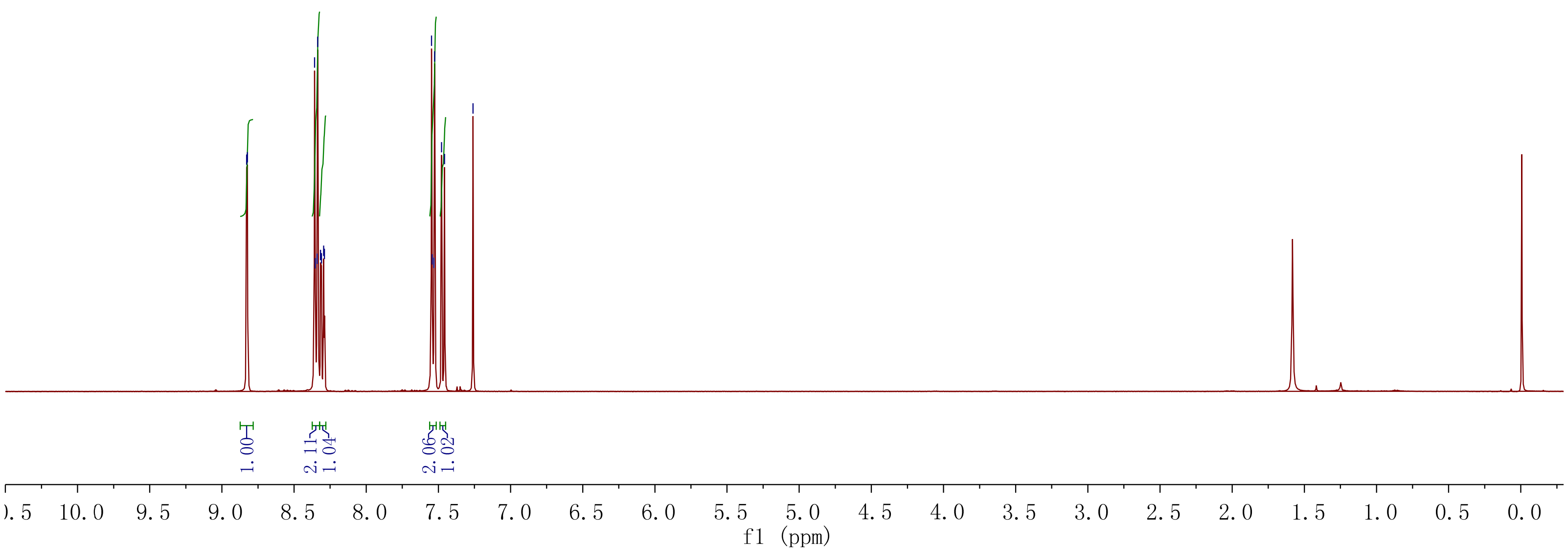
8.83
8.82
8.36
8.34
8.32
8.31
8.30
8.29
7.55
7.54
7.53
7.53
7.48
7.46
7.26
1.00
2.11
1.04
2.06
1.02
).5 10.0 9.5 9.0 8.5 8.0 7.5 7.0 6.5 6.0 5.5 5.0 4.5 4.0 3.5 3.0 2.5 2.0 1.5 1.0 0.5 0.0
f1 (ppm)

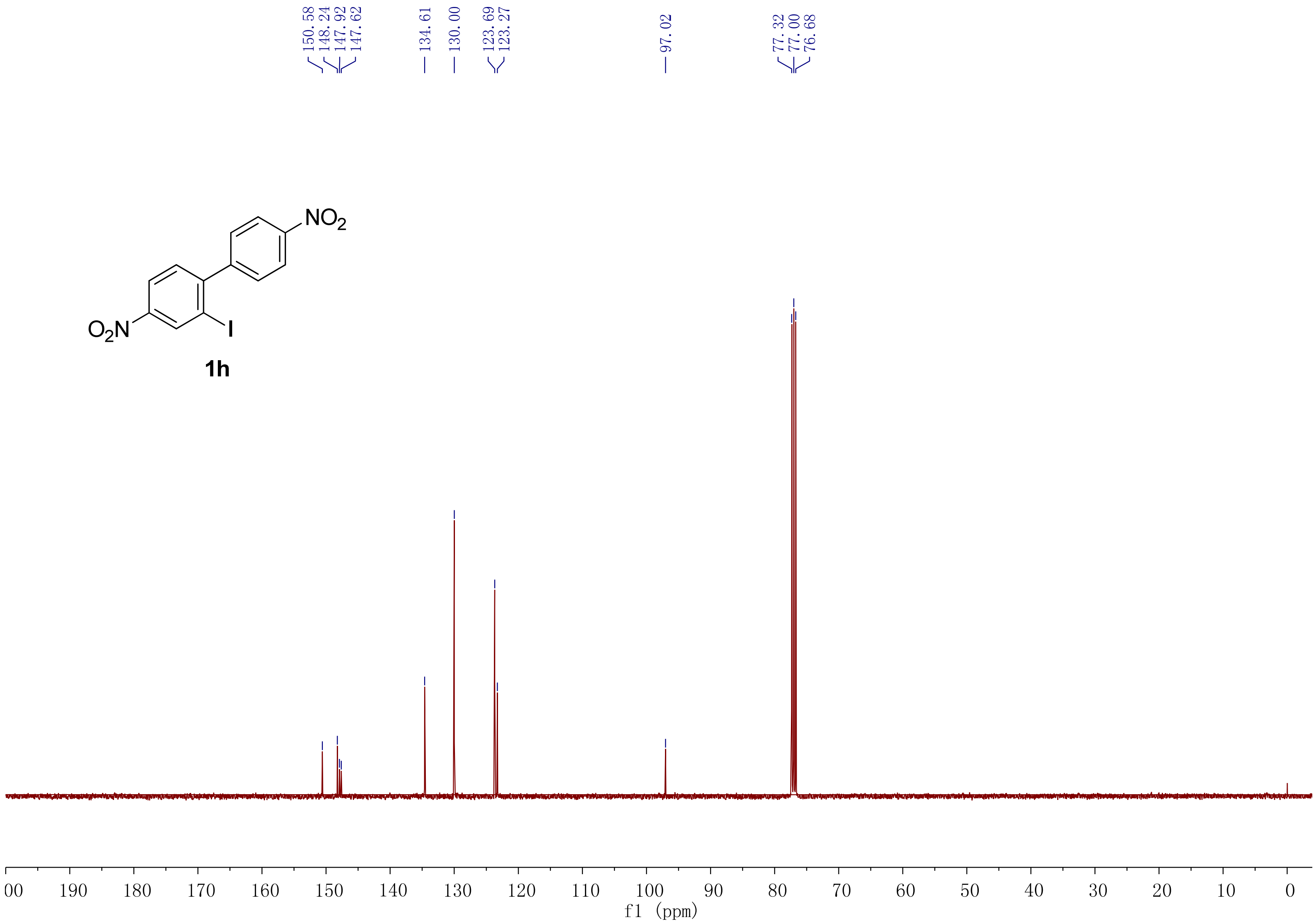
150.58
148.24
147.92
147.62
134.61
130.00
123.69
123.27
97.02
77.32
77.00
76.68
NO2
O2N
I
1h
00
190
180
170
160
150
140
130
120
110
100
90
80
70
60
50
40
30
20
10
0
f1 (ppm)

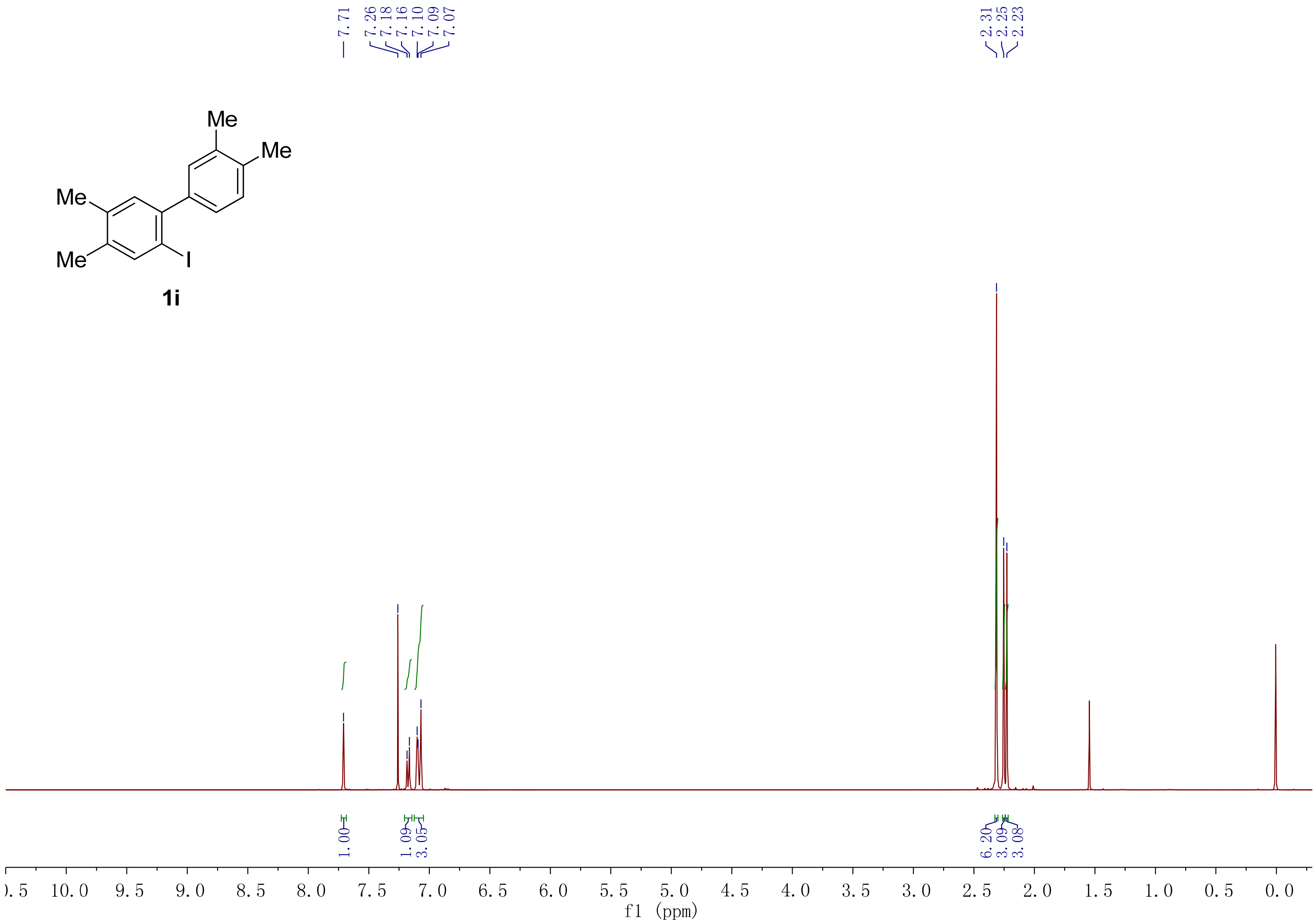
7.71
7.26
7.18
7.16
7.10
7.09
7.07
2.31
2.25
2.23
Me
Me
Me
Me
I
1i
1.00
1.09
3.05
6.20
3.09
3.08
f1 (ppm)

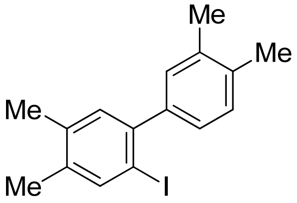
Me
Me
Me
Me
I

1i

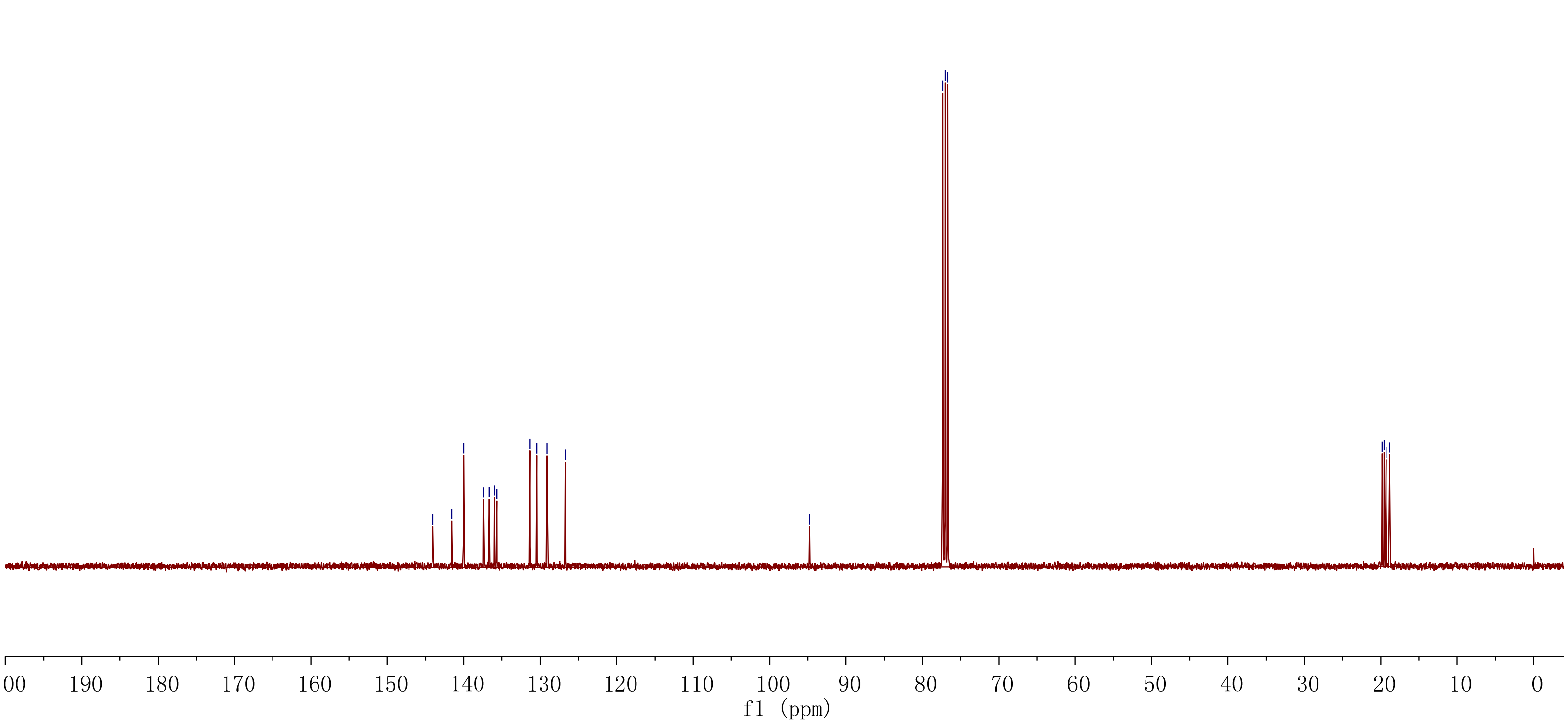
144.05
141.60
140.02
137.42
136.68
136.02
135.70
131.35
130.47
129.08
126.72
94.77
77.32
77.00
76.68
19.82
19.57
19.30
18.85
00 190 180 170 160 150 140 130 120 110 100 90 80 70 60 50 40 30 20 10 0
f1 (ppm)

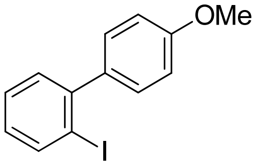

1j

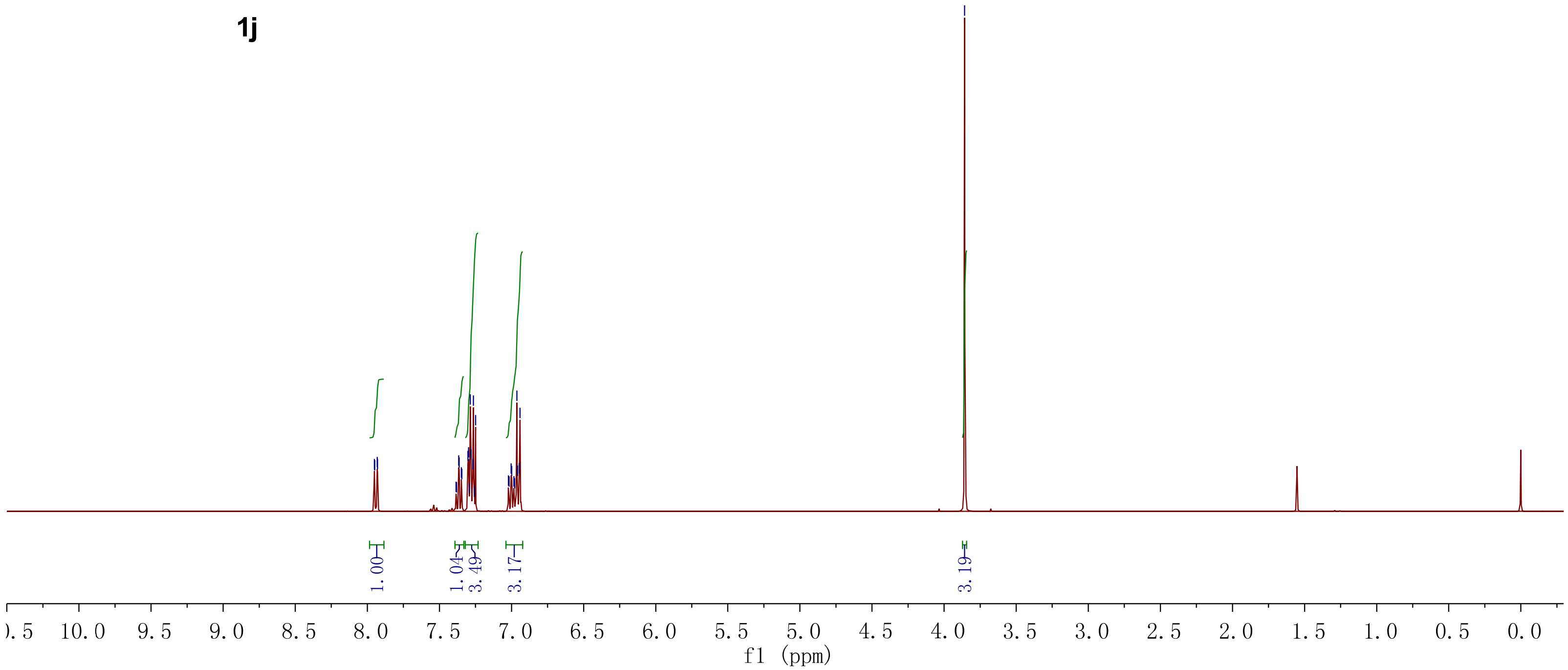

7.95
7.95
7.93
7.93
7.38
7.38
7.37
7.36
7.35
7.34
7.30
7.30
7.29
7.29
7.28
7.27
7.26
7.26
7.25
7.02
7.02
7.00
7.00
6.98
6.98
6.96
6.96
6.95
6.94
3.86
1.00
1.04
3.49
3.17
3.19
f1 (ppm)

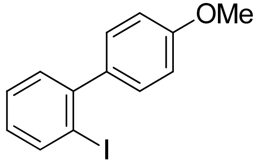
OMe
I

1j

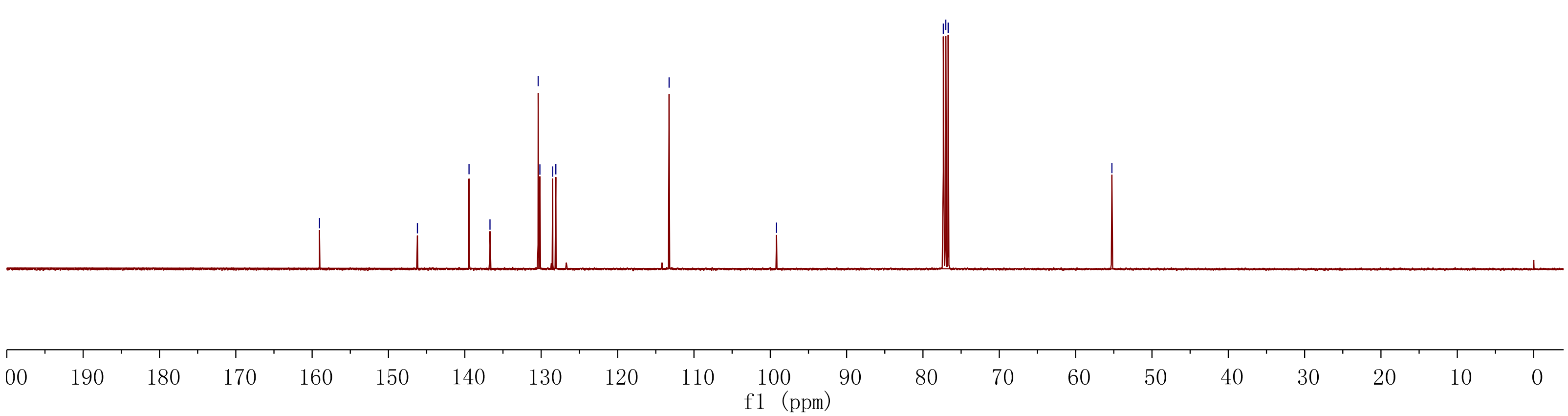
159.05
146.22
139.45
136.71
130.40
130.18
128.49
128.09
113.26
99.17
77.32
77.00
76.68
55.25
00
190
180
170
160
150
140
130
120
110
100
90
80
70
60
50
40
30
20
10
0
f1 (ppm)

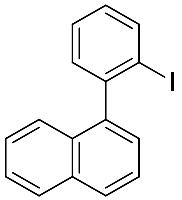

1k

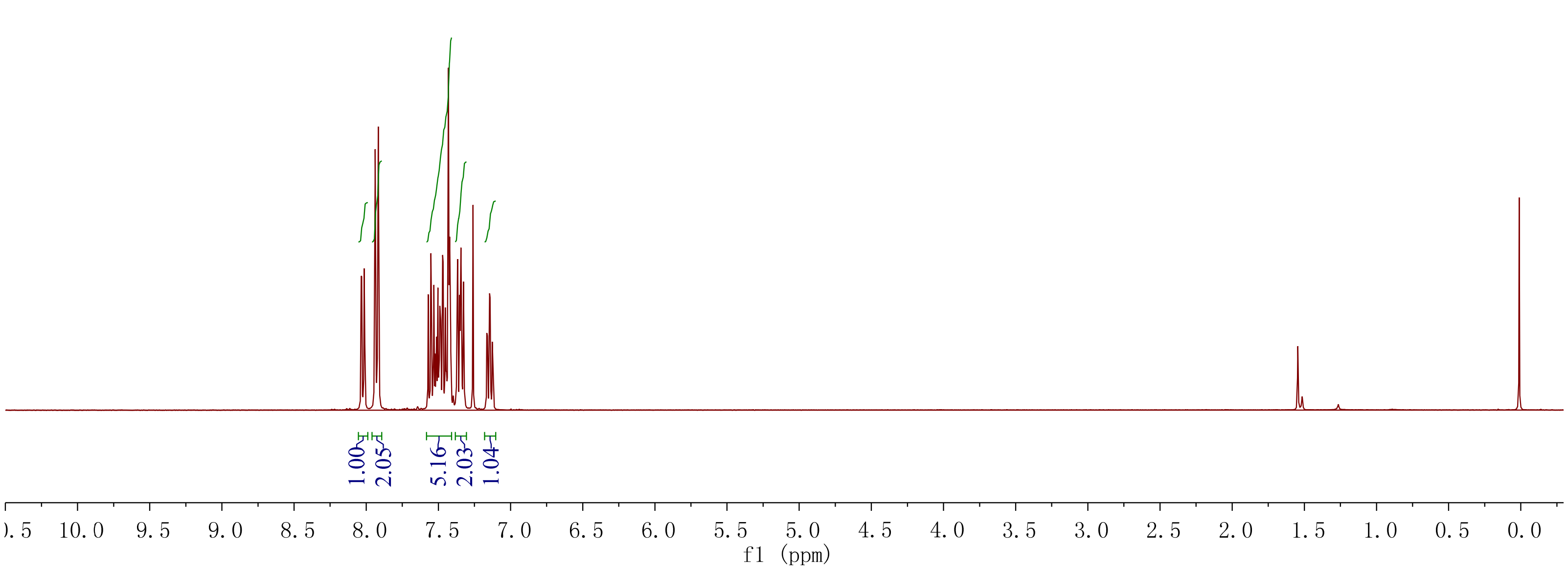
8.03 8.03 8.01 8.01 7.94 7.92 7.57 7.55 7.55 7.53 7.52 7.51 7.50 7.49 7.49 7.48 7.47 7.47 7.45 7.45 7.43 7.42 7.42 7.42 7.37 7.37 7.35 7.35 7.34 7.34 7.33 7.32 7.26 7.16 7.16 7.15 7.14 7.13 7.12
1.00 2.05 5.16 2.03 1.04
0.5 10.0 9.5 9.0 8.5 8.0 7.5 7.0 6.5 6.0 5.5 5.0 4.5 4.0 3.5 3.0 2.5 2.0 1.5 1.0 0.5 0.0
f1 (ppm)

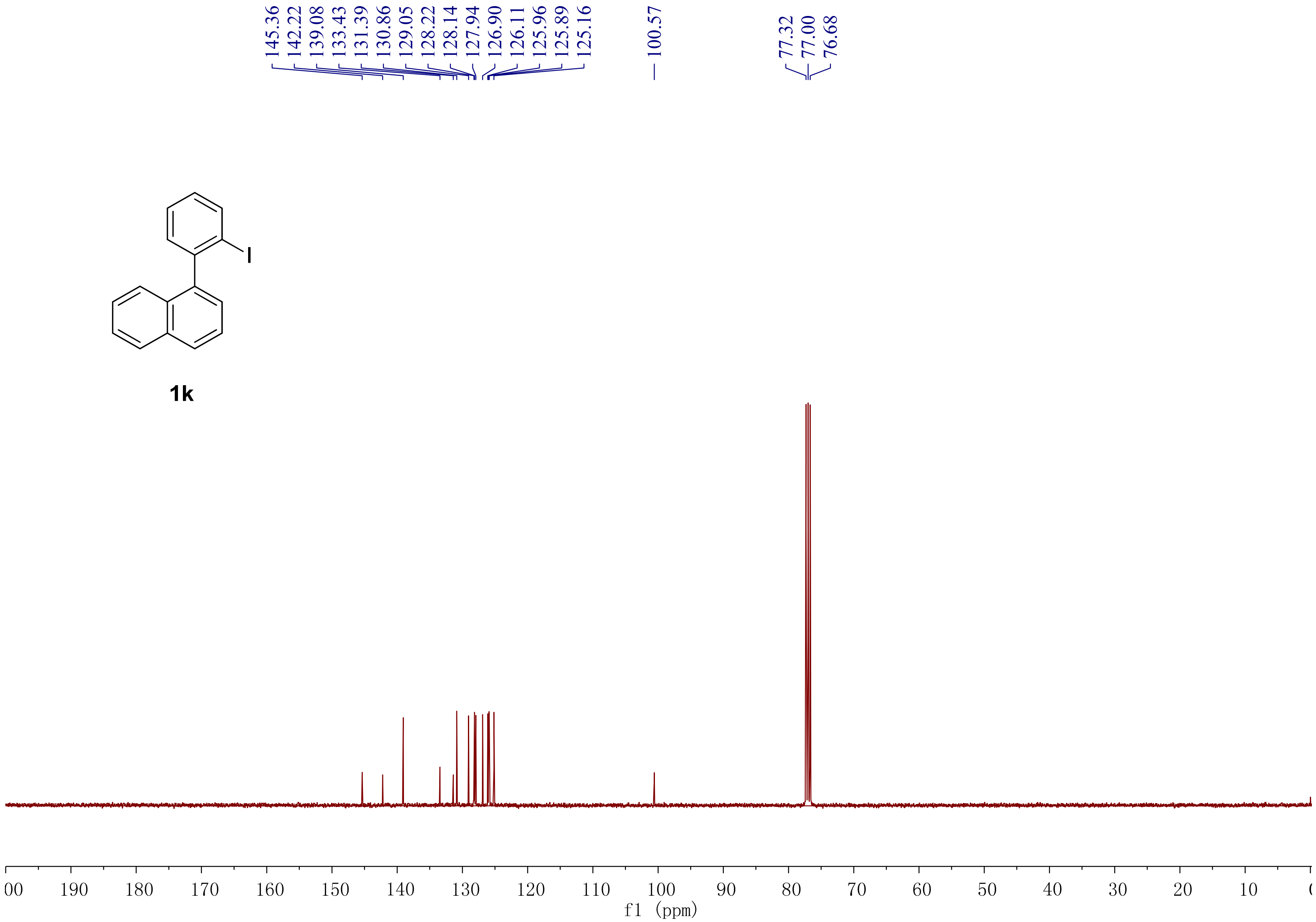
1k
145.36
142.22
139.08
133.43
131.39
130.86
129.05
128.22
128.14
127.94
126.90
126.11
125.96
125.89
125.16
100.57
77.32
77.00
76.68
f1 (ppm)

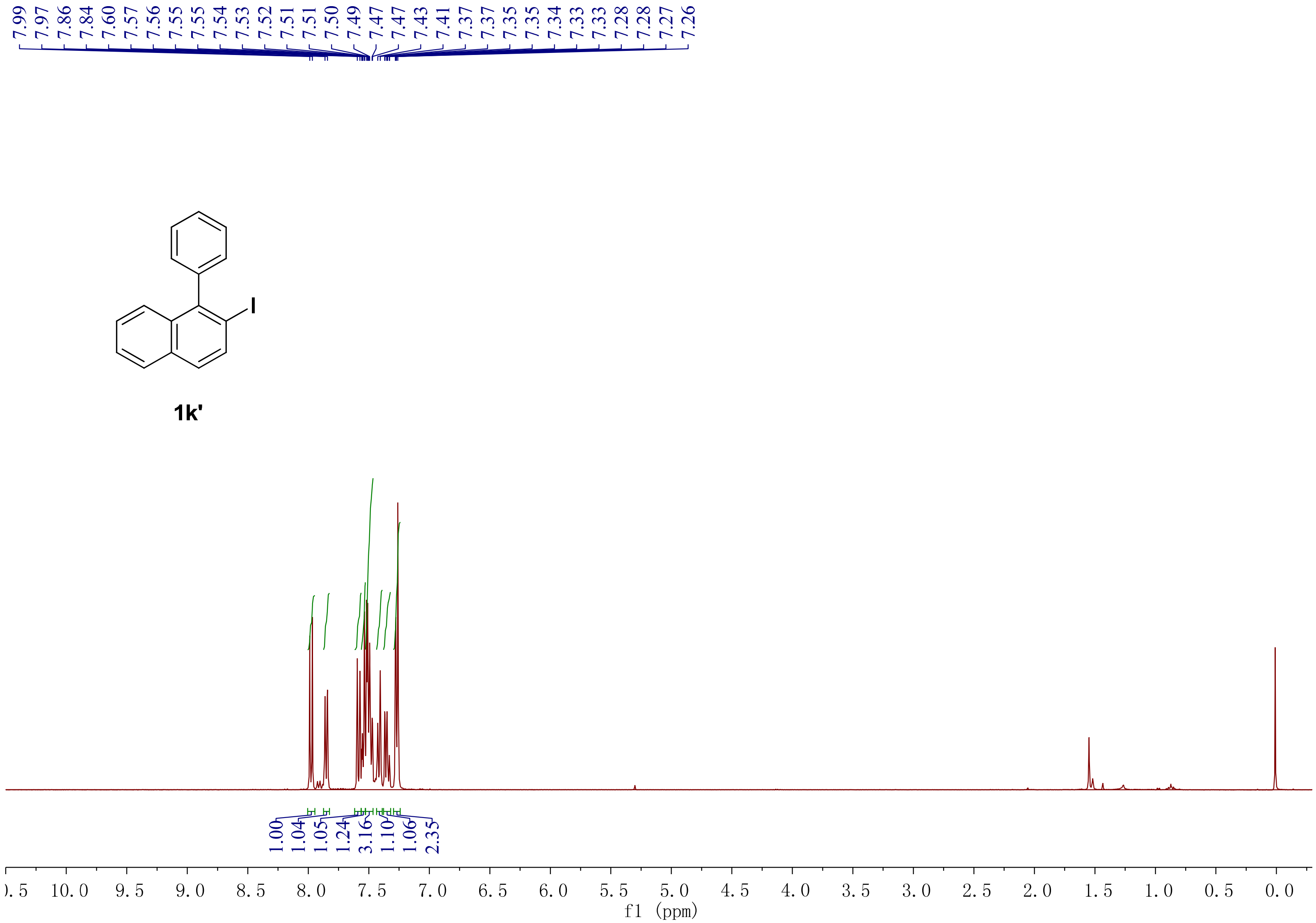
7.99 7.97 7.86 7.84 7.60 7.57 7.56 7.55 7.55 7.54 7.53 7.52 7.51 7.51 7.50 7.49 7.47 7.47 7.43 7.41 7.37 7.37 7.35 7.35 7.34 7.33 7.33 7.28 7.28 7.27 7.26
I
1k'
1.00 1.04 1.05 1.24 3.16 1.10 1.06 2.35
10.0 9.5 9.0 8.5 8.0 7.5 7.0 6.5 6.0 5.5 5.0 4.5 4.0 3.5 3.0 2.5 2.0 1.5 1.0 0.5 0.0
f1 (ppm)

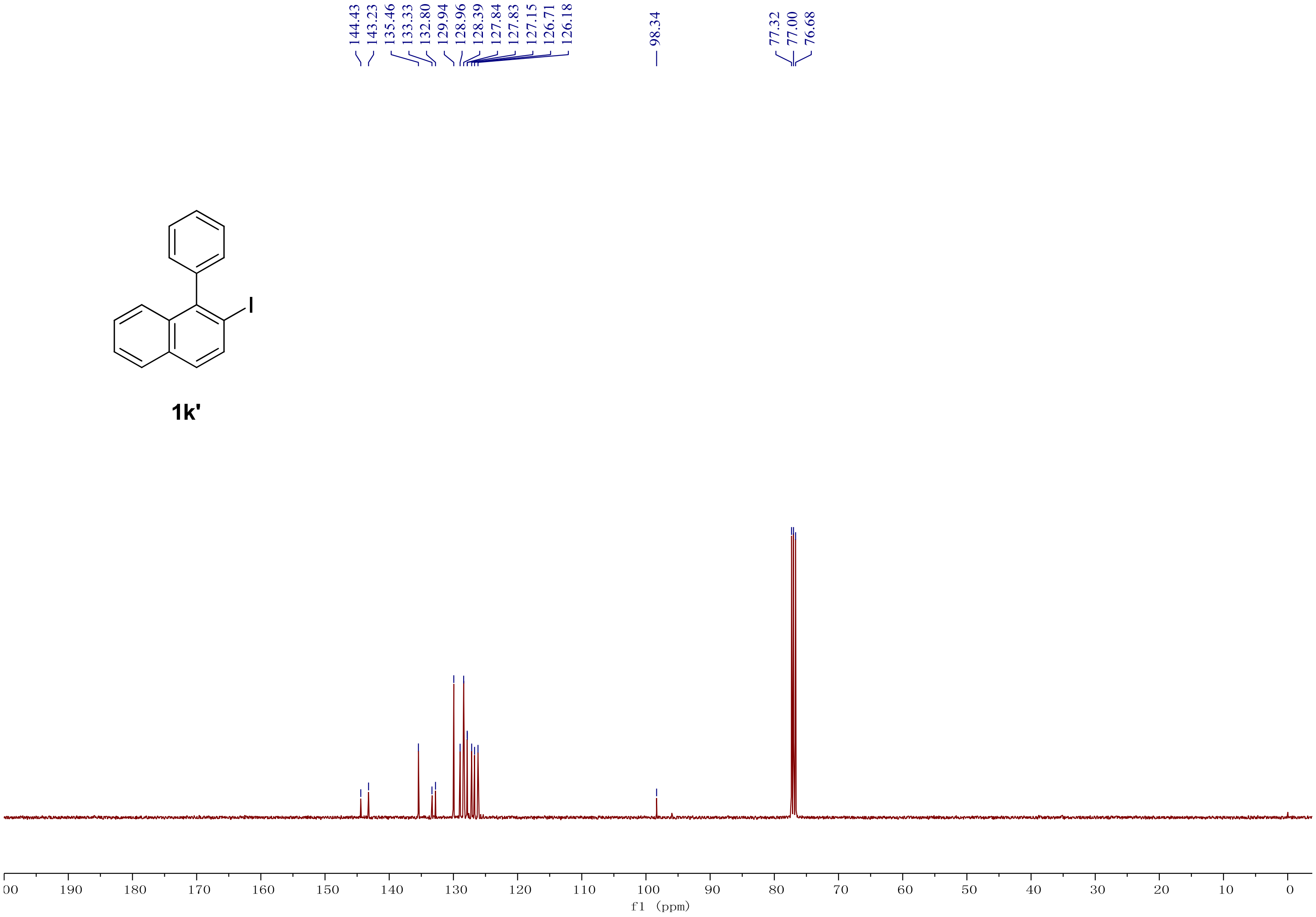
1k'
144.43
143.23
135.46
133.33
132.80
129.94
128.96
128.39
127.84
127.83
127.15
126.71
126.18
98.34
77.32
77.00
76.68
00 190 180 170 160 150 140 130 120 110 100 90 80 70 60 50 40 30 20 10 0
f1 (ppm)

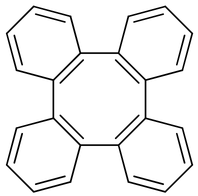

2aa

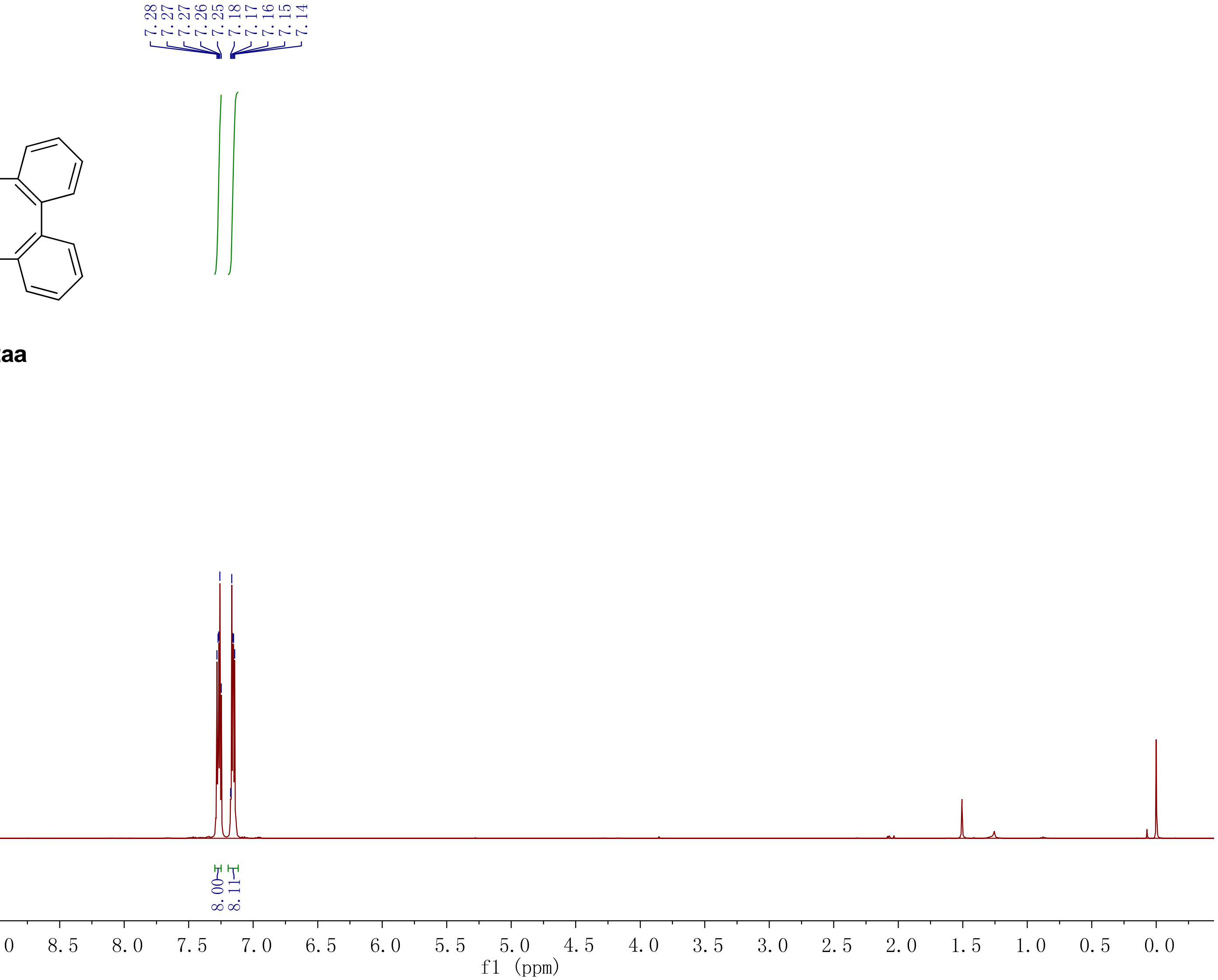
7.28
7.27
7.27
7.26
7.25
7.18
7.17
7.16
7.15
7.14
8.00
8.11
10.0 9.5 9.0 8.5 8.0 7.5 7.0 6.5 6.0 5.5 5.0 4.5 4.0 3.5 3.0 2.5 2.0 1.5 1.0 0.5 0.0
f1 (ppm)

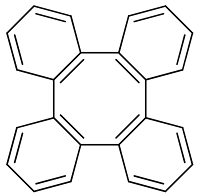

2aa

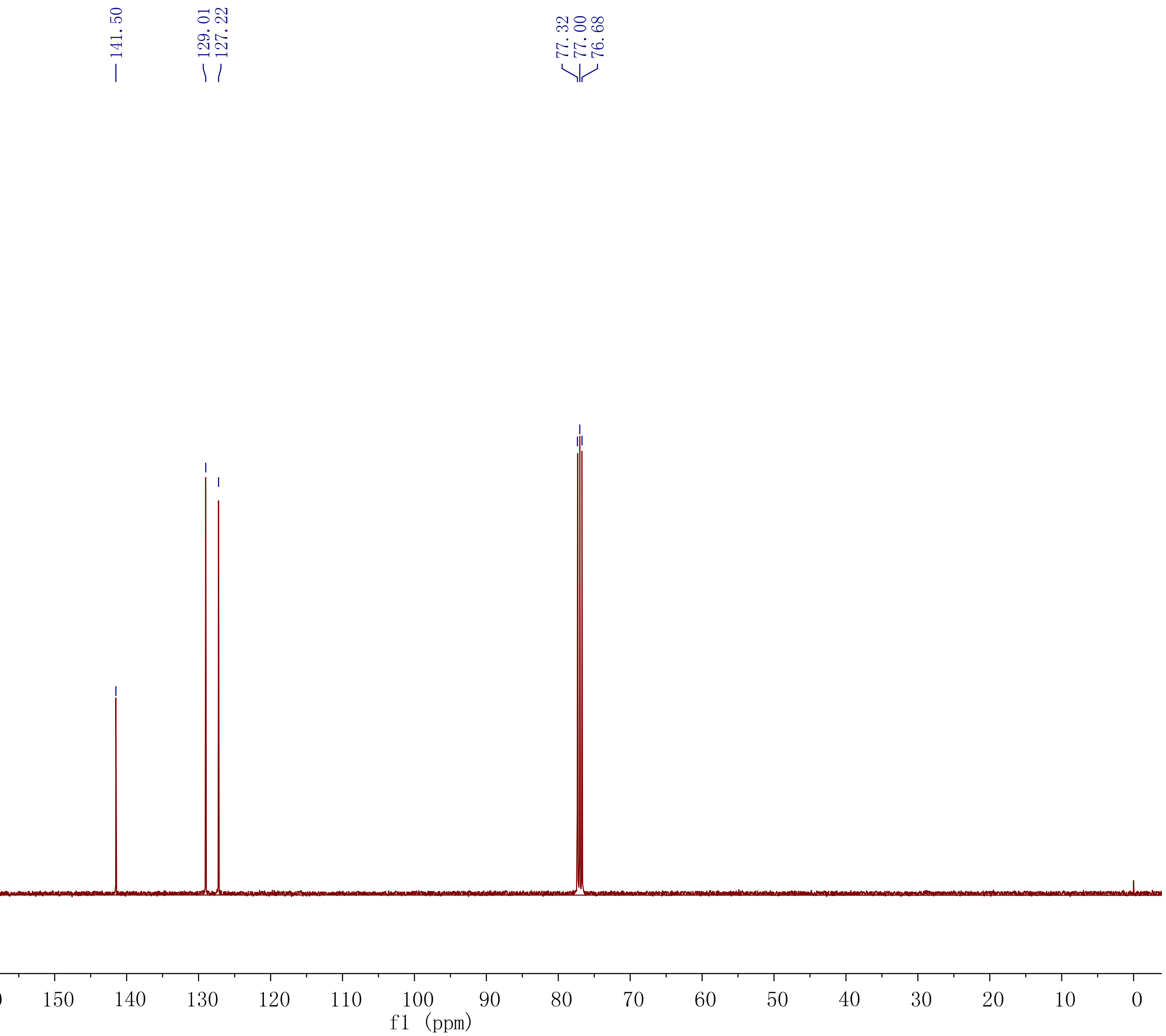

141.50
129.01
127.22
77.32
77.00
76.68
00
190
180
170
160
150
140
130
120
110
100
90
80
70
60
50
40
30
20
10
0
f1 (ppm)

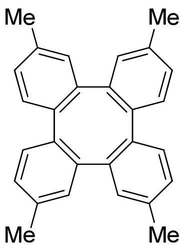
Me
Me
Me
Me

2bb/2cc

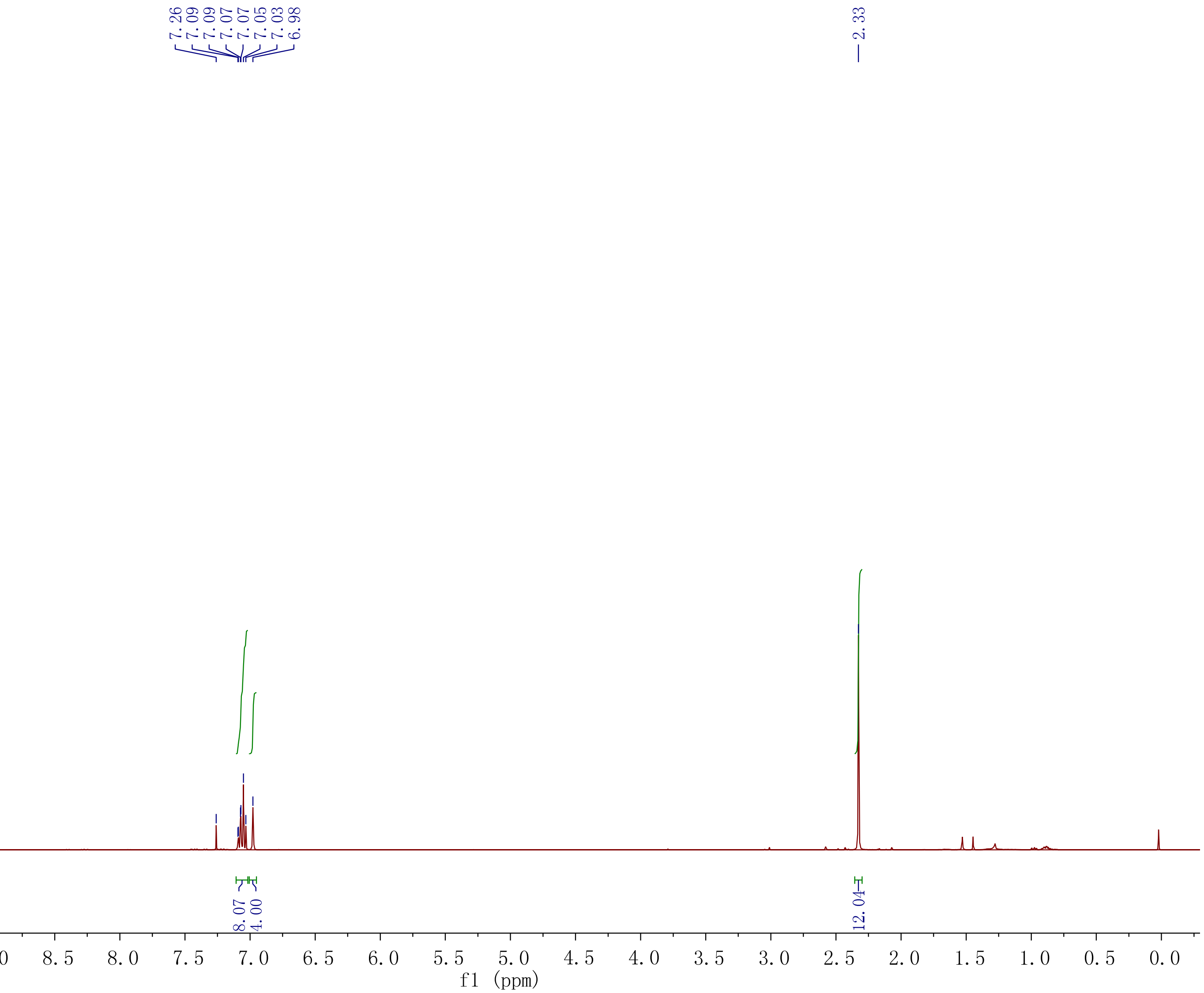
7.26
7.09
7.09
7.07
7.07
7.05
7.03
6.98
2.33
8.07
4.00
12.04
10.0
9.5
9.0
8.5
8.0
7.5
7.0
6.5
6.0
5.5
5.0
4.5
4.0
3.5
3.0
2.5
2.0
1.5
1.0
0.5
0.0
f1 (ppm)

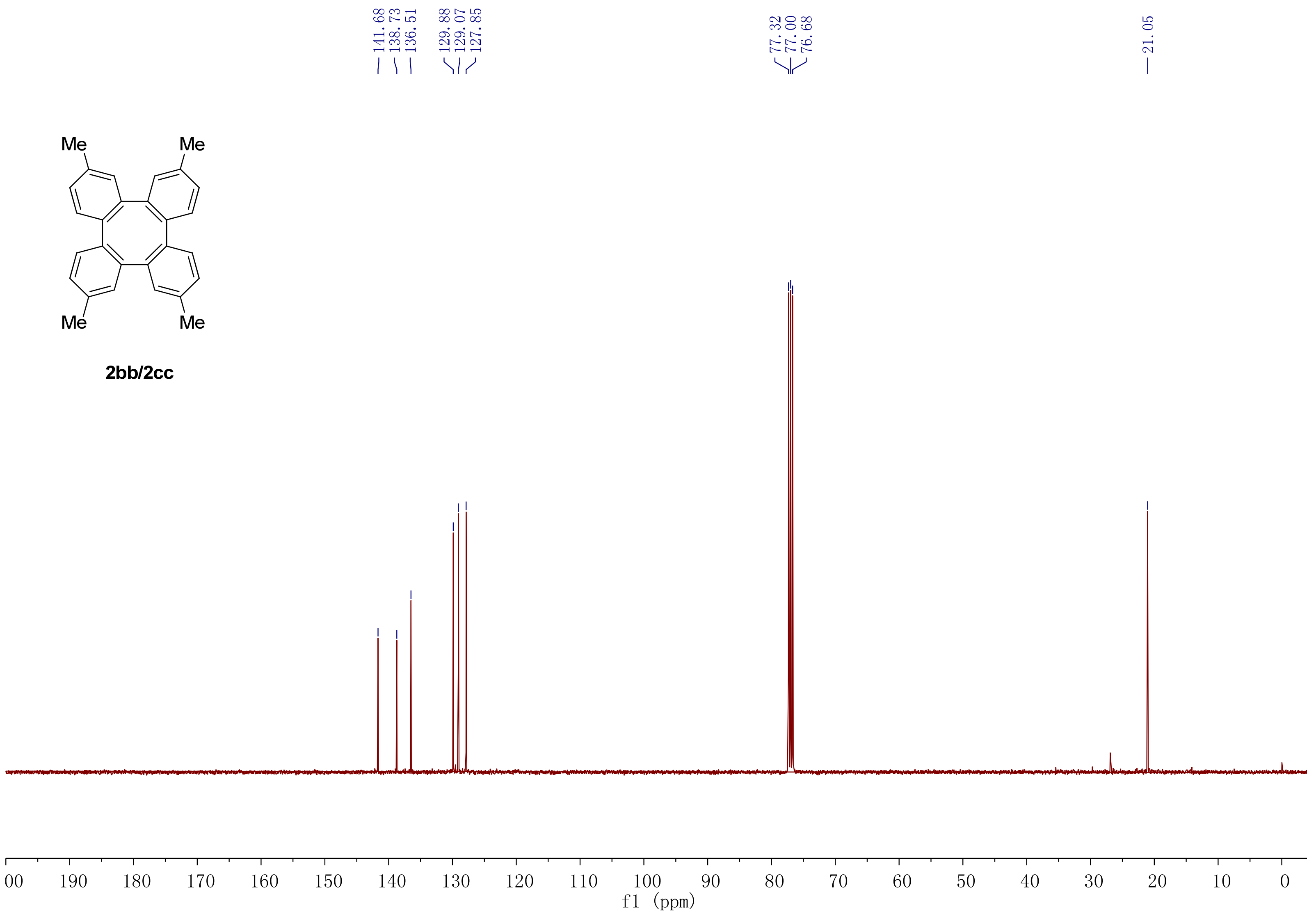
141.68
138.73
136.51
129.88
129.07
127.85
77.32
77.00
76.68
21.05
Me
Me
Me
Me
00
190
180
170
160
150
140
130
120
110
100
90
80
70
60
50
40
30
20
10
0
f1 (ppm)

2bb/2cc

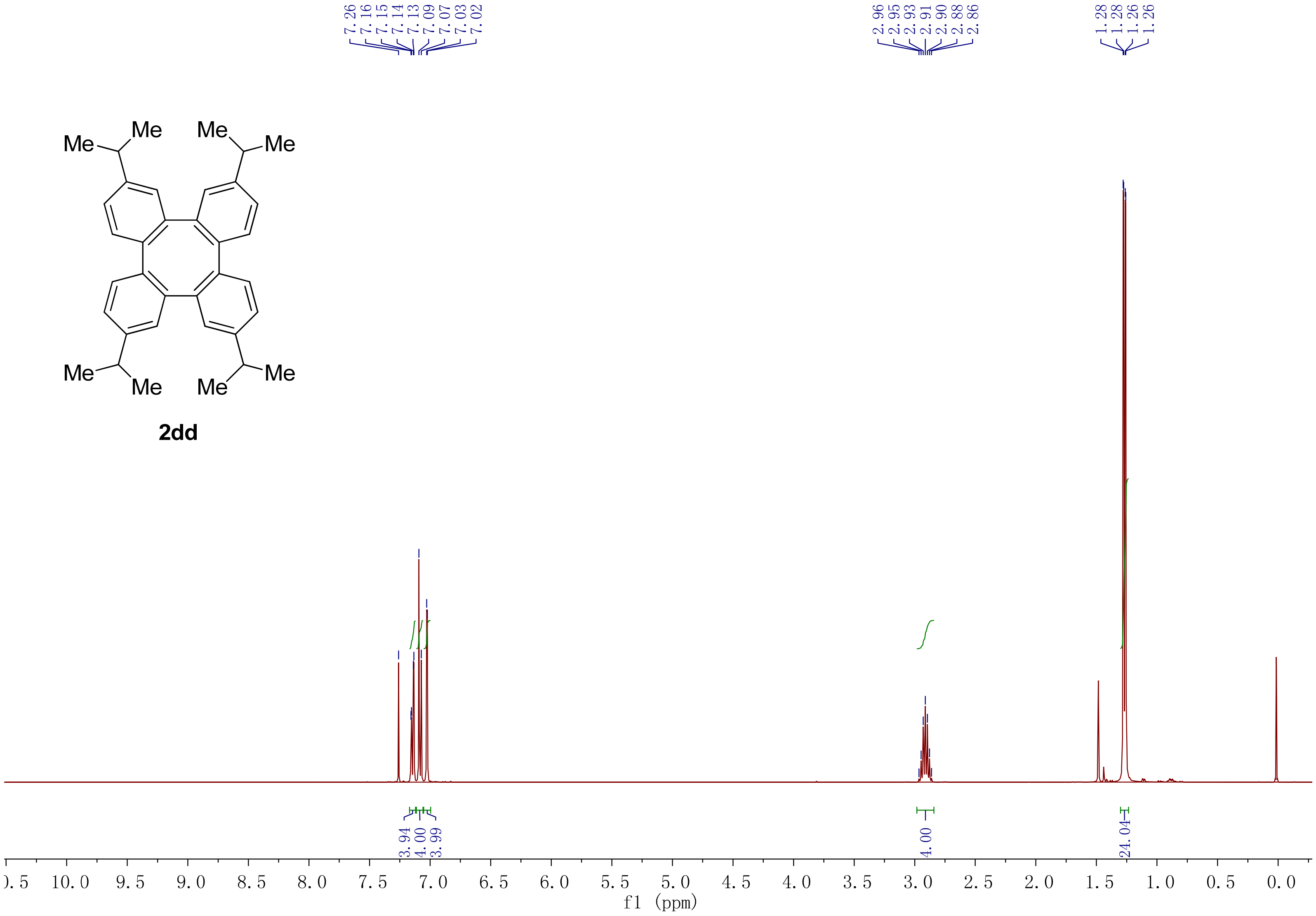

7.26
7.16
7.15
7.14
7.13
7.09
7.07
7.03
7.02
2.96
2.95
2.93
2.91
2.90
2.88
2.86
1.28
1.28
1.26
1.26
Me
Me
2dd
3.94
4.00
3.99
4.00
24.04
f1 (ppm)

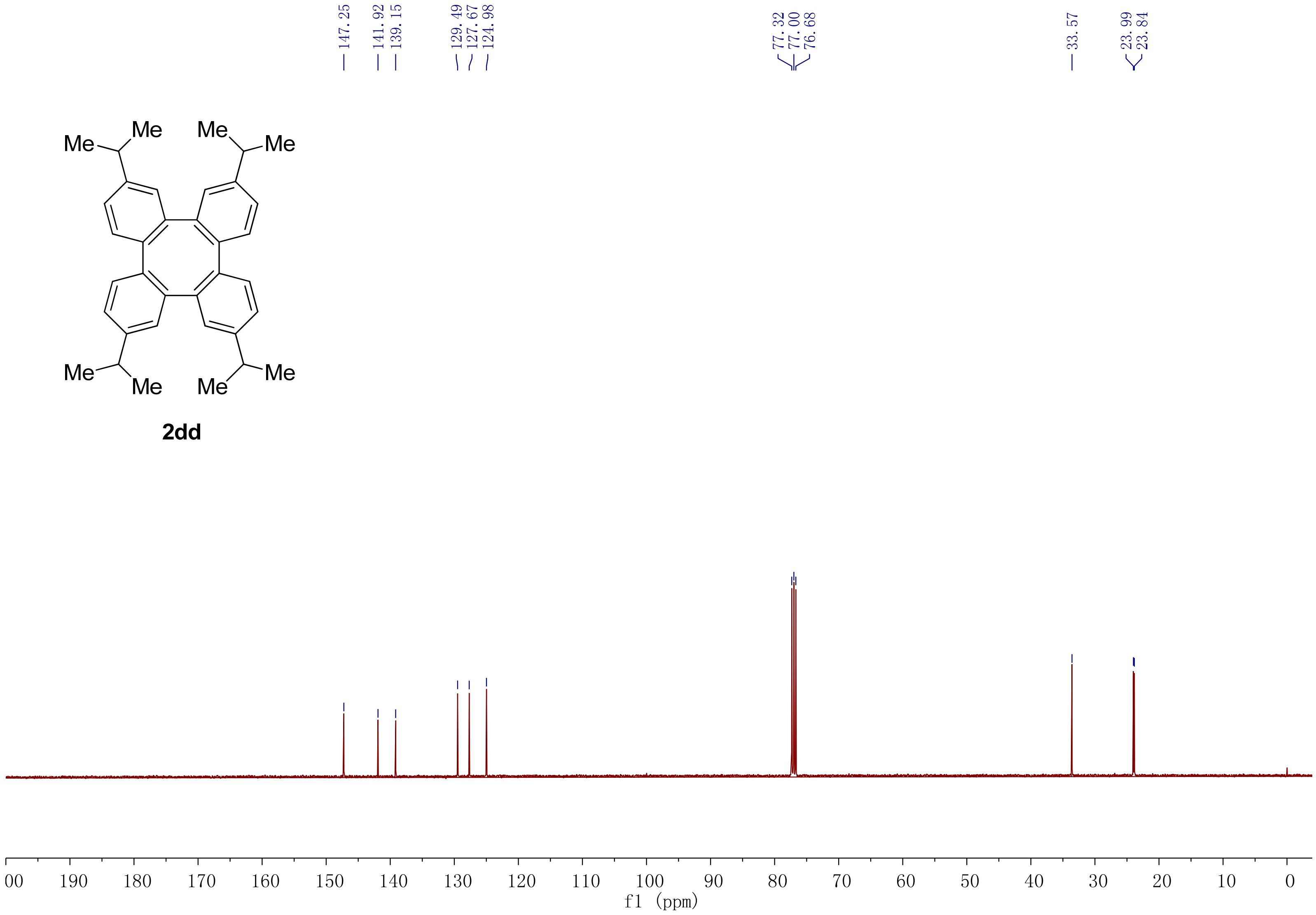

147.25
141.92
139.15
129.49
127.67
124.98
77.32
77.00
76.68
33.57
23.99
23.84
Me
Me
Me
Me
Me
Me
Me
Me
2dd
00
190
180
170
160
150
140
130
120
110
100
90
80
70
60
50
40
30
20
10
0
f1 (ppm)

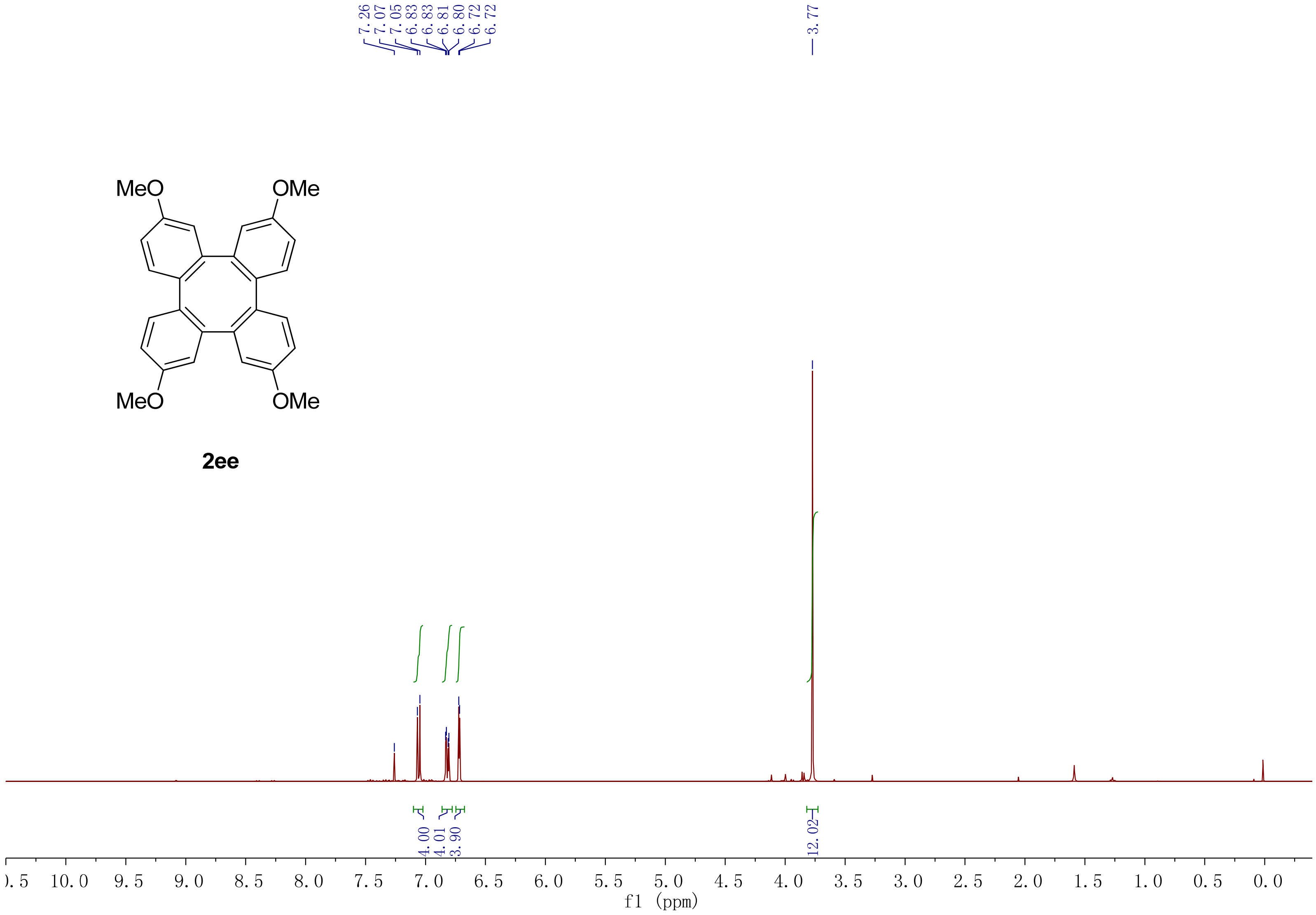
7.26
7.07
7.05
6.83
6.83
6.81
6.80
6.72
6.72
3.77
MeO
OMe
MeO
OMe
4.00
4.01
3.90
12.02
10.0
9.5
9.0
8.5
8.0
7.5
7.0
6.5
6.0
5.5
5.0
4.5
4.0
3.5
3.0
2.5
2.0
1.5
1.0
0.5
0.0
f1 (ppm)

2ee

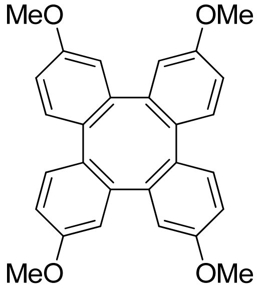
MeO
OMe
MeO
OMe

2ee

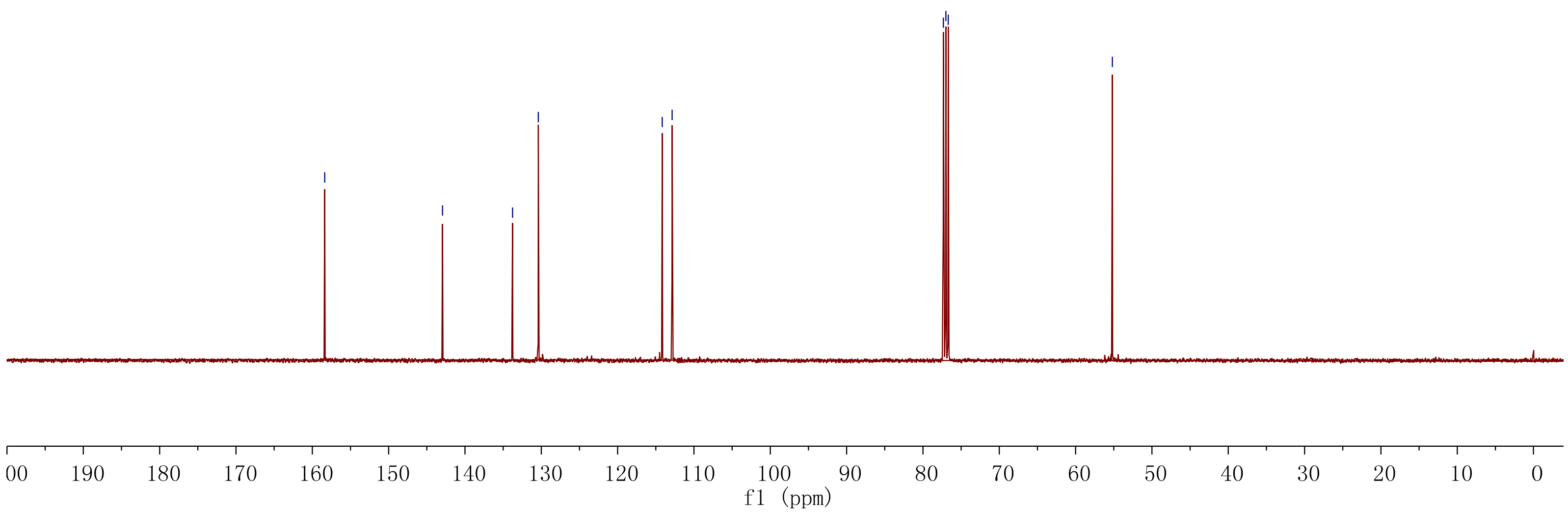
158.39
142.94
133.76
130.39
114.16
112.86
77.32
77.00
76.68
55.19
00 190 180 170 160 150 140 130 120 110 100 90 80 70 60 50 40 30 20 10 0
f1 (ppm)

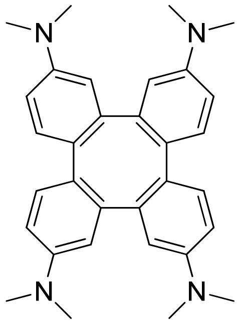

2ff

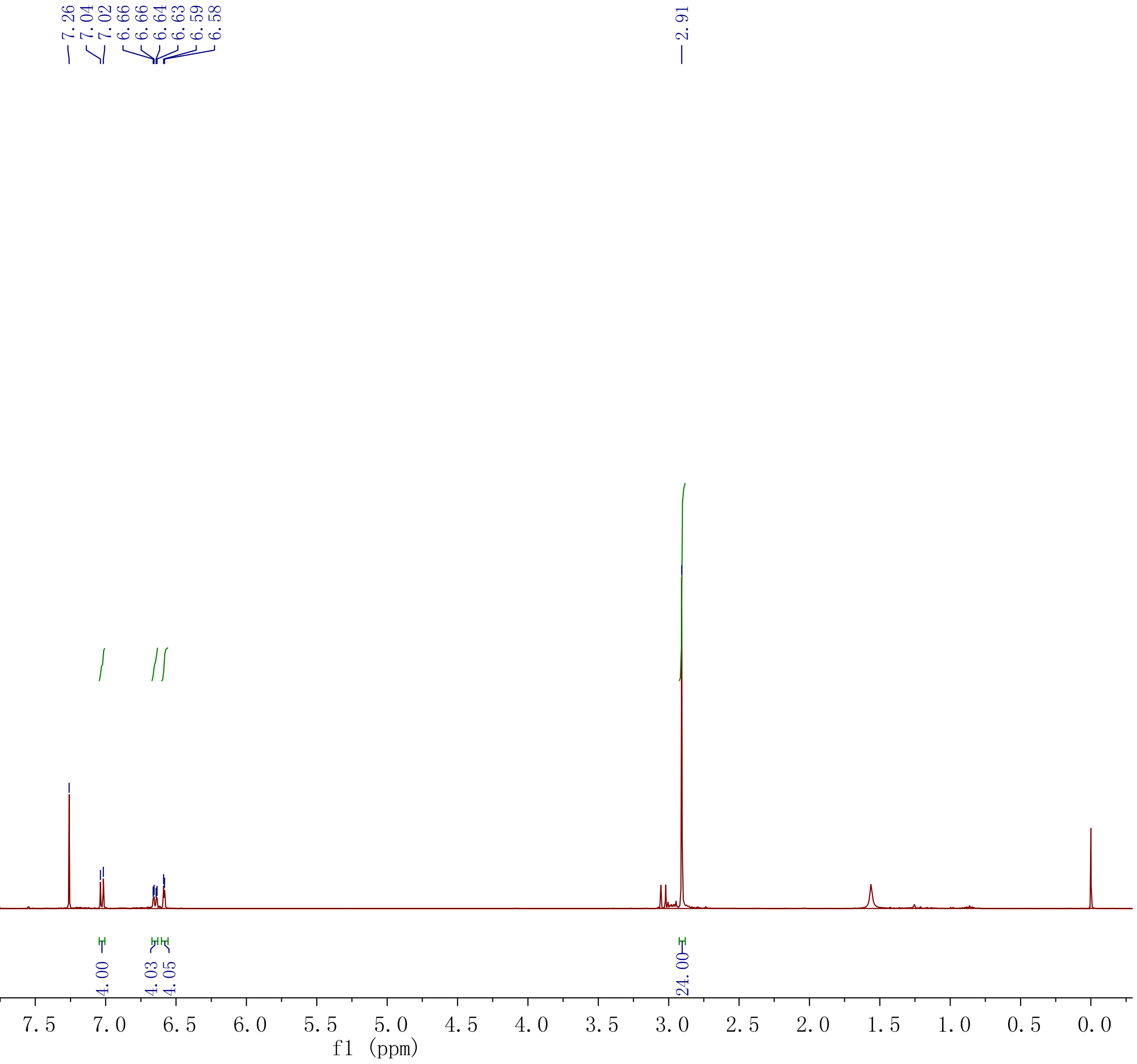
7.26
7.04
7.02
6.66
6.66
6.64
6.63
6.59
6.58
2.91
4.00
4.03
4.05
24.00
0.5 10.0 9.5 9.0 8.5 8.0 7.5 7.0 6.5 6.0 5.5 5.0 4.5 4.0 3.5 3.0 2.5 2.0 1.5 1.0 0.5 0.0
f1 (ppm)

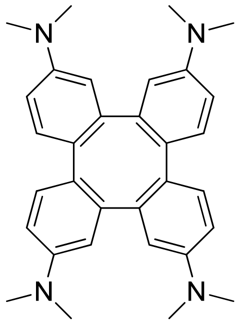
N
N
N
N

2ff

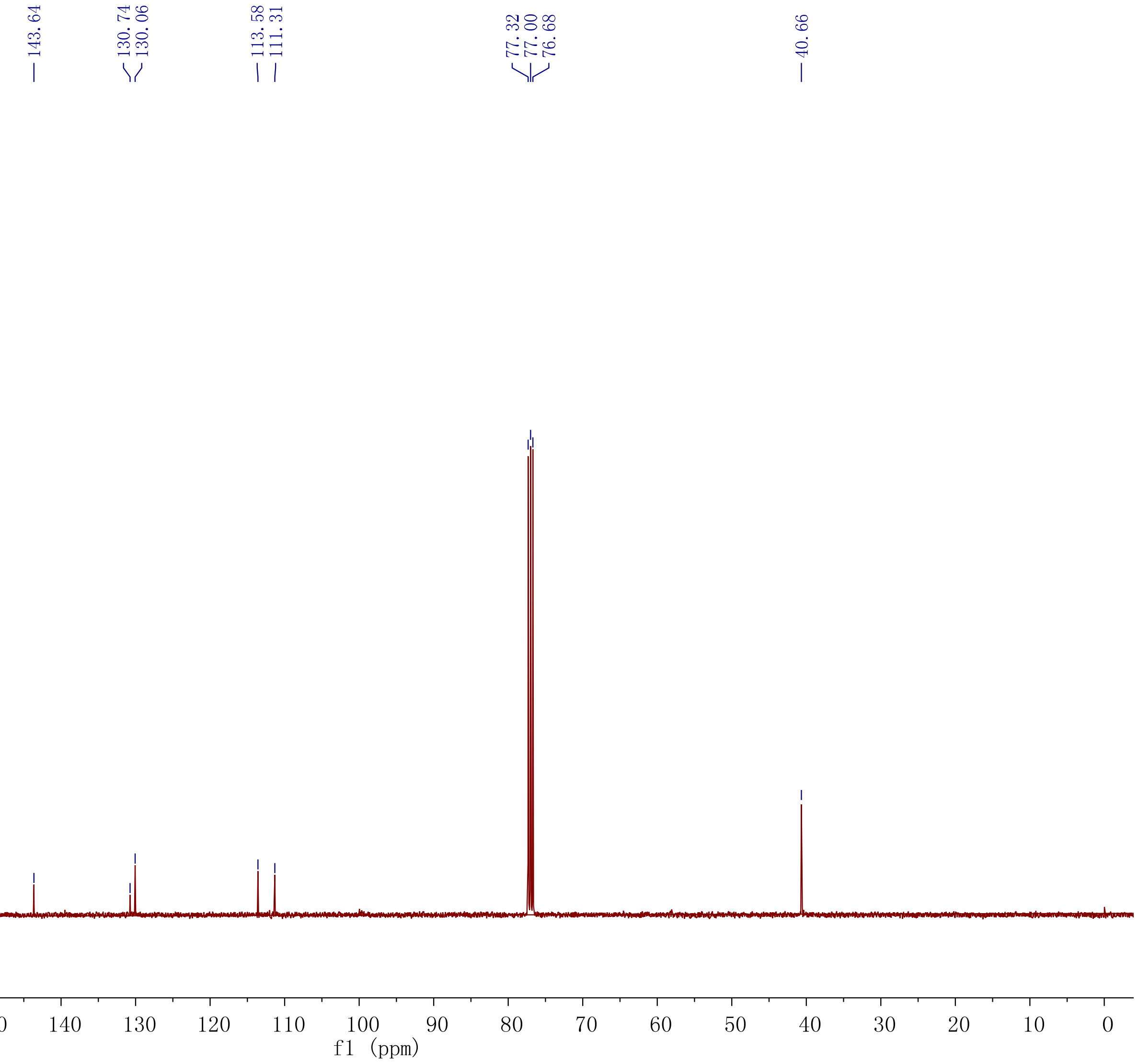
149.07
143.64
130.74
130.06
113.58
111.31
77.32
77.00
76.68
40.66
00
190
180
170
160
150
140
130
120
110
100
90
80
70
60
50
40
30
20
10
0
f1 (ppm)

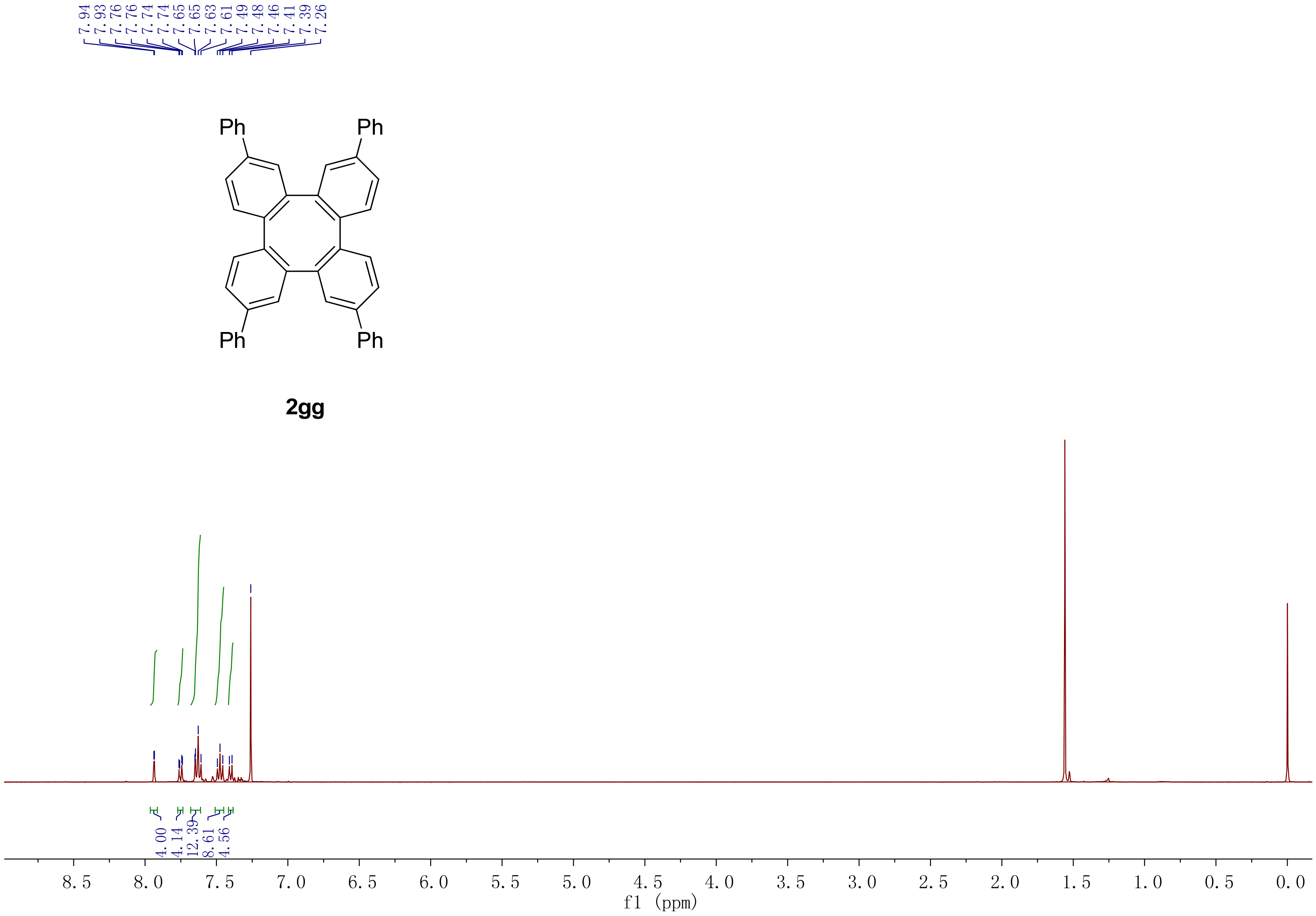
7.94
7.93
7.76
7.76
7.74
7.74
7.65
7.65
7.63
7.61
7.49
7.48
7.46
7.41
7.39
7.26
Ph
Ph
Ph
Ph
4.00
4.14
12.39
8.61
4.56
8.5
8.0
7.5
7.0
6.5
6.0
5.5
5.0
4.5
4.0
3.5
3.0
2.5
2.0
1.5
1.0
0.5
0.0
f1 (ppm)

2gg

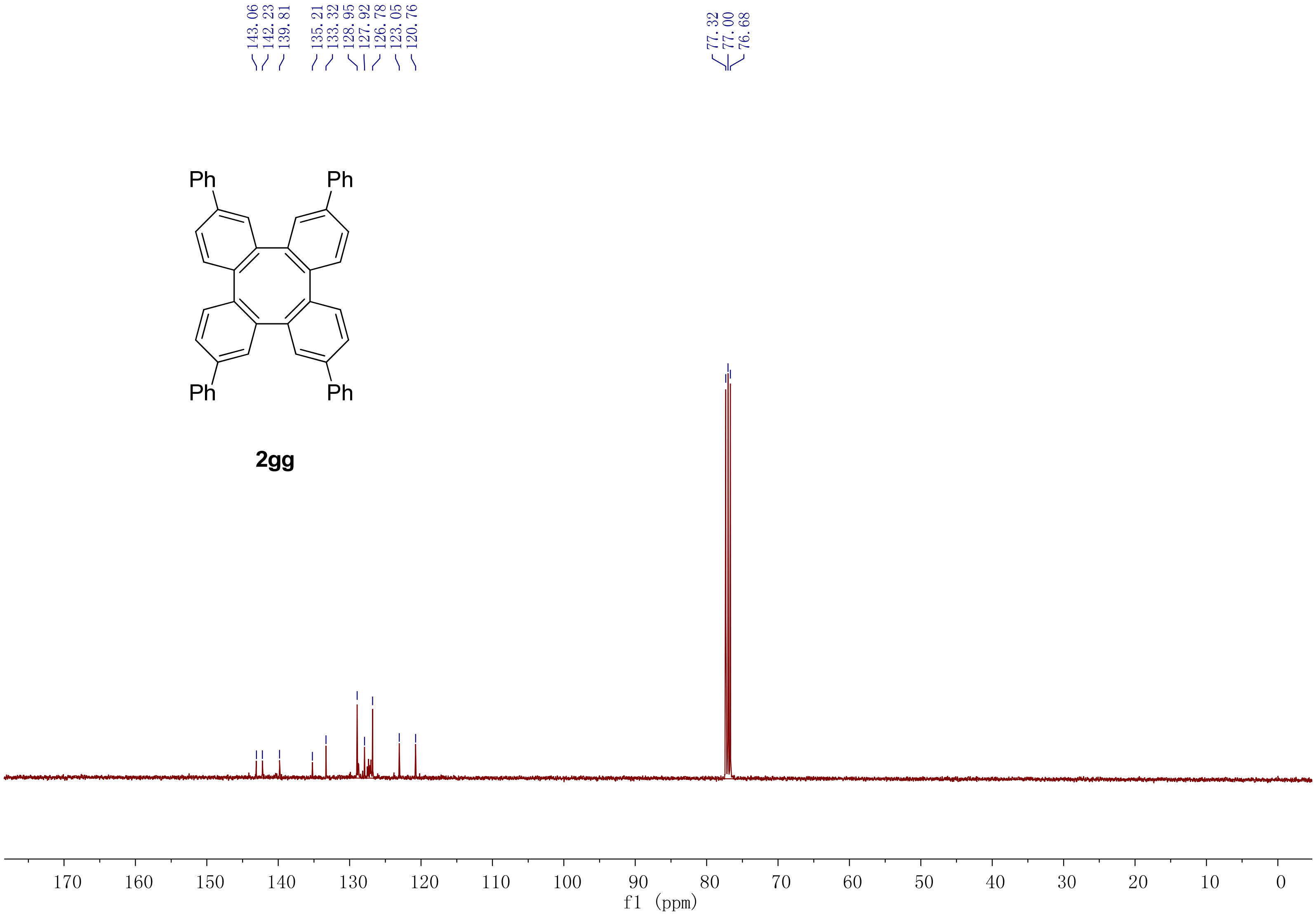
143.06
142.23
139.81
135.21
133.32
128.95
127.92
126.78
123.05
120.76
77.32
77.00
76.68
Ph
Ph
Ph
Ph
2gg
170
160
150
140
130
120
110
100
90
80
70
60
50
40
30
20
10
0
f1 (ppm)

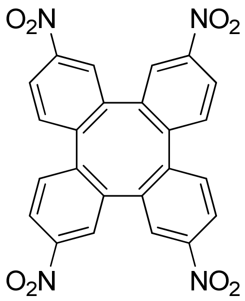

2hh

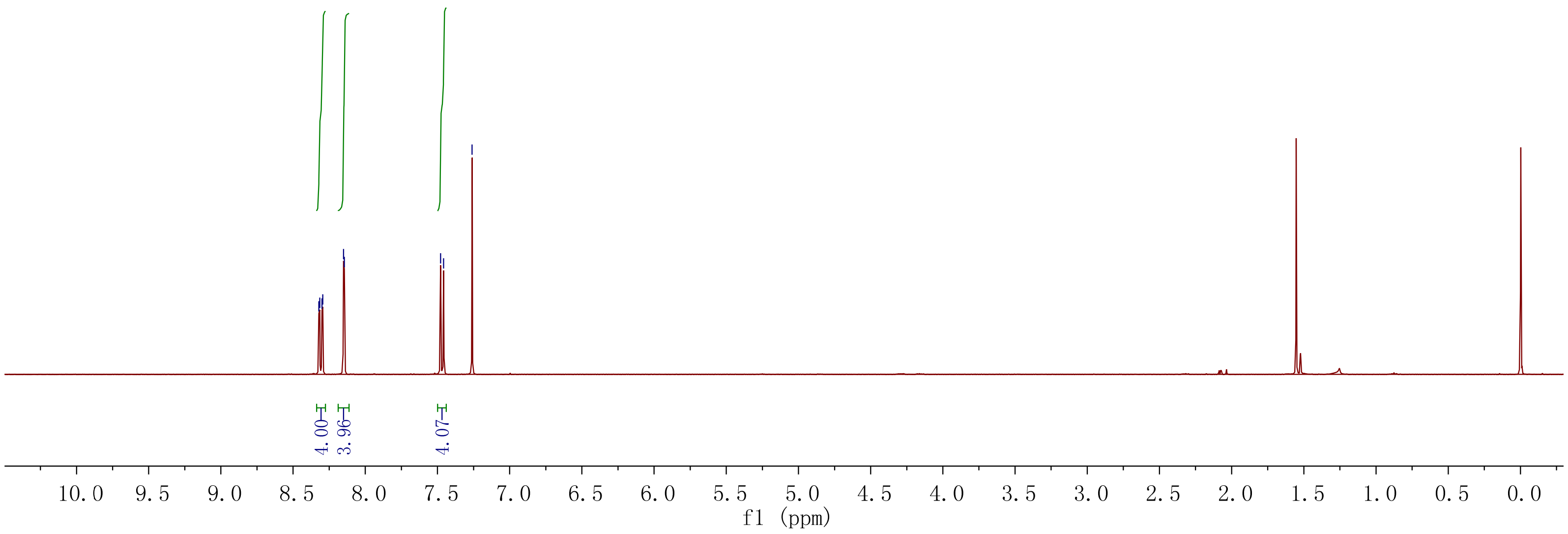
8.32
8.32
8.30
8.29
8.15
8.15
7.48
7.46
7.26
4.00
3.96
4.07
10.0
9.5
9.0
8.5
8.0
7.5
7.0
6.5
6.0
5.5
5.0
4.5
4.0
3.5
3.0
2.5
2.0
1.5
1.0
0.5
0.0
f1 (ppm)

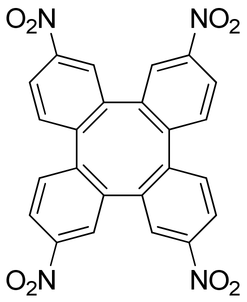
O2N
NO2
O2N
NO2

2hh

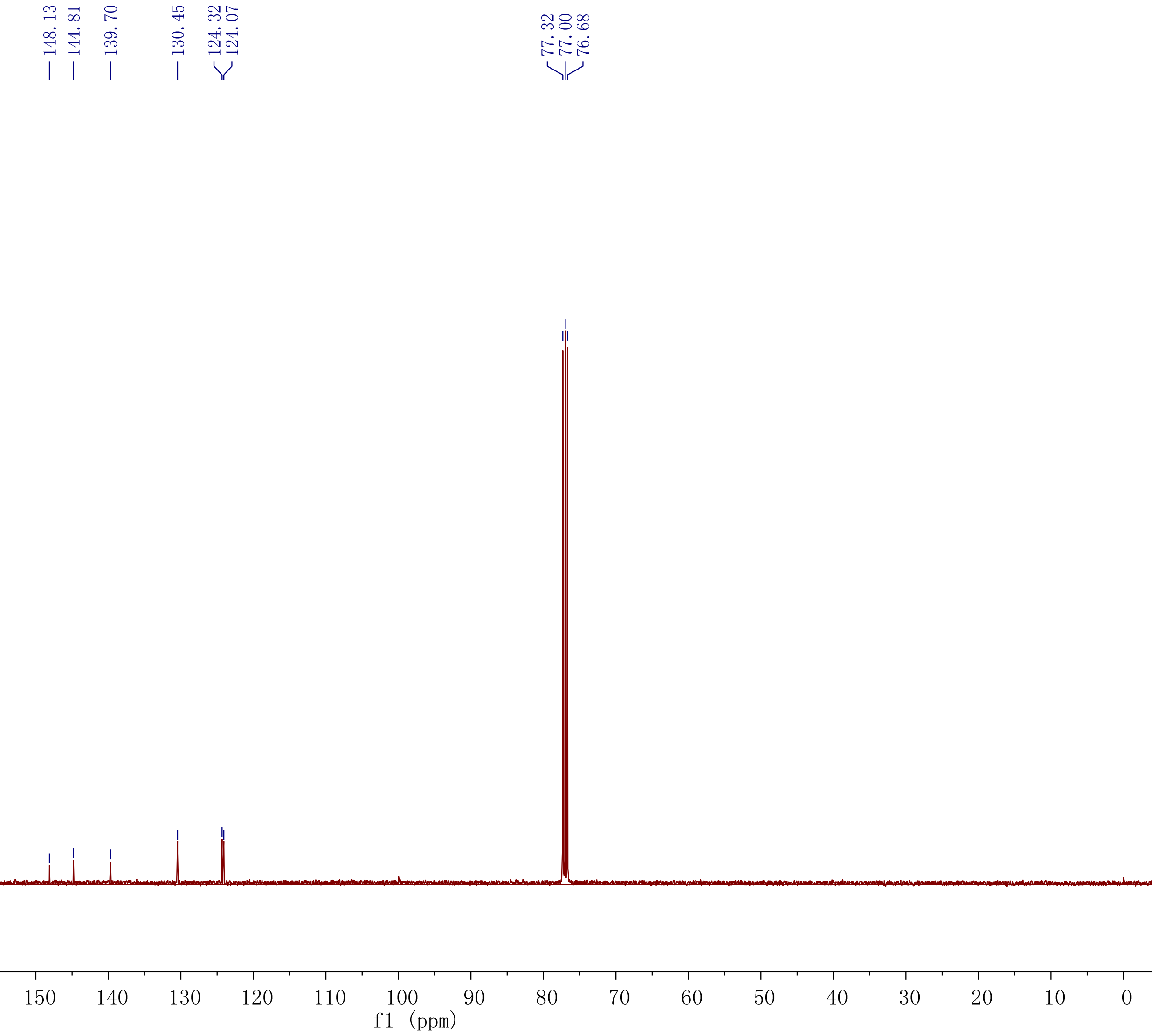
148.13
144.81
139.70
130.45
124.32
124.07
77.32
77.00
76.68
00
190
180
170
160
150
140
130
120
110
100
90
80
70
60
50
40
30
20
10
0
f1 (ppm)

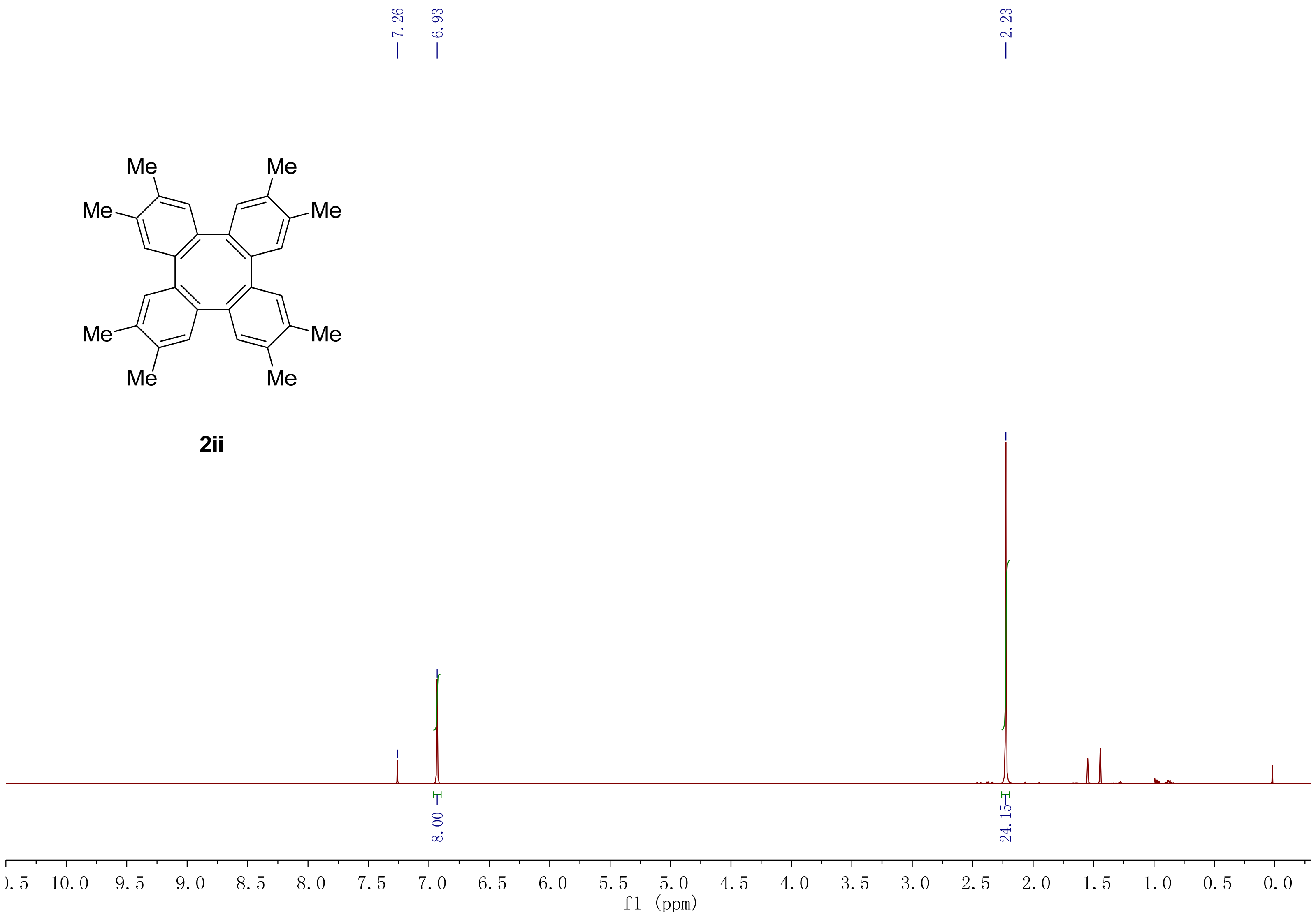
7.26
6.93
2.23
Me
2ii
8.00
24.15
f1 (ppm)

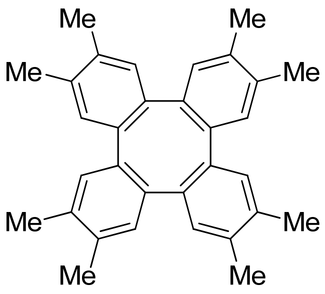
Me
Me
Me
Me
Me
Me
Me
Me

2ii

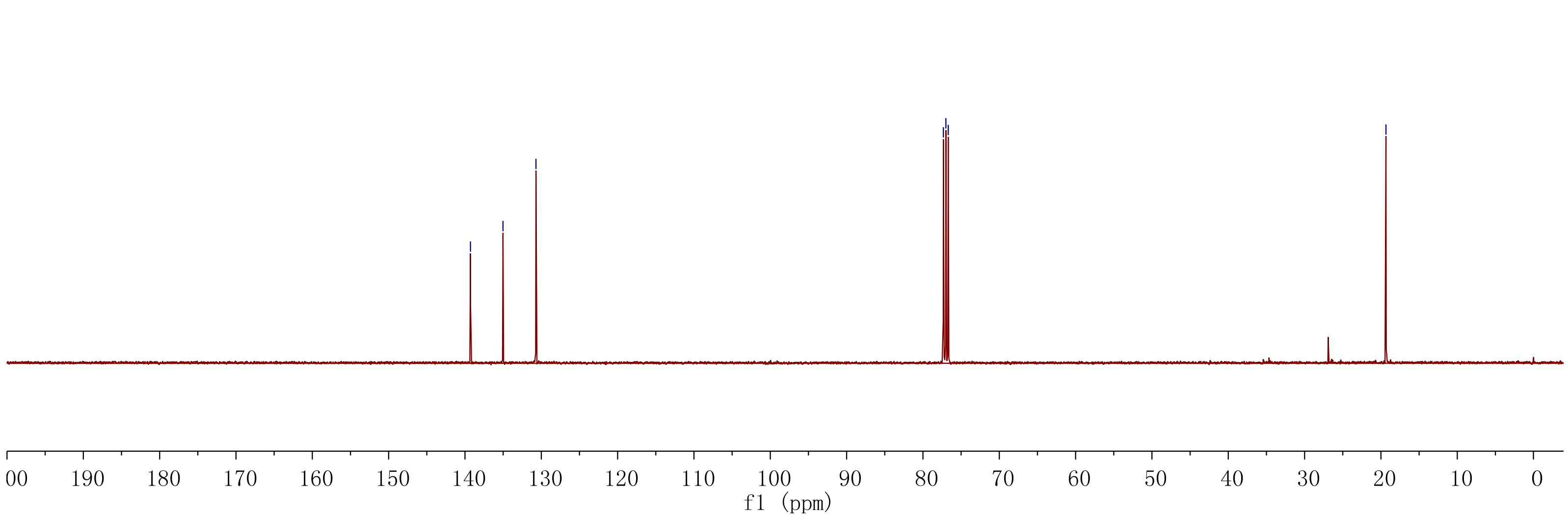
139.28
135.02
130.69
77.32
77.00
76.68
19.33
00
190
180
170
160
150
140
130
120
110
100
90
80
70
60
50
40
30
20
10
0
f1 (ppm)

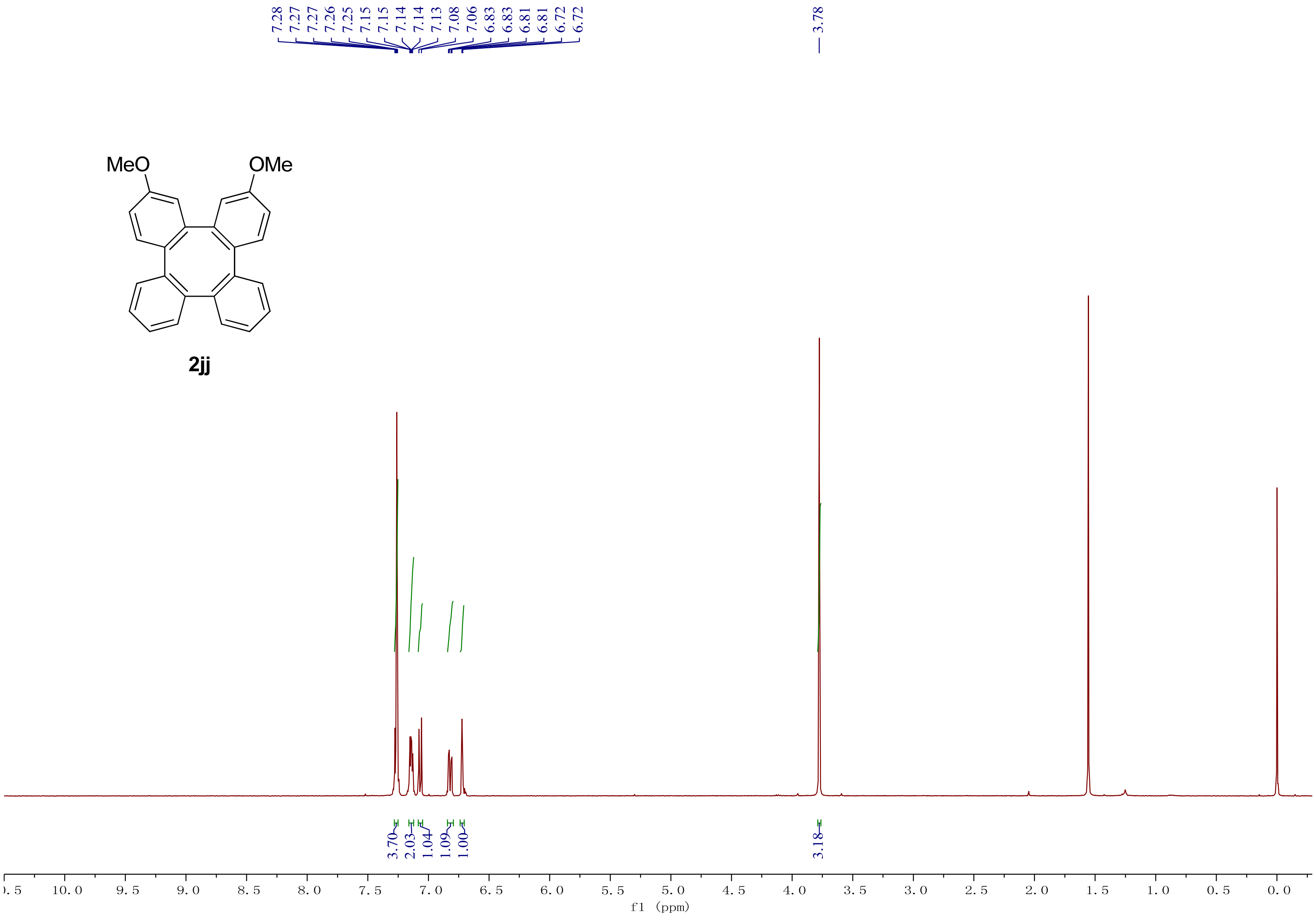
MeO
OMe
2jj
7.28
7.27
7.27
7.26
7.25
7.15
7.15
7.14
7.14
7.13
7.08
7.06
6.83
6.83
6.81
6.81
6.72
6.72
3.78
3.70
2.03
1.04
1.09
1.00
3.18
10.0
9.5
9.0
8.5
8.0
7.5
7.0
6.5
6.0
5.5
5.0
4.5
4.0
3.5
3.0
2.5
2.0
1.5
1.0
0.5
0.0
f1 (ppm)

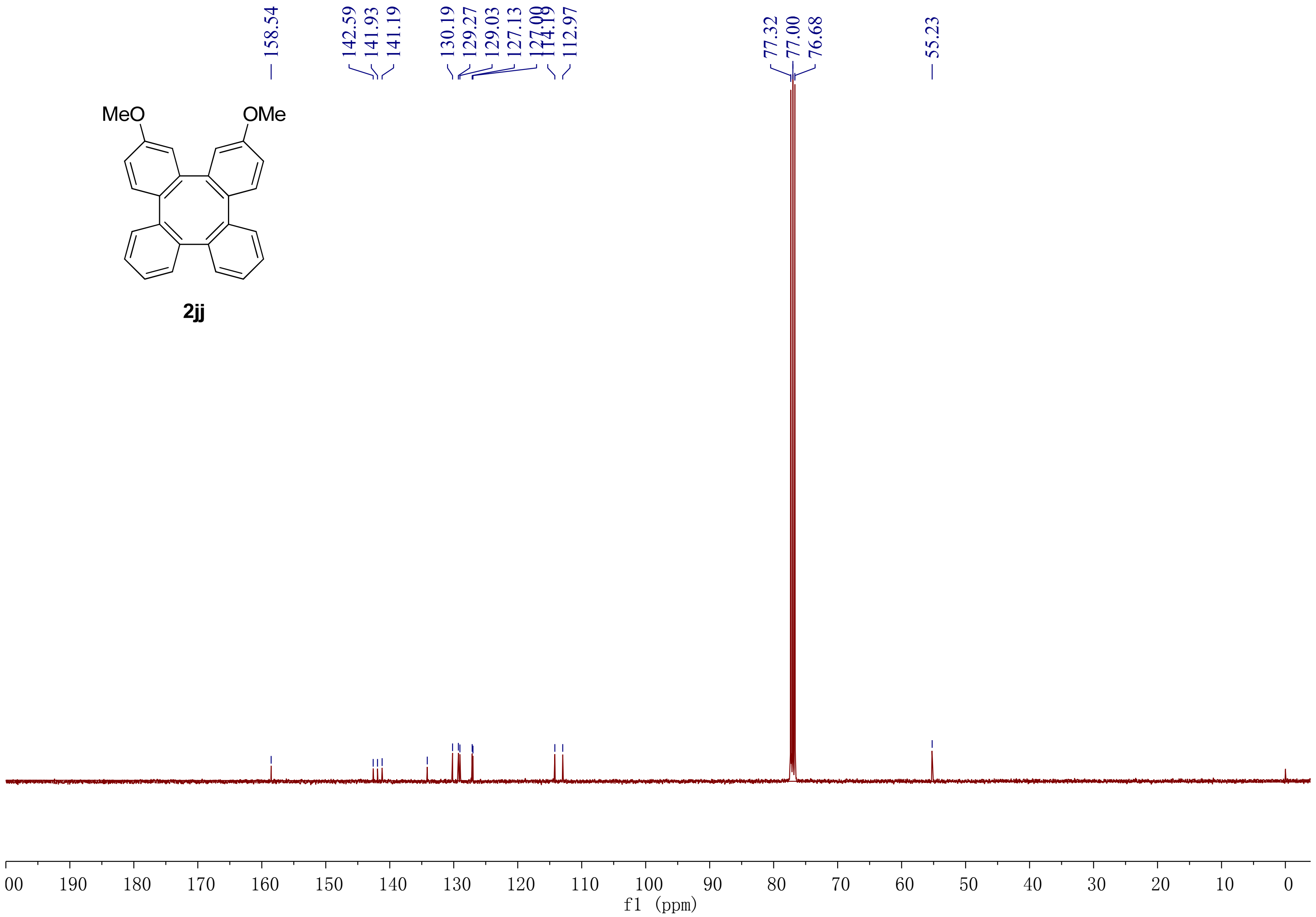
MeO
OMe
158.54
142.59
141.93
141.19
130.19
129.27
129.03
127.13
127.00
114.19
112.97
77.32
77.00
76.68
55.23
00
190
180
170
160
150
140
130
120
110
100
90
80
70
60
50
40
30
20
10
0
f1 (ppm)

2jj

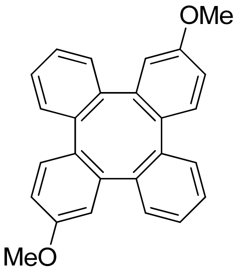
OMe
MeO

3jj

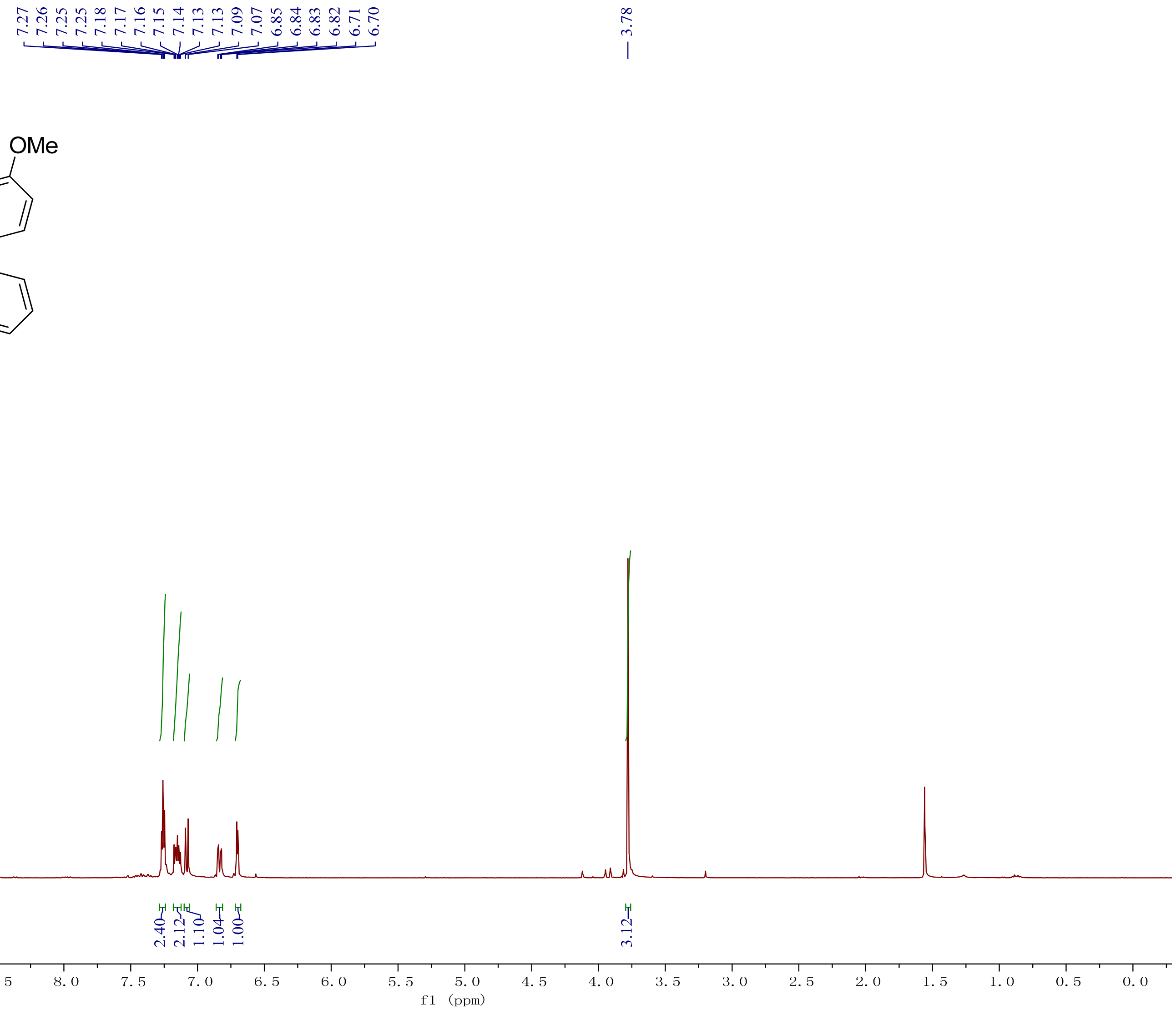
7.27
7.26
7.25
7.25
7.18
7.17
7.16
7.15
7.14
7.13
7.13
7.09
7.07
6.85
6.84
6.83
6.82
6.71
6.70
3.78
2.40
2.12
1.10
1.04
1.00
3.12
10.0
9.5
9.0
8.5
8.0
7.5
7.0
6.5
6.0
5.5
5.0
4.5
4.0
3.5
3.0
2.5
2.0
1.5
1.0
0.5
0.0
f1 (ppm)

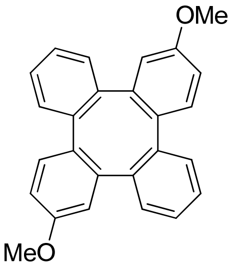
OMe
MeO
3jj

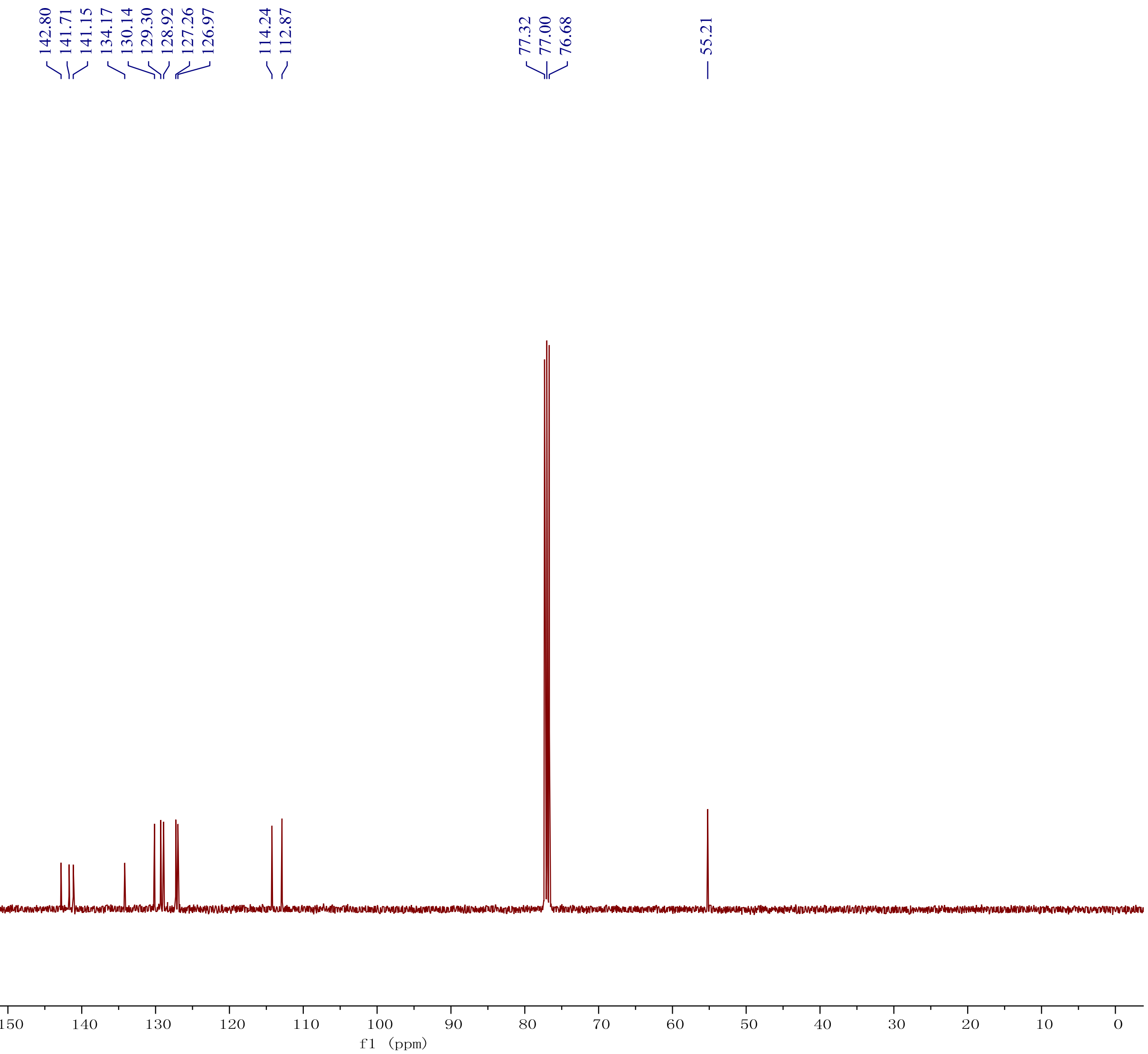
158.56
142.80
141.71
141.15
134.17
130.14
129.30
128.92
127.26
126.97
114.24
112.87
77.32
77.00
76.68
55.21
00
190
180
170
160
150
140
130
120
110
100
90
80
70
60
50
40
30
20
10
0
f1 (ppm)

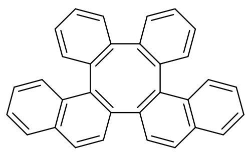

2kk

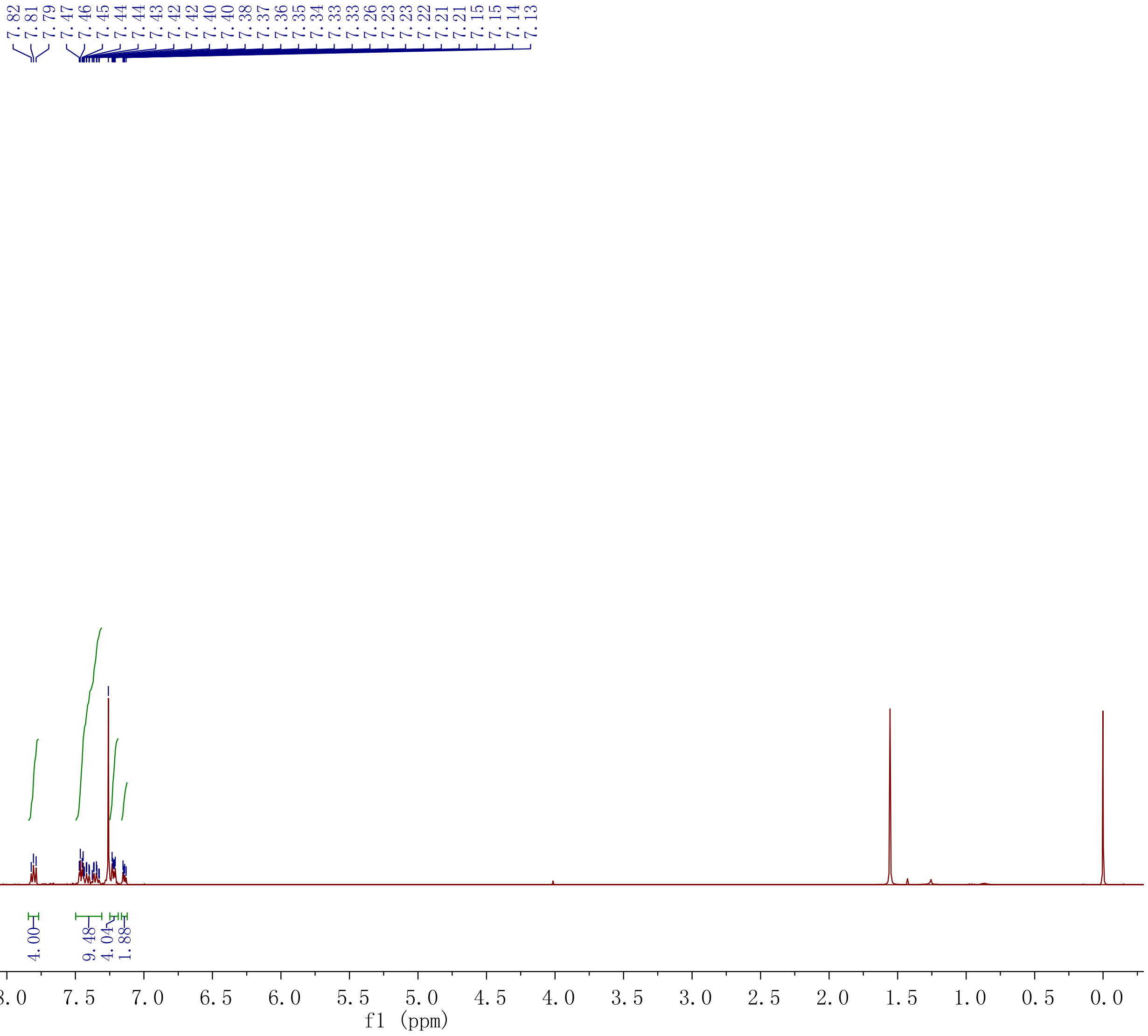
7.82
7.81
7.79
7.47
7.46
7.45
7.44
7.44
7.43
7.42
7.42
7.40
7.40
7.38
7.37
7.36
7.35
7.34
7.33
7.33
7.26
7.23
7.23
7.22
7.21
7.21
7.15
7.15
7.14
7.13
4.00
9.48
4.04
1.88
10.0
9.5
9.0
8.5
8.0
7.5
7.0
6.5
6.0
5.5
5.0
4.5
4.0
3.5
3.0
2.5
2.0
1.5
1.0
0.5
0.0
f1 (ppm)

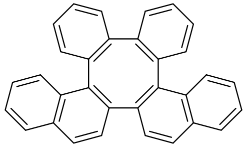

2kk

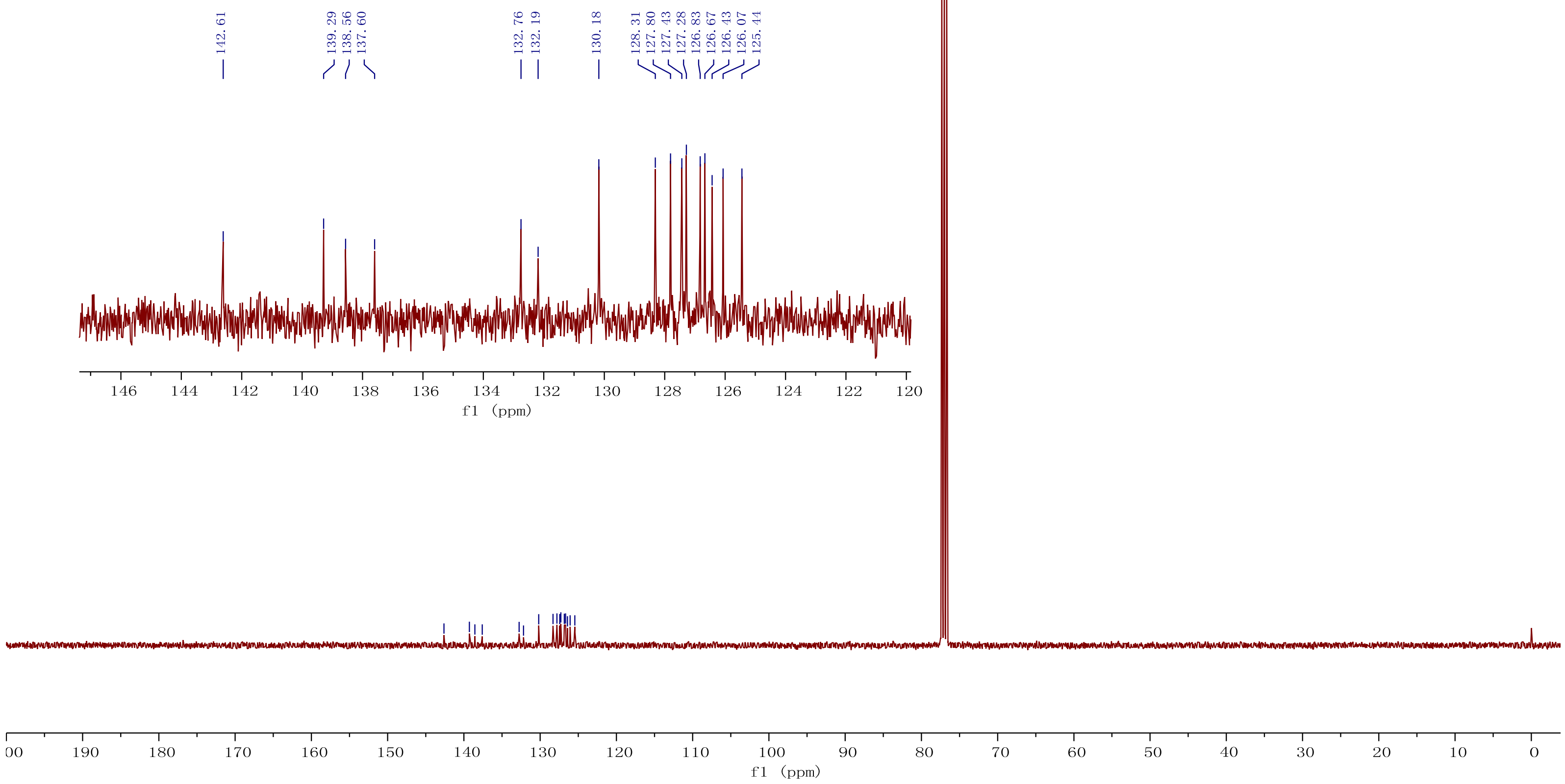
142.61
139.29
138.56
137.60
132.76
132.19
130.18
128.31
127.80
127.43
127.28
126.83
126.67
126.43
126.07
125.44
77.32 CDCl3
77.00 CDCl3
76.68 CDCl3
f1 (ppm)
f1 (ppm)

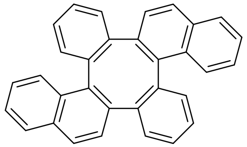

3kk

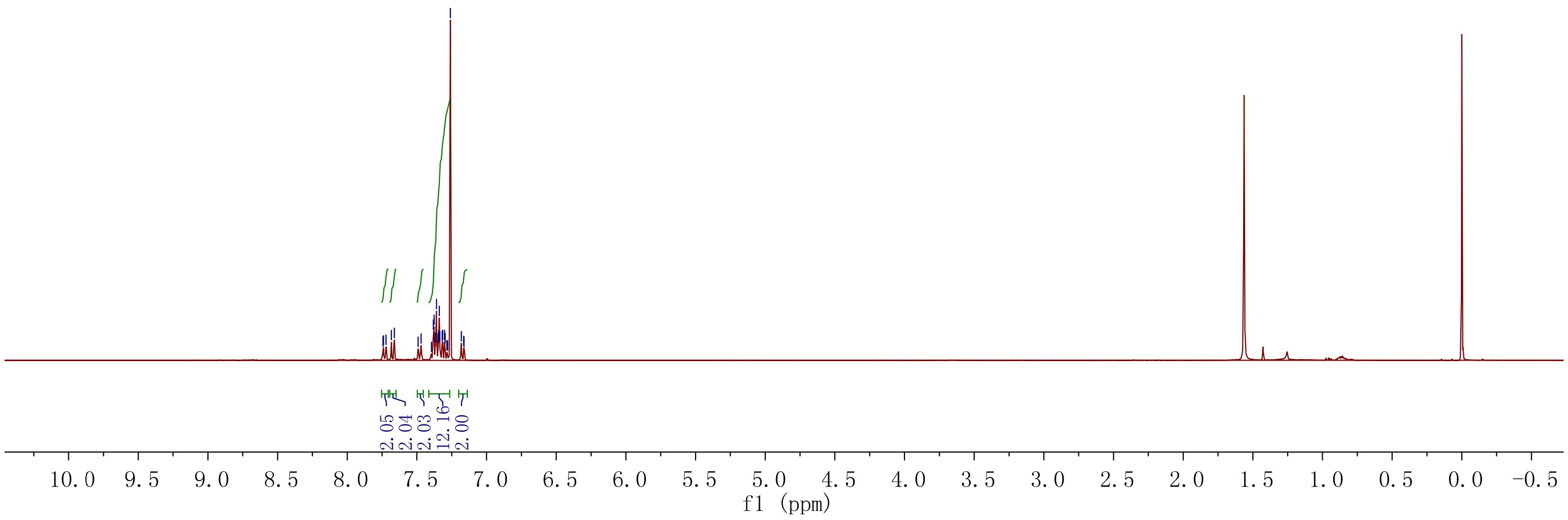
7.74
7.74
7.72
7.68
7.66
7.49
7.47
7.40
7.38
7.38
7.37
7.36
7.35
7.34
7.34
7.34
7.32
7.31
7.30
7.30
7.28
7.28
7.26
7.18
7.16
7.16
2.05
2.04
2.03
12.16
2.00
10.0 9.5 9.0 8.5 8.0 7.5 7.0 6.5 6.0 5.5 5.0 4.5 4.0 3.5 3.0 2.5 2.0 1.5 1.0 0.5 0.0 -0.5
f1 (ppm)

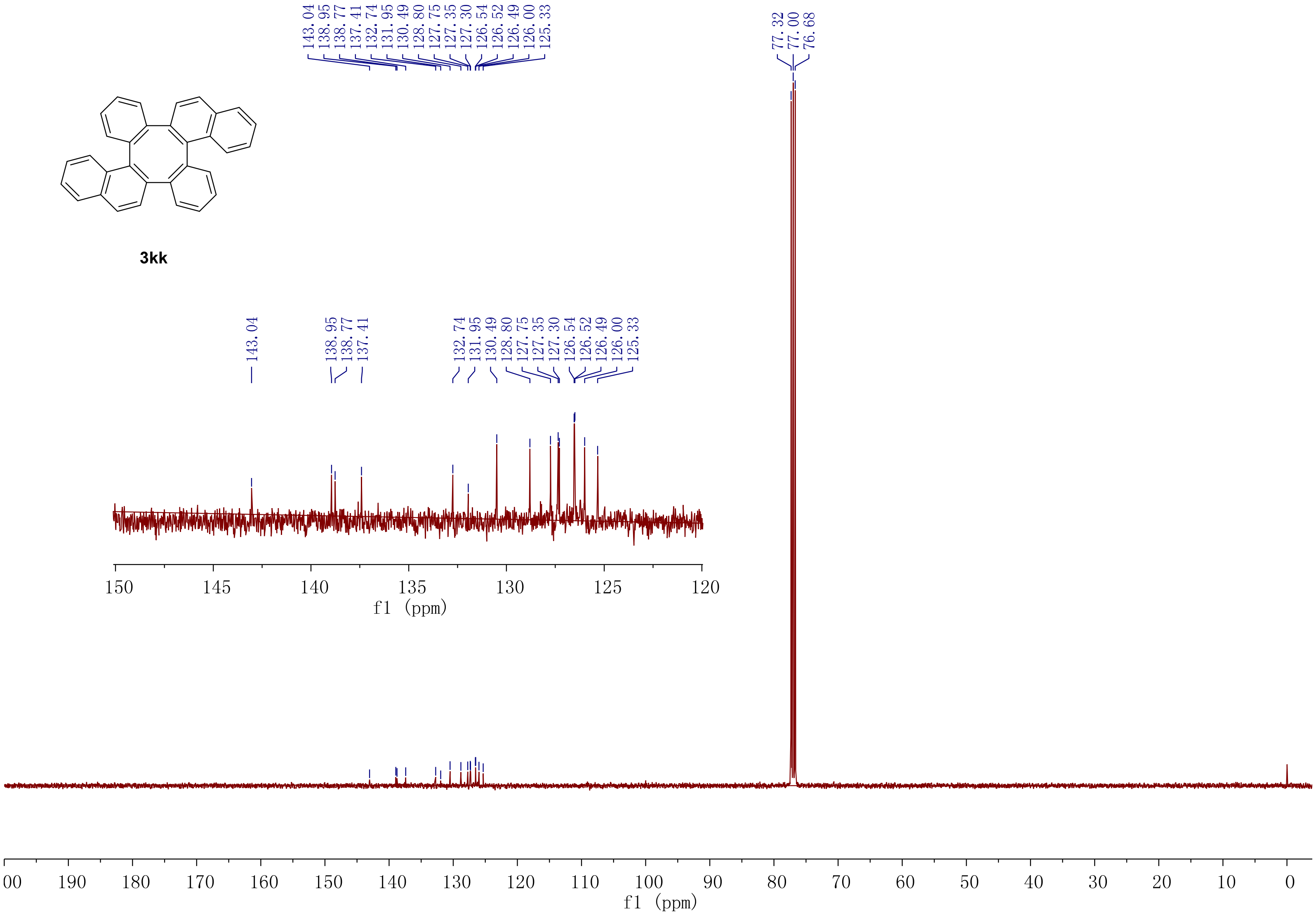
143.04
138.95
138.77
137.41
132.74
131.95
130.49
128.80
127.75
127.35
127.30
126.54
126.52
126.49
126.00
125.33
77.32
77.00
76.68
150
145
140
135
130
125
120
f1 (ppm)
00
190
180
170
160
150
140
130
120
110
100
90
80
70
60
50
40
30
20
10
0
f1 (ppm)

3kk

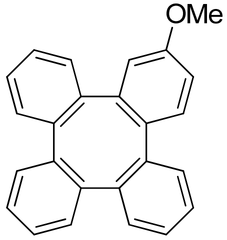
OMe

2aj

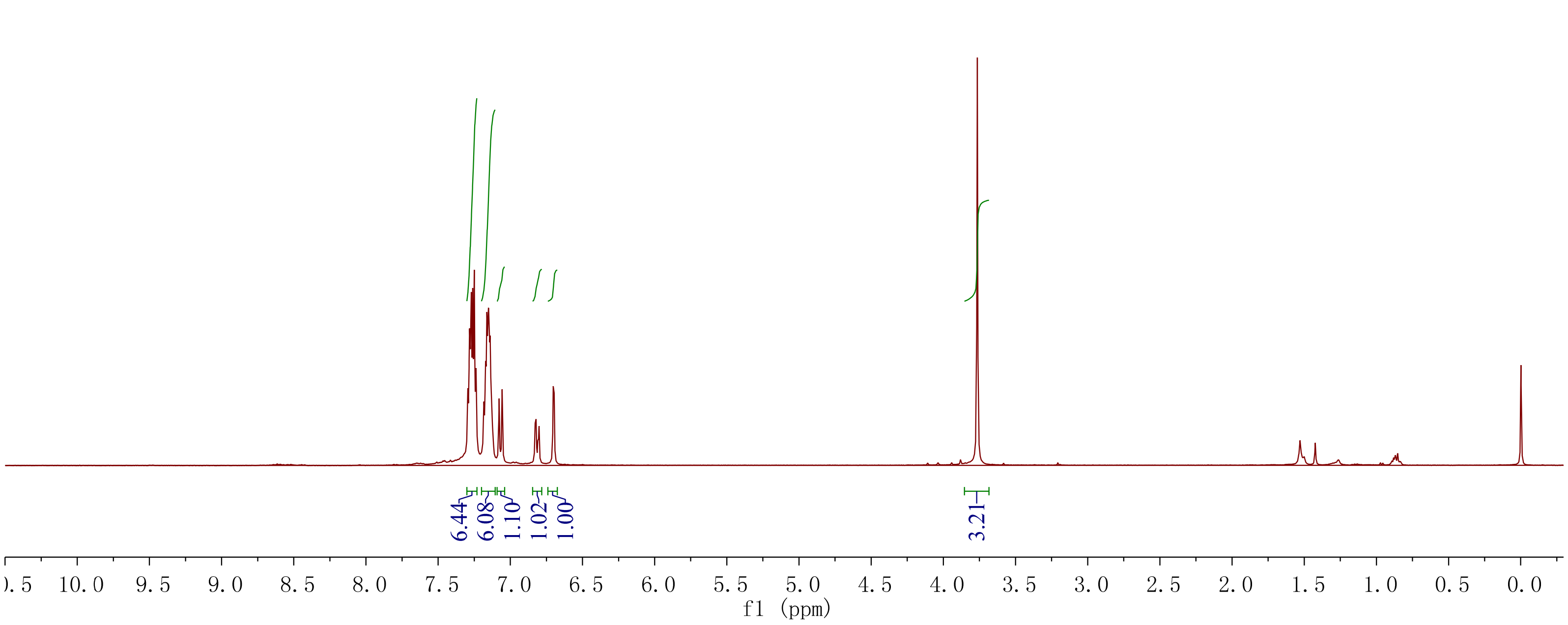
7.29
7.28
7.27
7.26
7.25
7.24
7.18
7.17
7.16
7.15
7.14
7.13
7.12
7.08
7.06
6.83
6.82
6.81
6.80
6.70
6.70
3.76
6.44
6.08
1.10
1.02
1.00
3.21
10.0 9.5 9.0 8.5 8.0 7.5 7.0 6.5 6.0 5.5 5.0 4.5 4.0 3.5 3.0 2.5 2.0 1.5 1.0 0.5 0.0
f1 (ppm)

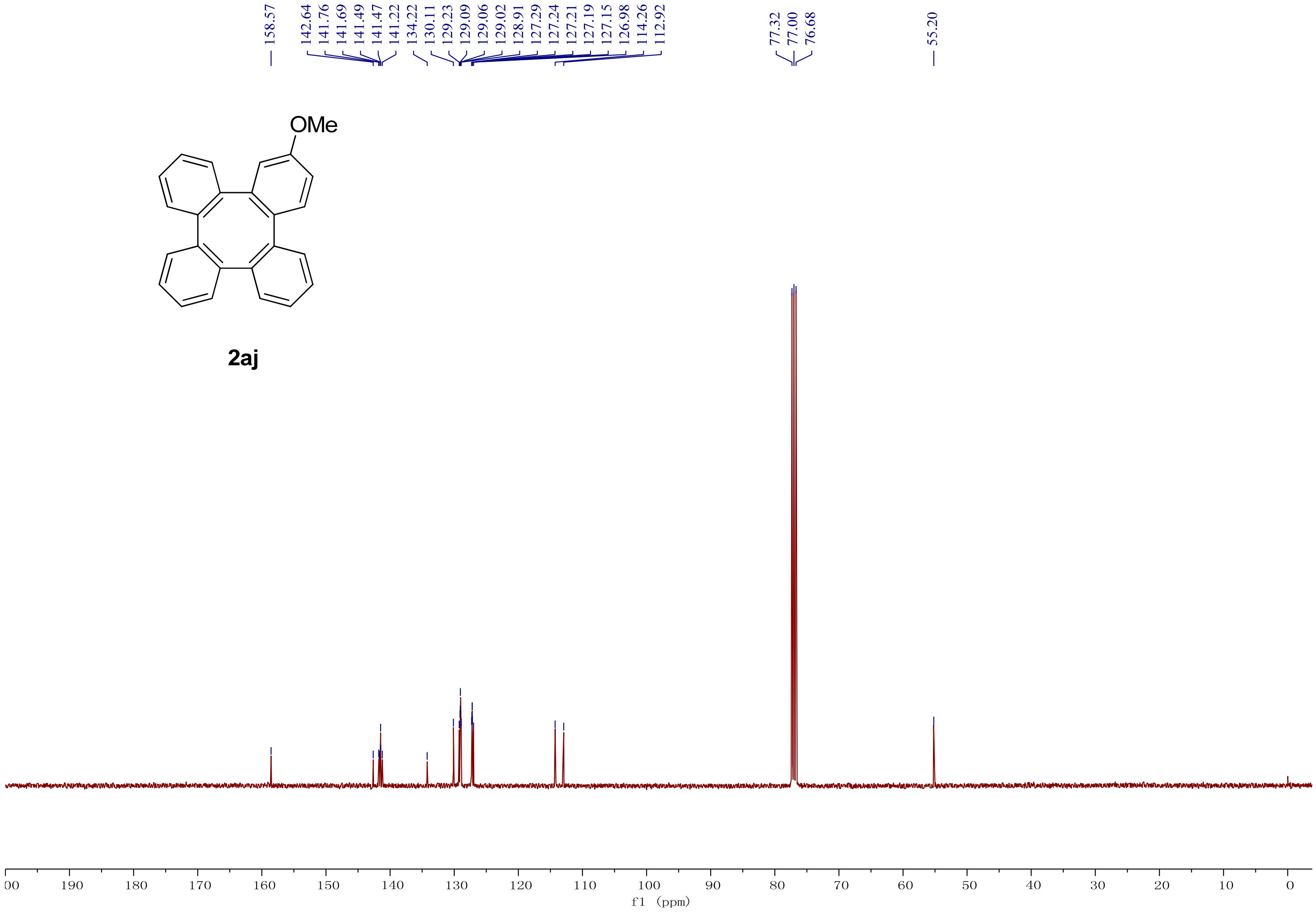
158.57
142.64
141.76
141.69
141.49
141.47
141.22
134.22
130.11
129.23
129.09
129.06
129.02
128.91
127.29
127.24
127.21
127.19
127.15
126.98
114.26
112.92
77.32
77.00
76.68
55.20
OMe
2aj
f1 (ppm)

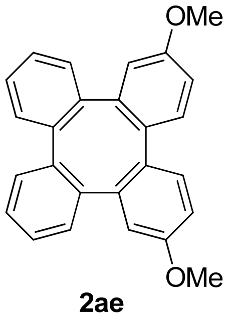

2ae

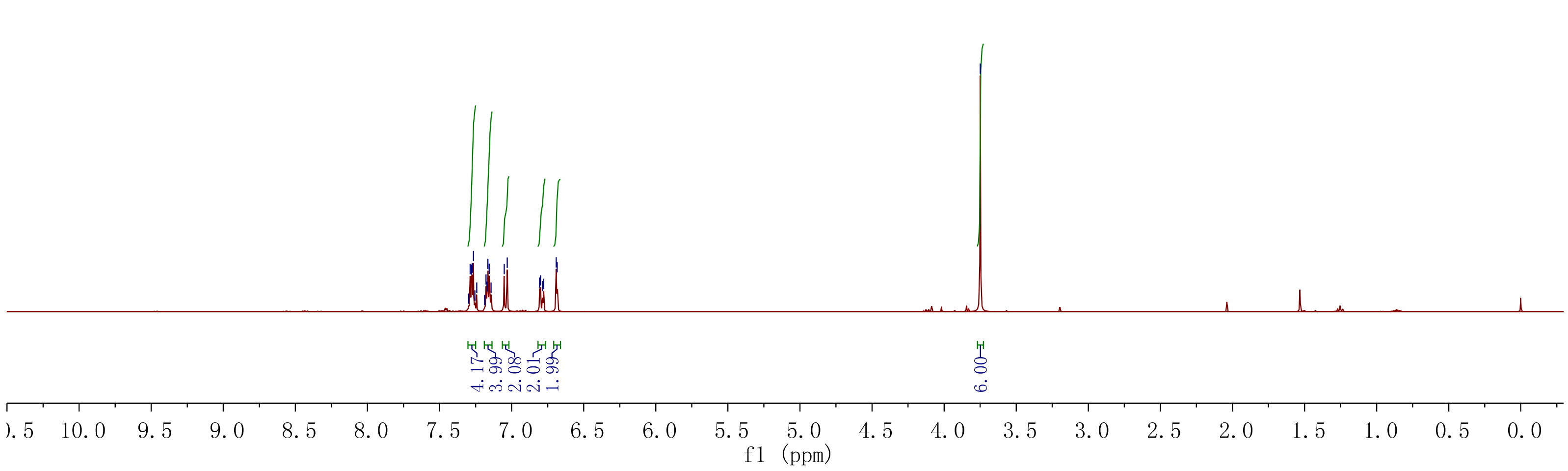

7.30
7.29
7.28
7.27
7.27
7.25
7.24
7.19
7.18
7.17
7.16
7.16
7.14
7.05
7.03
6.81
6.80
6.78
6.78
6.69
6.68
3.75
4.17
3.99
2.08
2.01
1.99
6.00
f1 (ppm)

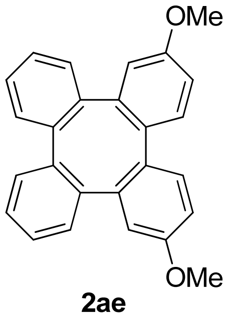
OMe
OMe

2ae

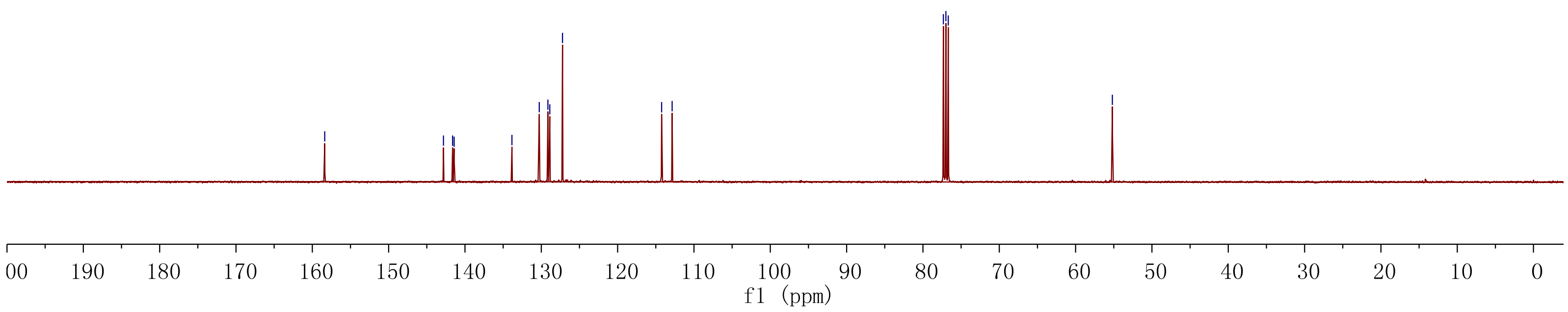
158.37
142.81
141.63
141.42
133.86
130.27
129.13
128.90
127.23
114.24
112.87
77.32
77.00
76.68
55.18
00
190
180
170
160
150
140
130
120
110
100
90
80
70
60
50
40
30
20
10
0
f1 (ppm)

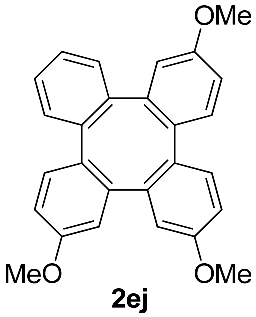
OMe
MeO
OMe

2ej

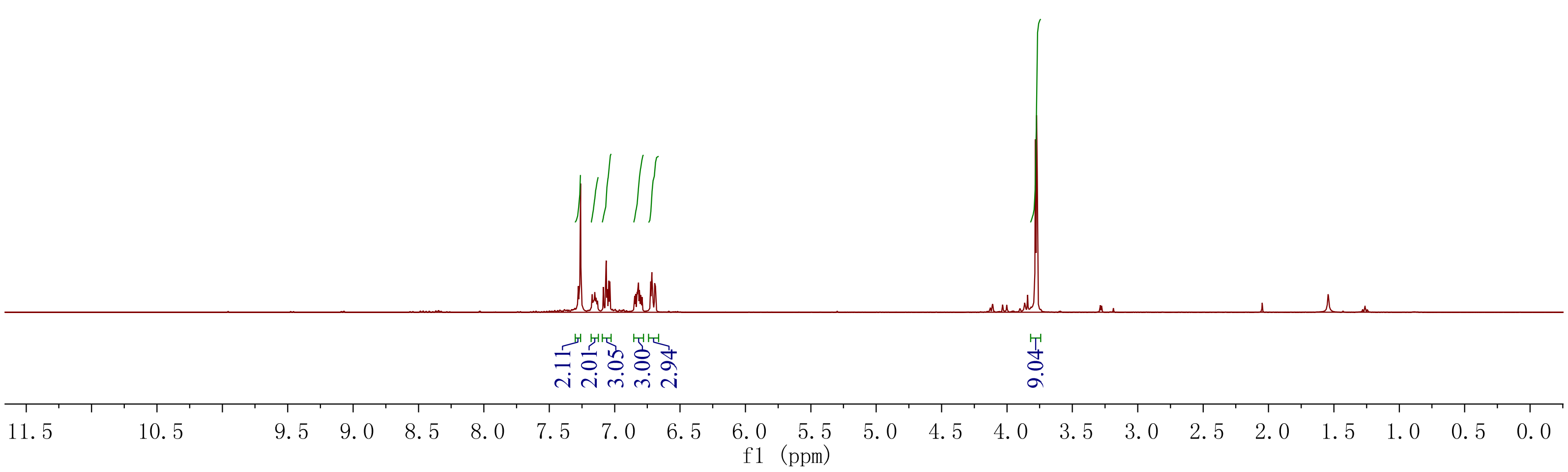
7.28
7.27
7.26
7.26
7.17
7.17
7.16
7.15
7.14
7.14
7.13
7.09
7.06
7.06
7.04
7.04
6.85
6.84
6.83
6.83
6.82
6.82
6.81
6.80
6.80
6.79
6.72
6.72
6.71
6.69
6.69
3.78
3.77
3.77
2.11
2.01
3.05
3.00
2.94
9.04
11.5
10.5
9.5
9.0
8.5
8.0
7.5
7.0
6.5
6.0
5.5
5.0
4.5
4.0
3.5
3.0
2.5
2.0
1.5
1.0
0.5
0.0
f1 (ppm)

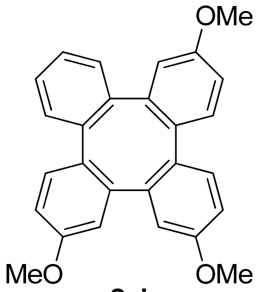
OMe
MeO
OMe

2ej

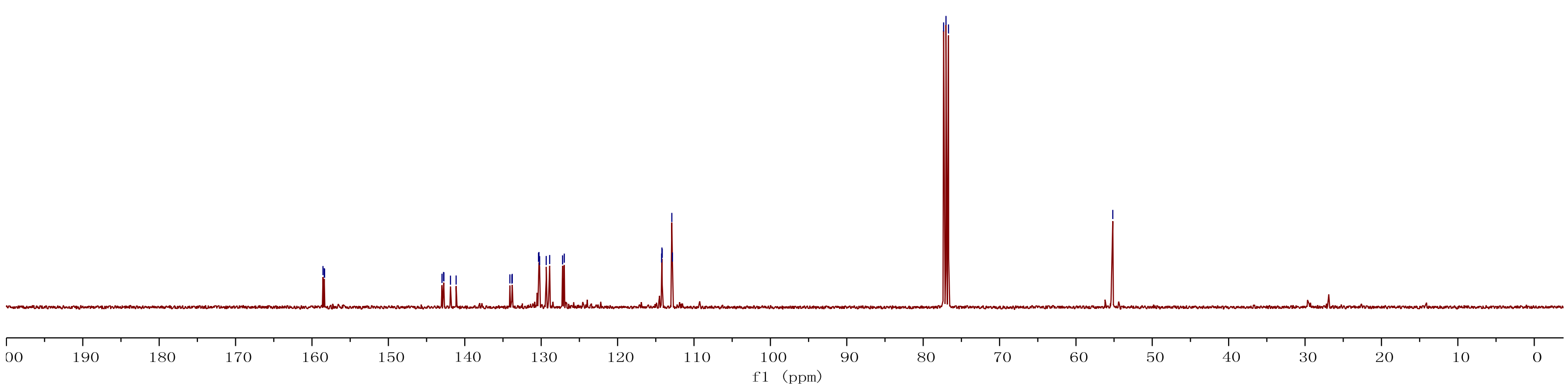
158.56
158.40
158.37
142.99
142.77
142.75
141.88
141.13
134.10
133.83
133.79
130.34
130.29
130.21
129.34
128.90
127.19
126.98
114.25
114.19
114.16
112.90
112.82
77.32
77.00
76.68
55.18
00 190 180 170 160 150 140 130 120 110 100 90 80 70 60 50 40 30 20 10 0
f1 (ppm)

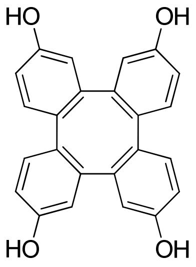
HO
OH
HO
OH

3ee

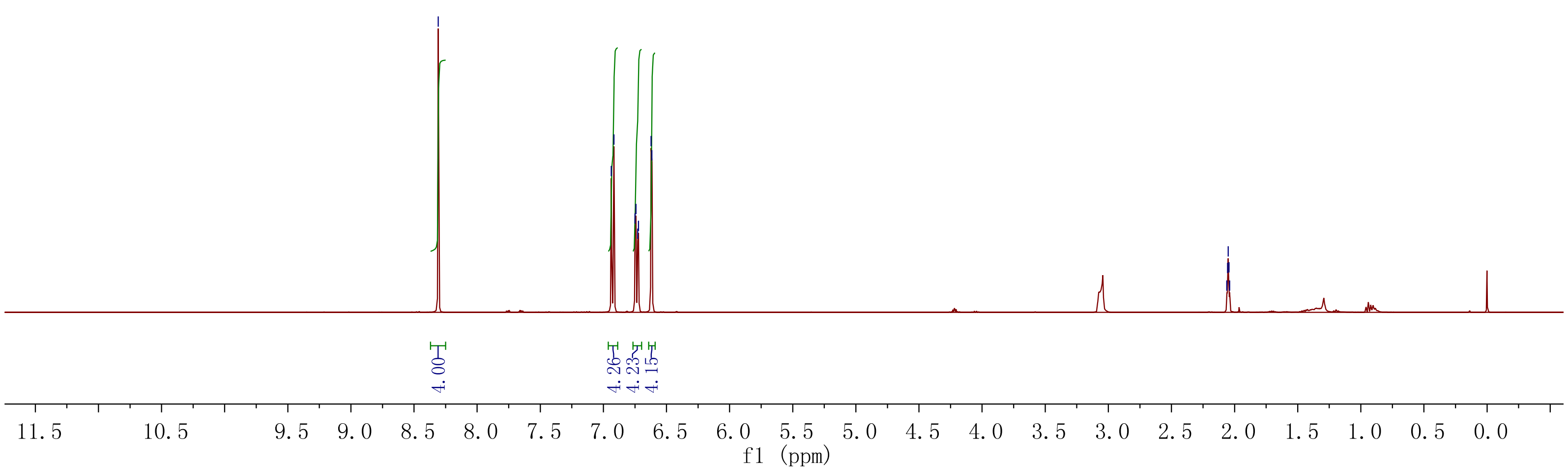
8.31
6.94
6.92
6.75
6.74
6.73
6.72
6.62
6.62
2.06
2.06
2.05
2.04
2.04
4.00
4.26
4.23
4.15
11.5
10.5
9.5
9.0
8.5
8.0
7.5
7.0
6.5
6.0
5.5
5.0
4.5
4.0
3.5
3.0
2.5
2.0
1.5
1.0
0.5
0.0
f1 (ppm)

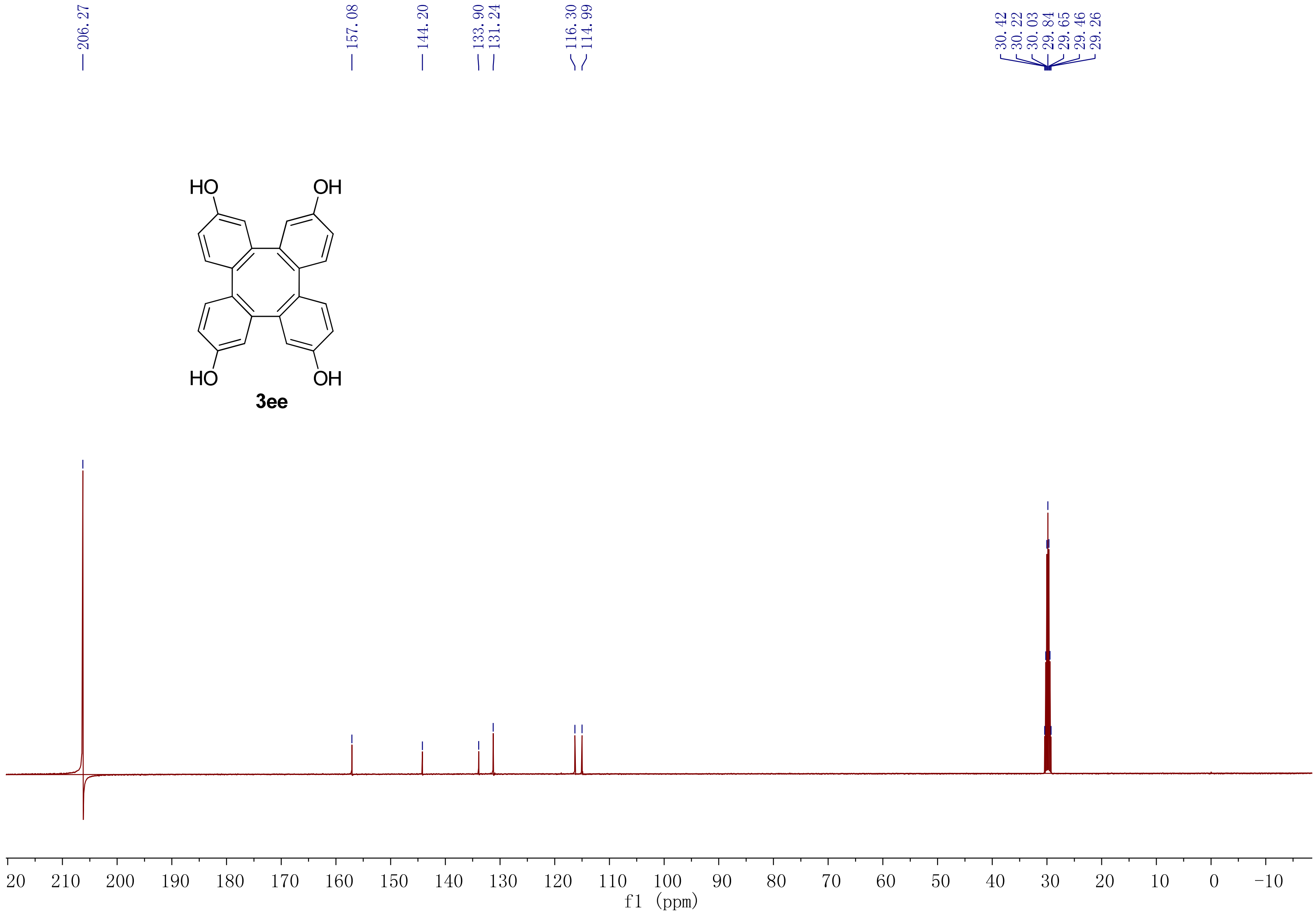
206.27
157.08
144.20
133.90
131.24
116.30
114.99
30.42
30.22
30.03
29.84
29.65
29.46
29.26
20 210 200 190 180 170 160 150 140 130 120 110 100 90 80 70 60 50 40 30 20 10 0 −10
f1 (ppm)
HO
OH
HO
OH

3ee

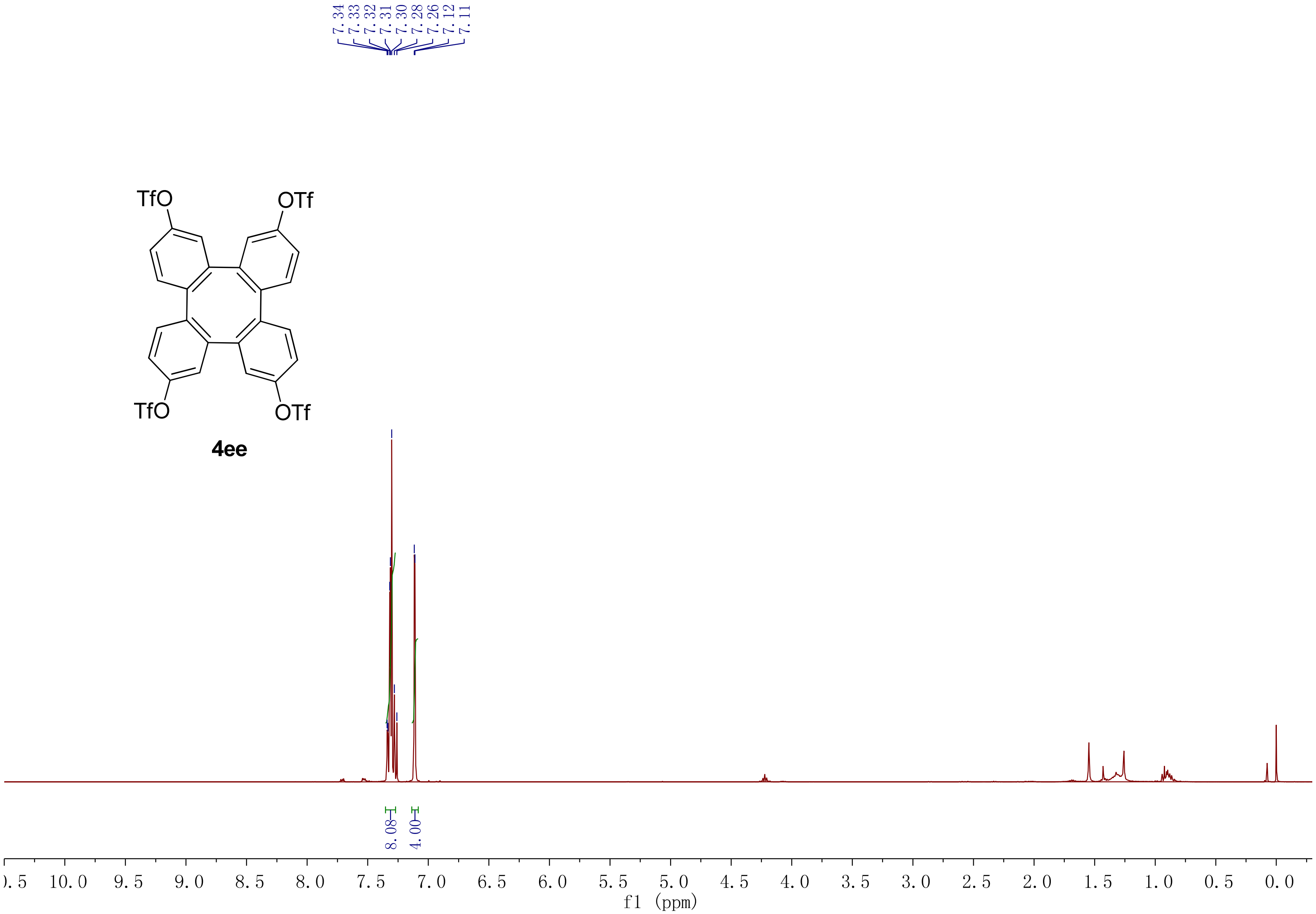
7.34
7.33
7.32
7.31
7.30
7.28
7.26
7.12
7.11
TfO
OTf
TfO
OTf
4ee
8.08
4.00
0.5
10.0
9.5
9.0
8.5
8.0
7.5
7.0
6.5
6.0
5.5
5.0
4.5
4.0
3.5
3.0
2.5
2.0
1.5
1.0
0.5
0.0
f1 (ppm)

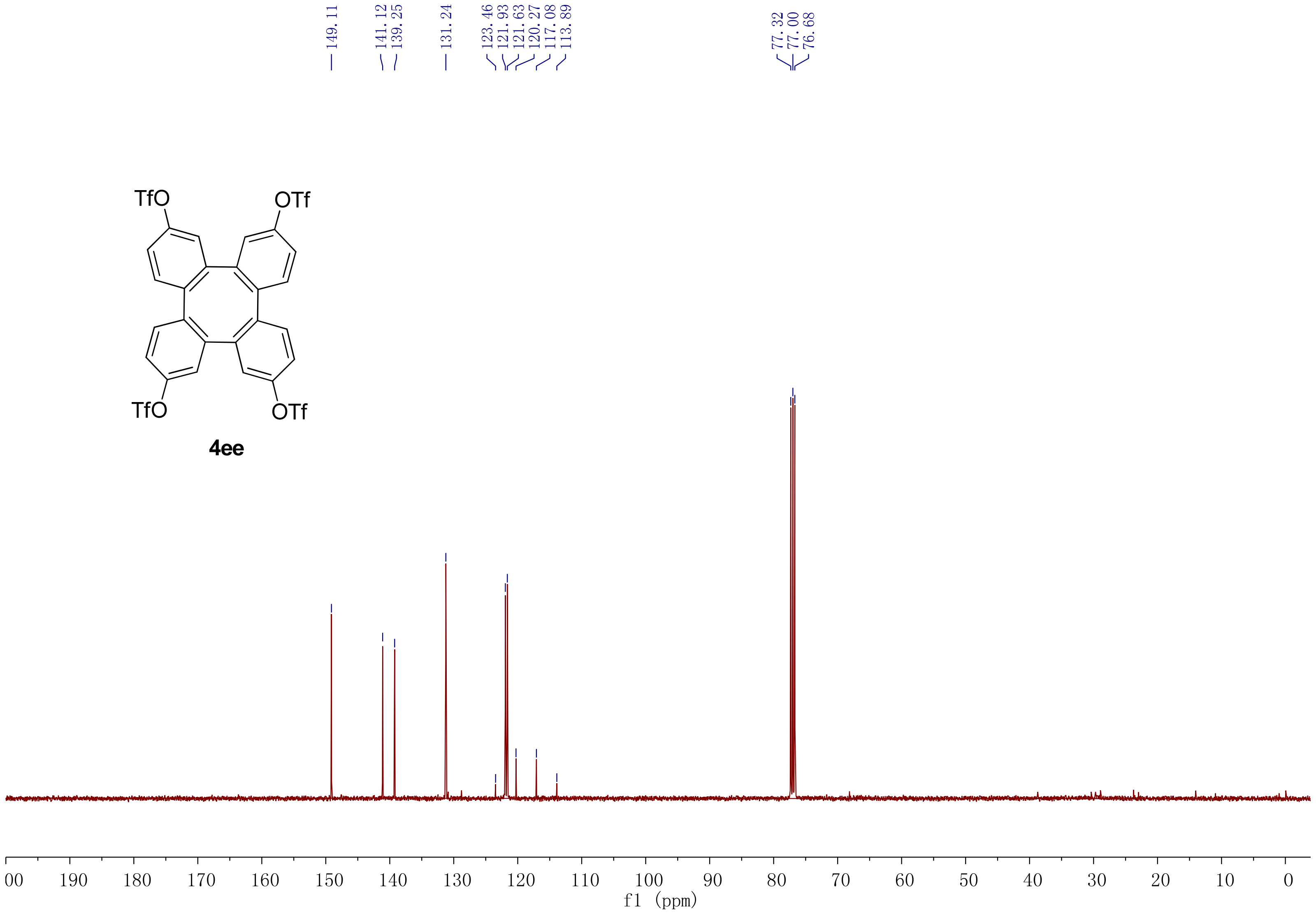
TfO
OTf
TfO
OTf
4ee
149.11
141.12
139.25
131.24
123.46
121.93
121.63
120.27
117.08
113.89
77.32
77.00
76.68
00
190
180
170
160
150
140
130
120
110
100
90
80
70
60
50
40
30
20
10
0
f1 (ppm)

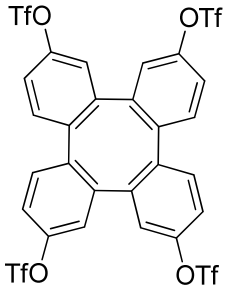
TfO
OTf
TfO
OTf

4ee

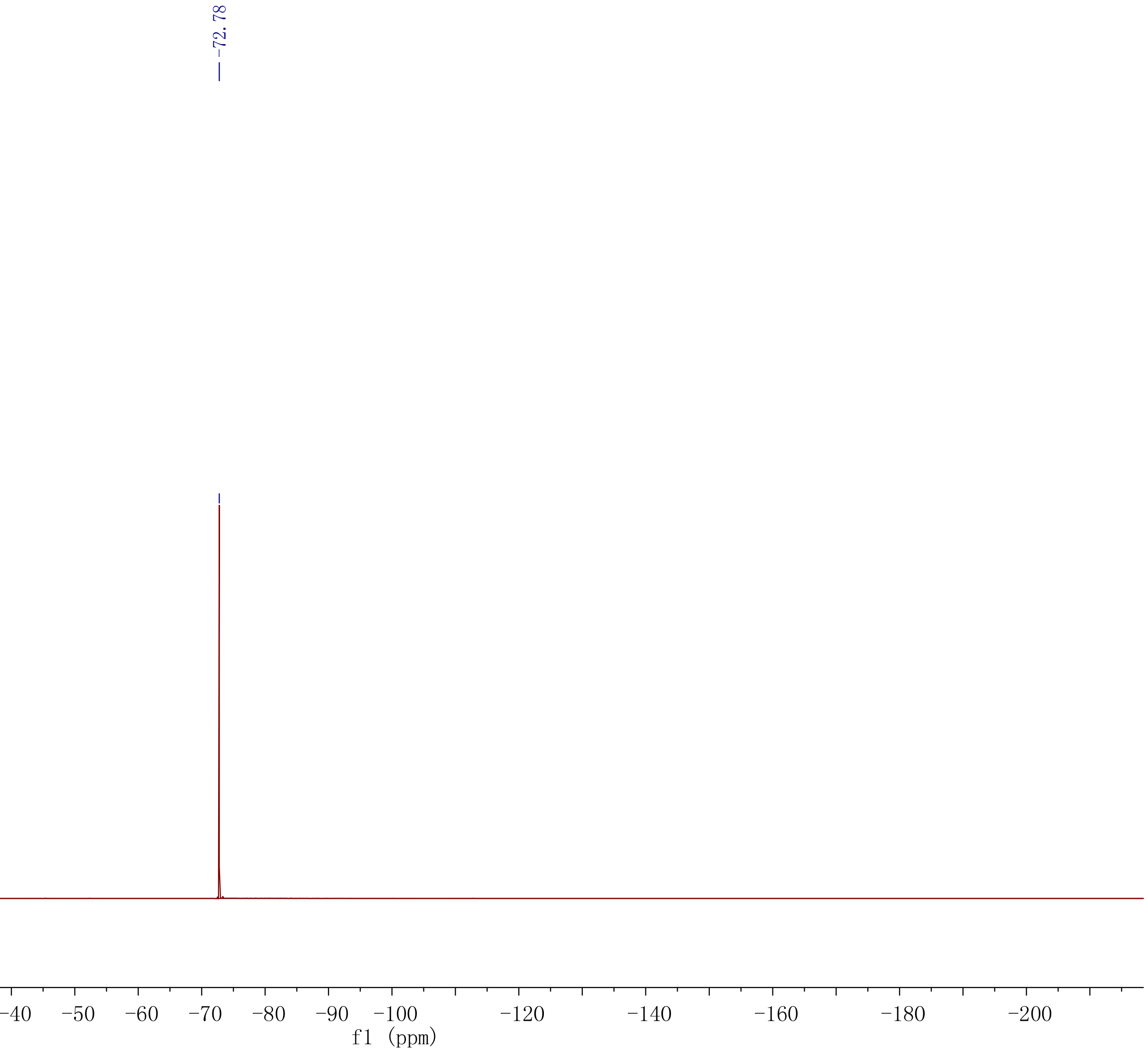
−72.78
10 0 −10 −20 −30 −40 −50 −60 −70 −80 −90 −100 −120 −140 −160 −180 −200
f1 (ppm)

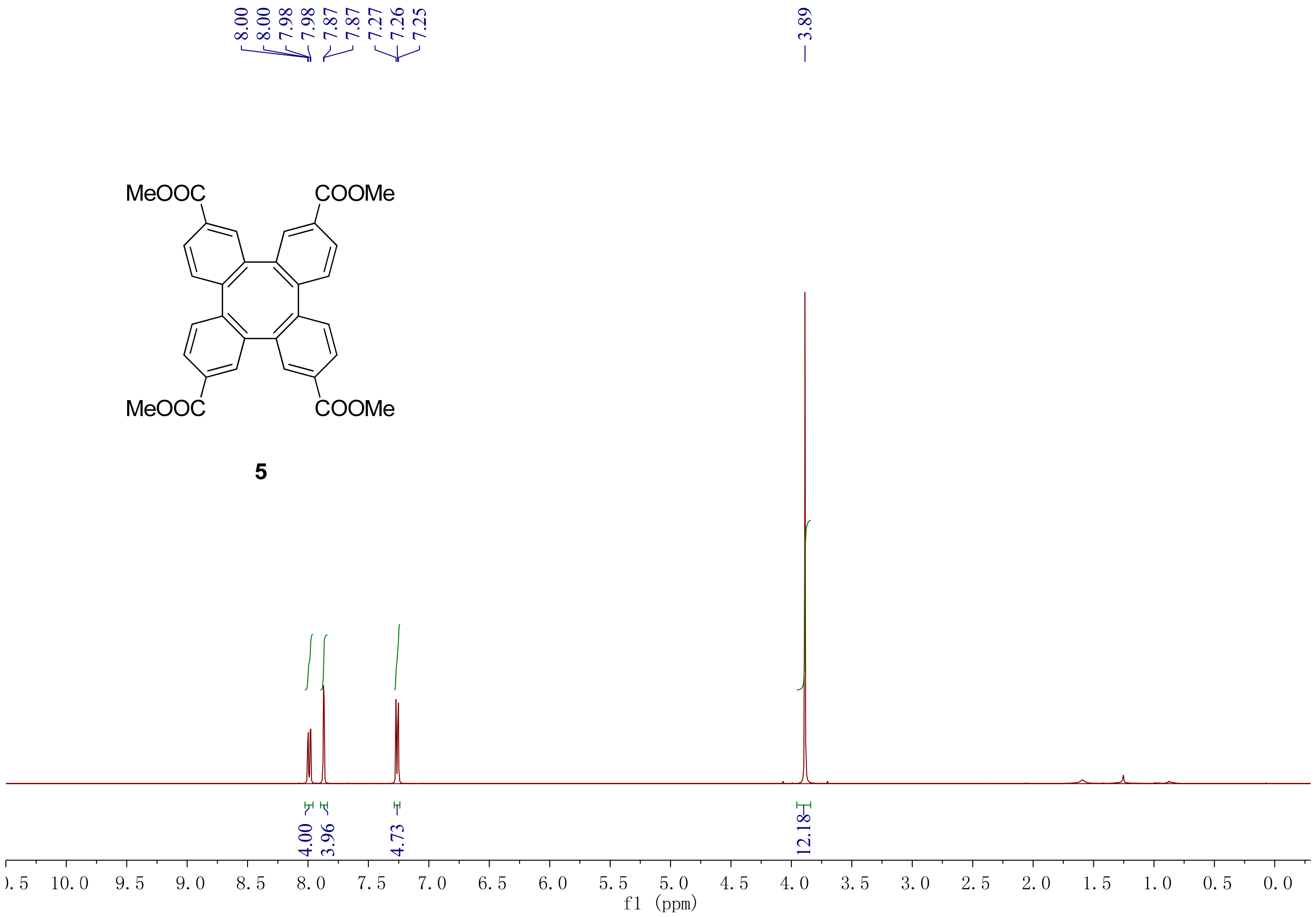
8.00
8.00
7.98
7.98
7.87
7.87
7.27
7.26
7.25
3.89
MeOOC
COOMe
MeOOC
COOMe
5
4.00
3.96
4.73
12.18
10.0 9.5 9.0 8.5 8.0 7.5 7.0 6.5 6.0 5.5 5.0 4.5 4.0 3.5 3.0 2.5 2.0 1.5 1.0 0.5 0.0
f1 (ppm)

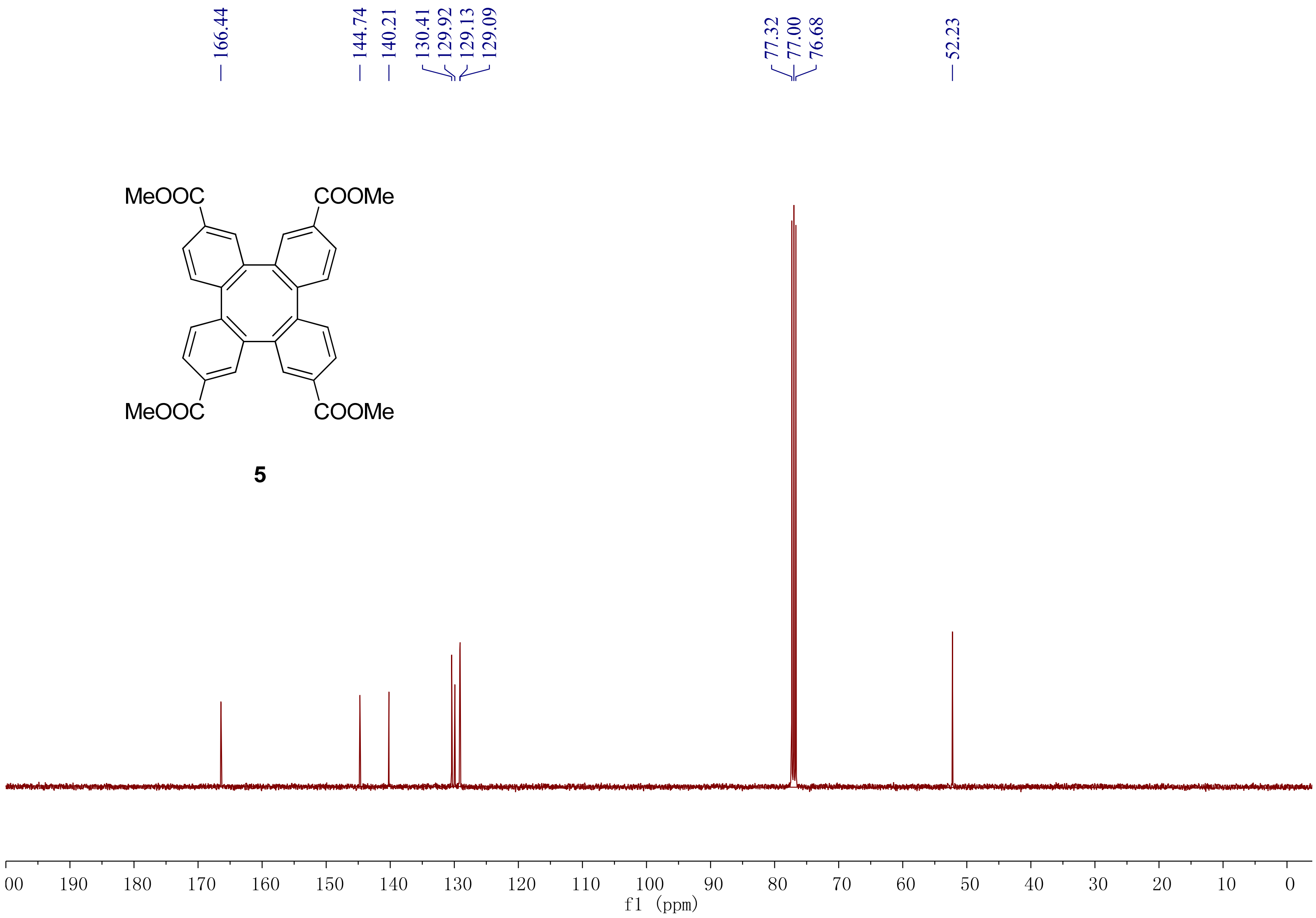
166.44
144.74
140.21
130.41
129.92
129.13
129.09
77.32
77.00
76.68
52.23
MeOOC
COOMe
MeOOC
COOMe
5
00
190
180
170
160
150
140
130
120
110
100
90
80
70
60
50
40
30
20
10
0
f1 (ppm)

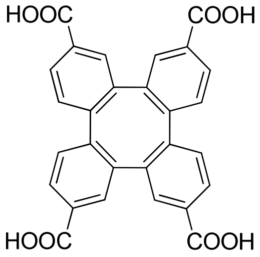
HOOC
COOH
HOOC
COOH

6

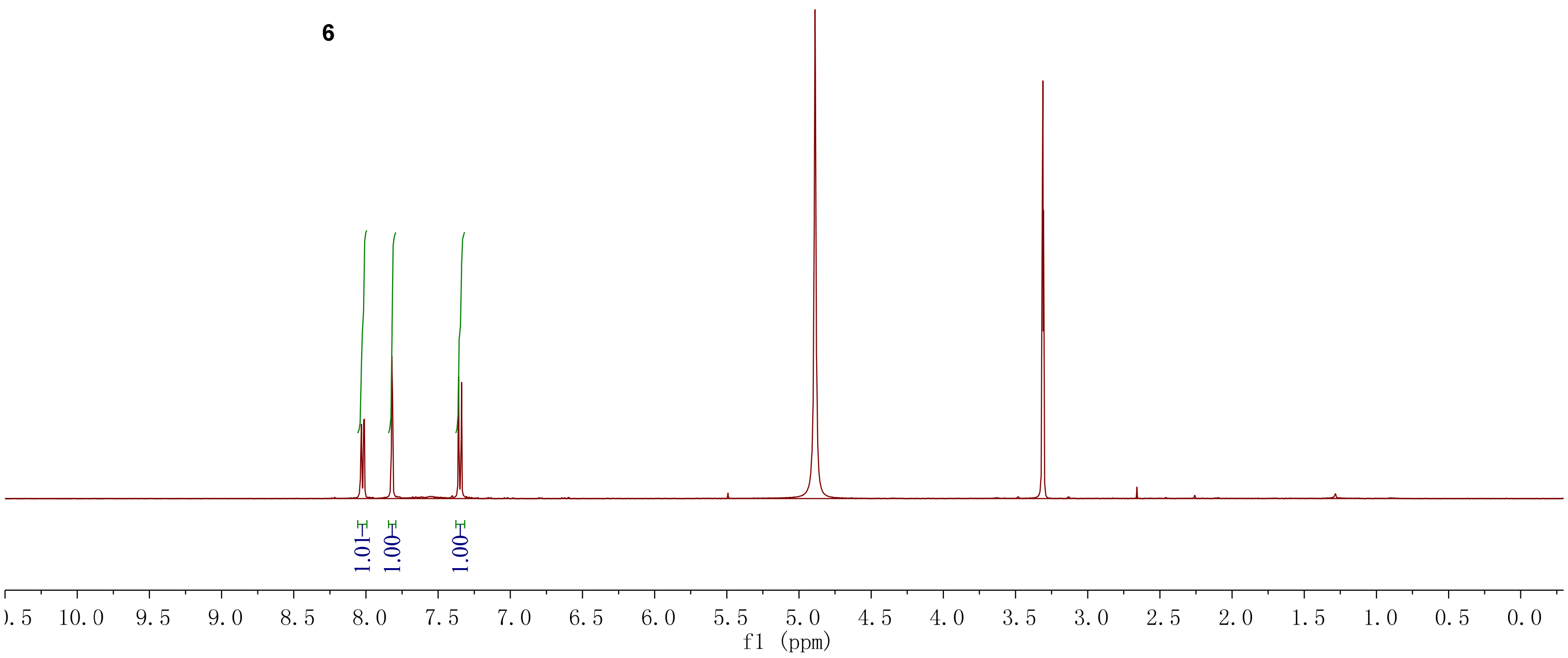
8.04
8.03
8.02
8.01
7.82
7.82
7.36
7.34
3.32
3.31
3.31
3.31
3.30
1.01
1.00
1.00
10.0
9.5
9.0
8.5
8.0
7.5
7.0
6.5
6.0
5.5
5.0
4.5
4.0
3.5
3.0
2.5
2.0
1.5
1.0
0.5
0.0
f1 (ppm)

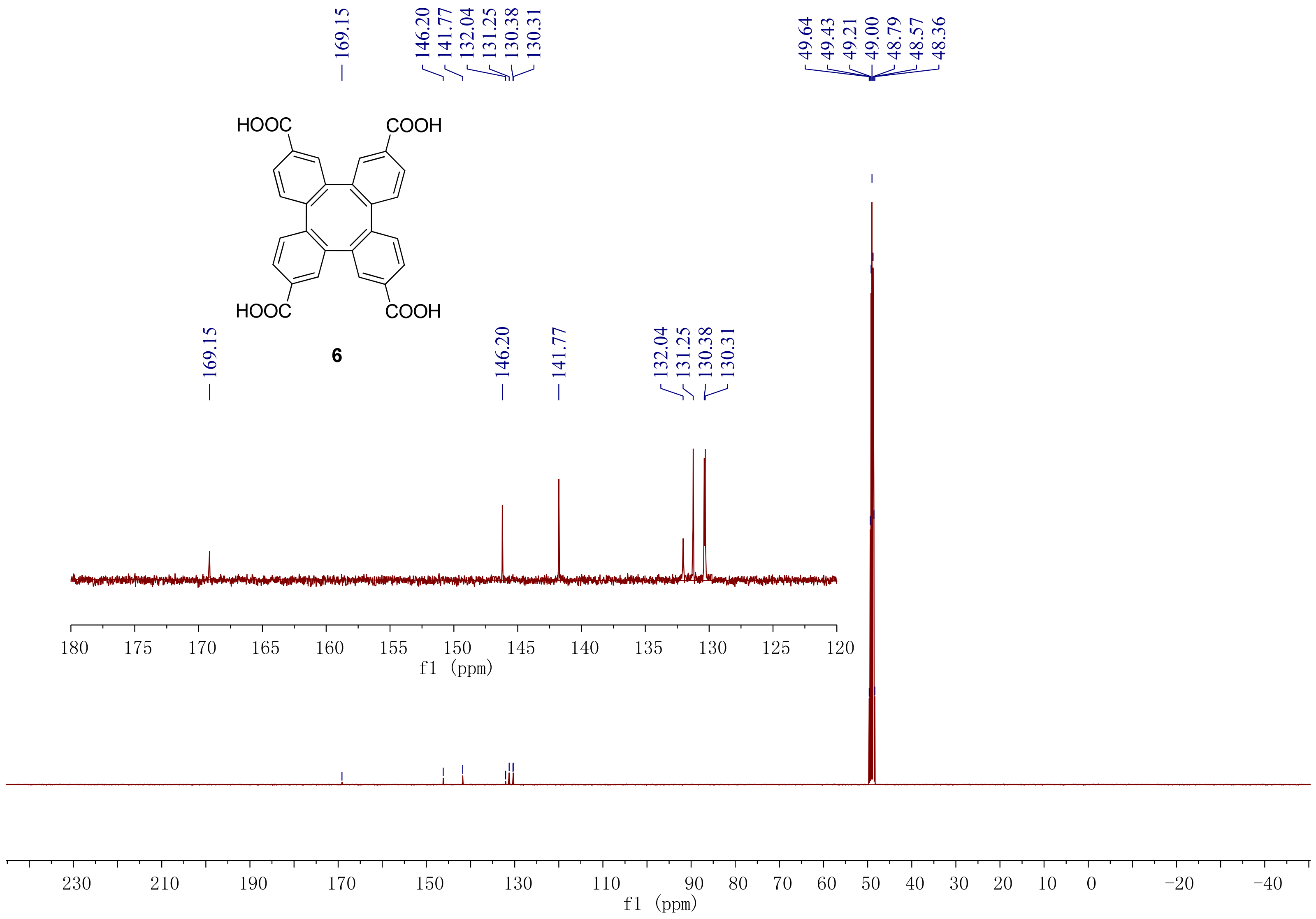
169.15
146.20
141.77
132.04
131.25
130.38
130.31
49.64
49.43
49.21
49.00
48.79
48.57
48.36
HOOC
COOH
HOOC
COOH
6
180 175 170 165 160 155 150 145 140 135 130 125 120
f1 (ppm)
230 210 190 170 150 130 110 90 80 70 60 50 40 30 20 10 0 −20 −40
f1 (ppm)

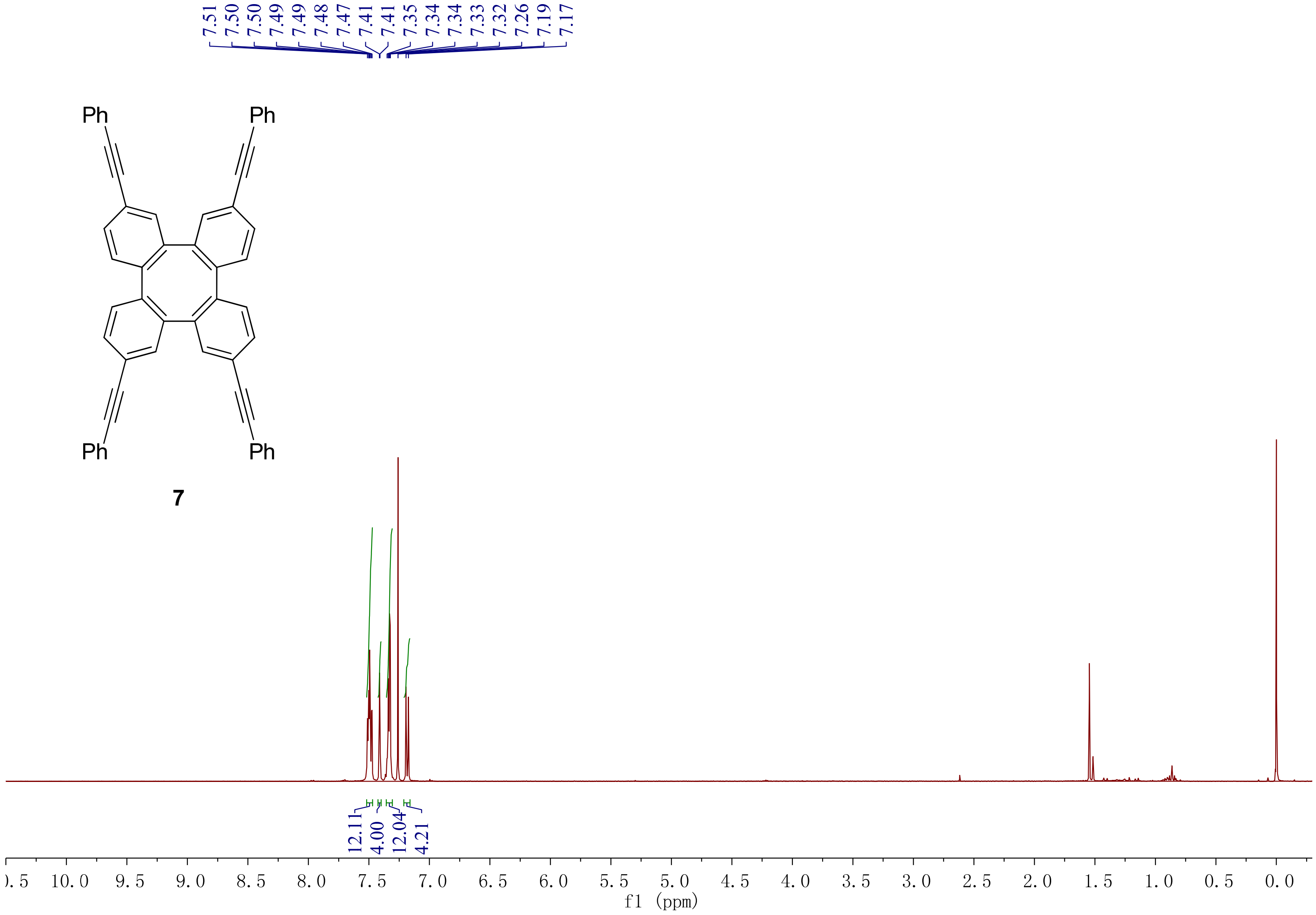
Ph
Ph
Ph
Ph
7.51
7.50
7.50
7.49
7.49
7.48
7.47
7.41
7.41
7.35
7.34
7.34
7.33
7.32
7.26
7.19
7.17
12.11
4.00
12.04
4.21
).5
10.0
9.5
9.0
8.5
8.0
7.5
7.0
6.5
6.0
5.5
5.0
4.5
4.0
3.5
3.0
2.5
2.0
1.5
1.0
0.5
0.0
f1 (ppm)

7

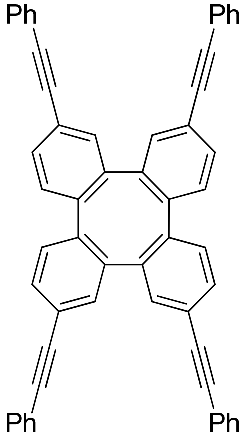
Ph
Ph
Ph
Ph

7

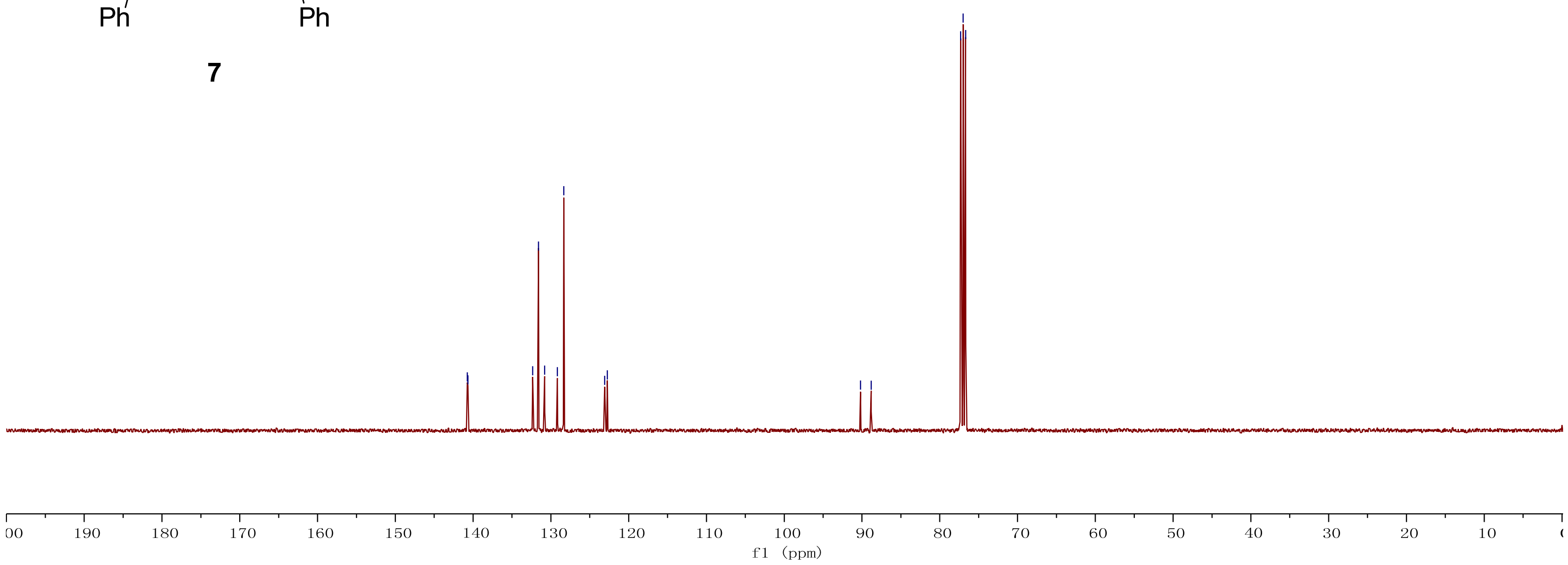
140.75
140.67
132.35
131.60
130.80
129.16
128.33
123.09
122.74
90.20
88.82
77.32
77.00
76.68
f1 (ppm)

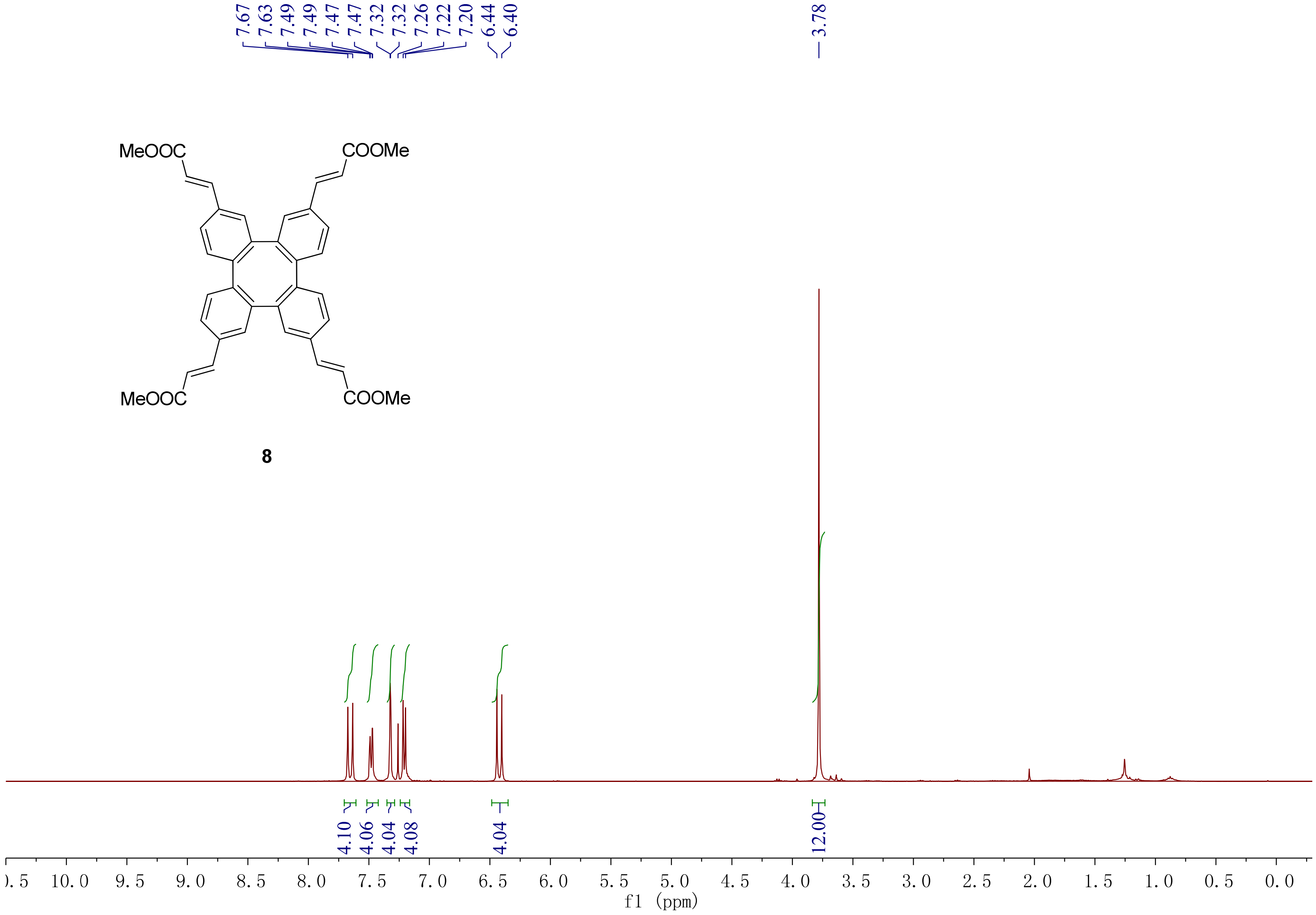
MeOOC
COOMe
MeOOC
COOMe
8
7.67
7.63
7.49
7.49
7.47
7.47
7.32
7.32
7.26
7.22
7.20
6.44
6.40
3.78
4.10
4.06
4.04
4.08
4.04
12.00
).5 10.0 9.5 9.0 8.5 8.0 7.5 7.0 6.5 6.0 5.5 5.0 4.5 4.0 3.5 3.0 2.5 2.0 1.5 1.0 0.5 0.0
f1 (ppm)

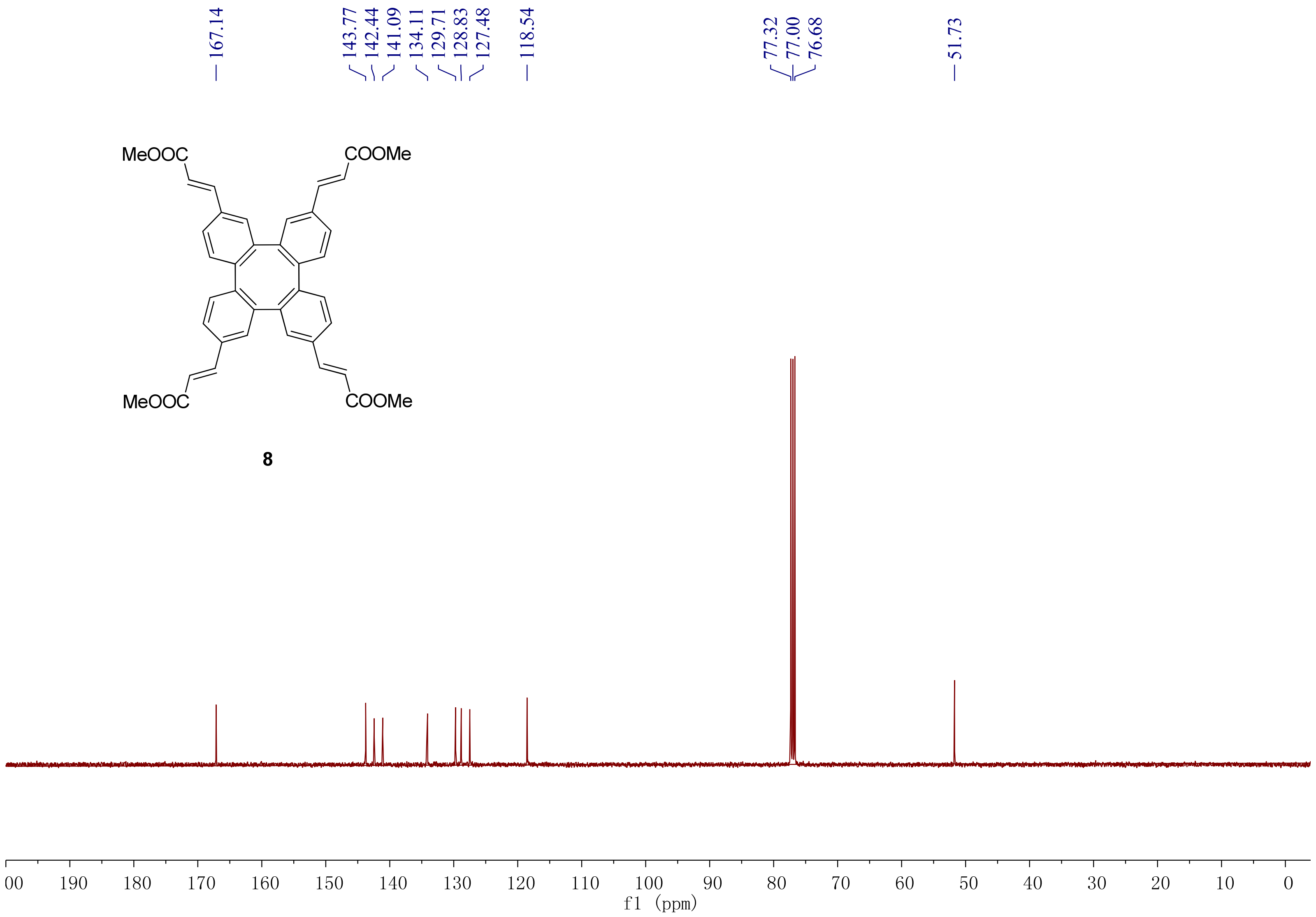
167.14
143.77
142.44
141.09
134.11
129.71
128.83
127.48
118.54
77.32
77.00
76.68
51.73
MeOOC
COOMe
MeOOC
COOMe
00
190
180
170
160
150
140
130
120
110
100
90
80
70
60
50
40
30
20
10
0
f1 (ppm)

8

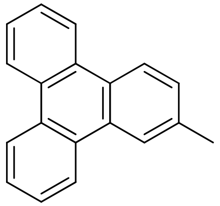

11

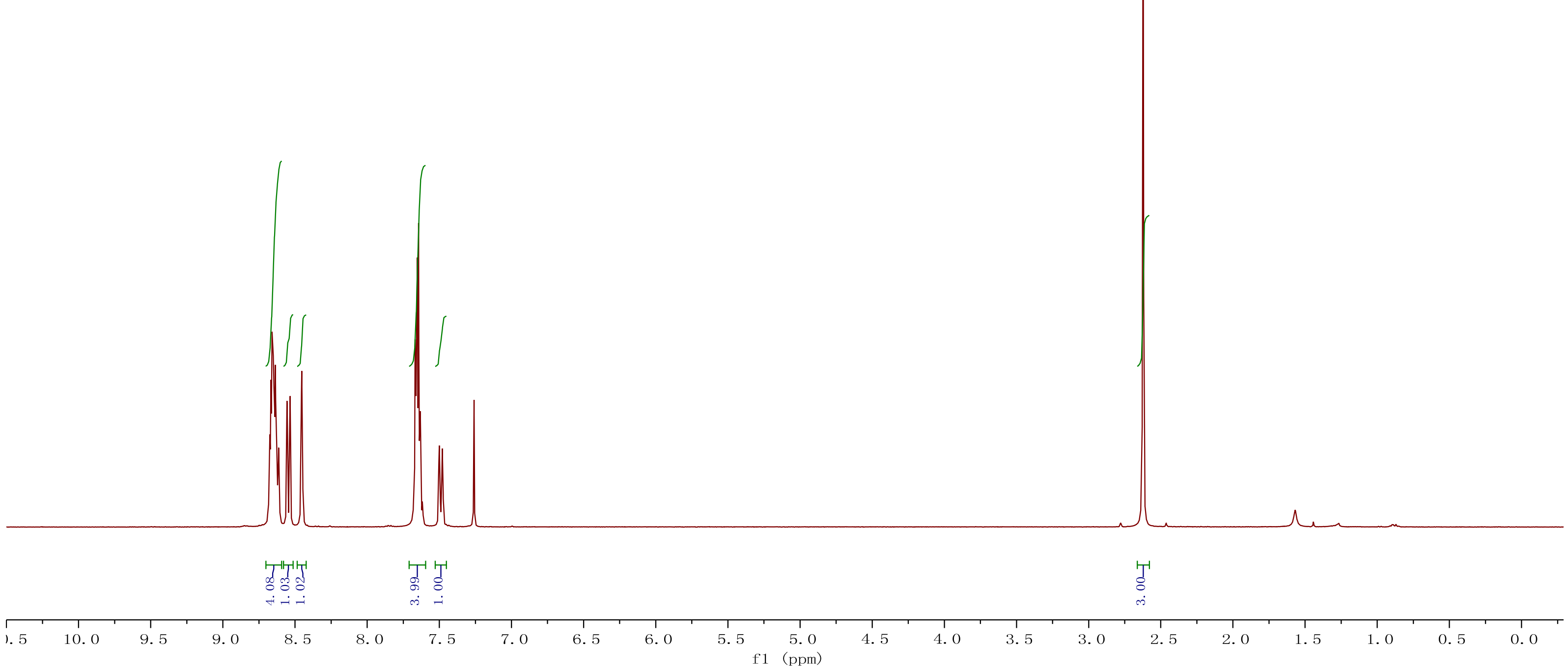
8.67
8.67
8.66
8.65
8.64
8.64
8.63
8.63
8.62
8.61
8.56
8.53
8.45
7.67
7.66
7.65
7.64
7.63
7.63
7.50
7.50
7.48
7.48
7.26
2.62
4.08
1.03
1.02
3.99
1.00
3.00
10.0
9.5
9.0
8.5
8.0
7.5
7.0
6.5
6.0
5.5
5.0
4.5
4.0
3.5
3.0
2.5
2.0
1.5
1.0
0.5
0.0
f1 (ppm)

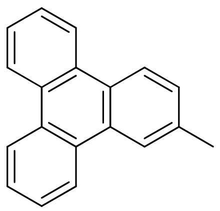

11

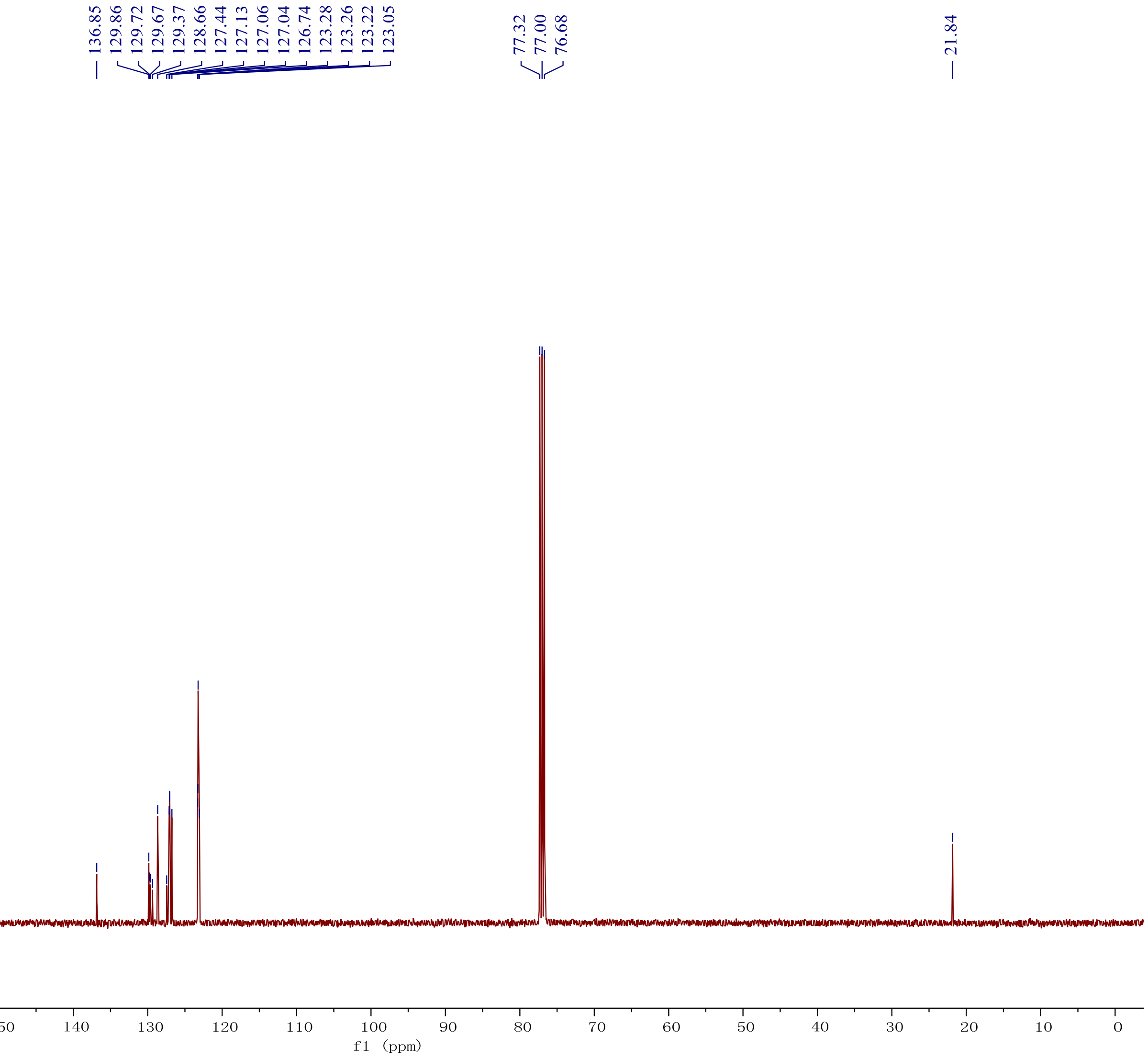
136.85
129.86
129.72
129.67
129.37
128.66
127.44
127.13
127.06
127.04
126.74
123.28
123.26
123.22
123.05
77.32
77.00
76.68
21.84
00
190
180
170
160
150
140
130
120
110
100
90
80
70
60
50
40
30
20
10
0
f1 (ppm)

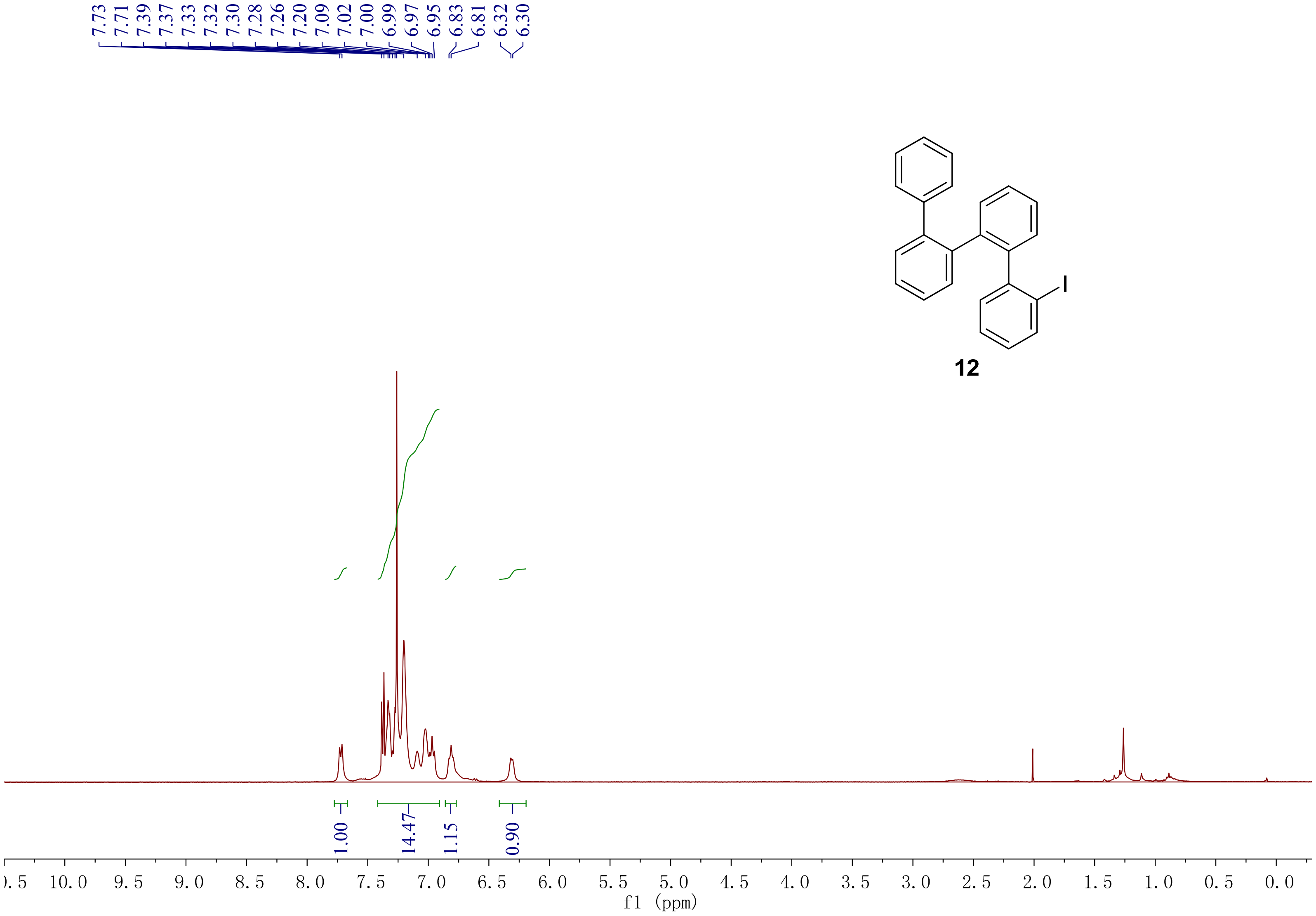
7.73
7.71
7.39
7.37
7.33
7.32
7.30
7.28
7.26
7.20
7.09
7.02
7.00
6.99
6.97
6.95
6.83
6.81
6.32
6.30
1.00
14.47
1.15
0.90
10.0 9.5 9.0 8.5 8.0 7.5 7.0 6.5 6.0 5.5 5.0 4.5 4.0 3.5 3.0 2.5 2.0 1.5 1.0 0.5 0.0
f1 (ppm)
I

12

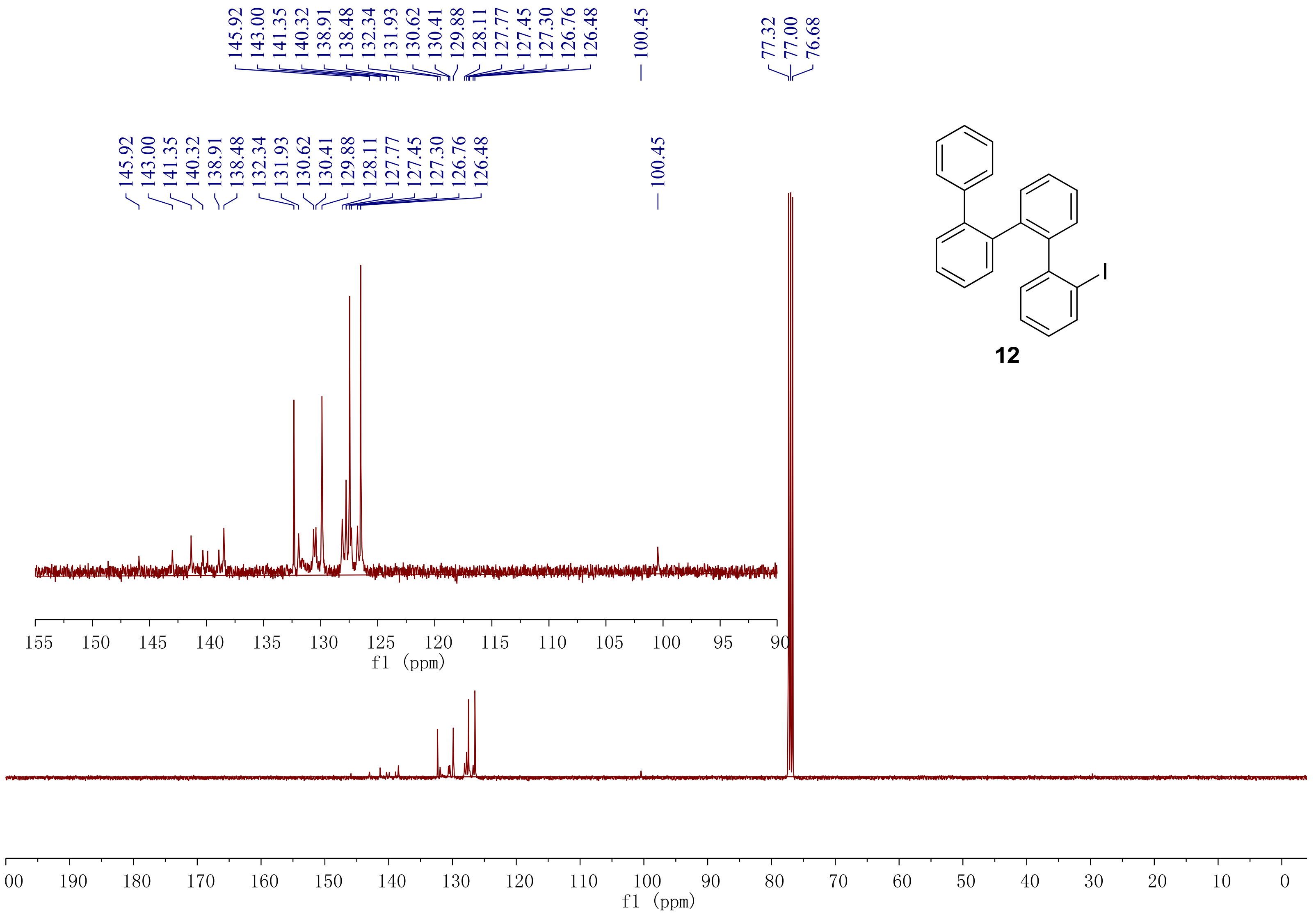
145.92
143.00
141.35
140.32
138.91
138.48
132.34
131.93
130.62
130.41
129.88
128.11
127.77
127.45
127.30
126.76
126.48
100.45
77.32
77.00
76.68
12
f1 (ppm)
f1 (ppm)
